# Supplementary material for: Global, regional, and national burden of HIV and other sexually transmitted infections among women of childbearing age from 1990 to 2021
Source: Microbiol Spectr. 2025 Oct 24;13(12):e00488-25. doi: 10.1128/spectrum.00488-25 (PMC12671144; doi:10.1128/spectrum.00488-25)
Supplement: Supplemental figures — Fig. S1 to S4. [file spectrum.00488-25-s0001.docx]

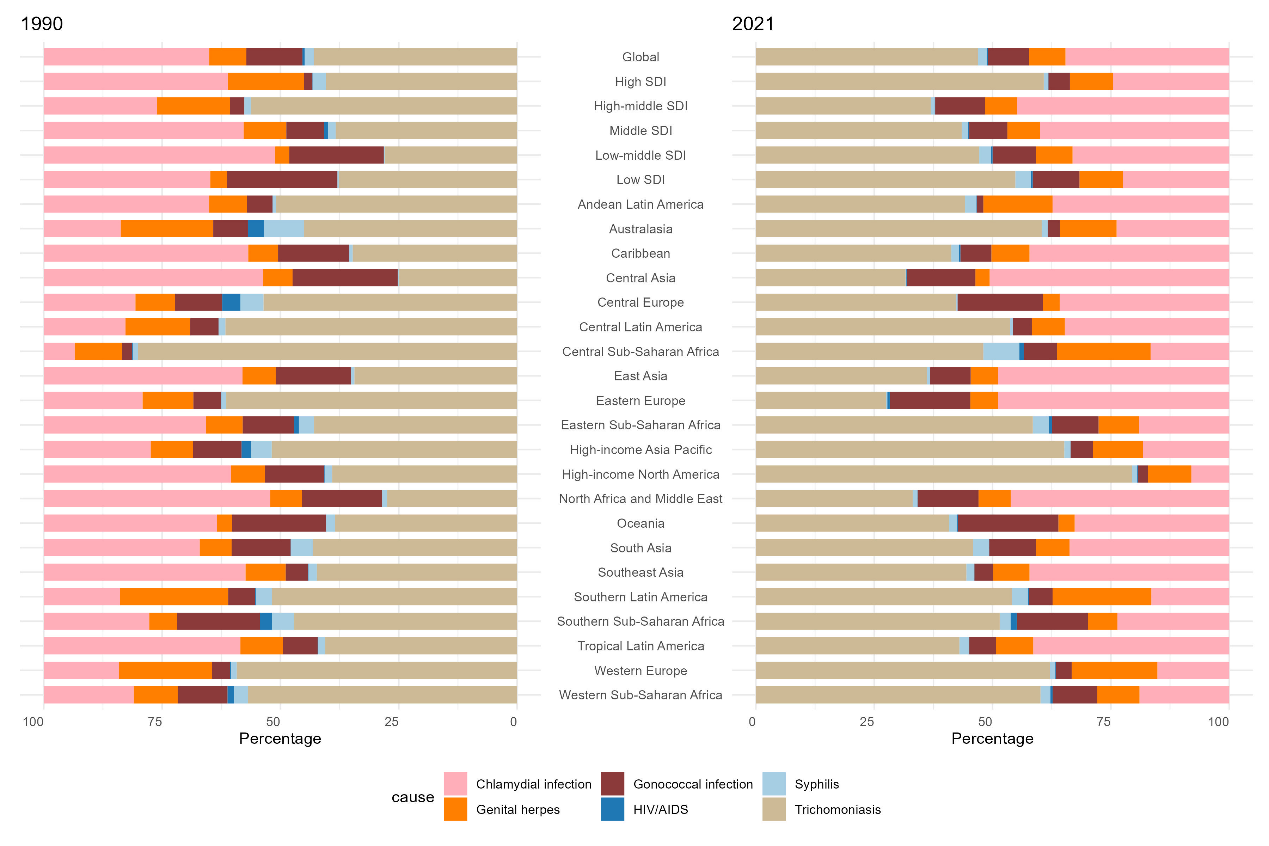


**Figure S1:** Incidence proportion of STIs.


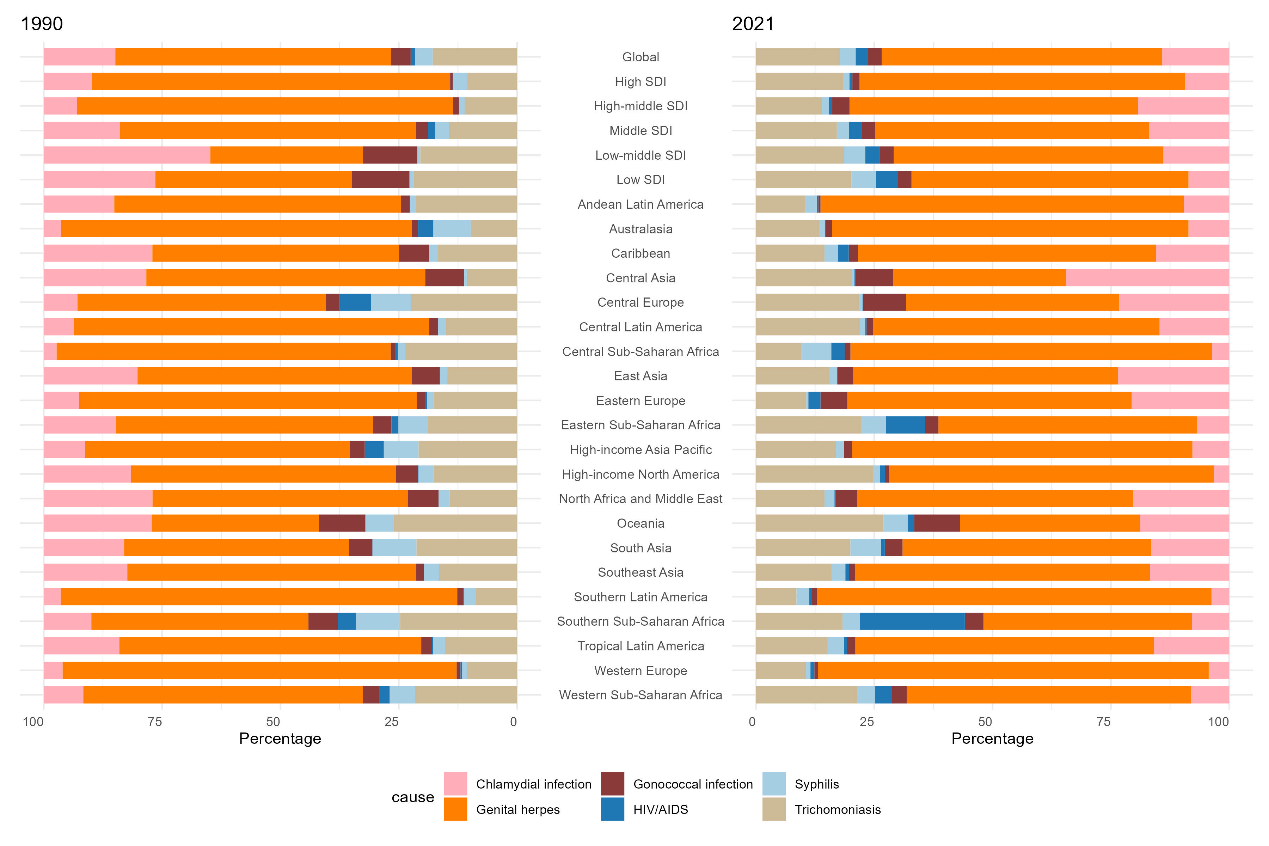


**Figure S2:** Prevalence proportion of STIs.


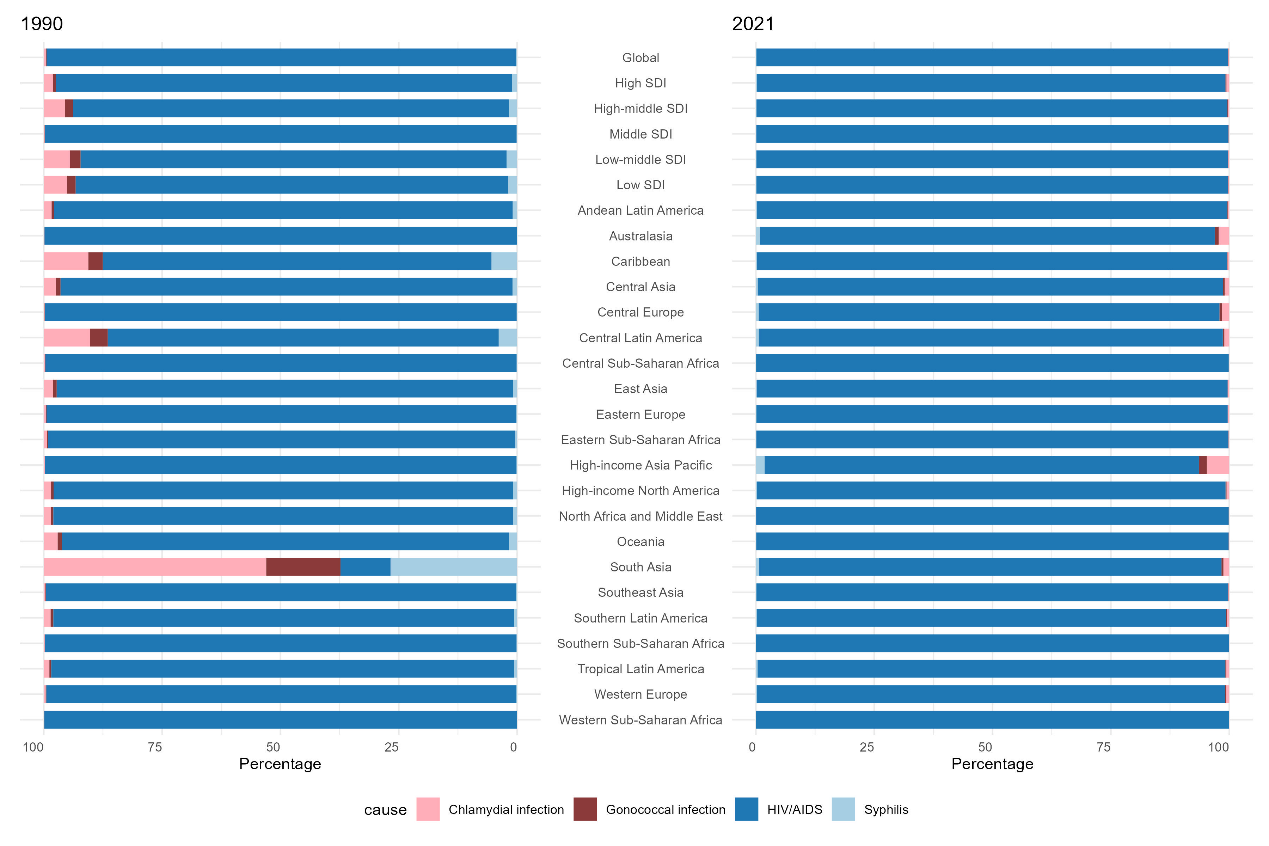


**Figure S3:** Deaths proportion of STIs.


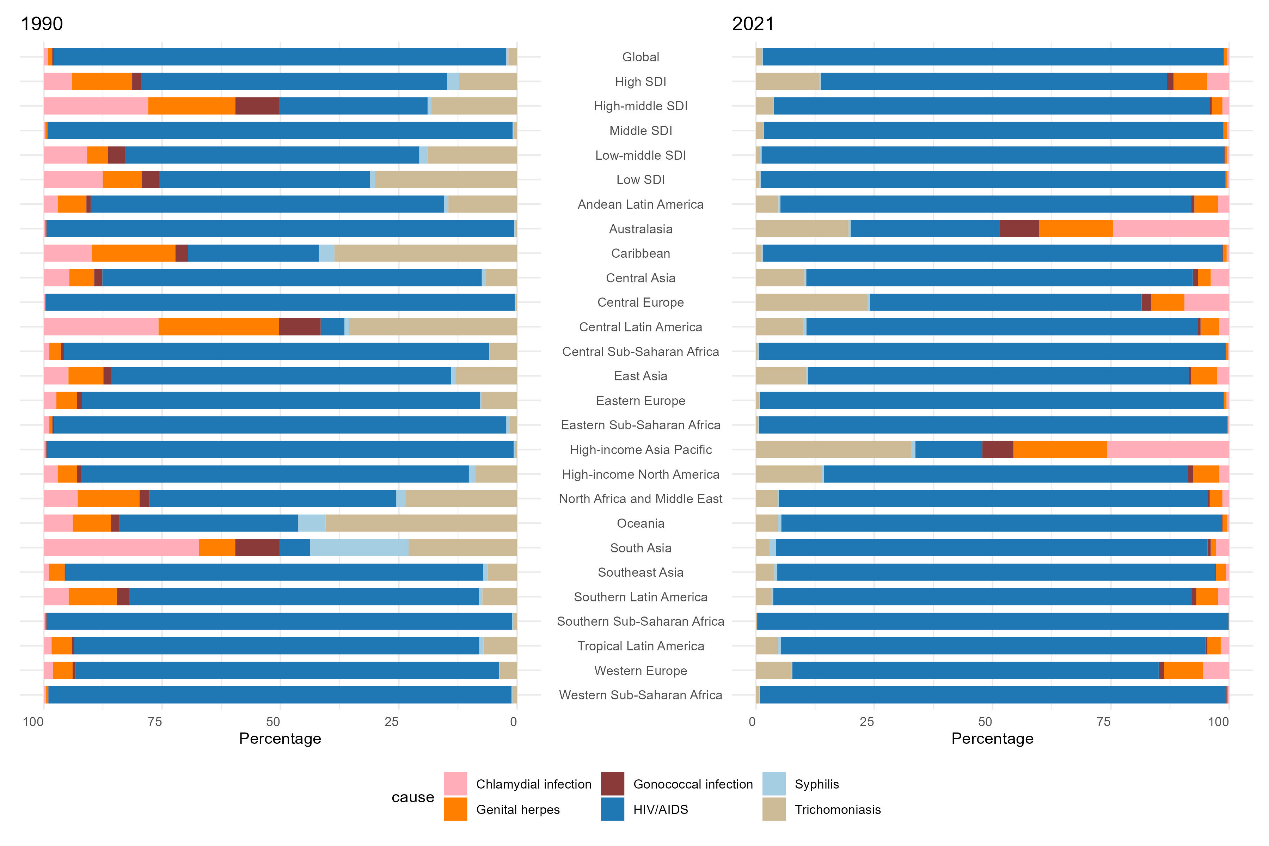


**Figure S4:** DALYs proportion of STIs.
