# Supplementary material for: Global, regional, and national burden of HIV and other sexually transmitted infections among women of childbearing age from 1990 to 2021
Source: Microbiol Spectr. 2025 Oct 24;13(12):e00488-25. doi: 10.1128/spectrum.00488-25 (PMC12671144; doi:10.1128/spectrum.00488-25)
Supplement: Table S1 — The age-standardized incidence, prevalence, mortality, and DALY rates at global and regional levels in 2021. [file spectrum.00488-25-s0002.docx]

**Table 1**: The age-standardized incidence, prevalence, mortality and DALYs rates at global and regional levels in 2021

|  |  | HIV/AIDS | Syphilis | Chlamydial infection | Gonococcal infection | Trichomoniasis | Genital herpes |
| --- | --- | --- | --- | --- | --- | --- | --- |
| Age-standardized Incidence | Global | 36.99  (32.06 to 43.14) | 299.02  (158.38 to 489.74) | 5179.06  (2938.63 to 8417.46) | 1430.11  (857.81 to 2239.55) | 6709.73  (3676.25 to 10839.25) | 1222.71  (861.48 to 1650.15) |
|  | High SDI | 9.83  (5.10 to 15.14) | 96.00  (49.84 to 160.75) | 2410.07  (1360.39 to 3905.41) | 516.55  (306.97 to 818.31) | 5400.12  (2927.91 to 8877.08) | 985.72  (674.91 to 1360.93) |
|  | High-middle SDI | 17.79  (11.81 to 28.94) | 120.01  (62.42 to 199.57) | 5786.30  (3276.39 to 9483.17) | 1826.12  (1086.35 to 2898.26) | 4333.29  (2342.16 to 7123.39) | 989.83  (681.76 to 1353.71) |
|  | Middle SDI | 32.76  (27.00 to 38.83) | 247.56  (130.55 to 406.07) | 6528.02  (3718.26 to 10605.95) | 1517.43  (825.50 to 2583.10) | 6657.49  (3624.23 to 10858.91) | 1222.15  (855.69 to 1655.72) |
|  | Low-middle SDI | 41.37  (33.71 to 51.50) | 360.37  (189.70 to 592.80) | 4722.69  (2672.83 to 7680.48) | 1306.93  (782.47 to 2031.37) | 6553.52  (3585.91 to 10568.38) | 1117.00  (788.24 to 1506.60) |
|  | Low SDI | 78.62  (57.48 to 107.11) | 604.30  (322.66 to 992.33) | 4473.43  (2505.55 to 7285.43) | 1738.48  (1020.04 to 2797.89) | 11107.32  (6223.08 to 17653.05) | 1717.48  (1235.32 to 2279.80) |
|  | East Asia | 1.73  (0.79 to 3.05) | 112.28  (57.32 to 190.60) | 7034.52  (3986.95 to 11589.38) | 1711.09  (816.69 to 3110.15) | 4752.44  (2549.16 to 7856.46) | 923.73  (630.34 to 1284.40) |
|  | Central Asia | 30.27  (15.92 to 58.60) | 47.98  (24.99 to 79.99) | 12970.31  (7476.95 to 20539.46) | 4378.55  (2226.91 to 7808.52) | 7630.33  (4222.23 to 12345.22) | 785.45  (534.13 to 1090.79) |
|  | South Asia | 7.01  (3.94 to 14.21) | 304.84  (159.95 to 501.40) | 3004.53  (1671.46 to 4993.97) | 881.60  (444.47 to 1584.85) | 3902.92  (2087.26 to 6484.89) | 630.21  (425.75 to 873.69) |
|  | Southeast Asia | 15.07  (10.20 to 22.11) | 244.56  (126.94 to 407.82) | 6042.39  (3417.89 to 9855.43) | 620.55  (366.85 to 979.11) | 5973.26  (3245.15 to 9774.51) | 1174.99  (813.30 to 1608.94) |
|  | High-income Asia Pacific | 1.16  (0.68 to 1.75) | 114.02  (59.08 to 189.99) | 1427.12  (781.02 to 2357.17) | 411.61  (239.30 to 662.97) | 4491.62  (2417.79 to 7442.39) | 897.52  (607.99 to 1251.90) |
|  | Central Europe | 2.16  (1.15 to 3.73) | 44.68  (23.09 to 75.28) | 5191.49  (2925.81 to 8489.15) | 3592.60  (2134.85 to 5719.68) | 5373.54  (2951.33 to 8844.99) | 535.92  (359.77 to 752.12) |
|  | Eastern Europe | 89.95  (56.67 to 153.67) | 43.51  (22.35 to 72.52) | 7602.20  (4326.39 to 12370.47) | 3895.94  (1957.64 to 6898.63) | 3781.24  (2024.45 to 6302.06) | 1127.87  (786.46 to 1534.72) |
|  | Western Europe | 7.10  (5.00 to 9.49) | 46.42  (23.86 to 77.47) | 614.13  (334.39 to 1014.87) | 138.14  (77.93 to 228.97) | 2195.98  (1192.11 to 3582.40) | 777.58  (526.61 to 1078.11) |
|  | Southern Latin America | 15.91  (11.94 to 20.74) | 298.86  (157.68 to 498.01) | 1329.50  (726.51 to 2219.87) | 428.38  (211.72 to 788.35) | 4134.95  (2269.51 to 6833.73) | 1869.18  (1300.52 to 2542.73) |
|  | High-income North America | 20.04  (8.00 to 33.28) | 129.04  (67.12 to 215.68) | 978.16  (531.62 to 1615.46) | 268.86  (130.42 to 488.46) | 8775.78  (4757.36 to 14420.87) | 1185.17  (813.43 to 1635.03) |
|  | Andean Latin America | 12.48  (7.55 to 20.42) | 384.27  (203.83 to 636.95) | 5652.66  (3119.46 to 9319.05) | 223.56  (109.76 to 408.09) | 6358.65  (3521.89 to 10441.19) | 2355.26  (1678.80 to 3148.57) |
|  | Central Latin America | 10.99  (6.71 to 17.03) | 159.33  (82.54 to 265.75) | 9240.77  (5291.18 to 14815.60) | 1138.59  (679.51 to 1777.38) | 13457.77  (7446.78 to 21755.65) | 1950.75  (1360.60 to 2629.87) |
|  | Tropical Latin America | 29.34  (15.71 to 51.51) | 543.45  (303.32 to 849.82) | 10564.02  (6051.61 to 17161.70) | 1666.83  (805.89 to 3026.74) | 9976.98  (5395.81 to 16351.27) | 2209.92  (1565.10 to 2960.69) |
|  | Central Sub-Saharan Africa | 155.37  (93.55 to 253.89) | 1210.05  (636.64 to 1997.65) | 2837.60  (1577.99 to 4725.88) | 1065.27  (514.85 to 1933.69) | 8399.86  (4685.02 to 13627.49) | 3099.41  (2294.47 to 4050.97) |
|  | Eastern Sub-Saharan Africa | 179.28  (128.73 to 253.59) | 847.27  (456.17 to 1384.69) | 5137.68  (2881.00 to 8358.67) | 2304.01  (1352.71 to 3709.47) | 16274.75  (9168.32 to 25791.41) | 2160.29  (1548.17 to 2865.02) |
|  | Southern Sub-Saharan Africa | 575.79  (439.93 to 730.32) | 1030.06  (538.12 to 1709.06) | 10155.85  (5821.89 to 16169.24) | 6937.92  (3816.51 to 11471.26) | 21438.37  (12145.47 to 33847.43) | 2845.49  (2097.76 to 3698.32) |
|  | Western Sub-Saharan Africa | 112.89  (90.76 to 139.09) | 501.87  (261.48 to 835.05) | 4855.51  (2729.72 to 7911.90) | 2101.37  (1263.76 to 3273.82) | 15844.89  (8912.27 to 25215.41) | 2172.89  (1547.72 to 2899.03) |
|  | North Africa and Middle East | 7.38  (2.44 to 22.51) | 141.46  (72.21 to 241.02) | 6425.24  (3661.39 to 10291.00) | 1913.72  (1031.60 to 3291.25) | 4342.92  (2373.84 to 7062.83) | 967.29  (657.30 to 1341.34) |
|  | Caribbean | 72.46  (36.98 to 128.17) | 393.00  (204.45 to 656.20) | 9778.42  (5577.09 to 15754.03) | 1639.14  (826.18 to 2957.04) | 8932.23  (4986.62 to 14348.42) | 1979.23  (1394.70 to 2669.12) |
|  | Oceania | 60.01  (28.72 to 103.72) | 704.00  (365.67 to 1195.66) | 13457.05  (7805.12 to 21350.70) | 8770.75  (4521.47 to 15285.40) | 16633.99  (9468.40 to 26070.46) | 1439.93  (989.21 to 1980.73) |
|  | Australasia | 2.95  (1.33 to 5.35) | 90.21  (46.83 to 152.22) | 1625.06  (891.48 to 2682.75) | 193.39  (105.78 to 326.41) | 3735.27  (2041.89 to 6104.91) | 913.74  (627.08 to 1264.62) |
| Age-standardized Prevalence | Global | 804.77  (759.54 to 858.92) | 1121.17  (684.72 to 1721.54) | 4570.25  (2705.08 to 7271.25) | 1008.52  (625.98 to 1548.20) | 5552.21  (2918.06 to 9234.17) | 17137.09  (13485.32 to 21121.75) |
|  | High SDI | 126.55  (73.59 to 184.60) | 338.45  (195.84 to 545.52) | 2229.55  (1349.84 to 3477.16) | 406.89  (262.47 to 607.73) | 4081.60  (2097.67 to 6962.12) | 13874.07  (10777.78 to 17468.22) |
|  | High-middle SDI | 170.39  (122.71 to 243.42) | 442.19  (261.61 to 696.68) | 5001.39  (2913.01 to 8076.50) | 1254.56  (761.47 to 1969.08) | 3351.84  (1712.41 to 5755.60) | 13348.01  (10317.95 to 16694.09) |
|  | Middle SDI | 844.85  (786.73 to 910.26) | 934.67  (570.43 to 1434.69) | 5653.88  (3320.64 to 9048.29) | 1050.72  (589.13 to 1760.48) | 5421.04  (2823.74 to 9069.76) | 16945.25  (13253.16 to 21013.92) |
|  | Low-middle SDI | 833.00  (768.82 to 899.29) | 1369.15  (839.58 to 2100.74) | 4242.16  (2532.74 to 6710.83) | 933.55  (584.22 to 1417.06) | 5521.08  (2914.24 to 9153.03) | 15970.48  (12604.07 to 19638.21) |
|  | Low SDI | 2120.09  (1914.17 to 2385.76) | 2382.26  (1461.20 to 3634.69) | 4124.15  (2487.40 to 6468.83) | 1259.53  (781.24 to 1964.32) | 9601.68  (5162.30 to 15593.01) | 27150.83  (21795.76 to 32865.60) |
|  | East Asia | 19.35  (9.59 to 37.62) | 428.96  (249.40 to 684.25) | 5982.02  (3442.14 to 9774.68) | 1160.52  (565.68 to 2094.27) | 3705.02  (1860.91 to 6448.79) | 11624.77  (8901.94 to 14758.34) |
|  | Central Asia | 94.49  (65.64 to 148.77) | 190.78  (114.47 to 292.40) | 11119.37  (6544.42 to 17422.35) | 2990.84  (1558.12 to 5277.78) | 6190.79  (3254.84 to 10300.40) | 10079.02  (7682.27 to 12851.02) |
|  | South Asia | 126.98  (98.99 to 174.35) | 1115.55  (677.09 to 1736.80) | 2822.41  (1712.69 to 4486.25) | 642.77  (350.46 to 1112.76) | 3276.79  (1654.08 to 5668.72) | 7939.34  (6023.65 to 10110.84) |
|  | Southeast Asia | 214.99  (153.97 to 302.92) | 938.14  (564.48 to 1465.85) | 5128.61  (2942.35 to 8303.78) | 427.66  (258.43 to 666.43) | 4578.96  (2372.12 to 7669.72) | 16941.30  (13245.64 to 21111.74) |
|  | High-income Asia Pacific | 14.58  (9.02 to 20.96) | 383.04  (220.54 to 621.45) | 1639.37  (1036.36 to 2454.75) | 384.07  (239.70 to 562.03) | 3303.83  (1691.51 to 5693.20) | 12081.03  (9317.02 to 15402.48) |
|  | Central Europe | 31.28  (18.98 to 48.23) | 155.62  (92.19 to 246.05) | 4598.79  (2709.65 to 7339.93) | 2441.65  (1470.97 to 3860.77) | 3819.87  (1983.86 to 6475.75) | 6867.17  (5189.27 to 8782.68) |
|  | Eastern Europe | 797.29  (545.54 to 1209.31) | 155.63  (90.07 to 248.78) | 6576.06  (3850.03 to 10557.31) | 2641.32  (1350.93 to 4642.90) | 3070.67  (1556.85 to 5331.23) | 16395.98  (12764.83 to 20426.27) |
|  | Western Europe | 108.59  (79.62 to 137.68) | 156.75  (91.18 to 250.16) | 701.08  (457.67 to 1038.47) | 128.32  (85.56 to 189.57) | 1585.94  (804.36 to 2718.60) | 11192.35  (8623.57 to 14111.27) |
|  | Southern Latin America | 203.92  (169.03 to 246.36) | 1103.41  (644.26 to 1745.72) | 1440.31  (928.18 to 2182.01) | 427.37  (273.28 to 669.35) | 3125.31  (1602.84 to 5349.26) | 28790.69  (22804.79 to 35511.95) |
|  | High-income North America | 263.81  (134.92 to 409.42) | 448.52  (259.08 to 723.03) | 969.35  (589.56 to 1504.11) | 253.08  (152.11 to 404.58) | 6716.16  (3447.79 to 11378.34) | 17226.06  (13425.95 to 21577.25) |
|  | Andean Latin America | 174.22  (121.01 to 249.86) | 1349.74  (787.47 to 2147.31) | 5097.79  (2965.84 to 8160.60) | 249.41  (143.92 to 382.30) | 5183.46  (2694.32 to 8701.66) | 37151.84  (29730.61 to 45086.38) |
|  | Central Latin America | 141.58  (92.06 to 207.02) | 592.42  (347.30 to 936.15) | 7937.70  (4642.97 to 12586.95) | 805.42  (497.73 to 1231.36) | 11198.36  (5991.45 to 18178.55) | 28920.41  (22773.87 to 35510.66) |
|  | Tropical Latin America | 338.99  (193.44 to 546.65) | 2067.56  (1400.09 to 2928.34) | 9204.93  (5442.40 to 14688.47) | 1192.41  (619.98 to 2099.98) | 8052.90  (4160.86 to 13590.98) | 31257.78  (24750.57 to 38343.48) |
|  | Central Sub-Saharan Africa | 2099.06  (1717.15 to 2544.07) | 4689.77  (2830.28 to 7249.79) | 2755.16  (1704.63 to 4316.21) | 851.28  (482.57 to 1430.65) | 7286.72  (3884.71 to 12240.34) | 56745.62  (47554.16 to 66194.76) |
|  | Eastern Sub-Saharan Africa | 5420.45  (4875.89 to 6120.29) | 3353.57  (2083.81 to 5095.66) | 4611.66  (2730.18 to 7295.21) | 1638.46  (1003.89 to 2573.86) | 15034.96  (8185.95 to 24147.90) | 35989.40  (28907.08 to 43474.94) |
|  | Southern Sub-Saharan Africa | 22745.44  (21536.92 to 24099.54) | 4196.66  (2535.17 to 6488.64) | 8847.22  (5233.23 to 13868.72) | 4738.98  (2655.23 to 7759.82) | 19947.23  (10974.86 to 31275.60) | 46257.68  (37797.83 to 54689.63) |
|  | Western Sub-Saharan Africa | 2075.85  (1868.14 to 2293.44) | 2043.73  (1235.49 to 3131.23) | 4693.08  (2918.48 to 7253.03) | 1543.23  (981.14 to 2324.83) | 12228.66  (6608.20 to 19696.22) | 33752.91  (26934.75 to 41075.08) |
|  | North Africa and Middle East | 59.02  (30.27 to 125.58) | 574.43  (334.02 to 901.48) | 5521.99  (3215.90 to 8743.22) | 1315.99  (726.41 to 2235.85) | 3761.77  (1951.28 to 6384.73) | 14007.08  (10837.19 to 17548.77) |
|  | Caribbean | 1142.09  (873.58 to 1421.47) | 1579.32  (962.62 to 2416.88) | 8379.84  (4879.08 to 13359.99) | 1158.33  (616.30 to 2035.58) | 7264.03  (3823.33 to 12016.09) | 30186.93  (23838.77 to 37070.23) |
|  | Oceania | 773.26  (588.60 to 984.46) | 3127.32  (1876.94 to 4839.56) | 11416.49  (6706.78 to 17990.07) | 5890.13  (3057.80 to 10233.65) | 16113.81  (8842.88 to 25826.93) | 21711.95  (16888.90 to 27094.86) |
|  | Australasia | 34.36  (17.54 to 57.05) | 290.92  (166.14 to 472.65) | 1848.97  (1102.28 to 2807.61) | 287.75  (154.75 to 443.32) | 2653.55  (1348.31 to 4603.07) | 14122.20  (10904.93 to 17726.85) |
| Age-standardized Mortality | Global | 12.98  (10.04 to 16.84) | 0.014  (0.008 to 0.020) | 0.025  (0.015 to 0.037) | 0.009  (0.005 to 0.013) | NA | NA |
|  | High SDI | 0.53  (0.53 to 0.53) | 0.001  (0.001 to 0.001) | 0.003  (0.003 to 0.003) | 0.001  (0.001 to 0.001) | NA | NA |
|  | High-middle SDI | 2.72  (2.60 to 3.00) | 0.003  (0.003 to 0.004) | 0.007  (0.006 to 0.009) | 0.003  (0.002 to 0.003) | NA | NA |
|  | Middle SDI | 10.05  (9.09 to 11.23) | 0.008  (0.006 to 0.010) | 0.014  (0.010 to 0.017) | 0.005  (0.004 to 0.006) | NA | NA |
|  | Low-middle SDI | 17.48  (11.94 to 24.37) | 0.022  (0.012 to 0.031) | 0.037  (0.021 to 0.054) | 0.013  (0.007 to 0.018) | NA | NA |
|  | Low SDI | 36.42  (26.08 to 50.48) | 0.035  (0.018 to 0.063) | 0.063  (0.032 to 0.116) | 0.022  (0.011 to 0.039) | NA | NA |
|  | East Asia | 0.89  (0.65 to 1.15) | 0.001  (0.001 to 0.002) | 0.002  (0.001 to 0.003) | 0.001  (0.000 to 0.001) | NA | NA |
|  | Central Asia | 1.56  (1.55 to 1.56) | 0.006  (0.004 to 0.009) | 0.015  (0.011 to 0.023) | 0.006  (0.004 to 0.010) | NA | NA |
|  | South Asia | 3.17  (1.83 to 5.79) | 0.024  (0.013 to 0.035) | 0.042  (0.022 to 0.060) | 0.014  (0.007 to 0.020) | NA | NA |
|  | Southeast Asia | 3.27  (2.69 to 4.07) | 0.004  (0.002 to 0.005) | 0.006  (0.004 to 0.009) | 0.002  (0.001 to 0.003) | NA | NA |
|  | High-income Asia Pacific | 0.03  (0.03 to 0.03) | 0.001  (0.000 to 0.001) | 0.001  (0.001 to 0.002) | 0.001  (0.000 to 0.001) | NA | NA |
|  | Central Europe | 0.30  (0.30 to 0.30) | 0.001  (0.001 to 0.002) | 0.003  (0.003 to 0.005) | 0.001  (0.001 to 0.002) | NA | NA |
|  | Eastern Europe | 12.54  (12.51 to 12.57) | 0.010  (0.008 to 0.012) | 0.027  (0.023 to 0.033) | 0.011  (0.009 to 0.013) | NA | NA |
|  | Western Europe | 0.36  (0.36 to 0.36) | 0.001  (0.001 to 0.001) | 0.002  (0.002 to 0.002) | 0.001  (0.001 to 0.001) | NA | NA |
|  | Southern Latin America | 2.42  (2.41 to 2.43) | 0.005  (0.004 to 0.007) | 0.013  (0.010 to 0.016) | 0.005  (0.004 to 0.007) | NA | NA |
|  | High-income North America | 0.75  (0.75 to 0.75) | 0.002  (0.002 to 0.002) | 0.005  (0.004 to 0.005) | 0.002  (0.002 to 0.002) | NA | NA |
|  | Andean Latin America | 2.95  (2.68 to 3.41) | 0.005  (0.003 to 0.009) | 0.010  (0.005 to 0.016) | 0.003  (0.002 to 0.006) | NA | NA |
|  | Central Latin America | 2.86  (2.86 to 2.87) | 0.017  (0.014 to 0.023) | 0.030  (0.024 to 0.039) | 0.010  (0.008 to 0.013) | NA | NA |
|  | Tropical Latin America | 4.47  (4.46 to 4.47) | 0.017  (0.016 to 0.019) | 0.030  (0.027 to 0.034) | 0.010  (0.009 to 0.011) | NA | NA |
|  | Central Sub-Saharan Africa | 55.57  (35.58 to 81.35) | 0.026  (0.011 to 0.055) | 0.045  (0.020 to 0.095) | 0.015  (0.007 to 0.032) | NA | NA |
|  | Eastern Sub-Saharan Africa | 87.34  (60.62 to 124.72) | 0.059  (0.030 to 0.127) | 0.112  (0.055 to 0.237) | 0.038  (0.019 to 0.081) | NA | NA |
|  | Southern Sub-Saharan Africa | 230.12  (195.75 to 278.99) | 0.027  (0.018 to 0.049) | 0.047  (0.030 to 0.086) | 0.016  (0.010 to 0.029) | NA | NA |
|  | Western Sub-Saharan Africa | 50.69  (34.49 to 72.60) | 0.010  (0.004 to 0.015) | 0.018  (0.007 to 0.026) | 0.006  (0.003 to 0.010) | NA | NA |
|  | North Africa and Middle East | 2.30  (1.34 to 4.74) | 0.001  (0.001 to 0.002) | 0.002  (0.001 to 0.004) | 0.001  (0.000 to 0.002) | NA | NA |
|  | Caribbean | 18.20  (10.57 to 28.70) | 0.040  (0.022 to 0.067) | 0.070  (0.038 to 0.117) | 0.023  (0.013 to 0.039) | NA | NA |
|  | Oceania | 8.75  (4.28 to 15.98) | 0.004  (0.002 to 0.008) | 0.006  (0.003 to 0.014) | 0.002  (0.001 to 0.004) | NA | NA |
|  | Australasia | 0.09  (0.09 to 0.09) | 0.001  (0.001 to 0.001) | 0.002  (0.002 to 0.003) | 0.001  (0.001 to 0.001) | NA | NA |
| Age-standardized DALYs | Global | 829.89  (658.73 to 1056.91) | 2.10  (1.55 to 2.85) | 4.58  (3.09 to 6.76) | 1.24  (0.85 to 1.84) | 10.54  (3.51 to 24.28) | 4.78  (2.02 to 9.63) |
|  | High SDI | 39.20  (34.37 to 45.41) | 0.20  (0.13 to 0.34) | 2.79  (1.64 to 4.45) | 0.85  (0.51 to 1.38) | 7.75  (2.52 to 18.16) | 3.87  (1.63 to 7.82) |
|  | High-middle SDI | 165.81  (154.23 to 184.52) | 0.39  (0.29 to 0.58) | 2.61  (1.69 to 4.01) | 0.67  (0.44 to 1.04) | 6.41  (2.08 to 14.96) | 3.76  (1.59 to 7.67) |
|  | Middle SDI | 667.81  (607.37 to 742.37) | 1.22  (0.89 to 1.69) | 3.40  (2.30 to 4.99) | 0.79  (0.55 to 1.17) | 10.31  (3.39 to 23.88) | 4.73  (1.99 to 9.56) |
|  | Low-middle SDI | 1077.76  (761.94 to 1478.66) | 2.94  (2.10 to 4.00) | 5.79  (3.90 to 8.35) | 1.54  (1.05 to 2.18) | 10.46  (3.50 to 24.14) | 4.43  (1.87 to 8.93) |
|  | Low SDI | 2283.96  (1693.06 to 3099.95) | 5.61  (4.00 to 8.00) | 8.49  (5.43 to 13.34) | 2.57  (1.66 to 4.10) | 18.17  (6.13 to 41.32) | 7.43  (3.17 to 14.90) |
|  | East Asia | 52.77  (38.36 to 68.20) | 0.25  (0.15 to 0.43) | 1.70  (1.01 to 2.74) | 0.34  (0.20 to 0.58) | 7.11  (2.27 to 16.88) | 3.32  (1.39 to 6.79) |
|  | Central Asia | 93.90  (90.21 to 99.80) | 0.44  (0.33 to 0.63) | 4.66  (2.91 to 7.34) | 1.37  (0.88 to 2.13) | 11.79  (3.94 to 27.08) | 2.86  (1.19 to 5.78) |
|  | South Asia | 191.34  (114.21 to 344.42) | 2.92  (2.03 to 3.94) | 6.19  (4.14 to 8.78) | 1.53  (1.04 to 2.14) | 6.20  (2.01 to 14.60) | 2.24  (0.94 to 4.55) |
|  | Southeast Asia | 209.96  (177.96 to 252.57) | 1.55  (1.03 to 2.24) | 1.54  (1.00 to 2.32) | 0.31  (0.21 to 0.45) | 8.75  (2.89 to 20.28) | 4.73  (2.00 to 9.56) |
|  | High-income Asia Pacific | 2.73  (2.20 to 3.46) | 0.17  (0.09 to 0.33) | 5.37  (2.78 to 9.32) | 1.39  (0.76 to 2.38) | 6.32  (2.04 to 14.78) | 3.42  (1.41 to 7.01) |
|  | Central Europe | 20.24  (18.87 to 22.36) | 0.14  (0.10 to 0.21) | 3.18  (1.86 to 5.13) | 0.73  (0.43 to 1.21) | 7.29  (2.40 to 16.99) | 1.96  (0.82 to 3.96) |
|  | Eastern Europe | 766.36  (733.75 to 816.00) | 0.61  (0.51 to 0.75) | 4.37  (3.05 to 6.26) | 1.27  (0.93 to 1.78) | 5.84  (1.90 to 13.78) | 4.56  (1.89 to 9.21) |
|  | Western Europe | 28.30  (24.87 to 32.38) | 0.10  (0.07 to 0.16) | 2.30  (1.37 to 3.67) | 0.49  (0.30 to 0.78) | 3.01  (0.97 to 7.04) | 3.12  (1.30 to 6.25) |
|  | Southern Latin America | 151.46  (144.76 to 161.17) | 0.64  (0.40 to 1.10) | 4.51  (2.69 to 7.41) | 2.01  (1.25 to 3.23) | 5.94  (1.95 to 13.80) | 7.93  (3.31 to 16.33) |
|  | High-income North America | 61.42  (50.53 to 76.12) | 0.29  (0.19 to 0.47) | 2.05  (1.25 to 3.17) | 1.00  (0.62 to 1.62) | 12.71  (4.13 to 30.12) | 4.76  (1.99 to 9.66) |
|  | Andean Latin America | 183.27  (167.85 to 208.44) | 0.93  (0.60 to 1.53) | 5.23  (2.97 to 8.66) | 1.41  (0.84 to 2.31) | 9.85  (3.21 to 22.95) | 10.23  (4.21 to 21.23) |
|  | Central Latin America | 174.25  (168.38 to 182.79) | 1.28  (1.03 to 1.69) | 4.58  (3.27 to 6.52) | 1.17  (0.88 to 1.63) | 21.29  (7.23 to 48.46) | 8.01  (3.39 to 16.19) |
|  | Tropical Latin America | 278.14  (263.19 to 299.86) | 1.97  (1.49 to 2.78) | 6.31  (4.28 to 9.18) | 1.56  (1.13 to 2.26) | 15.24  (5.02 to 35.65) | 8.64  (3.65 to 17.48) |
|  | Central Sub-Saharan Africa | 3380.06  (2241.74 to 4873.37) | 7.24  (4.08 to 11.76) | 7.06  (4.23 to 11.59) | 2.55  (1.56 to 4.12) | 13.73  (4.56 to 31.88) | 15.25  (6.39 to 30.55) |
|  | Eastern Sub-Saharan Africa | 5491.83  (3956.97 to 7634.99) | 8.41  (5.70 to 12.95) | 11.00  (6.54 to 19.81) | 3.72  (2.23 to 6.71) | 28.48  (9.80 to 64.56) | 9.80  (4.16 to 19.77) |
|  | Southern Sub-Saharan Africa | 15578.14  (13430.42 to 18465.43) | 5.76  (3.89 to 8.50) | 7.24  (4.78 to 10.95) | 2.50  (1.63 to 3.78) | 37.49  (13.06 to 84.75) | 12.52  (5.29 to 25.34) |
|  | Western Sub-Saharan Africa | 3056.64  (2135.50 to 4312.41) | 3.44  (2.36 to 4.85) | 8.31  (5.09 to 13.20) | 2.08  (1.28 to 3.29) | 23.15  (7.88 to 52.82) | 9.25  (3.89 to 18.56) |
|  | North Africa and Middle East | 136.84  (80.74 to 279.37) | 0.36  (0.23 to 0.60) | 2.21  (1.31 to 3.56) | 0.59  (0.35 to 1.00) | 7.11  (2.37 to 16.49) | 3.88  (1.64 to 7.88) |
|  | Caribbean | 1139.36  (685.20 to 1762.28) | 4.08  (2.47 to 6.16) | 6.84  (4.41 to 10.35) | 2.19  (1.41 to 3.28) | 13.75  (4.57 to 31.67) | 8.31  (3.51 to 16.81) |
|  | Oceania | 573.77  (318.00 to 978.11) | 4.04  (1.67 to 7.44) | 2.95  (1.59 to 5.04) | 0.96  (0.51 to 1.75) | 30.64  (10.59 to 70.47) | 6.00  (2.50 to 12.37) |
|  | Australasia | 7.90  (6.09 to 11.03) | 0.12  (0.05 to 0.25) | 6.89  (3.17 to 12.30) | 2.33  (1.01 to 4.39) | 5.04  (1.66 to 12.03) | 3.90  (1.58 to 7.88) |

**Abbreviations:** SDI, Socio-Demographic Index; DALYs, Disability-Adjusted Life Years; NA, not available
