# Supplementary material for: Global, regional, and national burden of HIV and other sexually transmitted infections among women of childbearing age from 1990 to 2021
Source: Microbiol Spectr. 2025 Oct 24;13(12):e00488-25. doi: 10.1128/spectrum.00488-25 (PMC12671144; doi:10.1128/spectrum.00488-25)
Supplement: Table S2 — The change trends of age-standardized rates from 1990 to 2021 in the global and regional levels. [file spectrum.00488-25-s0003.docx]

**Table 2:** The change trends of age-standardized rates from 1990 to 2021 in the global and regional levels

|  |  | HIV/AIDS | | Syphilis | | Chlamydial infection | | Gonococcal infection | | Trichomoniasis | | Genital herpes | |
| --- | --- | --- | --- | --- | --- | --- | --- | --- | --- | --- | --- | --- | --- |
|  |  | AAPC (95% CI) | p | AAPC (95% CI) | p | AAPC (95% CI) | p | AAPC (95% CI) | p | AAPC (95% CI) | p | AAPC (95% CI) | p |
| Age-standardized Incidence | Global | -1.88  (-2.17 to -1.59) | <0.001 | 0.26  (-0.10 to 0.63) | 0.151 | 0.10  (-0.11 to 0.30) | 0.356 | -0.46  (-0.49 to -0.42) | <0.001 | 0.27  (0.25 to 0.30) | <0.001 | 0.24  (0.21 to 0.27) | <0.001 |
|  | High SDI | 0.49  (-0.35 to 1.35) | 0.253 | -0.11  (-0.33 to 0.12) | 0.342 | 0.62  (0.42 to 0.82) | <0.001 | -0.51  (-0.57 to -0.44) | <0.001 | -0.02  (-0.17 to 0.12) | 0.751 | -0.29  (-0.31 to -0.27) | <0.001 |
|  | High-middle SDI | 5.27  (4.61 to 5.94) | <0.001 | 0.60  (0.35 to 0.85) | <0.001 | 0.19  (-0.33 to 0.72) | 0.479 | -0.51  (-0.58 to -0.44) | <0.001 | -0.06  (-0.12 to -0.00) | 0.043 | 0.09  (0.02 to 0.17) | 0.013 |
|  | Middle SDI | 1.95  (1.25 to 2.65) | <0.001 | -0.02  (-0.15 to 0.12) | 0.817 | -0.05  (-0.26 to 0.16) | 0.639 | -0.76  (-0.85 to -0.68) | <0.001 | 0.02  (-0.10 to 0.14) | 0.747 | 0.10  (0.08 to 0.12) | <0.001 |
|  | Low-middle SDI | -3.57  (-3.75 to -3.39) | <0.001 | -0.54  (-0.86 to -0.23) | 0.001 | -0.02  (-0.05 to 0.01) | 0.17 | -0.19  (-0.32 to -0.06) | 0.005 | 0.29  (0.12 to 0.45) | 0.001 | 0.24  (0.23 to 0.25) | <0.001 |
|  | Low SDI | -5.08  (-5.28 to -4.88) | <0.001 | -0.96  (-1.19 to -0.72) | <0.001 | -0.09  (-0.18 to 0.00) | 0.06 | -0.20  (-0.23 to -0.17) | <0.001 | 0.09  (0.04 to 0.14) | <0.001 | 0.05  (-0.02 to 0.13) | 0.163 |
|  | East Asia | 3.54  (2.48 to 4.62) | <0.001 | 0.29  (-0.41 to 0.99) | 0.421 | 0.48  (-0.41 to 1.39) | 0.292 | -0.58  (-0.77 to -0.39) | <0.001 | -0.20  (-0.29 to -0.11) | <0.001 | -0.06  (-0.21 to 0.10) | 0.481 |
|  | Central Asia | 6.89  (5.16 to 8.64) | <0.001 | -0.25  (-0.53 to 0.04) | 0.092 | -0.00  (-0.04 to 0.04) | 0.902 | -0.47  (-0.52 to -0.43) | <0.001 | 0.04  (0.03 to 0.06) | <0.001 | 0.02  (0.01 to 0.03) | 0.003 |
|  | South Asia | 3.68  (2.93 to 4.43) | <0.001 | -0.87  (-1.30 to -0.43) | <0.001 | 0.08  (-0.01 to 0.17) | 0.086 | -0.49  (-0.57 to -0.40) | <0.001 | 0.07  (-0.04 to 0.18) | 0.239 | 0.23  (0.21 to 0.25) | <0.001 |
|  | Southeast Asia | -0.07  (-1.65 to 1.53) | 0.93 | 0.05  (-0.34 to 0.45) | 0.789 | -0.09  (-0.14 to -0.05) | <0.001 | -0.12  (-0.14 to -0.11) | <0.001 | -0.11  (-0.28 to 0.05) | 0.185 | -0.10  (-0.11 to -0.08) | <0.001 |
|  | High-income Asia Pacific | 3.31  (2.26 to 4.38) | <0.001 | -0.15  (-0.19 to -0.12) | <0.001 | 0.16  (0.10 to 0.21) | <0.001 | -0.44  (-0.47 to -0.40) | <0.001 | -0.02  (-0.05 to 0.02) | 0.392 | -0.79  (-0.85 to -0.73) | <0.001 |
|  | Central Europe | 3.79  (2.98 to 4.61) | <0.001 | -0.23  (-0.35 to -0.12) | <0.001 | -0.04  (-0.05 to -0.02) | <0.001 | -0.35  (-0.39 to -0.31) | <0.001 | 0.14  (0.09 to 0.18) | <0.001 | 0.02  (-0.01 to 0.05) | 0.18 |
|  | Eastern Europe | 10.86  (9.71 to 12.03) | <0.001 | -0.20  (-0.39 to -0.01) | 0.035 | 0.02  (0.00 to 0.03) | 0.049 | -0.38  (-0.45 to -0.30) | <0.001 | 0.06  (-0.02 to 0.13) | 0.123 | 0.00  (-0.00 to 0.01) | 0.163 |
|  | Western Europe | -0.92  (-1.27 to -0.57) | <0.001 | -0.03  (-0.13 to 0.06) | 0.475 | -0.06  (-0.08 to -0.04) | <0.001 | -0.34  (-0.48 to -0.20) | <0.001 | 0.05  (-0.08 to 0.18) | 0.471 | -0.13  (-0.17 to -0.09) | <0.001 |
|  | Southern Latin America | 0.88  (0.69 to 1.06) | <0.001 | 0.57  (-0.01 to 1.15) | 0.053 | 0.16  (0.15 to 0.18) | <0.001 | -0.08  (-0.13 to -0.04) | 0.001 | 0.09  (-0.01 to 0.18) | 0.067 | -0.05  (-0.08 to -0.01) | 0.012 |
|  | High-income North America | 0.12  (-1.39 to 1.65) | 0.88 | -0.21  (-0.32 to -0.10) | <0.001 | 0.59  (0.38 to 0.81) | <0.001 | -0.12  (-0.55 to 0.31) | 0.577 | -0.11  (-0.35 to 0.12) | 0.344 | -0.32  (-0.37 to -0.28) | <0.001 |
|  | Andean Latin America | 3.22  (2.54 to 3.90) | <0.001 | -0.13  (-0.24 to -0.01) | 0.031 | -0.07  (-0.30 to 0.16) | 0.527 | -0.33  (-0.43 to -0.22) | <0.001 | 0.07  (0.05 to 0.09) | <0.001 | 0.08  (0.04 to 0.11) | <0.001 |
|  | Central Latin America | 0.88  (0.58 to 1.18) | <0.001 | -0.37  (-0.45 to -0.29) | <0.001 | 0.03  (-0.02 to 0.08) | 0.28 | -0.31  (-0.34 to -0.29) | <0.001 | -0.10  (-0.24 to 0.04) | 0.176 | -0.09  (-0.13 to -0.05) | <0.001 |
|  | Tropical Latin America | 1.86  (1.24 to 2.48) | <0.001 | 1.27  (0.91 to 1.64) | <0.001 | 0.01  (-0.17 to 0.20) | 0.891 | -0.13  (-0.28 to 0.03) | 0.112 | -0.04  (-0.09 to 0.01) | 0.114 | -0.01  (-0.04 to 0.03) | 0.651 |
|  | Central Sub-Saharan Africa | -4.10  (-4.24 to -3.97) | <0.001 | -0.75  (-1.09 to -0.41) | <0.001 | -0.05  (-0.06 to -0.03) | <0.001 | -0.17  (-0.26 to -0.09) | <0.001 | 0.06  (0.04 to 0.08) | <0.001 | -0.04  (-0.07 to -0.02) | <0.001 |
|  | Eastern Sub-Saharan Africa | -5.53  (-5.77 to -5.28) | <0.001 | -1.36  (-1.48 to -1.24) | <0.001 | -0.28  (-0.37 to -0.19) | <0.001 | -0.20  (-0.30 to -0.10) | <0.001 | 0.03  (-0.02 to 0.08) | 0.221 | -0.04  (-0.16 to 0.08) | 0.535 |
|  | Southern Sub-Saharan Africa | -2.33  (-2.77 to -1.89) | <0.001 | -2.34  (-2.48 to -2.19) | <0.001 | -0.22  (-0.30 to -0.15) | <0.001 | -0.35  (-0.55 to -0.15) | 0.001 | -0.34  (-0.49 to -0.18) | <0.001 | 0.18  (0.15 to 0.21) | <0.001 |
|  | Western Sub-Saharan Africa | -3.50  (-3.68 to -3.31) | <0.001 | -0.96  (-1.06 to -0.85) | <0.001 | 0.06  (-0.03 to 0.14) | 0.179 | -0.23  (-0.26 to -0.21) | <0.001 | 0.18  (0.03 to 0.32) | 0.015 | -0.03  (-0.07 to 0.01) | 0.149 |
|  | North Africa and Middle East | 4.53  (4.25 to 4.81) | <0.001 | -0.20  (-0.39 to -0.00) | 0.049 | -0.42  (-0.51 to -0.33) | <0.001 | -0.62  (-0.67 to -0.57) | <0.001 | -0.00  (-0.30 to 0.29) | 0.98 | -0.10  (-0.12 to -0.08) | <0.001 |
|  | Caribbean | -2.88  (-3.09 to -2.66) | <0.001 | 0.74  (0.21 to 1.28) | 0.006 | 0.01  (-0.00 to 0.01) | 0.064 | -0.13  (-0.18 to -0.09) | <0.001 | 0.02  (-0.04 to 0.09) | 0.488 | -0.12  (-0.15 to -0.09) | <0.001 |
|  | Oceania | 11.60  (10.90 to 12.30) | <0.001 | -0.33  (-0.58 to -0.07) | 0.013 | -0.51  (-0.59 to -0.43) | <0.001 | 0.34  (0.32 to 0.36) | <0.001 | -0.08  (-0.43 to 0.26) | 0.636 | 0.11  (0.07 to 0.15) | <0.001 |
|  | Australasia | 3.66  (2.65 to 4.68) | <0.001 | -0.19  (-0.23 to -0.14) | <0.001 | 0.00  (-0.12 to 0.13) | 0.981 | -0.33  (-0.41 to -0.24) | <0.001 | 0.13  (0.07 to 0.18) | <0.001 | -0.71  (-0.78 to -0.64) | <0.001 |
| Age-standardized Prevalence | Global | 3.50  (3.35 to 3.65) | <0.001 | 0.09  (-0.03 to 0.21) | 0.136 | 0.10  (-0.09 to 0.30) | 0.307 | -0.46  (-0.49 to -0.43) | <0.001 | 0.24  (0.19 to 0.28) | <0.001 | 0.18  (0.13 to 0.22) | <0.001 |
|  | High SDI | 1.52  (1.41 to 1.63) | <0.001 | -0.12  (-0.31 to 0.08) | 0.232 | 0.55  (0.38 to 0.73) | <0.001 | -0.58  (-0.64 to -0.52) | <0.001 | -0.07  (-0.11 to -0.03) | 0.001 | -0.45  (-0.49 to -0.41) | <0.001 |
|  | High-middle SDI | 7.09  (6.94 to 7.24) | <0.001 | 0.55  (0.50 to 0.60) | <0.001 | 0.19  (-0.32 to 0.69) | 0.468 | -0.51  (-0.57 to -0.44) | <0.001 | -0.11  (-0.18 to -0.05) | 0.001 | 0.04  (-0.02 to 0.10) | 0.22 |
|  | Middle SDI | 10.07  (9.40 to 10.74) | <0.001 | -0.08  (-0.19 to 0.03) | 0.18 | -0.03  (-0.23 to 0.18) | 0.792 | -0.73  (-0.83 to -0.64) | <0.001 | -0.03  (-0.10 to 0.05) | 0.47 | 0.02  (-0.05 to 0.09) | 0.538 |
|  | Low-middle SDI | 2.31  (2.08 to 2.54) | <0.001 | -0.67  (-0.77 to -0.57) | <0.001 | -0.03  (-0.07 to 0.01) | 0.096 | -0.22  (-0.34 to -0.09) | 0.001 | 0.17  (0.00 to 0.34) | 0.05 | 0.28  (0.26 to 0.30) | <0.001 |
|  | Low SDI | 0.28  (0.08 to 0.48) | 0.007 | -1.33  (-1.60 to -1.06) | <0.001 | -0.15  (-0.22 to -0.08) | <0.001 | -0.30  (-0.34 to -0.26) | <0.001 | -0.22  (-0.27 to -0.17) | <0.001 | 0.03  (-0.05 to 0.12) | 0.436 |
|  | East Asia | 5.92  (5.81 to 6.04) | <0.001 | 0.28  (-0.43 to 1.00) | 0.437 | 0.48  (-0.40 to 1.37) | 0.289 | -0.58  (-0.76 to -0.40) | <0.001 | -0.16  (-0.28 to -0.03) | 0.012 | -0.02  (-0.14 to 0.10) | 0.801 |
|  | Central Asia | 8.35  (8.14 to 8.56) | <0.001 | 0.63  (0.07 to 1.18) | 0.027 | -0.00  (-0.04 to 0.04) | 0.943 | -0.49  (-0.54 to -0.44) | <0.001 | -0.12  (-0.17 to -0.07) | <0.001 | 0.02  (0.01 to 0.03) | <0.001 |
|  | South Asia | 11.74  (10.91 to 12.57) | <0.001 | -0.97  (-1.40 to -0.55) | <0.001 | 0.02  (-0.05 to 0.10) | 0.551 | -0.52  (-0.63 to -0.40) | <0.001 | -0.11  (-0.21 to -0.00) | 0.047 | 0.30  (0.25 to 0.35) | <0.001 |
|  | Southeast Asia | 8.66  (8.45 to 8.86) | <0.001 | 0.06  (-0.36 to 0.49) | 0.778 | -0.09  (-0.14 to -0.05) | <0.001 | -0.13  (-0.14 to -0.12) | <0.001 | -0.22  (-0.26 to -0.17) | <0.001 | -0.17  (-0.18 to -0.15) | <0.001 |
|  | High-income Asia Pacific | 6.20  (5.99 to 6.42) | <0.001 | -0.25  (-0.28 to -0.21) | <0.001 | 0.21  (0.12 to 0.29) | <0.001 | -0.53  (-0.55 to -0.50) | <0.001 | -0.16  (-0.20 to -0.11) | <0.001 | -0.99  (-1.06 to -0.93) | <0.001 |
|  | Central Europe | 7.45  (7.20 to 7.70) | <0.001 | -0.25  (-0.39 to -0.12) | <0.001 | -0.02  (-0.03 to -0.01) | 0.001 | -0.35  (-0.39 to -0.31) | <0.001 | -0.13  (-0.27 to 0.02) | 0.099 | -0.03  (-0.06 to 0.00) | 0.058 |
|  | Eastern Europe | 12.84  (12.57 to 13.12) | <0.001 | -0.24  (-0.31 to -0.18) | <0.001 | 0.01  (-0.01 to 0.03) | 0.392 | -0.39  (-0.47 to -0.31) | <0.001 | 0.06  (-0.08 to 0.20) | 0.406 | 0.01  (0.00 to 0.02) | 0.013 |
|  | Western Europe | 1.77  (1.70 to 1.84) | <0.001 | 0.00  (-0.03 to 0.04) | 0.876 | 0.25  (0.21 to 0.29) | <0.001 | -0.13  (-0.23 to -0.02) | 0.017 | -0.14  (-0.18 to -0.10) | <0.001 | -0.25  (-0.28 to -0.23) | <0.001 |
|  | Southern Latin America | 3.20  (3.14 to 3.25) | <0.001 | 0.62  (-0.14 to 1.39) | 0.111 | 0.10  (0.08 to 0.13) | <0.001 | -0.23  (-0.30 to -0.16) | <0.001 | -0.16  (-0.17 to -0.15) | <0.001 | -0.19  (-0.20 to -0.17) | <0.001 |
|  | High-income North America | 0.99  (0.88 to 1.10) | <0.001 | -0.21  (-0.34 to -0.09) | 0.001 | 0.37  (0.16 to 0.57) | <0.001 | -0.56  (-0.90 to -0.21) | 0.002 | -0.14  (-0.18 to -0.10) | <0.001 | -0.46  (-0.55 to -0.38) | <0.001 |
|  | Andean Latin America | 6.09  (5.93 to 6.24) | <0.001 | -0.36  (-0.57 to -0.15) | 0.001 | -0.03  (-0.25 to 0.19) | 0.802 | -0.07  (-0.18 to 0.03) | 0.179 | -0.13  (-0.21 to -0.06) | 0.001 | -0.06  (-0.10 to -0.03) | <0.001 |
|  | Central Latin America | 5.74  (5.65 to 5.82) | <0.001 | -0.24  (-0.43 to -0.04) | 0.016 | 0.03  (-0.01 to 0.07) | 0.176 | -0.34  (-0.37 to -0.31) | <0.001 | -0.05  (-0.09 to -0.01) | 0.01 | -0.26  (-0.31 to -0.21) | <0.001 |
|  | Tropical Latin America | 3.83  (3.74 to 3.93) | <0.001 | 1.26  (0.96 to 1.56) | <0.001 | 0.16  (-0.06 to 0.37) | 0.154 | 0.02  (-0.14 to 0.18) | 0.833 | -0.06  (-0.08 to -0.04) | <0.001 | -0.26  (-0.29 to -0.22) | <0.001 |
|  | Central Sub-Saharan Africa | -0.49  (-0.74 to -0.25) | <0.001 | -0.93  (-1.12 to -0.73) | <0.001 | -0.10  (-0.12 to -0.09) | <0.001 | -0.25  (-0.33 to -0.18) | <0.001 | -0.17  (-0.22 to -0.13) | <0.001 | -0.04  (-0.08 to 0.01) | 0.106 |
|  | Eastern Sub-Saharan Africa | 0.53  (0.35 to 0.72) | <0.001 | -1.64  (-1.84 to -1.45) | <0.001 | -0.30  (-0.36 to -0.24) | <0.001 | -0.28  (-0.39 to -0.16) | <0.001 | -0.24  (-0.30 to -0.19) | <0.001 | -0.08  (-0.28 to 0.12) | 0.414 |
|  | Southern Sub-Saharan Africa | 6.22  (5.57 to 6.87) | <0.001 | -2.35  (-2.63 to -2.07) | <0.001 | -0.26  (-0.34 to -0.18) | <0.001 | -0.38  (-0.58 to -0.18) | <0.001 | -0.61  (-1.06 to -0.16) | 0.008 | 0.22  (0.17 to 0.27) | <0.001 |
|  | Western Sub-Saharan Africa | 1.59  (1.38 to 1.79) | <0.001 | -1.18  (-1.28 to -1.07) | <0.001 | -0.14  (-0.24 to -0.05) | 0.004 | -0.40  (-0.44 to -0.36) | <0.001 | -0.07  (-0.23 to 0.08) | 0.334 | -0.04  (-0.08 to -0.01) | 0.016 |
|  | North Africa and Middle East | 7.34  (7.15 to 7.53) | <0.001 | -0.33  (-0.69 to 0.03) | 0.069 | -0.41  (-0.48 to -0.33) | <0.001 | -0.61  (-0.65 to -0.56) | <0.001 | -0.13  (-0.45 to 0.18) | 0.409 | -0.15  (-0.19 to -0.11) | <0.001 |
|  | Caribbean | 1.21  (1.11 to 1.31) | <0.001 | 0.74  (0.58 to 0.89) | <0.001 | 0.00  (-0.01 to 0.01) | 0.74 | -0.16  (-0.20 to -0.12) | <0.001 | -0.09  (-0.19 to 0.02) | 0.102 | -0.12  (-0.13 to -0.11) | <0.001 |
|  | Oceania | 16.90  (16.29 to 17.52) | <0.001 | -0.39  (-0.56 to -0.22) | <0.001 | -0.52  (-0.60 to -0.44) | <0.001 | 0.33  (0.30 to 0.35) | <0.001 | 0.05  (-0.07 to 0.17) | 0.415 | 0.12  (0.01 to 0.23) | 0.032 |
|  | Australasia | 3.70  (3.63 to 3.77) | <0.001 | -0.20  (-0.21 to -0.18) | <0.001 | 0.05  (-0.08 to 0.17) | 0.451 | -0.41  (-0.44 to -0.38) | <0.001 | -0.14  (-0.19 to -0.08) | <0.001 | -0.84  (-1.12 to -0.55) | <0.001 |
| Age-standardized Deaths | Global | 1.49  (0.97 to 2.02) | <0.001 | -1.14  (-1.32 to -0.96) | <0.001 | -1.12  (-1.30 to -0.95) | <0.001 | -1.16  (-1.34 to -0.97) | <0.001 | NA | NA | NA | NA |
|  | High SDI | -3.41  (-4.06 to -2.76) | <0.001 | -1.88  (-2.20 to -1.55) | <0.001 | -1.94  (-2.23 to -1.66) | <0.001 | -1.94  (-2.22 to -1.66) | <0.001 | NA | NA | NA | NA |
|  | High-middle SDI | 4.83  (4.34 to 5.33) | <0.001 | -1.45  (-2.02 to -0.87) | <0.001 | -1.34  (-1.97 to -0.70) | <0.001 | -1.36  (-2.01 to -0.71) | <0.001 | NA | NA | NA | NA |
|  | Middle SDI | 6.12  (5.40 to 6.85) | <0.001 | -1.76  (-2.04 to -1.49) | <0.001 | -1.75  (-2.01 to -1.49) | <0.001 | -1.77  (-2.05 to -1.49) | <0.001 | NA | NA | NA | NA |
|  | Low-middle SDI | 2.02  (1.51 to 2.53) | <0.001 | -1.95  (-2.24 to -1.66) | <0.001 | -1.96  (-2.24 to -1.68) | <0.001 | -1.97  (-2.26 to -1.68) | <0.001 | NA | NA | NA | NA |
|  | Low SDI | -1.94  (-2.67 to -1.20) | <0.001 | -2.32  (-2.57 to -2.07) | <0.001 | -2.31  (-2.55 to -2.06) | <0.001 | -2.32  (-2.58 to -2.05) | <0.001 | NA | NA | NA | NA |
|  | East Asia | 7.81  (6.90 to 8.72) | <0.001 | -4.55  (-5.32 to -3.78) | <0.001 | -4.54  (-5.32 to -3.75) | <0.001 | -4.53  (-5.42 to -3.63) | <0.001 | NA | NA | NA | NA |
|  | Central Asia | 2.43  (1.57 to 3.29) | <0.001 | -3.49  (-4.15 to -2.84) | <0.001 | -3.46  (-4.10 to -2.82) | <0.001 | -3.48  (-4.12 to -2.84) | <0.001 | NA | NA | NA | NA |
|  | South Asia | 18.48  (15.72 to 21.31) | <0.001 | -2.36  (-2.77 to -1.96) | <0.001 | -2.40  (-2.82 to -1.98) | <0.001 | -2.40  (-2.82 to -1.99) | <0.001 | NA | NA | NA | NA |
|  | Southeast Asia | 1.51  (0.70 to 2.33) | <0.001 | -0.49  (-0.66 to -0.31) | <0.001 | -0.50  (-0.67 to -0.33) | <0.001 | -0.48  (-0.65 to -0.30) | <0.001 | NA | NA | NA | NA |
|  | High-income Asia Pacific | 3.08  (2.56 to 3.60) | <0.001 | -0.04  (-0.59 to 0.51) | 0.888 | -0.02  (-0.56 to 0.52) | 0.942 | -0.06  (-0.62 to 0.49) | 0.819 | NA | NA | NA | NA |
|  | Central Europe | 1.62  (0.67 to 2.57) | 0.001 | -2.93  (-3.50 to -2.36) | <0.001 | -2.95  (-3.52 to -2.37) | <0.001 | -2.94  (-3.50 to -2.37) | <0.001 | NA | NA | NA | NA |
|  | Eastern Europe | 7.66  (6.75 to 8.59) | <0.001 | -0.43  (-1.13 to 0.28) | 0.233 | -0.33  (-1.04 to 0.38) | 0.363 | -0.41  (-1.12 to 0.30) | 0.258 | NA | NA | NA | NA |
|  | Western Europe | -4.14  (-5.21 to -3.06) | <0.001 | -2.30  (-2.71 to -1.89) | <0.001 | -2.27  (-2.66 to -1.87) | <0.001 | -2.29  (-2.70 to -1.87) | <0.001 | NA | NA | NA | NA |
|  | Southern Latin America | 3.35  (2.29 to 4.42) | <0.001 | -0.38  (-0.94 to 0.18) | 0.183 | -0.41  (-0.99 to 0.16) | 0.157 | -0.39  (-0.95 to 0.18) | 0.18 | NA | NA | NA | NA |
|  | High-income North America | -4.85  (-6.86 to -2.79) | <0.001 | -1.64  (-2.19 to -1.10) | <0.001 | -1.61  (-2.19 to -1.02) | <0.001 | -1.59  (-2.15 to -1.04) | <0.001 | NA | NA | NA | NA |
|  | Andean Latin America | 4.15  (3.48 to 4.82) | <0.001 | -1.68  (-2.79 to -0.55) | 0.004 | -1.68  (-2.77 to -0.58) | 0.003 | -1.70  (-2.80 to -0.60) | 0.003 | NA | NA | NA | NA |
|  | Central Latin America | 1.41  (0.95 to 1.87) | <0.001 | 0.10  (-0.12 to 0.33) | 0.364 | 0.09  (-0.14 to 0.32) | 0.434 | 0.08  (-0.14 to 0.30) | 0.486 | NA | NA | NA | NA |
|  | Tropical Latin America | 1.30  (0.39 to 2.21) | 0.005 | -0.50  (-0.82 to -0.18) | 0.002 | -0.45  (-0.80 to -0.10) | 0.011 | -0.49  (-0.82 to -0.17) | 0.003 | NA | NA | NA | NA |
|  | Central Sub-Saharan Africa | -1.12  (-1.79 to -0.45) | 0.001 | -1.96  (-2.19 to -1.73) | <0.001 | -1.95  (-2.20 to -1.70) | <0.001 | -1.95  (-2.20 to -1.70) | <0.001 | NA | NA | NA | NA |
|  | Eastern Sub-Saharan Africa | -1.67  (-2.46 to -0.87) | <0.001 | -2.24  (-2.37 to -2.12) | <0.001 | -2.19  (-2.32 to -2.06) | <0.001 | -2.20  (-2.33 to -2.08) | <0.001 | NA | NA | NA | NA |
|  | Southern Sub-Saharan Africa | 3.22  (2.00 to 4.46) | <0.001 | -3.60  (-4.75 to -2.44) | <0.001 | -3.58  (-4.68 to -2.46) | <0.001 | -3.68  (-4.86 to -2.50) | <0.001 | NA | NA | NA | NA |
|  | Western Sub-Saharan Africa | 1.00  (0.01 to 1.99) | 0.048 | -1.45  (-1.55 to -1.35) | <0.001 | -1.43  (-1.53 to -1.33) | <0.001 | -1.43  (-1.53 to -1.33) | <0.001 | NA | NA | NA | NA |
|  | North Africa and Middle East | 6.99  (6.51 to 7.47) | <0.001 | -2.01  (-2.28 to -1.75) | <0.001 | -1.97  (-2.22 to -1.71) | <0.001 | -1.97  (-2.15 to -1.78) | <0.001 | NA | NA | NA | NA |
|  | Caribbean | -1.49  (-2.40 to -0.57) | 0.001 | 1.19  (0.55 to 1.83) | <0.001 | 1.20  (0.55 to 1.85) | <0.001 | 1.18  (0.55 to 1.83) | <0.001 | NA | NA | NA | NA |
|  | Oceania | 9.86  (7.79 to 11.97) | <0.001 | -1.48  (-1.75 to -1.21) | <0.001 | -1.47  (-1.74 to -1.20) | <0.001 | -1.47  (-1.74 to -1.20) | <0.001 | NA | NA | NA | NA |
|  | Australasia | -1.93  (-3.50 to -0.34) | 0.018 | -3.63  (-4.62 to -2.63) | <0.001 | -3.57  (-4.57 to -2.57) | <0.001 | -3.65  (-4.63 to -2.65) | <0.001 | NA | NA | NA | NA |
| Age-standardized DALYs | Global | 1.52  (1.02 to 2.01) | <0.001 | -0.70  (-0.91 to -0.48) | <0.001 | -0.27  (-0.35 to -0.20) | <0.001 | -0.75  (-0.84 to -0.67) | <0.001 | 0.24  (0.19 to 0.28) | <0.001 | 0.18  (0.16 to 0.21) | <0.001 |
|  | High SDI | -2.85  (-3.30 to -2.40) | <0.001 | -0.91  (-1.01 to -0.81) | <0.001 | -0.15  (-0.20 to -0.09) | <0.001 | -1.09  (-1.15 to -1.04) | <0.001 | -0.09  (-0.13 to -0.05) | <0.001 | -0.43  (-0.47 to -0.40) | <0.001 |
|  | High-middle SDI | 4.82  (4.23 to 5.41) | <0.001 | -0.82  (-1.30 to -0.35) | 0.001 | -0.15  (-0.37 to 0.07) | 0.188 | -0.75  (-1.02 to -0.48) | <0.001 | -0.11  (-0.18 to -0.04) | 0.001 | 0.05  (-0.02 to 0.12) | 0.136 |
|  | Middle SDI | 6.30  (5.60 to 7.00) | <0.001 | -0.90  (-1.03 to -0.78) | <0.001 | -0.11  (-0.21 to -0.02) | 0.017 | -0.73  (-0.83 to -0.63) | <0.001 | -0.03  (-0.10 to 0.04) | 0.382 | 0.03  (-0.04 to 0.11) | 0.428 |
|  | Low-middle SDI | 1.85  (1.38 to 2.32) | <0.001 | -1.68  (-1.98 to -1.38) | <0.001 | -0.99  (-1.09 to -0.89) | <0.001 | -1.31  (-1.48 to -1.14) | <0.001 | 0.18  (-0.00 to 0.36) | 0.055 | 0.27  (0.24 to 0.30) | <0.001 |
|  | Low SDI | -1.85  (-2.53 to -1.17) | <0.001 | -2.06  (-2.20 to -1.92) | <0.001 | -1.60  (-1.81 to -1.39) | <0.001 | -1.86  (-2.02 to -1.71) | <0.001 | -0.20  (-0.24 to -0.15) | <0.001 | 0.05  (-0.05 to 0.15) | 0.304 |
|  | East Asia | 7.64  (6.79 to 8.49) | <0.001 | -2.92  (-3.12 to -2.71) | <0.001 | -0.47  (-0.60 to -0.35) | <0.001 | -1.37  (-1.49 to -1.25) | <0.001 | -0.15  (-0.27 to -0.03) | 0.016 | 0.00  (-0.12 to 0.12) | 0.996 |
|  | Central Asia | 2.93  (2.19 to 3.68) | <0.001 | -2.91  (-3.46 to -2.35) | <0.001 | -0.92  (-1.19 to -0.66) | <0.001 | -1.80  (-2.12 to -1.48) | <0.001 | -0.12  (-0.19 to -0.06) | <0.001 | 0.02  (-0.02 to 0.06) | 0.264 |
|  | South Asia | 16.70  (14.59 to 18.84) | <0.001 | -2.13  (-2.56 to -1.70) | <0.001 | -1.31  (-1.46 to -1.17) | <0.001 | -1.72  (-2.07 to -1.38) | <0.001 | -0.10  (-0.21 to 0.01) | 0.075 | 0.31  (0.27 to 0.35) | <0.001 |
|  | Southeast Asia | 1.89  (0.35 to 3.45) | 0.016 | 0.39  (0.30 to 0.47) | <0.001 | -0.15  (-0.23 to -0.07) | <0.001 | -0.41  (-0.52 to -0.30) | <0.001 | -0.21  (-0.25 to -0.16) | <0.001 | -0.15  (-0.19 to -0.12) | <0.001 |
|  | High-income Asia Pacific | 3.64  (3.18 to 4.10) | <0.001 | -0.17  (-0.34 to -0.01) | 0.038 | 0.40  (0.35 to 0.44) | <0.001 | -0.73  (-0.78 to -0.69) | <0.001 | -0.16  (-0.18 to -0.14) | <0.001 | -0.96  (-1.03 to -0.89) | <0.001 |
|  | Central Europe | 1.90  (1.24 to 2.57) | <0.001 | -1.96  (-2.37 to -1.55) | <0.001 | -0.10  (-0.13 to -0.07) | <0.001 | -1.00  (-1.13 to -0.88) | <0.001 | -0.14  (-0.24 to -0.04) | 0.008 | -0.02  (-0.06 to 0.02) | 0.36 |
|  | Eastern Europe | 7.76  (6.71 to 8.83) | <0.001 | -0.51  (-1.63 to 0.63) | 0.378 | -0.29  (-0.78 to 0.21) | 0.26 | -0.79  (-1.42 to -0.17) | 0.013 | 0.06  (-0.08 to 0.20) | 0.39 | 0.01  (-0.01 to 0.02) | 0.304 |
|  | Western Europe | -3.79  (-4.50 to -3.07) | <0.001 | -1.32  (-1.58 to -1.06) | <0.001 | 0.93  (0.77 to 1.09) | <0.001 | -0.09  (-0.34 to 0.16) | 0.485 | -0.14  (-0.18 to -0.10) | <0.001 | -0.25  (-0.29 to -0.21) | <0.001 |
|  | Southern Latin America | 3.26  (2.50 to 4.02) | <0.001 | 0.15  (-0.32 to 0.62) | 0.532 | -0.10  (-0.26 to 0.06) | 0.206 | -0.49  (-0.62 to -0.36) | <0.001 | -0.16  (-0.19 to -0.13) | <0.001 | -0.18  (-0.20 to -0.16) | <0.001 |
|  | High-income North America | -4.17  (-4.79 to -3.54) | <0.001 | -0.81  (-1.05 to -0.57) | <0.001 | -0.98  (-1.09 to -0.87) | <0.001 | -1.50  (-1.63 to -1.37) | <0.001 | -0.16  (-0.20 to -0.11) | <0.001 | -0.46  (-0.54 to -0.38) | <0.001 |
|  | Andean Latin America | 4.13  (3.51 to 4.76) | <0.001 | -2.49  (-2.72 to -2.25) | <0.001 | 0.04  (-0.23 to 0.32) | 0.749 | -0.48  (-0.67 to -0.28) | <0.001 | -0.14  (-0.21 to -0.07) | <0.001 | -0.05  (-0.11 to 0.01) | 0.095 |
|  | Central Latin America | 1.56  (1.12 to 2.01) | <0.001 | -0.22  (-0.49 to 0.05) | 0.103 | 0.06  (-0.08 to 0.20) | 0.408 | -0.41  (-0.60 to -0.23) | <0.001 | -0.06  (-0.10 to -0.02) | 0.005 | -0.25  (-0.30 to -0.19) | <0.001 |
|  | Tropical Latin America | 1.11  (0.64 to 1.58) | <0.001 | 0.13  (-0.06 to 0.32) | 0.189 | 1.85  (1.67 to 2.02) | <0.001 | 1.20  (1.01 to 1.39) | <0.001 | -0.06  (-0.08 to -0.04) | <0.001 | -0.22  (-0.27 to -0.18) | <0.001 |
|  | Central Sub-Saharan Africa | -1.14  (-1.91 to -0.35) | 0.004 | -1.95  (-2.19 to -1.71) | <0.001 | -1.09  (-1.21 to -0.96) | <0.001 | -1.19  (-1.30 to -1.08) | <0.001 | -0.15  (-0.20 to -0.09) | <0.001 | -0.01  (-0.06 to 0.04) | 0.708 |
|  | Eastern Sub-Saharan Africa | -1.64  (-2.40 to -0.87) | <0.001 | -2.28  (-2.42 to -2.13) | <0.001 | -1.78  (-1.83 to -1.72) | <0.001 | -1.90  (-1.96 to -1.85) | <0.001 | -0.23  (-0.30 to -0.15) | <0.001 | -0.09  (-0.24 to 0.06) | 0.22 |
|  | Southern Sub-Saharan Africa | 3.44  (2.19 to 4.72) | <0.001 | -1.82  (-2.35 to -1.29) | <0.001 | -2.23  (-2.65 to -1.80) | <0.001 | -2.37  (-2.86 to -1.88) | <0.001 | -0.63  (-1.04 to -0.23) | 0.002 | 0.20  (0.14 to 0.25) | <0.001 |
|  | Western Sub-Saharan Africa | 0.89  (-0.05 to 1.84) | 0.063 | -1.19  (-1.27 to -1.11) | <0.001 | -1.27  (-1.35 to -1.19) | <0.001 | -1.70  (-1.83 to -1.57) | <0.001 | -0.06  (-0.23 to 0.11) | 0.482 | -0.03  (-0.07 to 0.01) | 0.118 |
|  | North Africa and Middle East | 6.93  (6.47 to 7.40) | <0.001 | -1.72  (-1.93 to -1.52) | <0.001 | -0.22  (-0.29 to -0.15) | <0.001 | -0.34  (-0.46 to -0.22) | <0.001 | -0.13  (-0.47 to 0.20) | 0.428 | -0.15  (-0.19 to -0.11) | <0.001 |
|  | Caribbean | -1.44  (-2.33 to -0.54) | 0.002 | 0.75  (0.32 to 1.18) | 0.001 | 0.55  (0.20 to 0.89) | 0.002 | 0.41  (0.06 to 0.77) | 0.022 | -0.09  (-0.18 to 0.00) | 0.064 | -0.12  (-0.15 to -0.09) | <0.001 |
|  | Oceania | 10.23  (8.20 to 12.31) | <0.001 | 0.06  (-0.26 to 0.38) | 0.707 | -1.43  (-1.57 to -1.29) | <0.001 | -0.98  (-1.16 to -0.81) | <0.001 | 0.06  (-0.09 to 0.20) | 0.461 | 0.14  (0.01 to 0.27) | 0.04 |
|  | Australasia | -1.17  (-2.69 to 0.38) | 0.139 | -1.96  (-2.34 to -1.58) | <0.001 | 0.06  (-0.03 to 0.14) | 0.185 | -0.63  (-0.68 to -0.59) | <0.001 | -0.13  (-0.19 to -0.06) | <0.001 | -0.93  (-0.99 to -0.86) | <0.001 |

**Abbreviations:** AAPC, average annual percent change; CI, confidence interval; NA, not available.
