# Supplementary material for: Global, regional, and national burden of HIV and other sexually transmitted infections among women of childbearing age from 1990 to 2021
Source: Microbiol Spectr. 2025 Oct 24;13(12):e00488-25. doi: 10.1128/spectrum.00488-25 (PMC12671144; doi:10.1128/spectrum.00488-25)
Supplement: Table S3 — The age-standardized incidence, prevalence, mortality, and DALY rates of 204 countries and territories. [file spectrum.00488-25-s0004.docx]

**Table 3**: The age-standardized incidence, prevalence, mortality and DALYs rates of 204 countries and territories.

|  |  | | HIV/AIDS | | | | Syphilis | | |  | |
| --- | --- | --- | --- | --- | --- | --- | --- | --- | --- | --- | --- |
|  | |  | 1990 | 2021 | AAPC (95% CI) | P | 1990 | 2021 | AAPC(95% CI) | P |  |
| Age-standardized Incidence | | Afghanistan | 0.63  (0.25 to 1.56) | 1.50  (0.59 to 2.75) | 2.82  (2.15 to 3.49) | <0.001 | 104.66  (53.95 to 176.50) | 108.25  (55.62 to 182.64) | 0.14  (0.07 to 0.21) | <0.001 | |
|  | | Albania | 0.15  (0.11 to 0.22) | 0.11  (0.06 to 0.21) | -0.94  (-1.35 to -0.53) | <0.001 | 36.39  (18.73 to 61.44) | 36.69  (18.78 to 63.21) | 0.03  (0.02 to 0.03) | <0.001 | |
|  | | Algeria | 0.52  (0.38 to 0.68) | 6.50  (4.85 to 8.38) | 8.52  (8.22 to 8.82) | <0.001 | 113.67  (58.39 to 192.04) | 113.38  (59.13 to 188.83) | 0.11  (-0.7 to 0.93) | 0.789 | |
|  | | American Samoa | 0.67  (0.37 to 1.44) | 18.93  (10.41 to 27.86) | 11.73  (11.2 to 12.26) | <0.001 | 518.23  (266.04 to 859.83) | 512.81  (267.14 to 856.28) | -0.03  (-0.07 to 0) | 0.082 | |
|  | | Andorra | 0.92  (0.66 to 1.22) | 0.97  (0.70 to 1.29) | -0.22  (-0.9 to 0.46) | 0.519 | 44.52  (22.99 to 75.55) | 43.56  (22.25 to 74.07) | -0.05  (-0.1 to 0.01) | 0.095 | |
|  | | Angola | 62.63  (13.33 to 152.65) | 439.82  (197.75 to 841.59) | 6.42  (6.02 to 6.83) | <0.001 | 1321.90  (689.43 to 2166.68) | 1188.50  (621.85 to 1993.08) | -0.3  (-0.44 to -0.16) | <0.001 | |
|  | | Antigua and Barbuda | 19.48  (11.21 to 44.66) | 16.25  (8.95 to 28.91) | -0.6  (-0.87 to -0.32) | <0.001 | 220.69  (114.37 to 369.79) | 229.30  (120.52 to 383.68) | 0.27  (0.09 to 0.45) | 0.003 | |
|  | | Argentina | 16.47  (11.98 to 21.71) | 15.06  (11.10 to 19.64) | -0.28  (-0.58 to 0.01) | 0.06 | 326.60  (176.74 to 545.52) | 349.75  (182.47 to 585.15) | 0.65  (-0.07 to 1.37) | 0.077 | |
|  | | Armenia | 0.31  (0.00 to 1.59) | 7.69  (3.88 to 14.26) | 12.84  (10.44 to 15.29) | <0.001 | 19.75  (10.03 to 33.03) | 18.21  (9.16 to 30.60) | -0.32  (-0.46 to -0.17) | <0.001 | |
|  | | Australia | 1.01  (0.65 to 1.45) | 3.25  (1.41 to 6.01) | 3.98  (2.94 to 5.03) | <0.001 | 95.54  (49.96 to 157.35) | 90.58  (46.86 to 153.79) | -0.17  (-0.22 to -0.12) | <0.001 | |
|  | | Austria | 8.27  (4.59 to 13.14) | 4.63  (1.75 to 9.10) | -1.8  (-2.61 to -0.98) | <0.001 | 43.57  (22.28 to 72.97) | 42.17  (21.88 to 70.54) | -0.08  (-0.12 to -0.04) | <0.001 | |
|  | | Azerbaijan | 0.49  (0.25 to 1.33) | 2.77  (1.30 to 5.44) | 5.94  (5.43 to 6.45) | <0.001 | 47.61  (24.20 to 79.90) | 42.91  (21.92 to 71.18) | -0.36  (-0.43 to -0.29) | <0.001 | |
|  | | Bahamas | 142.21  (105.49 to 185.97) | 125.43  (63.53 to 219.36) | -0.4  (-0.8 to 0) | 0.051 | 371.84  (190.46 to 625.86) | 380.79  (196.47 to 640.26) | 0.04  (-0.07 to 0.15) | 0.519 | |
|  | | Bahrain | 1.03  (0.67 to 1.53) | 0.98  (0.56 to 1.67) | -0.32  (-0.68 to 0.05) | 0.086 | 182.70  (91.81 to 306.39) | 182.79  (92.90 to 307.13) | 0  (0 to 0.01) | 0.613 | |
|  | | Bangladesh | 0.01  (0.01 to 0.01) | 1.18  (0.83 to 1.63) | 16.37  (15.06 to 17.69) | <0.001 | 505.68  (263.22 to 829.13) | 420.85  (220.53 to 699.05) | -0.59  (-0.68 to -0.49) | <0.001 | |
|  | | Barbados | 62.11  (40.86 to 92.91) | 50.67  (30.28 to 86.15) | -0.81  (-2.44 to 0.86) | 0.339 | 92.92  (49.79 to 152.74) | 103.90  (56.09 to 169.52) | 0.35  (0.31 to 0.38) | <0.001 | |
|  | | Belarus | 7.13  (0.00 to 21.81) | 57.21  (31.80 to 113.19) | 7.41  (6.19 to 8.64) | <0.001 | 28.08  (14.46 to 47.19) | 26.65  (13.75 to 44.42) | -0.21  (-0.49 to 0.07) | 0.15 | |
|  | | Belgium | 7.73  (4.34 to 12.10) | 11.72  (5.59 to 18.56) | 1.34  (0.29 to 2.41) | 0.012 | 43.99  (22.45 to 74.15) | 42.57  (21.92 to 70.66) | -0.07  (-0.13 to -0.02) | 0.008 | |
|  | | Belize | 33.19  (23.49 to 46.09) | 52.39  (29.17 to 91.57) | 1.48  (0.94 to 2.03) | <0.001 | 160.08  (83.93 to 267.13) | 160.64  (84.99 to 264.39) | 0  (-0.04 to 0.04) | 0.895 | |
|  | | Benin | 88.98  (44.16 to 152.27) | 57.60  (23.11 to 123.17) | -1.36  (-1.86 to -0.85) | <0.001 | 422.52  (221.18 to 703.01) | 376.61  (198.77 to 621.19) | -0.3  (-0.76 to 0.16) | 0.201 | |
|  | | Bermuda | 20.53  (12.01 to 47.99) | 7.36  (4.17 to 12.80) | -3.35  (-3.6 to -3.1) | <0.001 | 251.52  (129.42 to 419.61) | 248.95  (129.74 to 415.09) | -0.04  (-0.08 to 0) | 0.041 | |
|  | | Bhutan | 2.10  (1.15 to 3.47) | 7.88  (3.21 to 14.31) | 4.34  (3.98 to 4.71) | <0.001 | 959.10  (485.66 to 1590.36) | 857.83  (433.83 to 1454.51) | -0.38  (-0.43 to -0.32) | <0.001 | |
|  | | Bolivia  (Plurinational State of) | 2.82  (0.65 to 7.70) | 9.50  (5.42 to 14.88) | 3.85  (2.92 to 4.8) | <0.001 | 561.75  (292.87 to 942.34) | 529.42  (276.27 to 895.61) | -0.48  (-0.91 to -0.06) | 0.026 | |
|  | | Bosnia and Herzegovina | 0.04  (0.03 to 0.05) | 0.10  (0.05 to 0.17) | 2.62  (2.03 to 3.22) | <0.001 | 35.44  (18.31 to 60.09) | 36.42  (18.21 to 62.91) | 0.09  (0.08 to 0.1) | <0.001 | |
|  | | Botswana | 2855.60  (1945.05 to 3998.48) | 490.46  (245.80 to 916.40) | -5.59  (-5.82 to -5.36) | <0.001 | 2015.73  (1240.10 to 2961.16) | 979.07  (516.89 to 1611.85) | -2.33  (-2.72 to -1.94) | <0.001 | |
|  | | Brazil | 17.83  (13.45 to 23.42) | 29.88  (15.93 to 52.51) | 1.86  (1.23 to 2.48) | <0.001 | 358.08  (187.56 to 593.49) | 538.02  (301.33 to 837.45) | 1.29  (0.94 to 1.65) | <0.001 | |
|  | | Brunei Darussalam | 1.57  (0.83 to 2.59) | 4.86  (2.12 to 9.19) | 3.89  (2.11 to 5.7) | <0.001 | 98.26  (51.24 to 162.51) | 95.66  (50.00 to 157.73) | -0.1  (-0.24 to 0.04) | 0.152 | |
|  | | Bulgaria | 0.67  (0.00 to 1.19) | 3.14  (1.60 to 5.13) | 5.3  (4.52 to 6.08) | <0.001 | 21.04  (10.67 to 35.18) | 20.81  (10.64 to 35.11) | -0.02  (-0.06 to 0.03) | 0.405 | |
|  | | Burkina Faso | 605.50  (343.29 to 971.99) | 16.00  (5.44 to 34.86) | -11.03  (-11.31 to -10.75) | <0.001 | 401.64  (213.21 to 663.54) | 408.95  (215.64 to 671.91) | 0.12  (-0.14 to 0.37) | 0.371 | |
|  | | Burundi | 1405.71  (476.23 to 2644.98) | 11.55  (4.90 to 23.48) | -14.38  (-14.98 to -13.78) | <0.001 | 427.46  (232.07 to 695.59) | 386.26  (210.91 to 627.09) | -0.1  (-0.58 to 0.38) | 0.684 | |
|  | | Cabo Verde | 154.50  (42.30 to 313.07) | 26.41  (5.12 to 121.51) | -5.54  (-6.08 to -4.99) | <0.001 | 331.24  (172.41 to 555.63) | 316.58  (165.22 to 528.66) | -0.13  (-0.42 to 0.16) | 0.385 | |
|  | | Cambodia | 0.46  (0.33 to 0.61) | 11.35  (6.30 to 17.66) | 10.58  (6.64 to 14.67) | <0.001 | 49.82  (25.88 to 83.46) | 47.50  (24.94 to 78.27) | -0.26  (-0.79 to 0.27) | 0.332 | |
|  | | Cameroon | 416.52  (249.97 to 632.13) | 219.34  (114.75 to 386.98) | -1.95  (-2.31 to -1.58) | <0.001 | 1598.61  (872.98 to 2548.21) | 831.02  (426.27 to 1398.20) | -2.1  (-2.29 to -1.9) | <0.001 | |
|  | | Canada | 6.01  (2.38 to 11.01) | 11.97  (4.70 to 22.19) | 2.1  (1.22 to 2.99) | <0.001 | 27.11  (13.85 to 45.36) | 26.14  (13.44 to 43.79) | -0.13  (-0.3 to 0.05) | 0.153 | |
|  | | Central African Republic | 3177.21  (1833.94 to 5149.67) | 269.48  (84.63 to 671.25) | -7.69  (-8.11 to -7.27) | <0.001 | 1412.28  (735.47 to 2344.42) | 1362.17  (718.14 to 2240.23) | 0.06  (-0.62 to 0.74) | 0.863 | |
|  | | Chad | 244.41  (94.01 to 472.12) | 78.49  (29.83 to 164.03) | -3.6  (-4.32 to -2.89) | <0.001 | 892.30  (458.21 to 1499.17) | 758.56  (388.43 to 1280.38) | -0.52  (-0.59 to -0.46) | <0.001 | |
|  | | Chile | 3.19  (1.84 to 4.88) | 15.23  (6.88 to 28.30) | 5.16  (4.84 to 5.49) | <0.001 | 167.06  (88.05 to 278.91) | 164.55  (87.82 to 269.80) | 0.26  (-0.62 to 1.14) | 0.57 | |
|  | | China | 0.58  (0.35 to 1.00) | 1.64  (0.76 to 2.95) | 3.32  (2.27 to 4.38) | <0.001 | 100.09  (51.03 to 169.85) | 113.26  (57.82 to 192.26) | 0.3  (-0.43 to 1.03) | 0.421 | |
|  | | Colombia | 4.52  (1.71 to 18.34) | 14.48  (7.17 to 26.52) | 3.66  (1.65 to 5.72) | <0.001 | 188.72  (96.81 to 316.63) | 177.76  (91.11 to 295.62) | -0.24  (-0.3 to -0.17) | <0.001 | |
|  | | Comoros | 0.27  (0.06 to 0.67) | 0.27  (0.09 to 0.49) | -0.01  (-0.82 to 0.8) | 0.971 | 1177.76  (621.50 to 1939.60) | 759.93  (400.37 to 1257.44) | -1.4  (-1.83 to -0.96) | <0.001 | |
|  | | Congo | 1215.11  (582.14 to 2119.03) | 261.79  (81.72 to 657.77) | -4.82  (-5.03 to -4.62) | <0.001 | 1040.74  (544.08 to 1722.41) | 939.86  (494.22 to 1549.75) | -0.2  (-0.46 to 0.06) | 0.133 | |
|  | | Cook Islands | 0.68  (0.37 to 1.49) | 20.20  (11.13 to 29.81) | 11.81  (11.17 to 12.45) | <0.001 | 535.84  (280.13 to 897.27) | 514.38  (263.56 to 865.38) | -0.15  (-0.18 to -0.11) | <0.001 | |
|  | | Costa Rica | 2.89  (1.87 to 4.35) | 7.42  (3.69 to 12.56) | 3.04  (2.61 to 3.47) | <0.001 | 146.89  (75.83 to 244.26) | 139.98  (72.60 to 233.71) | -0.13  (-0.2 to -0.06) | <0.001 | |
|  | | Coted'Ivoire | 1848.11  (1083.65 to 2834.26) | 108.69  (49.83 to 212.52) | -8.74  (-9.07 to -8.41) | <0.001 | 846.76  (470.03 to 1364.61) | 601.30  (311.35 to 1012.08) | -0.99  (-1.37 to -0.6) | <0.001 | |
|  | | Croatia | 0.73  (0.44 to 1.10) | 0.66  (0.29 to 1.17) | -0.59  (-4.3 to 3.27) | 0.761 | 36.52  (18.61 to 60.96) | 36.76  (18.81 to 63.24) | 0.02  (0.01 to 0.03) | <0.001 | |
|  | | Cuba | 1.63  (1.12 to 2.38) | 11.18  (4.54 to 27.28) | 6.29  (5.2 to 7.4) | <0.001 | 186.97  (97.11 to 309.98) | 190.96  (103.85 to 305.92) | 0.07  (-0.31 to 0.45) | 0.709 | |
|  | | Cyprus | 0.32  (0.15 to 0.53) | 1.82  (1.00 to 3.27) | 5.46  (4.56 to 6.37) | <0.001 | 43.04  (22.01 to 72.37) | 42.47  (21.87 to 71.84) | -0.02  (-0.09 to 0.05) | 0.634 | |
|  | | Czechia | 0.18  (0.11 to 0.29) | 1.08  (0.41 to 1.91) | 5.97  (5.34 to 6.6) | <0.001 | 36.44  (18.69 to 61.45) | 37.84  (19.30 to 63.80) | 0.12  (0.11 to 0.13) | <0.001 | |
|  | | Democratic People's Republic of Korea | 0.16  (0.05 to 0.32) | 5.52  (1.00 to 12.61) | 11.78  (11 to 12.56) | <0.001 | 90.33  (46.10 to 150.89) | 91.76  (46.95 to 153.85) | 0.05  (0.04 to 0.06) | <0.001 | |
|  | | Democratic Republic of the Congo | 474.09  (283.73 to 740.80) | 16.32  (8.28 to 29.68) | -10.39  (-10.79 to -9.99) | <0.001 | 1450.53  (786.93 to 2328.41) | 1222.96  (640.46 to 2017.31) | -0.78  (-1.1 to -0.47) | <0.001 | |
|  | | Denmark | 6.31  (4.61 to 8.32) | 2.72  (1.98 to 3.57) | -2.57  (-3.33 to -1.81) | <0.001 | 25.80  (13.18 to 43.59) | 25.47  (12.89 to 42.68) | -0.2  (-0.68 to 0.28) | 0.415 | |
|  | | Djibouti | 24.31  (2.90 to 83.60) | 147.55  (45.23 to 366.69) | 5.77  (4.66 to 6.89) | <0.001 | 906.18  (472.72 to 1493.12) | 881.79  (458.54 to 1449.96) | -0.05  (-0.17 to 0.06) | 0.365 | |
|  | | Dominica | 15.50  (10.93 to 21.27) | 12.54  (6.46 to 23.45) | -0.65  (-1.06 to -0.23) | 0.002 | 440.00  (223.25 to 738.59) | 441.09  (225.03 to 750.70) | 0  (-0.06 to 0.06) | 0.915 | |
|  | | Dominican Republic | 186.53  (80.05 to 341.16) | 23.17  (5.80 to 62.67) | -6.44  (-6.95 to -5.92) | <0.001 | 372.91  (191.66 to 617.83) | 385.80  (200.64 to 639.23) | 0.25  (0.11 to 0.4) | 0.001 | |
|  | | Ecuador | 2.54  (1.37 to 6.40) | 21.41  (10.38 to 41.39) | 7.12  (6.75 to 7.5) | <0.001 | 419.55  (222.24 to 700.27) | 407.60  (213.80 to 679.40) | -0.08  (-0.12 to -0.05) | <0.001 | |
|  | | Egypt | 0.25  (0.16 to 0.40) | 1.20  (0.54 to 2.30) | 5.06  (4.71 to 5.41) | <0.001 | 91.74  (45.20 to 156.91) | 91.89  (45.96 to 158.66) | 0.01  (-0.02 to 0.04) | 0.441 | |
|  | | El Salvador | 7.19  (4.32 to 11.06) | 11.57  (6.04 to 20.94) | 1.5  (1.27 to 1.72) | <0.001 | 79.21  (41.60 to 130.60) | 74.41  (39.34 to 123.68) | -0.2  (-0.31 to -0.09) | <0.001 | |
|  | | Equatorial Guinea | 171.86  (50.91 to 424.57) | 1246.18  (250.43 to 3327.65) | 6.6  (6.15 to 7.05) | <0.001 | 1663.52  (860.35 to 2769.06) | 1506.26  (792.30 to 2490.52) | 0.04  (-0.41 to 0.49) | 0.866 | |
|  | | Eritrea | 398.32  (112.58 to 759.95) | 22.48  (9.71 to 45.37) | -8.9  (-9.12 to -8.68) | <0.001 | 581.99  (307.62 to 973.78) | 521.46  (282.12 to 847.26) | -0.57  (-1.1 to -0.03) | 0.038 | |
|  | | Estonia | 0.73  (0.35 to 1.44) | 23.04  (13.67 to 40.44) | 11.95  (9.54 to 14.42) | <0.001 | 42.49  (21.96 to 70.32) | 38.33  (19.31 to 65.07) | -0.37  (-0.54 to -0.19) | <0.001 | |
|  | | Eswatini | 153.48  (2.67 to 798.64) | 701.56  (332.93 to 1241.31) | 4.56  (2.33 to 6.84) | <0.001 | 1786.52  (928.62 to 2795.14) | 1018.89  (533.31 to 1674.85) | -1.92  (-2.16 to -1.67) | <0.001 | |
|  | | Ethiopia | 373.36  (152.26 to 660.98) | 47.65  (26.75 to 97.22) | -6.39  (-6.75 to -6.01) | <0.001 | 1042.77  (534.32 to 1762.35) | 546.94  (280.94 to 920.17) | -1.89  (-2.2 to -1.58) | <0.001 | |
|  | | Fiji | 2.46  (1.37 to 5.98) | 10.86  (5.44 to 19.96) | 5.37  (4.46 to 6.3) | <0.001 | 1210.26  (613.57 to 2042.63) | 937.07  (475.16 to 1584.82) | -0.83  (-0.91 to -0.74) | <0.001 | |
|  | | Finland | 2.35  (1.30 to 3.71) | 1.72  (0.69 to 3.28) | -1.21  (-3.04 to 0.66) | 0.202 | 44.16  (22.53 to 74.23) | 42.47  (21.84 to 71.65) | -0.11  (-0.13 to -0.08) | <0.001 | |
|  | | France | 7.02  (4.81 to 10.10) | 11.31  (8.14 to 15.00) | 1.54  (0.85 to 2.23) | <0.001 | 60.55  (30.50 to 100.57) | 58.92  (29.66 to 98.21) | -0.09  (-0.17 to -0.02) | 0.019 | |
|  | | Gabon | 447.62  (209.42 to 775.63) | 274.15  (93.98 to 699.32) | -1.63  (-1.96 to -1.3) | <0.001 | 1424.52  (756.26 to 2324.10) | 1084.12  (570.72 to 1804.49) | -0.95  (-1.22 to -0.69) | <0.001 | |
|  | | Gambia | 69.29  (23.41 to 145.97) | 261.54  (103.37 to 520.40) | 4.36  (4.08 to 4.64) | <0.001 | 834.61  (424.45 to 1423.29) | 742.04  (380.31 to 1249.68) | -0.32  (-0.64 to 0) | 0.052 | |
|  | | Georgia | 1.82  (0.40 to 5.38) | 5.33  (2.70 to 11.56) | 4.07  (1.66 to 6.54) | 0.001 | 89.52  (45.00 to 151.34) | 85.18  (43.05 to 145.09) | -0.28  (-0.49 to -0.06) | 0.011 | |
|  | | Germany | 3.90  (2.35 to 6.04) | 3.24  (1.39 to 6.43) | -0.54  (-1.44 to 0.37) | 0.245 | 37.86  (19.54 to 63.52) | 36.83  (19.07 to 61.47) | -0.09  (-0.24 to 0.07) | 0.274 | |
|  | | Ghana | 326.62  (220.21 to 452.85) | 161.65  (87.02 to 276.03) | -2.2  (-2.47 to -1.94) | <0.001 | 691.54  (358.04 to 1150.27) | 644.86  (333.57 to 1073.70) | 0.12  (-0.41 to 0.64) | 0.661 | |
|  | | Greece | 2.27  (1.28 to 3.44) | 5.05  (2.73 to 7.97) | 2.78  (2.18 to 3.38) | <0.001 | 40.23  (26.38 to 54.92) | 45.46  (23.08 to 76.97) | 0.35  (0.06 to 0.65) | 0.018 | |
|  | | Greenland | 15.14  (9.03 to 22.48) | 16.84  (2.43 to 38.98) | 0.3  (-0.9 to 1.51) | 0.63 | 52.65  (26.47 to 88.54) | 51.83  (26.53 to 86.92) | -0.04  (-0.06 to -0.02) | <0.001 | |
|  | | Grenada | 13.90  (8.30 to 30.74) | 4.26  (2.47 to 7.37) | -3.81  (-4.28 to -3.34) | <0.001 | 465.60  (234.98 to 783.93) | 497.56  (255.85 to 842.33) | 0.33  (0.23 to 0.43) | <0.001 | |
|  | | Guam | 0.80  (0.43 to 1.97) | 6.46  (3.25 to 12.15) | 7.27  (6.74 to 7.81) | <0.001 | 533.66  (279.00 to 885.36) | 515.98  (266.89 to 857.80) | -0.11  (-0.16 to -0.06) | <0.001 | |
|  | | Guatemala | 9.95  (6.87 to 14.46) | 7.35  (3.87 to 13.72) | -0.65  (-1.11 to -0.19) | 0.006 | 135.81  (71.19 to 225.64) | 138.78  (72.81 to 229.66) | 0.33  (-0.09 to 0.75) | 0.122 | |
|  | | Guinea | 138.19  (61.23 to 254.72) | 113.96  (50.66 to 210.69) | -0.59  (-1.01 to -0.17) | 0.006 | 768.16  (402.68 to 1279.18) | 659.04  (343.98 to 1096.06) | -0.35  (-0.75 to 0.05) | 0.085 | |
|  | | Guinea-Bissau | 110.47  (21.78 to 228.61) | 415.33  (31.92 to 1175.87) | 4.32  (4.01 to 4.63) | <0.001 | 606.23  (318.72 to 1002.26) | 498.01  (261.68 to 824.78) | -0.6  (-0.78 to -0.41) | <0.001 | |
|  | | Guyana | 46.97  (31.61 to 69.18) | 64.35  (36.48 to 110.53) | 0.89  (0.54 to 1.24) | <0.001 | 95.21  (50.81 to 155.70) | 104.19  (56.89 to 166.52) | 0.31  (0.26 to 0.36) | <0.001 | |
|  | | Haiti | 677.19  (343.54 to 1133.07) | 160.17  (53.07 to 337.36) | -4.51  (-4.8 to -4.22) | <0.001 | 700.26  (352.65 to 1200.34) | 596.13  (301.71 to 1021.95) | -0.25  (-0.8 to 0.29) | 0.358 | |
|  | | Honduras | 7.69  (5.66 to 10.25) | 2.76  (1.62 to 4.69) | -3.29  (-4.21 to -2.37) | <0.001 | 94.80  (50.72 to 155.60) | 86.75  (46.26 to 142.57) | -0.08  (-0.64 to 0.48) | 0.772 | |
|  | | Hungary | 0.49  (0.34 to 0.67) | 0.80  (0.37 to 1.33) | 1.49  (0.35 to 2.64) | 0.01 | 74.35  (37.42 to 126.81) | 76.94  (41.18 to 124.73) | 0.13  (-0.11 to 0.37) | 0.281 | |
|  | | Iceland | 3.05  (1.76 to 4.63) | 8.53  (3.37 to 16.18) | 3.31  (-2.64 to 9.63) | 0.282 | 44.31  (23.06 to 73.96) | 42.10  (21.60 to 69.98) | -0.14  (-0.18 to -0.1) | <0.001 | |
|  | | India | 3.00  (1.67 to 5.09) | 6.84  (4.42 to 10.11) | 2.68  (1.53 to 3.85) | <0.001 | 373.81  (195.45 to 616.25) | 271.77  (143.69 to 447.67) | -1.07  (-1.6 to -0.53) | <0.001 | |
|  | | Indonesia | 0.59  (0.19 to 0.94) | 4.67  (2.85 to 7.53) | 7.01  (6 to 8.02) | <0.001 | 401.14  (206.55 to 676.31) | 380.89  (196.75 to 637.09) | -0.06  (-0.19 to 0.08) | 0.39 | |
|  | | Iran  (Islamic Republic of) | 0.12  (0.05 to 0.35) | 2.71  (1.56 to 4.29) | 10.84  (9.99 to 11.69) | <0.001 | 76.62  (38.51 to 132.19) | 78.24  (39.87 to 132.79) | 0.06  (0.03 to 0.1) | 0.001 | |
|  | | Iraq | 0.29  (0.13 to 0.71) | 1.34  (0.32 to 4.06) | 5.01  (4.5 to 5.52) | <0.001 | 107.82  (55.27 to 180.22) | 107.17  (54.87 to 181.24) | -0.02  (-0.03 to -0.01) | 0.003 | |
|  | | Ireland | 9.17  (5.19 to 14.64) | 7.98  (2.93 to 15.53) | -0.36  (-2.44 to 1.75) | 0.734 | 43.19  (22.47 to 72.19) | 43.06  (22.04 to 73.04) | 0.02  (-0.03 to 0.07) | 0.419 | |
|  | | Israel | 3.77  (1.91 to 6.48) | 7.09  (3.31 to 11.68) | 2.21  (1.6 to 2.83) | <0.001 | 42.83  (21.97 to 72.18) | 41.83  (21.54 to 70.03) | -0.05  (-0.11 to 0) | 0.066 | |
|  | | Italy | 12.88  (7.81 to 20.78) | 5.69  (3.19 to 9.31) | -2.6  (-3.73 to -1.45) | <0.001 | 46.79  (23.95 to 78.33) | 44.75  (23.07 to 75.67) | -0.12  (-0.18 to -0.06) | <0.001 | |
|  | | Jamaica | 40.74  (20.25 to 96.67) | 46.99  (23.14 to 90.90) | 0.39  (-0.18 to 0.97) | 0.18 | 761.03  (441.76 to 1144.67) | 467.67  (238.31 to 783.84) | -1.59  (-1.81 to -1.37) | <0.001 | |
|  | | Japan | 0.37  (0.18 to 0.65) | 1.23  (0.66 to 1.93) | 4  (3.4 to 4.61) | <0.001 | 115.41  (59.88 to 193.00) | 107.49  (55.89 to 179.03) | -0.23  (-0.27 to -0.19) | <0.001 | |
|  | | Jordan | 0.24  (0.15 to 0.38) | 0.19  (0.10 to 0.31) | -0.83  (-1.24 to -0.42) | <0.001 | 154.83  (79.03 to 262.26) | 152.73  (79.03 to 256.59) | -0.04  (-0.07 to -0.02) | 0.001 | |
|  | | Kazakhstan | 7.86  (4.99 to 11.55) | 49.81  (24.96 to 99.22) | 5.97  (4.69 to 7.28) | <0.001 | 42.53  (21.97 to 71.42) | 42.79  (22.47 to 70.62) | -0.06  (-0.73 to 0.61) | 0.849 | |
|  | | Kenya | 2417.60  (1939.90 to 2924.08) | 142.61  (99.49 to 191.90) | -8.69  (-8.98 to -8.39) | <0.001 | 1390.37  (729.18 to 2303.70) | 646.18  (338.58 to 1063.91) | -2.5  (-2.62 to -2.38) | <0.001 | |
|  | | Kiribati | 2.04  (1.44 to 2.77) | 1.10  (0.39 to 3.06) | -2.08  (-2.98 to -1.17) | <0.001 | 773.42  (398.64 to 1282.95) | 728.43  (377.60 to 1209.83) | -0.27  (-0.6 to 0.07) | 0.12 | |
|  | | Kuwait | 0.73  (0.53 to 0.96) | 0.08  (0.04 to 0.16) | -7.98  (-11.16 to -4.68) | <0.001 | 108.34  (56.27 to 180.08) | 107.38  (55.28 to 180.28) | -0.03  (-0.04 to -0.02) | <0.001 | |
|  | | Kyrgyzstan | 1.54  (1.01 to 2.27) | 57.65  (26.85 to 107.18) | 12.5  (9.83 to 15.22) | <0.001 | 26.10  (13.45 to 43.84) | 24.89  (12.93 to 41.28) | -0.24  (-0.44 to -0.03) | 0.025 | |
|  | | Lao People's Democratic Republic | 0.08  (0.05 to 0.13) | 6.43  (4.17 to 9.41) | 14.61  (13.19 to 16.04) | <0.001 | 106.23  (55.20 to 178.70) | 104.51  (53.48 to 173.88) | -0.05  (-0.08 to -0.02) | <0.001 | |
|  | | Latvia | 9.53  (5.76 to 14.97) | 50.53  (26.48 to 75.51) | 5.51  (2.52 to 8.58) | <0.001 | 42.87  (21.98 to 72.30) | 39.09  (19.91 to 66.20) | -0.33  (-0.48 to -0.18) | <0.001 | |
|  | | Lebanon | 0.72  (0.51 to 0.98) | 3.57  (2.59 to 4.70) | 5.34  (4.99 to 5.69) | <0.001 | 138.66  (69.88 to 235.95) | 139.43  (72.28 to 235.05) | 0.02  (0.01 to 0.03) | <0.001 | |
|  | | Lesotho | 1316.21  (865.25 to 1866.06) | 1324.93  (745.53 to 2131.74) | 0.06  (-0.44 to 0.57) | 0.809 | 1287.12  (670.91 to 2135.12) | 1022.03  (535.85 to 1698.24) | -0.65  (-0.75 to -0.55) | <0.001 | |
|  | | Liberia | 158.27  (43.69 to 364.09) | 79.86  (33.64 to 167.16) | -2.05  (-2.6 to -1.51) | <0.001 | 1429.50  (726.01 to 2418.34) | 1199.58  (600.61 to 2058.27) | -0.41  (-0.57 to -0.25) | <0.001 | |
|  | | Libya | 0.97  (0.07 to 4.65) | 3.15  (0.04 to 20.04) | 4.08  (3.15 to 5.01) | <0.001 | 107.76  (55.48 to 183.09) | 109.26  (56.44 to 184.55) | 0.05  (0.04 to 0.05) | <0.001 | |
|  | | Lithuania | 4.89  (3.33 to 6.83) | 7.84  (4.67 to 13.15) | 1.58  (1.41 to 1.74) | <0.001 | 37.65  (19.58 to 63.30) | 35.54  (18.27 to 58.90) | -0.18  (-0.32 to -0.04) | 0.01 | |
|  | | Luxembourg | 13.59  (7.67 to 21.16) | 9.14  (3.87 to 17.59) | -0.32  (-0.96 to 0.33) | 0.328 | 45.08  (22.77 to 76.97) | 42.54  (22.00 to 71.25) | -0.16  (-0.21 to -0.11) | <0.001 | |
|  | | Madagascar | 1.09  (0.28 to 2.49) | 41.81  (14.75 to 90.02) | 12.56  (11.88 to 13.25) | <0.001 | 1638.29  (877.87 to 2601.52) | 1156.49  (610.06 to 1925.38) | -1.58  (-1.76 to -1.39) | <0.001 | |
|  | | Malawi | 2383.78  (1714.60 to 3160.94) | 328.65  (188.54 to 529.22) | -6.18  (-6.58 to -5.78) | <0.001 | 914.10  (573.51 to 1286.62) | 418.33  (280.09 to 551.48) | -2.54  (-2.85 to -2.23) | <0.001 | |
|  | | Malaysia | 6.82  (3.78 to 11.49) | 13.52  (6.72 to 25.44) | 2.12  (1.62 to 2.63) | <0.001 | 47.70  (24.69 to 79.45) | 44.78  (23.69 to 74.22) | -0.27  (-0.48 to -0.05) | 0.016 | |
|  | | Maldives | 0.63  (0.39 to 0.95) | 0.78  (0.47 to 1.27) | 0.7  (0.44 to 0.95) | <0.001 | 127.42  (66.27 to 210.86) | 94.37  (49.09 to 156.67) | -0.94  (-1 to -0.88) | <0.001 | |
|  | | Mali | 163.89  (48.52 to 351.90) | 54.35  (22.69 to 109.42) | -3.52  (-3.82 to -3.23) | <0.001 | 1022.18  (521.35 to 1727.45) | 846.09  (430.25 to 1425.19) | -0.42  (-0.54 to -0.3) | <0.001 | |
|  | | Malta | 3.08  (1.86 to 4.63) | 6.74  (2.61 to 12.73) | 2.55  (1.22 to 3.9) | <0.001 | 37.28  (19.29 to 62.35) | 37.27  (19.24 to 62.62) | 0.02  (-0.07 to 0.12) | 0.651 | |
|  | | Marshall Islands | 1.51  (0.86 to 3.21) | 39.77  (22.43 to 57.92) | 11.45  (10.9 to 12.01) | <0.001 | 686.47  (356.10 to 1157.96) | 677.45  (343.97 to 1143.98) | -0.21  (-0.63 to 0.2) | 0.308 | |
|  | | Mauritania | 0.15  (0.06 to 0.37) | 0.02  (0.01 to 0.05) | -6.19  (-7.74 to -4.61) | <0.001 | 876.85  (452.35 to 1463.43) | 689.82  (357.80 to 1160.46) | -0.77  (-0.85 to -0.69) | <0.001 | |
|  | | Mauritius | 2.59  (1.88 to 3.78) | 19.44  (10.28 to 34.76) | 7.15  (1.76 to 12.83) | 0.009 | 183.64  (92.92 to 309.79) | 167.54  (86.00 to 283.44) | -0.3  (-0.35 to -0.25) | <0.001 | |
|  | | Mexico | 10.47  (7.38 to 15.33) | 8.57  (5.24 to 13.36) | -0.72  (-1.18 to -0.25) | 0.003 | 148.48  (77.26 to 246.68) | 128.25  (67.78 to 211.50) | -0.59  (-0.77 to -0.41) | <0.001 | |
|  | | Micronesia  (Federated States of) | 1.54  (0.87 to 3.31) | 41.23  (23.26 to 59.92) | 11.47  (10.86 to 12.08) | <0.001 | 716.76  (361.77 to 1211.54) | 695.03  (357.48 to 1154.27) | -0.03  (-0.13 to 0.07) | 0.528 | |
|  | | Monaco | 1.44  (1.02 to 1.98) | 1.80  (1.34 to 2.34) | 0.67  (-0.06 to 1.41) | 0.07 | 42.95  (22.07 to 72.98) | 42.14  (21.52 to 72.02) | -0.04  (-0.08 to 0) | 0.035 | |
|  | | Mongolia | NA | 0.48  (0.10 to 1.32) | NA | NA | 190.48  (87.08 to 348.50) | 228.58  (106.17 to 414.58) | 0.96  (0.04 to 1.9) | 0.042 | |
|  | | Montenegro | 0.44  (0.30 to 0.60) | 1.04  (0.60 to 1.68) | 2.66  (1.92 to 3.4) | <0.001 | 36.59  (18.54 to 62.48) | 37.54  (19.05 to 63.64) | 0.09  (0.07 to 0.1) | <0.001 | |
|  | | Morocco | 3.12  (1.88 to 4.93) | 2.82  (1.80 to 4.02) | -0.48  (-1.13 to 0.17) | 0.145 | 448.01  (211.20 to 814.48) | 426.85  (203.82 to 780.78) | -0.2  (-1.05 to 0.66) | 0.645 | |
|  | | Mozambique | 430.38  (208.85 to 772.19) | 802.74  (289.69 to 1725.59) | 2.4  (1.52 to 3.28) | <0.001 | 2201.33  (1279.91 to 3352.36) | 1257.20  (652.09 to 2067.48) | -2.06  (-2.29 to -1.83) | <0.001 | |
|  | | Myanmar | 22.65  (12.49 to 36.78) | 28.51  (14.77 to 42.88) | 0.52  (-1.24 to 2.32) | 0.564 | 172.16  (87.77 to 292.01) | 167.56  (84.97 to 286.05) | 0.17  (-0.38 to 0.73) | 0.544 | |
|  | | Namibia | 769.62  (511.89 to 1110.54) | 365.70  (225.38 to 547.55) | -2.53  (-3.02 to -2.05) | <0.001 | 1029.12  (533.78 to 1714.22) | 940.88  (491.92 to 1567.70) | -0.1  (-1.07 to 0.89) | 0.844 | |
|  | | Nauru | 0.67  (0.36 to 1.48) | 20.07  (11.06 to 29.67) | 11.9  (11.33 to 12.46) | <0.001 | 793.09  (404.40 to 1354.94) | 775.61  (391.05 to 1321.43) | -0.05  (-0.08 to -0.02) | 0.003 | |
|  | | Nepal | NA | 3.06  (1.94 to 4.53) | NA | NA | 557.04  (292.16 to 919.04) | 480.01  (248.31 to 796.57) | -0.47  (-0.49 to -0.44) | <0.001 | |
|  | | Netherlands | 2.18  (1.25 to 3.37) | 2.97  (1.21 to 5.69) | 1  (-0.11 to 2.13) | 0.078 | 44.28  (22.63 to 74.59) | 42.42  (21.99 to 70.91) | -0.11  (-0.16 to -0.06) | <0.001 | |
|  | | New Zealand | 1.09  (0.63 to 1.70) | 1.50  (0.70 to 2.91) | 1.23  (-0.93 to 3.44) | 0.266 | 96.74  (49.93 to 161.66) | 88.47  (45.24 to 148.83) | -0.28  (-0.3 to -0.26) | <0.001 | |
|  | | Nicaragua | 1.38  (0.87 to 2.17) | 14.66  (6.21 to 30.37) | 8.09  (7.5 to 8.67) | <0.001 | 109.42  (57.14 to 181.89) | 102.02  (52.79 to 169.20) | -0.15  (-1.01 to 0.71) | 0.725 | |
|  | | Niger | 88.99  (31.13 to 177.01) | 8.30  (2.46 to 22.00) | -7.37  (-7.72 to -7.02) | <0.001 | 183.83  (97.89 to 297.66) | 135.78  (74.15 to 220.28) | -1.03  (-1.16 to -0.9) | <0.001 | |
|  | | Nigeria | 213.87  (156.90 to 280.18) | 127.36  (96.64 to 168.89) | -1.66  (-1.94 to -1.38) | <0.001 | 605.20  (308.79 to 1017.09) | 416.11  (212.72 to 699.13) | -1.15  (-1.61 to -0.68) | <0.001 | |
|  | | Niue | 0.66  (0.36 to 1.50) | 19.43  (10.57 to 28.94) | 11.74  (11.25 to 12.22) | <0.001 | 542.13  (278.52 to 912.70) | 522.24  (267.67 to 877.92) | -0.13  (-0.19 to -0.07) | <0.001 | |
|  | | North Macedonia | 0.04  (0.03 to 0.05) | 0.10  (0.05 to 0.18) | 2.99  (2.23 to 3.76) | <0.001 | 35.45  (17.97 to 60.24) | 36.49  (18.53 to 62.15) | 0.1  (0.06 to 0.13) | <0.001 | |
|  | | Northern Mariana Islands | 1.67  (0.90 to 4.21) | 7.01  (3.54 to 13.07) | 5.32  (3.53 to 7.15) | <0.001 | 580.15  (303.05 to 969.54) | 553.36  (284.77 to 920.90) | -0.16  (-0.19 to -0.13) | <0.001 | |
|  | | Norway | 7.04  (4.64 to 10.30) | 9.01  (3.92 to 16.65) | 0.81  (-0.8 to 2.45) | 0.327 | 46.83  (24.19 to 78.65) | 44.32  (22.95 to 74.05) | -0.16  (-0.21 to -0.11) | <0.001 | |
|  | | Oman | 0.92  (0.58 to 1.44) | 3.85  (1.42 to 6.61) | 4.65  (3.59 to 5.72) | <0.001 | 36.21  (18.81 to 59.84) | 36.21  (18.76 to 59.77) | 0.03  (-0.1 to 0.17) | 0.625 | |
|  | | Pakistan | 0.00  (0.00 to 0.02) | 12.97  (0.20 to 67.96) | 30.36  (25.27 to 35.67) | <0.001 | 442.22  (223.15 to 749.98) | 392.58  (198.36 to 662.87) | -0.36  (-0.43 to -0.3) | <0.001 | |
|  | | Palau | 0.67  (0.37 to 1.47) | 17.98  (9.72 to 27.11) | 11.57  (10.9 to 12.24) | <0.001 | 322.04  (163.75 to 543.86) | 309.51  (156.69 to 523.06) | -0.12  (-0.12 to -0.11) | <0.001 | |
|  | | Palestine | 0.07  (0.04 to 0.16) | 1.02  (0.46 to 2.06) | 9.03  (8.27 to 9.79) | <0.001 | 107.10  (54.65 to 182.33) | 107.31  (55.12 to 181.49) | 0.01  (0 to 0.02) | 0.111 | |
|  | | Panama | 28.04  (16.69 to 43.68) | 44.40  (24.59 to 77.19) | 1.67  (0.34 to 3.02) | 0.013 | 247.81  (125.43 to 415.44) | 244.62  (126.80 to 408.21) | -0.12  (-0.24 to 0.01) | 0.072 | |
|  | | Papua New Guinea | 2.18  (0.86 to 4.33) | 68.84  (29.54 to 123.83) | 11.88  (11.01 to 12.76) | <0.001 | 748.61  (393.69 to 1237.25) | 691.79  (357.88 to 1175.12) | -0.24  (-0.6 to 0.13) | 0.202 | |
|  | | Paraguay | 3.05  (0.70 to 10.93) | 13.28  (6.16 to 26.22) | 4.87  (3.29 to 6.47) | <0.001 | 742.53  (379.62 to 1243.20) | 699.74  (357.85 to 1198.19) | 0.85  (-0.12 to 1.84) | 0.086 | |
|  | | Peru | 7.41  (3.49 to 23.90) | 9.05  (4.23 to 17.01) | 0.91  (0 to 1.82) | 0.049 | 337.96  (177.41 to 561.39) | 323.66  (171.47 to 534.07) | -0.12  (-0.4 to 0.16) | 0.418 | |
|  | | Philippines | 0.12  (0.05 to 0.23) | 25.69  (12.29 to 47.02) | 7.8  (5.63 to 10.02) | <0.001 | 269.91  (139.61 to 449.28) | 280.42  (146.97 to 469.73) | 0.26  (0.11 to 0.4) | <0.001 | |
|  | | Poland | 0.61  (0.27 to 1.14) | 1.72  (0.73 to 3.35) | 3.25  (2.6 to 3.9) | <0.001 | 43.13  (22.06 to 72.23) | 39.39  (20.08 to 66.67) | -0.29  (-0.32 to -0.27) | <0.001 | |
|  | | Portugal | 24.12  (17.50 to 31.65) | 4.99  (3.56 to 6.71) | -5.24  (-6.69 to -3.78) | <0.001 | 43.50  (22.41 to 72.55) | 43.43  (22.23 to 73.42) | 0.01  (-0.03 to 0.05) | 0.579 | |
|  | | Puerto Rico | 141.16  (110.42 to 177.83) | 14.42  (8.41 to 24.53) | -7.1  (-7.78 to -6.41) | <0.001 | 251.21  (130.53 to 416.67) | 251.64  (130.44 to 418.53) | 0  (-0.03 to 0.03) | 0.87 | |
|  | | Qatar | 1.71  (1.16 to 2.48) | 0.26  (0.16 to 0.41) | -5.79  (-7.82 to -3.72) | <0.001 | 182.73  (88.43 to 318.52) | 178.44  (85.78 to 312.90) | -0.08  (-0.11 to -0.06) | <0.001 | |
|  | | Republic of Korea | 0.29  (0.00 to 0.70) | 0.90  (0.23 to 1.80) | 3.47  (1.72 to 5.24) | <0.001 | 131.62  (67.51 to 218.43) | 128.95  (66.60 to 216.20) | -0.05  (-0.11 to 0.02) | 0.173 | |
|  | | Republic of Moldova | 5.68  (1.89 to 26.56) | 26.68  (13.18 to 49.86) | 5.42  (2.25 to 8.68) | 0.001 | 86.79  (43.92 to 145.37) | 71.50  (36.50 to 119.95) | -0.67  (-1.03 to -0.32) | <0.001 | |
|  | | Romania | 1.64  (0.99 to 2.51) | 7.15  (3.43 to 13.35) | 4.95  (3.89 to 6.02) | <0.001 | 76.79  (38.96 to 130.83) | 68.28  (34.18 to 116.80) | -0.18  (-0.37 to 0.01) | 0.058 | |
|  | | Russian Federation | 2.60  (1.59 to 3.79) | 90.58  (58.72 to 149.09) | 12.1  (9.86 to 14.39) | <0.001 | 48.21  (24.56 to 81.58) | 45.00  (23.03 to 75.49) | -0.22  (-0.24 to -0.21) | <0.001 | |
|  | | Rwanda | 691.63  (221.83 to 1380.90) | 116.05  (53.81 to 224.56) | -5.17  (-5.66 to -4.66) | <0.001 | 752.69  (400.98 to 1246.28) | 626.65  (339.34 to 1020.16) | -0.29  (-0.84 to 0.26) | 0.297 | |
|  | | Saint Kitts and Nevis | 26.06  (14.45 to 61.34) | 30.44  (20.80 to 42.94) | 0.38  (-0.1 to 0.87) | 0.124 | 579.72  (288.86 to 986.01) | 529.33  (266.85 to 898.09) | -0.25  (-0.43 to -0.08) | 0.005 | |
|  | | Saint Lucia | 95.54  (67.38 to 131.41) | 49.00  (25.39 to 87.15) | -2.12  (-2.74 to -1.51) | <0.001 | 449.49  (228.66 to 747.18) | 464.13  (240.11 to 772.81) | 0.25  (-0.01 to 0.51) | 0.057 | |
|  | | Saint Vincent and the Grenadines | 52.82  (34.87 to 76.81) | 26.76  (14.28 to 47.69) | -2.24  (-2.82 to -1.65) | <0.001 | 246.76  (127.84 to 411.07) | 250.90  (132.02 to 414.81) | 0.06  (0.04 to 0.07) | <0.001 | |
|  | | Samoa | 1.50  (0.85 to 3.22) | 39.97  (22.12 to 58.25) | 11.49  (10.96 to 12.03) | <0.001 | 156.52  (83.14 to 258.18) | 155.77  (82.75 to 253.08) | 0.05  (-0.06 to 0.15) | 0.376 | |
|  | | San Marino | 1.54  (1.10 to 2.09) | 1.77  (1.31 to 2.30) | 0.48  (-0.11 to 1.08) | 0.108 | 42.41  (21.84 to 71.63) | 42.10  (21.52 to 70.42) | -0.01  (-0.08 to 0.06) | 0.756 | |
|  | | Sao Tome and Principe | 0.93  (0.27 to 4.40) | 0.31  (0.12 to 0.65) | -3.97  (-5.24 to -2.68) | <0.001 | 374.09  (196.31 to 618.31) | 348.60  (183.27 to 574.47) | -0.27  (-0.65 to 0.1) | 0.155 | |
|  | | Saudi Arabia | 2.38  (1.14 to 5.66) | 4.64  (1.04 to 15.91) | 2.14  (1.71 to 2.56) | <0.001 | 51.43  (26.65 to 85.79) | 52.94  (27.93 to 88.28) | 0.04  (-0.14 to 0.22) | 0.661 | |
|  | | Senegal | 77.44  (40.92 to 130.31) | 30.35  (12.29 to 60.21) | -2.85  (-3.34 to -2.36) | <0.001 | 316.30  (169.64 to 520.31) | 271.27  (143.44 to 441.47) | -1.33  (-1.99 to -0.66) | <0.001 | |
|  | | Serbia | 0.40  (0.00 to 0.72) | 0.21  (0.10 to 0.45) | -1.81  (-4.4 to 0.85) | 0.181 | 36.62  (18.56 to 63.08) | 36.90  (18.73 to 63.05) | 0.02  (0.01 to 0.04) | 0.002 | |
|  | | Seychelles | 12.74  (7.85 to 19.66) | 6.08  (3.83 to 9.50) | -2.62  (-3.06 to -2.17) | <0.001 | 110.40  (57.91 to 182.13) | 92.92  (47.82 to 155.53) | -0.54  (-0.6 to -0.48) | <0.001 | |
|  | | Sierra Leone | 88.24  (22.07 to 203.39) | 84.29  (32.78 to 181.59) | -0.28  (-0.68 to 0.11) | 0.161 | 423.67  (223.26 to 705.06) | 387.69  (204.88 to 640.65) | -0.21  (-0.49 to 0.07) | 0.142 | |
|  | | Singapore | 1.67  (0.83 to 2.90) | 1.40  (0.54 to 2.62) | -0.62  (-2.13 to 0.9) | 0.421 | 112.75  (57.94 to 188.54) | 107.54  (56.31 to 177.51) | -0.16  (-0.3 to -0.03) | 0.016 | |
|  | | Slovakia | 0.09  (0.07 to 0.12) | 0.17  (0.08 to 0.32) | 2.09  (1.26 to 2.93) | <0.001 | 23.08  (11.74 to 38.72) | 24.59  (12.67 to 41.70) | 0.21  (0.12 to 0.3) | <0.001 | |
|  | | Slovenia | 0.25  (0.00 to 0.45) | 0.28  (0.15 to 0.57) | -1.14  (-3.64 to 1.43) | 0.381 | 36.85  (18.83 to 62.31) | 36.90  (18.92 to 62.54) | 0  (-0.01 to 0.02) | 0.502 | |
|  | | Solomon Islands | 1.57  (0.90 to 3.31) | 40.97  (22.86 to 58.96) | 11.38  (10.77 to 12) | <0.001 | 880.51  (452.12 to 1478.00) | 847.57  (439.43 to 1444.58) | -0.13  (-0.22 to -0.04) | 0.004 | |
|  | | Somalia | 30.02  (4.87 to 96.95) | 30.80  (9.24 to 73.43) | -0.18  (-0.74 to 0.38) | 0.53 | 1090.86  (576.11 to 1793.60) | 865.38  (471.87 to 1411.27) | -0.64  (-1.2 to -0.08) | 0.025 | |
|  | | South Africa | 312.16  (218.61 to 427.03) | 645.86  (461.76 to 862.53) | 2.24  (1.35 to 3.13) | <0.001 | 2455.44  (1398.07 to 3790.32) | 1113.43  (578.63 to 1861.56) | -2.48  (-2.65 to -2.32) | <0.001 | |
|  | | South Sudan | 121.92  (15.83 to 395.46) | 199.87  (34.32 to 573.77) | 1.68  (1.14 to 2.21) | <0.001 | 1406.18  (724.65 to 2346.18) | 1314.02  (678.83 to 2193.51) | -0.19  (-0.48 to 0.09) | 0.189 | |
|  | | Spain | 26.45  (18.05 to 37.01) | 5.34  (3.15 to 8.00) | -5.04  (-5.27 to -4.81) | <0.001 | 46.41  (23.98 to 78.19) | 44.64  (22.94 to 75.20) | -0.12  (-0.2 to -0.04) | 0.004 | |
|  | | Sri Lanka | 0.83  (0.07 to 4.28) | 1.60  (0.66 to 3.34) | 1.9  (0.82 to 2.99) | 0.001 | 45.38  (23.83 to 75.13) | 41.84  (22.15 to 68.68) | -0.37  (-0.63 to -0.11) | 0.005 | |
|  | | Sudan | 21.88  (2.03 to 79.98) | 72.02  (6.34 to 280.19) | 3.79  (3.46 to 4.12) | <0.001 | 347.15  (166.09 to 610.43) | 333.99  (161.27 to 587.68) | -0.19  (-0.41 to 0.03) | 0.083 | |
|  | | Suriname | 37.44  (22.56 to 63.78) | 99.21  (51.03 to 176.94) | 3.12  (2.6 to 3.64) | <0.001 | 87.98  (46.18 to 142.60) | 91.87  (49.19 to 147.77) | 0.14  (0.06 to 0.22) | 0.001 | |
|  | | Sweden | 10.60  (6.31 to 17.22) | 8.90  (3.75 to 14.70) | -0.7  (-2.92 to 1.56) | 0.539 | 45.65  (23.47 to 77.18) | 43.32  (22.06 to 74.36) | -0.15  (-0.19 to -0.11) | <0.001 | |
|  | | Switzerland | 32.17  (16.52 to 53.33) | 5.88  (2.19 to 11.34) | -5.14  (-7.63 to -2.57) | <0.001 | 32.48  (16.86 to 54.21) | 31.23  (16.07 to 53.36) | -0.1  (-0.2 to 0) | 0.056 | |
|  | | Syrian Arab Republic | 0.15  (0.10 to 0.20) | 0.26  (0.15 to 0.45) | 1.62  (0.95 to 2.31) | <0.001 | 123.97  (64.16 to 207.95) | 115.27  (58.65 to 193.30) | -0.23  (-0.24 to -0.22) | <0.001 | |
|  | | Taiwan  (Province of China) | 0.54  (0.31 to 0.98) | 2.26  (0.58 to 4.76) | 4.56  (3.2 to 5.93) | <0.001 | 88.78  (46.18 to 148.05) | 84.17  (43.78 to 142.63) | -0.17  (-0.22 to -0.12) | <0.001 | |
|  | | Tajikistan | 4.30  (2.64 to 6.82) | 12.01  (5.32 to 21.13) | 3.39  (2.89 to 3.89) | <0.001 | 40.25  (20.52 to 67.39) | 37.19  (19.26 to 61.25) | -0.22  (-0.71 to 0.27) | 0.378 | |
|  | | Thailand | 79.88  (41.07 to 131.59) | 43.24  (21.25 to 77.60) | -2.18  (-4.39 to 0.09) | 0.06 | 101.95  (52.81 to 170.90) | 96.34  (50.26 to 158.56) | -0.32  (-1.1 to 0.47) | 0.423 | |
|  | | Timor-Leste | 31.63  (22.82 to 41.83) | 22.69  (15.75 to 31.68) | -1.17  (-1.99 to -0.34) | 0.006 | 147.53  (74.88 to 251.37) | 143.41  (73.33 to 241.88) | -0.1  (-0.14 to -0.06) | <0.001 | |
|  | | Togo | 409.56  (222.48 to 655.91) | 80.63  (38.64 to 148.99) | -5.12  (-5.89 to -4.33) | <0.001 | 476.16  (252.70 to 785.67) | 419.03  (220.73 to 692.43) | -0.25  (-1.27 to 0.78) | 0.633 | |
|  | | Tokelau | 0.70  (0.37 to 1.56) | 18.66  (10.07 to 27.93) | 11.57  (11.05 to 12.08) | <0.001 | 555.94  (284.68 to 940.00) | 528.20  (276.20 to 884.07) | -0.16  (-0.18 to -0.15) | <0.001 | |
|  | | Tonga | 1.55  (0.88 to 3.32) | 44.49  (25.02 to 64.81) | 11.64  (11.25 to 12.02) | <0.001 | 396.61  (207.30 to 661.22) | 390.51  (202.65 to 649.83) | -0.02  (-0.15 to 0.11) | 0.764 | |
|  | | Trinidad and Tobago | 63.11  (36.33 to 101.55) | 191.79  (118.14 to 327.35) | 3.66  (1.36 to 6.01) | 0.002 | 158.87  (83.99 to 264.85) | 159.96  (84.96 to 261.99) | 0.01  (-0.22 to 0.23) | 0.939 | |
|  | | Tunisia | 0.10  (0.01 to 0.24) | 0.99  (0.46 to 1.72) | 7.68  (6.72 to 8.65) | <0.001 | 107.57  (55.26 to 181.14) | 109.00  (56.26 to 183.61) | 0.04  (0.03 to 0.05) | <0.001 | |
|  | | Turkmenistan | 4.86  (2.59 to 19.10) | 2.45  (1.48 to 4.18) | -1.13  (-1.69 to -0.56) | <0.001 | 53.23  (27.20 to 88.99) | 45.69  (24.20 to 75.80) | -0.5  (-0.53 to -0.47) | <0.001 | |
|  | | Tuvalu | 0.70  (0.37 to 1.58) | 19.43  (10.65 to 28.89) | 11.66  (11.09 to 12.24) | <0.001 | 404.25  (197.97 to 695.36) | 378.35  (187.42 to 642.18) | -0.22  (-0.28 to -0.15) | <0.001 | |
|  | | Türkiye | 0.06  (0.00 to 0.16) | 0.68  (0.34 to1.22) | 6.91  (6.33 to 7.49) | <0.001 | 162.30  (81.55 to 276.94) | 161.91  (81.64 to 277.66) | -0.01  (-0.03 to 0.01) | 0.538 | |
|  | | Uganda | 1138.29  (788.88 to 1531.76) | 284.51  (130.81 to 550.33) | -4.31  (-4.64 to -3.99) | <0.001 | 1197.39  (622.07 to 1970.26) | 1239.11  (648.12 to 2058.29) | 0.26  (-0.05 to 0.57) | 0.101 | |
|  | | Ukraine | 5.64  (3.43 to 9.57) | 108.90  (50.86 to 222.64) | 9.89  (8.69 to 11.11) | <0.001 | 41.95  (21.11 to 70.49) | 40.46  (21.04 to 67.70) | -0.09  (-0.4 to 0.21) | 0.551 | |
|  | | United Arab Emirates | 0.46  (0.25 to 0.91) | 1.86  (1.04 to 2.93) | 4.76  (4.22 to 5.31) | <0.001 | 39.85  (20.49 to 66.00) | 40.14  (20.73 to 66.01) | 0.07  (-0.12 to 0.26) | 0.465 | |
|  | | United Kingdom | 4.11  (2.44 to 6.47) | 11.07  (6.55 to 16.46) | 3.41  (2.48 to 4.35) | <0.001 | 57.14  (29.46 to 95.69) | 55.81  (28.76 to 93.06) | -0.06  (-0.09 to -0.02) | 0.001 | |
|  | | United Republic of Tanzania | 1494.83  (925.79 to 2197.36) | 124.32  (41.66 to 286.84) | -7.5  (-8.15 to -6.84) | <0.001 | 1592.60  (1005.95 to 2300.83) | 1100.86  (578.60 to 1804.31) | -1.44  (-2.09 to -0.79) | <0.001 | |
|  | | United States of America | 20.30  (11.37 to 29.79) | 20.95  (7.90 to 35.07) | 0.03  (-1.53 to 1.61) | 0.972 | 149.34  (77.50 to 250.13) | 140.03  (72.79 to 234.06) | -0.22  (-0.32 to -0.13) | <0.001 | |
|  | | United States Virgin Islands | 18.65  (10.77 to 42.79) | 8.72  (4.98 to 15.36) | -2.45  (-2.84 to -2.05) | <0.001 | 253.91  (132.74 to 425.08) | 260.58  (135.92 to 435.15) | 0.09  (0.06 to 0.13) | <0.001 | |
|  | | Uruguay | 6.74  (2.34 to 21.71) | 31.70  (12.23 to 62.20) | 4.82  (3.7 to 5.95) | <0.001 | 319.85  (165.17 to 534.98) | 316.30  (164.50 to 528.93) | 0.06  (-0.03 to 0.14) | 0.18 | |
|  | | Uzbekistan | 2.77  (0.94 to 5.42) | 39.10  (11.13 to 88.68) | 8.15  (3.75 to 12.74) | <0.001 | 49.40  (25.40 to 81.65) | 43.24  (22.38 to 73.58) | -0.44  (-0.46 to -0.41) | <0.001 | |
|  | | Vanuatu | 1.49  (0.84 to 3.20) | 40.13  (22.67 to 57.91) | 11.54  (11.05 to 12.02) | <0.001 | 752.42  (386.21 to 1266.52) | 748.70  (383.49 to 1268.91) | 0.13  (-0.14 to 0.41) | 0.343 | |
|  | | Venezuela  (Bolivarian Republic of) | 5.35  (3.65 to 8.11) | 17.37  (9.09 to 30.66) | 4.12  (3.44 to 4.81) | <0.001 | 377.88  (188.18 to 651.34) | 369.80  (182.39 to 645.75) | 0.1  (-0.13 to 0.33) | 0.395 | |
|  | | Viet Nam | 3.69  (2.64 to 4.91) | 12.16  (6.79 to 22.18) | 3.9  (3.04 to 4.76) | <0.001 | 91.86  (47.11 to 153.58) | 90.98  (46.70 to 151.55) | -0.04  (-0.05 to -0.02) | <0.001 | |
|  | | Yemen | 0.47  (0.19 to 1.10) | 4.46  (1.80 to 8.34) | 7.59  (7.29 to 7.89) | <0.001 | 83.73  (42.01 to 141.74) | 85.38  (43.83 to 143.92) | 0.06  (0.05 to 0.07) | <0.001 | |
|  | | Zambia | 2657.31  (1794.88 to 3672.79) | 406.68  (210.53 to 710.04) | -5.89  (-6.09 to -5.68) | <0.001 | 1485.09  (888.83 to 2199.32) | 1158.42  (608.59 to 1893.98) | -0.94  (-1.53 to -0.35) | 0.002 | |
|  | | Zimbabwe | 4483.89  (3274.49 to 5781.19) | 266.14  (154.74 to 414.87) | -8.85  (-9.23 to -8.47) | <0.001 | 1194.98  (616.29 to 1945.84) | 755.09  (402.64 to 1226.80) | -1.67  (-2.03 to -1.31) | <0.001 | |
| Age-standardized Prevalence | | Afghanistan | 3.48  (1.51 to 5.84) | 16.15  (8.71 to 24.45) | 5.07  (4.98 to 5.15) | <0.001 | 417.86  (241.64 to 661.67) | 401.62  (232.66 to 643.61) | -0.12  (-0.2 to -0.03) | 0.01 | |
|  | | Albania | 1.11  (0.78 to 1.48) | 1.32  (0.68 to 2.69) | 0.54  (0.39 to 0.7) | <0.001 | 125.35  (70.37 to 200.97) | 127.73  (73.78 to 205.46) | 0.06  (0.04 to 0.08) | <0.001 | |
|  | | Algeria | 2.63  (2.22 to 3.08) | 61.81  (52.36 to 72.20) | 10.72  (10.67 to 10.76) | <0.001 | 412.58  (235.41 to 667.98) | 391.71  (225.87 to 629.11) | -0.04  (-0.96 to 0.89) | 0.934 | |
|  | | American Samoa | 3.29  (2.04 to 4.41) | 123.18  (83.15 to 163.19) | 12.4  (12.3 to 12.51) | <0.001 | 2083.07  (1207.38 to 3286.19) | 2118.52  (1270.66 to 3323.52) | 0.06  (0.03 to 0.08) | <0.001 | |
|  | | Andorra | 5.85  (4.72 to 7.13) | 16.37  (13.02 to 19.80) | 3.36  (3.27 to 3.45) | <0.001 | 256.20  (129.13 to 483.51) | 253.31  (144.97 to 408.45) | -0.02  (-0.06 to 0.02) | 0.412 | |
|  | | Angola | 190.20  (55.48 to 413.34) | 3708.52  (2505.97 to 5244.96) | 9.94  (9.62 to 10.26) | <0.001 | 5435.90  (3322.48 to 8289.75) | 4578.94  (2762.44 to 7164.21) | -0.56  (-0.62 to -0.5) | <0.001 | |
|  | | Antigua and Barbuda | 71.51  (48.02 to 104.53) | 256.62  (164.39 to 408.49) | 4.23  (4.01 to 4.44) | <0.001 | 798.11  (456.11 to 1274.19) | 771.21  (440.10 to 1251.23) | -0.02  (-0.19 to 0.16) | 0.864 | |
|  | | Argentina | 106.56  (87.30 to 128.80) | 214.29  (181.73 to 249.95) | 2.27  (2.21 to 2.33) | <0.001 | 1235.24  (754.63 to 1902.12) | 1324.43  (765.14 to 2098.87) | 0.76  (-0.09 to 1.61) | 0.081 | |
|  | | Armenia | 0.30  (0.00 to 0.54) | 48.97  (32.58 to 76.53) | 17.99  (17.53 to 18.45) | <0.001 | 61.76  (35.25 to 100.87) | 55.69  (31.45 to 90.63) | -0.36  (-0.48 to -0.23) | <0.001 | |
|  | | Australia | 11.74  (6.87 to 17.26) | 35.02  (16.87 to 60.06) | 3.59  (3.52 to 3.66) | <0.001 | 303.02  (172.50 to 489.09) | 287.92  (163.55 to 470.77) | -0.16  (-0.18 to -0.14) | <0.001 | |
|  | | Austria | 17.05  (9.67 to 26.38) | 85.88  (37.40 to 150.82) | 5.34  (5.13 to 5.54) | <0.001 | 139.94  (79.13 to 228.03) | 135.03  (76.97 to 217.91) | -0.1  (-0.16 to -0.05) | <0.001 | |
|  | | Azerbaijan | 3.10  (1.95 to 4.41) | 22.97  (13.16 to 38.67) | 6.67  (6.43 to 6.91) | <0.001 | 164.17  (94.72 to 261.33) | 145.63  (83.29 to 234.50) | -0.39  (-0.44 to -0.33) | <0.001 | |
|  | | Bahamas | 607.17  (405.09 to 857.35) | 1248.13  (680.32 to 2101.37) | 2.32  (2.16 to 2.49) | <0.001 | 1408.71  (810.27 to 2238.34) | 1401.62  (807.83 to 2196.09) | -0.06  (-0.14 to 0.02) | 0.138 | |
|  | | Bahrain | 5.38  (3.69 to 7.52) | 13.05  (9.92 to 18.37) | 2.87  (2.68 to 3.05) | <0.001 | 677.14  (384.13 to 1089.89) | 686.62  (393.14 to 1100.70) | 0.04  (0.03 to 0.05) | <0.001 | |
|  | | Bangladesh | 0.04  (0.03 to 0.05) | 9.60  (7.83 to 11.97) | 19.74  (18.99 to 20.51) | <0.001 | 1776.39  (1066.95 to 2752.54) | 1436.90  (843.92 to 2281.03) | -0.69  (-0.84 to -0.54) | <0.001 | |
|  | | Barbados | 102.96  (63.67 to 162.37) | 559.98  (422.78 to 769.99) | 5.57  (5.39 to 5.74) | <0.001 | 321.44  (191.48 to 508.93) | 336.81  (201.86 to 525.99) | 0.13  (0.05 to 0.21) | 0.002 | |
|  | | Belarus | 9.77  (0.00 to 15.09) | 277.66  (171.44 to 472.60) | 11.36  (11.03 to 11.68) | <0.001 | 92.58  (52.78 to 151.43) | 86.07  (49.66 to 139.47) | -0.29  (-0.46 to -0.12) | 0.001 | |
|  | | Belgium | 25.37  (13.39 to 39.63) | 157.48  (89.56 to 229.92) | 6.07  (5.94 to 6.21) | <0.001 | 139.76  (78.51 to 229.85) | 135.52  (76.84 to 217.17) | -0.08  (-0.13 to -0.03) | 0.001 | |
|  | | Belize | 125.57  (94.61 to 162.93) | 659.26  (359.17 to 1153.32) | 5.48  (5.28 to 5.68) | <0.001 | 562.58  (327.65 to 896.64) | 530.64  (311.77 to 840.21) | -0.24  (-0.45 to -0.02) | 0.031 | |
|  | | Benin | 115.25  (64.62 to 178.96) | 1285.78  (931.70 to 1705.76) | 8.02  (7.2 to 8.85) | <0.001 | 1697.13  (1030.32 to 2615.97) | 1383.26  (822.23 to 2147.74) | -0.65  (-0.87 to -0.43) | <0.001 | |
|  | | Bermuda | 78.38  (52.89 to 113.40) | 128.60  (90.28 to 195.32) | 1.66  (1.38 to 1.93) | <0.001 | 903.45  (519.44 to 1440.62) | 822.80  (467.78 to 1320.16) | -0.3  (-0.33 to -0.27) | <0.001 | |
|  | | Bhutan | 7.51  (4.00 to 12.14) | 107.55  (59.32 to 158.85) | 8.96  (8.77 to 9.15) | <0.001 | 3739.38  (2246.84 to 5829.63) | 3407.35  (1996.52 to 5359.13) | -0.34  (-0.47 to -0.22) | <0.001 | |
|  | | Bolivia  (Plurinational State of) | 5.55  (1.12 to 16.69) | 161.51  (118.46 to 208.50) | 11.13  (10.61 to 11.64) | <0.001 | 2263.69  (1323.51 to 3565.28) | 2000.85  (1143.80 to 3167.93) | -0.6  (-0.89 to -0.31) | <0.001 | |
|  | | Bosnia and Herzegovina | 0.40  (0.30 to 0.50) | 0.76  (0.46 to 1.20) | 1.97  (1.64 to 2.3) | <0.001 | 126.33  (71.24 to 203.07) | 131.15  (75.69 to 210.13) | 0.12  (0.09 to 0.15) | <0.001 | |
|  | | Botswana | 7127.29  (5332.99 to 9510.54) | 19995.51  (16021.05 to 24857.09) | 3.34  (3.05 to 3.63) | <0.001 | 8987.78  (7241.47 to 10900.65) | 3689.10  (2187.66 to 5707.96) | -2.8  (-3.2 to -2.39) | <0.001 | |
|  | | Brazil | 107.63  (72.45 to 154.70) | 345.09  (196.76 to 557.63) | 3.82  (3.73 to 3.92) | <0.001 | 1362.97  (795.98 to 2149.45) | 2045.34  (1392.46 to 2883.43) | 1.25  (0.99 to 1.51) | <0.001 | |
|  | | Brunei Darussalam | 9.84  (4.90 to 17.23) | 72.19  (35.09 to 124.29) | 6.63  (6.44 to 6.82) | <0.001 | 328.68  (192.06 to 520.47) | 332.70  (198.51 to 523.42) | 0.03  (0.01 to 0.06) | 0.006 | |
|  | | Bulgaria | 2.25  (0.00 to 3.86) | 33.10  (21.77 to 47.01) | 9.07  (8.83 to 9.3) | <0.001 | 68.78  (38.80 to 112.32) | 65.80  (36.78 to 107.04) | -0.14  (-0.2 to -0.07) | <0.001 | |
|  | | Burkina Faso | 4900.81  (3510.70 to 6476.69) | 733.44  (484.41 to 986.90) | -5.96  (-6.09 to -5.83) | <0.001 | 1572.32  (967.94 to 2427.17) | 1544.99  (923.94 to 2401.54) | -0.11  (-0.3 to 0.08) | 0.267 | |
|  | | Burundi | 5333.78  (1482.72 to 12956.38) | 1237.57  (921.66 to 1640.15) | -4.71  (-4.97 to -4.44) | <0.001 | 1489.46  (915.42 to 2298.12) | 1262.34  (779.87 to 1947.07) | -0.36  (-0.75 to 0.02) | 0.066 | |
|  | | Cabo Verde | 540.12  (154.64 to 1035.20) | 815.97  (324.84 to 1958.01) | 1.35  (1.11 to 1.59) | <0.001 | 1190.35  (687.16 to 1900.90) | 1103.39  (647.98 to 1748.56) | -0.25  (-0.42 to -0.08) | 0.005 | |
|  | | Cambodia | 0.52  (0.37 to 0.70) | 443.12  (291.74 to 555.19) | 21.85  (19.93 to 23.8) | <0.001 | 175.02  (103.66 to 281.59) | 155.15  (89.48 to 247.54) | -0.57  (-1.12 to -0.01) | 0.047 | |
|  | | Cameroon | 1078.08  (684.08 to 1594.14) | 4921.66  (4010.30 to 5997.47) | 4.97  (4.72 to 5.22) | <0.001 | 8623.48  (6016.40 to 11437.11) | 3826.05  (2264.99 to 5891.74) | -2.62  (-2.74 to -2.51) | <0.001 | |
|  | | Canada | 41.28  (16.70 to 76.24) | 163.33  (76.63 to 273.12) | 4.52  (4.44 to 4.61) | <0.001 | 82.40  (46.67 to 134.48) | 78.60  (45.01 to 126.58) | -0.15  (-0.34 to 0.04) | 0.124 | |
|  | | Central African Republic | 6962.14  (4121.91 to 10868.24) | 4940.76  (3212.62 to 7523.40) | -1.21  (-1.42 to -0.99) | <0.001 | 5952.81  (3631.27 to 9138.36) | 5759.66  (3554.88 to 8737.27) | 0.09  (-0.49 to 0.68) | 0.762 | |
|  | | Chad | 889.47  (424.43 to 1618.16) | 1765.14  (1164.09 to 2429.99) | 2.19  (1.8 to 2.57) | <0.001 | 4188.48  (2550.15 to 6410.31) | 3484.30  (2082.64 to 5331.19) | -0.61  (-0.84 to -0.38) | <0.001 | |
|  | | Chile | 21.02  (13.41 to 30.15) | 160.21  (77.41 to 281.85) | 6.75  (6.67 to 6.83) | <0.001 | 573.23  (332.66 to 926.03) | 538.61  (311.98 to 864.95) | 0.05  (-0.84 to 0.94) | 0.916 | |
|  | | China | 3.34  (1.89 to 4.59) | 18.63  (9.01 to 37.15) | 5.7  (5.59 to 5.82) | <0.001 | 389.45  (226.02 to 618.79) | 433.81  (252.20 to 691.97) | 0.29  (-0.44 to 1.03) | 0.43 | |
|  | | Colombia | 12.62  (6.27 to 31.23) | 212.81  (129.54 to 334.66) | 9.51  (9.2 to 9.82) | <0.001 | 676.92  (389.01 to 1081.88) | 630.68  (362.96 to 1001.61) | -0.34  (-0.42 to -0.25) | <0.001 | |
|  | | Comoros | 1.08  (0.24 to 3.13) | 8.48  (3.99 to 13.08) | 6.85  (6.65 to 7.05) | <0.001 | 5151.83  (3209.29 to 7808.65) | 2789.18  (1669.66 to 4346.32) | -1.95  (-2.5 to -1.4) | <0.001 | |
|  | | Congo | 4886.38  (2927.85 to 7338.47) | 3668.34  (2299.45 to 5927.47) | -0.96  (-1.05 to -0.87) | <0.001 | 3989.30  (2397.91 to 6147.14) | 3395.73  (2035.29 to 5311.94) | -0.52  (-0.65 to -0.39) | <0.001 | |
|  | | Cook Islands | 2.82  (1.81 to 3.92) | 122.30  (84.32 to 160.56) | 12.9  (12.7 to 13.11) | <0.001 | 2134.23  (1227.02 to 3393.42) | 2276.75  (1356.76 to 3563.82) | 0.22  (0.19 to 0.24) | <0.001 | |
|  | | Costa Rica | 17.17  (11.27 to 26.88) | 72.97  (38.30 to 119.13) | 4.75  (4.65 to 4.86) | <0.001 | 506.66  (291.92 to 810.83) | 474.14  (272.23 to 753.89) | -0.19  (-0.24 to -0.13) | <0.001 | |
|  | | Coted'Ivoire | 6979.15  (3879.63 to 11271.78) | 3626.48  (2810.07 to 4521.86) | -2.09  (-2.56 to -1.62) | <0.001 | 3947.24  (2647.26 to 5588.17) | 2575.02  (1520.07 to 3976.32) | -1.35  (-1.42 to -1.28) | <0.001 | |
|  | | Croatia | 1.60  (0.96 to 2.33) | 8.31  (4.60 to 14.23) | 5.41  (5.25 to 5.57) | <0.001 | 121.51  (68.70 to 197.44) | 119.82  (67.03 to 196.11) | -0.04  (-0.05 to -0.04) | <0.001 | |
|  | | Cuba | 9.69  (7.11 to 13.65) | 121.16  (59.41 to 244.13) | 8.54  (8.22 to 8.87) | <0.001 | 641.78  (365.93 to 1029.31) | 622.86  (378.69 to 958.54) | -0.07  (-0.49 to 0.34) | 0.723 | |
|  | | Cyprus | 1.85  (1.08 to 2.84) | 15.88  (8.31 to 28.19) | 7.17  (6.97 to 7.37) | <0.001 | 147.70  (84.35 to 238.59) | 151.26  (88.80 to 237.70) | 0.1  (0.04 to 0.15) | <0.001 | |
|  | | Czechia | 0.86  (0.53 to 1.38) | 12.70  (5.47 to 21.63) | 9.08  (8.92 to 9.25) | <0.001 | 122.17  (67.94 to 198.79) | 122.54  (69.01 to 199.08) | 0.02  (-0.02 to 0.05) | 0.395 | |
|  | | Democratic People's Republic of Korea | 0.69  (0.09 to 1.50) | 67.39  (27.26 to 123.60) | 15.99  (15.69 to 16.3) | <0.001 | 326.71  (186.84 to 522.51) | 331.19  (191.14 to 527.09) | 0.04  (0.04 to 0.05) | <0.001 | |
|  | | Democratic Republic of the Congo | 2599.77  (1822.79 to 3634.46) | 925.39  (685.76 to 1207.22) | -3.25  (-3.62 to -2.87) | <0.001 | 6124.80  (3929.46 to 9031.18) | 4741.90  (2848.44 to 7345.22) | -1.72  (-2.81 to -0.61) | 0.002 | |
|  | | Denmark | 19.11  (14.96 to 23.82) | 19.81  (16.10 to 23.91) | 0.11  (0.04 to 0.19) | 0.003 | 78.32  (43.82 to 128.02) | 76.98  (43.03 to 125.03) | -0.26  (-0.9 to 0.39) | 0.433 | |
|  | | Djibouti | 27.68  (3.36 to 80.57) | 2108.93  (1063.27 to 3937.51) | 14.84  (14.05 to 15.63) | <0.001 | 3655.81  (2203.33 to 5627.13) | 3336.77  (2021.27 to 5169.29) | -0.28  (-0.39 to -0.18) | <0.001 | |
|  | | Dominica | 64.84  (46.13 to 86.42) | 171.68  (104.49 to 298.89) | 3.13  (2.79 to 3.46) | <0.001 | 1737.59  (988.24 to 2786.36) | 1854.55  (1107.50 to 2873.37) | 0.21  (0.17 to 0.25) | <0.001 | |
|  | | Dominican Republic | 339.88  (148.57 to 641.54) | 662.59  (348.06 to 1021.88) | 2.1  (1.89 to 2.3) | <0.001 | 1394.81  (810.18 to 2191.42) | 1422.09  (833.32 to 2251.40) | 0.27  (0.2 to 0.34) | <0.001 | |
|  | | Ecuador | 18.28  (11.87 to 28.25) | 251.73  (157.75 to 402.97) | 8.83  (8.55 to 9.12) | <0.001 | 1573.30  (905.93 to 2475.16) | 1440.27  (829.12 to 2305.12) | -0.29  (-0.31 to -0.26) | <0.001 | |
|  | | Egypt | 1.60  (1.16 to 2.02) | 10.21  (4.64 to 19.56) | 6.16  (6.01 to 6.32) | <0.001 | 335.07  (183.32 to 553.56) | 312.12  (170.74 to 519.07) | -0.23  (-0.24 to -0.21) | <0.001 | |
|  | | El Salvador | 28.52  (18.10 to 44.01) | 215.48  (120.52 to 367.57) | 6.76  (6.58 to 6.93) | <0.001 | 266.75  (156.36 to 422.80) | 240.57  (139.79 to 388.20) | -0.37  (-0.42 to -0.31) | <0.001 | |
|  | | Equatorial Guinea | 488.98  (131.88 to 1188.84) | 13339.01  (6469.46 to 23586.59) | 11.21  (10.69 to 11.74) | <0.001 | 7301.82  (4467.80 to 11078.65) | 5900.75  (3525.24 to 9231.28) | -0.39  (-0.76 to -0.02) | 0.038 | |
|  | | Eritrea | 1206.25  (285.70 to 2618.35) | 629.63  (428.64 to 903.46) | -2.22  (-2.57 to -1.88) | <0.001 | 2198.69  (1330.72 to 3355.67) | 1796.05  (1101.80 to 2768.84) | -0.76  (-0.89 to -0.63) | <0.001 | |
|  | | Estonia | 0.76  (0.36 to 1.42) | 333.13  (210.03 to 561.52) | 21.92  (21.11 to 22.74) | <0.001 | 145.27  (84.22 to 229.60) | 129.59  (72.86 to 209.53) | -0.37  (-0.42 to -0.31) | <0.001 | |
|  | | Eswatini | 212.73  (32.77 to 742.00) | 31504.10  (28023.19 to 35036.76) | 16.76  (15.14 to 18.4) | <0.001 | 8010.79  (4944.96 to 11128.92) | 3975.49  (2363.96 to 6205.08) | -2.47  (-2.61 to -2.33) | <0.001 | |
|  | | Ethiopia | 1070.30  (455.08 to 1880.63) | 1271.87  (955.75 to 1724.67) | 0.48  (0.11 to 0.84) | 0.01 | 4437.42  (2712.74 to 6837.62) | 2126.38  (1265.32 to 3343.70) | -2.26  (-2.53 to -1.98) | <0.001 | |
|  | | Fiji | 13.69  (8.71 to 19.32) | 97.33  (51.62 to 169.14) | 6.52  (6.34 to 6.71) | <0.001 | 5551.70  (3323.60 to 8593.80) | 4244.97  (2528.66 to 6579.98) | -0.84  (-0.89 to -0.79) | <0.001 | |
|  | | Finland | 5.86  (3.58 to 8.52) | 25.43  (13.09 to 41.03) | 4.86  (4.7 to 5.02) | <0.001 | 138.75  (78.27 to 224.89) | 134.90  (76.08 to 218.09) | -0.08  (-0.15 to -0.01) | 0.023 | |
|  | | France | 90.54  (71.06 to 112.43) | 162.05  (130.19 to 194.02) | 1.88  (1.78 to 1.98) | <0.001 | 198.04  (112.25 to 318.70) | 196.88  (110.18 to 319.17) | -0.03  (-0.11 to 0.05) | 0.486 | |
|  | | Gabon | 1404.93  (709.38 to 2191.19) | 5928.22  (3679.07 to 8789.89) | 4.76  (4.31 to 5.22) | <0.001 | 5751.62  (3581.88 to 8614.22) | 3989.33  (2372.56 to 6271.33) | -1.32  (-1.52 to -1.12) | <0.001 | |
|  | | Gambia | 196.43  (58.32 to 394.62) | 2984.16  (1769.77 to 4601.08) | 9.12  (8.2 to 10.06) | <0.001 | 3667.50  (2197.03 to 5739.12) | 3236.94  (1897.64 to 5045.28) | -0.45  (-0.55 to -0.35) | <0.001 | |
|  | | Georgia | 0.87  (0.39 to 1.87) | 50.58  (26.63 to 100.57) | 13.8  (13.26 to 14.33) | <0.001 | 326.69  (184.85 to 526.07) | 327.31  (184.36 to 525.05) | -0.17  (-0.58 to 0.24) | 0.406 | |
|  | | Germany | 38.32  (24.54 to 55.35) | 66.09  (34.52 to 111.93) | 1.79  (1.69 to 1.9) | <0.001 | 119.46  (67.94 to 193.87) | 116.09  (66.29 to 187.28) | -0.07  (-0.34 to 0.2) | 0.608 | |
|  | | Ghana | 1184.58  (815.17 to 1599.83) | 2328.47  (1762.63 to 3021.01) | 2.18  (1.93 to 2.43) | <0.001 | 2984.64  (1791.36 to 4558.99) | 2707.62  (1623.69 to 4157.37) | 0  (-0.63 to 0.64) | 0.994 | |
|  | | Greece | 8.61  (6.05 to 11.59) | 44.02  (27.43 to 62.76) | 5.4  (5.1 to 5.7) | <0.001 | 127.88  (98.12 to 162.77) | 146.88  (83.08 to 239.62) | 0.41  (0.14 to 0.68) | 0.003 | |
|  | | Greenland | 147.02  (99.67 to 217.07) | 254.13  (88.57 to 508.18) | 1.82  (1.63 to 2.01) | <0.001 | 253.02  (135.92 to 440.31) | 273.54  (160.36 to 446.34) | 0.26  (0.22 to 0.3) | <0.001 | |
|  | | Grenada | 54.23  (37.68 to 75.30) | 85.69  (58.53 to 133.73) | 1.43  (1.27 to 1.59) | <0.001 | 1844.38  (1044.13 to 2959.33) | 1978.84  (1139.48 to 3136.30) | 0.43  (0.19 to 0.68) | <0.001 | |
|  | | Guam | 3.55  (2.30 to 5.41) | 49.72  (30.75 to 84.93) | 8.87  (8.54 to 9.2) | <0.001 | 2004.74  (1151.26 to 3217.85) | 1976.85  (1158.15 to 3145.94) | -0.05  (-0.08 to -0.03) | <0.001 | |
|  | | Guatemala | 55.53  (38.35 to 74.27) | 97.38  (54.38 to 169.99) | 1.78  (1.62 to 1.94) | <0.001 | 484.09  (285.98 to 765.27) | 479.39  (279.77 to 754.26) | 0.18  (-0.23 to 0.58) | 0.396 | |
|  | | Guinea | 457.44  (182.21 to 854.77) | 2067.25  (1525.65 to 2709.27) | 4.82  (4.43 to 5.21) | <0.001 | 3547.45  (2193.35 to 5380.95) | 2912.31  (1770.75 to 4423.88) | -0.6  (-0.69 to -0.5) | <0.001 | |
|  | | Guinea-Bissau | 363.52  (67.97 to 765.85) | 4827.37  (871.84 to 9517.04) | 8.73  (8.27 to 9.2) | <0.001 | 2727.39  (1681.29 to 4091.53) | 2054.55  (1242.07 to 3156.56) | -0.88  (-0.94 to -0.82) | <0.001 | |
|  | | Guyana | 201.54  (135.76 to 296.58) | 1004.60  (594.59 to 1624.74) | 5.31  (5.23 to 5.38) | <0.001 | 312.88  (186.38 to 496.48) | 333.46  (204.70 to 513.79) | 0.25  (0.05 to 0.46) | 0.016 | |
|  | | Haiti | 3816.74  (2090.89 to 5872.49) | 2666.16  (1802.13 to 3528.73) | -1.17  (-1.26 to -1.09) | <0.001 | 3404.62  (2075.89 to 5209.27) | 2704.81  (1632.24 to 4153.11) | -0.07  (-1.32 to 1.2) | 0.918 | |
|  | | Honduras | 54.66  (39.84 to 72.68) | 34.90  (20.60 to 57.22) | -1.43  (-1.78 to -1.08) | <0.001 | 315.01  (187.45 to 494.35) | 278.83  (163.98 to 442.62) | -0.29  (-0.79 to 0.22) | 0.266 | |
|  | | Hungary | 7.57  (5.36 to 10.50) | 5.72  (3.82 to 8.17) | -0.86  (-1.09 to -0.63) | <0.001 | 267.69  (150.67 to 435.65) | 287.01  (184.43 to 421.52) | 0.31  (-0.03 to 0.65) | 0.076 | |
|  | | Iceland | 21.80  (13.47 to 32.03) | 90.31  (48.02 to 145.71) | 4.69  (4.41 to 4.98) | <0.001 | 139.35  (78.71 to 224.89) | 132.89  (74.75 to 216.95) | -0.14  (-0.16 to -0.12) | <0.001 | |
|  | | India | 4.86  (3.13 to 7.25) | 148.73  (120.71 to 180.36) | 11.54  (10.81 to 12.28) | <0.001 | 1442.53  (885.37 to 2218.07) | 1020.63  (625.44 to 1568.30) | -1.16  (-1.68 to -0.64) | <0.001 | |
|  | | Indonesia | 1.00  (0.65 to 1.37) | 59.76  (38.51 to 99.50) | 14.11  (13.68 to 14.55) | <0.001 | 1581.64  (924.12 to 2518.97) | 1485.63  (891.89 to 2327.47) | -0.07  (-0.22 to 0.08) | 0.351 | |
|  | | Iran  (Islamic Republic of) | 0.76  (0.46 to 1.27) | 23.53  (15.80 to 33.68) | 11.81  (11.22 to 12.39) | <0.001 | 286.42  (161.76 to 470.37) | 289.05  (165.28 to 471.10) | 0.03  (0.01 to 0.05) | <0.001 | |
|  | | Iraq | 1.37  (0.70 to 2.67) | 11.13  (3.89 to 25.47) | 6.96  (6.7 to 7.22) | <0.001 | 388.38  (222.97 to 621.80) | 378.68  (216.98 to 609.28) | -0.08  (-0.11 to -0.06) | <0.001 | |
|  | | Ireland | 10.90  (6.15 to 17.00) | 80.68  (40.74 to 133.69) | 6.67  (6.46 to 6.88) | <0.001 | 139.23  (78.31 to 226.12) | 136.44  (76.56 to 221.86) | -0.05  (-0.12 to 0.01) | 0.119 | |
|  | | Israel | 15.85  (8.43 to 26.48) | 105.75  (56.84 to 158.26) | 6.31  (6.12 to 6.5) | <0.001 | 140.26  (79.51 to 224.45) | 135.61  (76.57 to 218.57) | -0.1  (-0.15 to -0.04) | 0.001 | |
|  | | Italy | 122.10  (79.77 to 172.08) | 125.27  (79.44 to 182.44) | 0.09  (-0.07 to 0.25) | 0.255 | 156.64  (88.94 to 252.67) | 148.29  (83.81 to 241.29) | -0.15  (-0.21 to -0.09) | <0.001 | |
|  | | Jamaica | 138.49  (79.91 to 236.81) | 573.04  (316.47 to 981.84) | 4.72  (4.3 to 5.14) | <0.001 | 3207.81  (2422.05 to 4081.25) | 1792.77  (1032.47 to 2827.67) | -1.9  (-2.12 to -1.69) | <0.001 | |
|  | | Japan | 2.11  (1.04 to 3.67) | 16.98  (9.79 to 25.10) | 6.92  (6.78 to 7.06) | <0.001 | 402.94  (229.86 to 652.71) | 367.20  (209.73 to 593.78) | -0.3  (-0.34 to -0.26) | <0.001 | |
|  | | Jordan | 1.15  (0.76 to 1.73) | 2.48  (1.65 to 3.87) | 2.49  (2.19 to 2.79) | <0.001 | 577.08  (330.46 to 918.34) | 557.26  (319.70 to 897.00) | -0.12  (-0.14 to -0.09) | <0.001 | |
|  | | Kazakhstan | 6.54  (4.41 to 9.19) | 140.03  (92.05 to 225.92) | 10.37  (9.99 to 10.74) | <0.001 | 146.99  (83.54 to 236.24) | 143.00  (82.10 to 227.87) | -0.28  (-0.72 to 0.16) | 0.212 | |
|  | | Kenya | 6428.58  (5287.88 to 7627.87) | 5310.87  (4391.83 to 6132.83) | -0.63  (-0.83 to -0.44) | <0.001 | 5601.67  (3408.94 to 8559.31) | 2486.46  (1527.16 to 3801.71) | -2.59  (-2.82 to -2.36) | <0.001 | |
|  | | Kiribati | 15.09  (12.45 to 18.11) | 12.49  (5.77 to 23.13) | -0.63  (-0.72 to -0.53) | <0.001 | 3586.46  (2176.76 to 5490.50) | 3412.34  (2123.51 to 5149.73) | -0.29  (-0.35 to -0.24) | <0.001 | |
|  | | Kuwait | 0.87  (0.63 to 1.14) | 1.03  (0.58 to 1.78) | 0.55  (0.35 to 0.74) | <0.001 | 379.91  (218.39 to 612.06) | 363.79  (209.92 to 585.74) | -0.14  (-0.16 to -0.13) | <0.001 | |
|  | | Kyrgyzstan | 6.55  (4.27 to 9.75) | 169.71  (97.57 to 280.55) | 11.05  (10.92 to 11.18) | <0.001 | 90.02  (51.89 to 144.28) | 80.38  (45.30 to 131.16) | -0.42  (-0.8 to -0.04) | 0.032 | |
|  | | Lao People's Democratic Republic | 0.30  (0.22 to 0.38) | 145.11  (116.46 to 175.57) | 22.29  (21.35 to 23.24) | <0.001 | 418.96  (247.76 to 659.92) | 387.31  (227.13 to 616.25) | -0.25  (-0.27 to -0.24) | <0.001 | |
|  | | Latvia | 17.99  (11.84 to 25.49) | 373.03  (280.75 to 498.58) | 10.26  (9.98 to 10.53) | <0.001 | 145.27  (84.22 to 234.82) | 135.16  (78.47 to 215.73) | -0.24  (-0.26 to -0.21) | <0.001 | |
|  | | Lebanon | 4.44  (3.72 to 5.27) | 30.40  (24.55 to 36.75) | 6.39  (6.26 to 6.51) | <0.001 | 505.29  (281.20 to 824.10) | 481.79  (274.08 to 772.49) | -0.16  (-0.17 to -0.15) | <0.001 | |
|  | | Lesotho | 3262.07  (2170.30 to 4622.49) | 32032.26  (27208.12 to 36481.06) | 7.59  (7.18 to 8) | <0.001 | 5413.52  (3256.85 to 8396.83) | 4087.23  (2457.98 to 6329.11) | -0.82  (-1.11 to -0.52) | <0.001 | |
|  | | Liberia | 400.61  (117.90 to 1001.56) | 1775.30  (1266.03 to 2512.89) | 4.84  (4.48 to 5.21) | <0.001 | 7322.13  (4431.37 to 11120.75) | 6274.26  (3733.09 to 9605.15) | -0.28  (-0.47 to -0.09) | 0.004 | |
|  | | Libya | 5.55  (0.45 to 21.20) | 30.95  (0.58 to 181.06) | 5.72  (5.47 to 5.96) | <0.001 | 388.43  (219.59 to 629.85) | 375.38  (215.96 to 605.12) | -0.11  (-0.12 to -0.1) | <0.001 | |
|  | | Lithuania | 29.48  (20.55 to 42.11) | 63.97  (39.97 to 106.61) | 2.49  (2.38 to 2.6) | <0.001 | 124.45  (70.83 to 202.12) | 117.13  (67.09 to 187.18) | -0.15  (-0.29 to 0) | 0.048 | |
|  | | Luxembourg | 33.64  (19.25 to 51.31) | 100.00  (47.90 to 171.99) | 3.57  (3.48 to 3.66) | <0.001 | 143.90  (81.92 to 232.08) | 134.35  (75.17 to 218.72) | -0.2  (-0.26 to -0.14) | <0.001 | |
|  | | Madagascar | 1.58  (0.61 to 3.02) | 392.41  (204.53 to 674.33) | 19.43  (19.01 to 19.86) | <0.001 | 7609.52  (4969.21 to 10672.98) | 4692.71  (2862.67 to 7173.33) | -2.25  (-3.07 to -1.42) | <0.001 | |
|  | | Malawi | 9963.51  (6875.64 to 13562.54) | 11768.11  (9986.03 to 13516.25) | 0.52  (0.22 to 0.83) | 0.001 | 3718.12  (3087.09 to 4465.34) | 1422.61  (1242.60 to 1685.10) | -3.08  (-3.6 to -2.56) | <0.001 | |
|  | | Malaysia | 14.96  (8.71 to 22.78) | 135.95  (76.31 to 224.43) | 7.37  (7.07 to 7.68) | <0.001 | 157.74  (91.42 to 252.94) | 141.98  (81.55 to 228.47) | -0.48  (-0.67 to -0.28) | <0.001 | |
|  | | Maldives | 4.29  (2.59 to 6.33) | 7.17  (4.57 to 12.16) | 1.61  (1.38 to 1.83) | <0.001 | 460.78  (273.67 to 719.05) | 334.73  (198.02 to 525.94) | -1.02  (-1.08 to -0.96) | <0.001 | |
|  | | Mali | 494.37  (193.67 to 1159.72) | 1210.61  (867.32 to 1685.94) | 2.77  (2.37 to 3.17) | <0.001 | 4928.60  (3034.13 to 7485.34) | 3947.60  (2333.96 to 6028.43) | -0.35  (-0.47 to -0.23) | <0.001 | |
|  | | Malta | 13.93  (8.93 to 19.81) | 103.83  (50.50 to 173.51) | 6.7  (6.49 to 6.9) | <0.001 | 120.32  (67.84 to 196.41) | 117.94  (66.23 to 190.39) | -0.05  (-0.1 to -0.01) | 0.018 | |
|  | | Marshall Islands | 6.95  (4.57 to 9.40) | 223.89  (151.81 to 289.80) | 11.83  (11.68 to 11.99) | <0.001 | 2953.39  (1731.52 to 4662.55) | 3022.43  (1822.13 to 4680.33) | 0  (-0.56 to 0.57) | 0.996 | |
|  | | Mauritania | 2.83  (1.02 to 6.69) | 7.21  (1.71 to 9.76) | 3.12  (2.93 to 3.3) | <0.001 | 3999.39  (2457.58 to 6069.26) | 2913.84  (1706.31 to 4519.40) | -1.05  (-1.16 to -0.95) | <0.001 | |
|  | | Mauritius | 6.22  (5.23 to 7.52) | 187.53  (131.29 to 276.35) | 11.65  (11.08 to 12.22) | <0.001 | 664.83  (376.06 to 1068.11) | 629.22  (356.88 to 1002.22) | -0.15  (-0.37 to 0.06) | 0.167 | |
|  | | Mexico | 25.76  (18.52 to 35.07) | 96.34  (61.42 to 143.31) | 4.3  (4.16 to 4.44) | <0.001 | 540.58  (313.87 to 861.07) | 454.26  (267.00 to 723.83) | -0.68  (-0.87 to -0.49) | <0.001 | |
|  | | Micronesia  (Federated States of) | 8.10  (4.71 to 10.96) | 235.02  (160.73 to 305.23) | 11.41  (11.17 to 11.66) | <0.001 | 3107.27  (1833.72 to 4823.34) | 3095.05  (1851.74 to 4796.46) | 0.11  (-0.12 to 0.35) | 0.352 | |
|  | | Monaco | 10.11  (8.27 to 12.26) | 27.34  (22.49 to 32.35) | 3.26  (3.14 to 3.38) | <0.001 | 262.34  (130.83 to 493.63) | 226.24  (132.60 to 367.36) | -0.46  (-0.5 to -0.42) | <0.001 | |
|  | | Mongolia | NA | 5.73  (1.70 to 14.32) | NA | NA | 982.22  (535.90 to 1574.38) | 1553.02  (850.06 to 2478.00) | 3.37  (1.99 to 4.77) | <0.001 | |
|  | | Montenegro | 2.00  (1.33 to 2.81) | 12.39  (8.69 to 17.07) | 6.06  (5.94 to 6.18) | <0.001 | 134.06  (75.47 to 214.69) | 150.93  (89.13 to 236.50) | 0.38  (0.35 to 0.41) | <0.001 | |
|  | | Morocco | 13.15  (8.88 to 20.62) | 70.47  (54.67 to 86.78) | 5.55  (5.34 to 5.77) | <0.001 | 2321.34  (1276.03 to 3708.54) | 2333.17  (1291.21 to 3699.41) | -0.27  (-1.39 to 0.86) | 0.64 | |
|  | | Mozambique | 1309.38  (721.25 to 2252.29) | 16072.93  (13037.79 to 20198.39) | 8.39  (7.79 to 8.99) | <0.001 | 11074.40  (8547.94 to 14030.90) | 5157.57  (3143.25 to 7910.04) | -3.52  (-5.42 to -1.59) | <0.001 | |
|  | | Myanmar | 30.69  (18.61 to 45.61) | 553.52  (322.45 to 709.37) | 9.64  (8.9 to 10.39) | <0.001 | 700.20  (403.05 to 1123.43) | 672.83  (387.82 to 1064.51) | 0.3  (0.22 to 0.37) | <0.001 | |
|  | | Namibia | 1926.41  (1331.67 to 2700.58) | 12821.46  (10971.96 to 14728.21) | 6.15  (5.23 to 7.08) | <0.001 | 4108.15  (2432.84 to 6374.19) | 3581.41  (2090.58 to 5689.97) | -0.23  (-1.34 to 0.88) | 0.682 | |
|  | | Nauru | 3.38  (2.04 to 4.55) | 120.00  (83.87 to 157.46) | 12.15  (11.91 to 12.4) | <0.001 | 3605.14  (2105.37 to 5640.86) | 3909.50  (2400.39 to 5924.32) | 0.36  (0.23 to 0.49) | <0.001 | |
|  | | Nepal | NA | 83.32  (65.92 to 101.95) | NA | NA | 2053.08  (1232.61 to 3168.88) | 1724.91  (1015.32 to 2716.37) | -0.56  (-0.58 to -0.55) | <0.001 | |
|  | | Netherlands | 27.38  (16.94 to 40.45) | 77.28  (42.86 to 122.89) | 3.44  (3.28 to 3.6) | <0.001 | 139.80  (79.54 to 228.41) | 135.08  (76.25 to 217.19) | -0.09  (-0.14 to -0.04) | <0.001 | |
|  | | New Zealand | 7.99  (4.66 to 12.10) | 30.80  (17.52 to 51.01) | 4.44  (4.28 to 4.6) | <0.001 | 339.76  (193.26 to 544.73) | 306.41  (174.62 to 498.19) | -0.33  (-0.35 to -0.31) | <0.001 | |
|  | | Nicaragua | 8.76  (6.33 to 12.20) | 127.36  (64.90 to 220.76) | 9.05  (8.91 to 9.2) | <0.001 | 378.49  (220.10 to 604.68) | 338.73  (195.36 to 545.56) | -0.32  (-1.21 to 0.56) | 0.473 | |
|  | | Niger | 272.26  (93.30 to 543.05) | 326.66  (193.05 to 525.13) | 0.56  (-0.03 to 1.17) | 0.065 | 611.12  (378.06 to 940.10) | 426.65  (257.57 to 666.65) | -1.22  (-1.34 to -1.1) | <0.001 | |
|  | | Nigeria | 643.52  (479.35 to 851.98) | 1962.69  (1640.20 to 2293.16) | 3.58  (3.34 to 3.82) | <0.001 | 2299.70  (1344.83 to 3630.80) | 1513.00  (889.86 to 2417.02) | -1.26  (-1.53 to -0.98) | <0.001 | |
|  | | Niue | 3.38  (2.12 to 4.53) | 118.76  (81.55 to 156.84) | 12.15  (11.88 to 12.43) | <0.001 | 2339.49  (1323.38 to 3791.12) | 2465.26  (1491.31 to 3838.52) | 0.18  (0.07 to 0.29) | 0.001 | |
|  | | North Macedonia | 0.32  (0.22 to 0.43) | 1.00  (0.58 to 1.69) | 3.79  (3.53 to 4.05) | <0.001 | 126.28  (70.70 to 202.39) | 131.56  (76.01 to 208.59) | 0.13  (0.12 to 0.15) | <0.001 | |
|  | | Northern Mariana Islands | 8.02  (5.49 to 11.53) | 65.18  (40.51 to 109.24) | 6.92  (6.66 to 7.19) | <0.001 | 2197.42  (1295.56 to 3460.68) | 2301.36  (1406.51 to 3535.98) | 0.15  (0.13 to 0.18) | <0.001 | |
|  | | Norway | 8.07  (5.15 to 11.44) | 129.96  (70.59 to 209.33) | 9.39  (9.21 to 9.58) | <0.001 | 156.03  (88.41 to 251.21) | 145.91  (82.55 to 236.67) | -0.2  (-0.22 to -0.18) | <0.001 | |
|  | | Oman | 3.88  (2.60 to 5.66) | 45.86  (19.44 to 77.50) | 8.31  (8.15 to 8.46) | <0.001 | 114.38  (65.90 to 185.67) | 111.72  (64.62 to 181.60) | -0.1  (-0.32 to 0.13) | 0.397 | |
|  | | Pakistan | 0.01  (0.00 to 0.06) | 82.85  (2.05 to 417.88) | 32.88  (30.99 to 34.81) | <0.001 | 1526.69  (870.39 to 2482.09) | 1361.44  (794.37 to 2190.77) | -0.36  (-0.43 to -0.3) | <0.001 | |
|  | | Palau | 3.03  (2.01 to 4.11) | 110.99  (75.39 to 148.30) | 12.26  (12.01 to 12.51) | <0.001 | 1168.52  (657.51 to 1907.14) | 1261.37  (742.03 to 1997.57) | 0.3  (0.2 to 0.4) | <0.001 | |
|  | | Palestine | 0.37  (0.23 to 0.58) | 12.93  (7.19 to 21.28) | 12.22  (12.06 to 12.38) | <0.001 | 387.95  (220.13 to 628.13) | 377.05  (216.01 to 611.43) | -0.1  (-0.11 to -0.08) | <0.001 | |
|  | | Panama | 86.83  (54.05 to 133.57) | 451.41  (288.40 to 689.84) | 5.45  (5.21 to 5.69) | <0.001 | 909.42  (516.14 to 1470.00) | 919.60  (536.79 to 1452.91) | -0.06  (-0.23 to 0.1) | 0.44 | |
|  | | Papua New Guinea | 4.12  (1.99 to 6.96) | 936.00  (704.45 to 1203.02) | 18.91  (18.09 to 19.74) | <0.001 | 3366.94  (2061.93 to 5147.68) | 3056.70  (1814.44 to 4761.91) | -0.37  (-0.61 to -0.13) | 0.003 | |
|  | | Paraguay | 14.64  (6.56 to 25.85) | 142.03  (79.44 to 242.37) | 7.52  (7.13 to 7.91) | <0.001 | 2865.90  (1655.09 to 4611.34) | 2730.08  (1577.19 to 4345.59) | 1.05  (-0.04 to 2.15) | 0.059 | |
|  | | Peru | 39.11  (24.15 to 61.60) | 141.39  (82.34 to 224.11) | 4.2  (3.98 to 4.43) | <0.001 | 1230.76  (725.17 to 1939.10) | 1090.98  (634.79 to 1744.79) | -0.38  (-0.72 to -0.05) | 0.026 | |
|  | | Philippines | 0.34  (0.17 to 0.68) | 162.44  (75.72 to 315.76) | 21.63  (21.17 to 22.08) | <0.001 | 978.89  (572.31 to 1557.99) | 1088.10  (657.38 to 1689.93) | 0.51  (0.37 to 0.65) | <0.001 | |
|  | | Poland | 1.51  (0.75 to 2.70) | 25.68  (13.75 to 43.09) | 9.59  (9.28 to 9.89) | <0.001 | 146.11  (83.61 to 236.54) | 131.14  (74.80 to 212.95) | -0.35  (-0.37 to -0.33) | <0.001 | |
|  | | Portugal | 29.21  (22.54 to 36.86) | 123.02  (98.21 to 150.45) | 4.76  (4.43 to 5.09) | <0.001 | 144.64  (81.69 to 234.93) | 140.21  (79.06 to 227.39) | -0.1  (-0.15 to -0.04) | 0.001 | |
|  | | Puerto Rico | 220.14  (161.82 to 284.22) | 78.62  (53.87 to 122.38) | -3.3  (-3.46 to -3.14) | <0.001 | 883.15  (507.54 to 1414.66) | 841.53  (486.00 to 1348.03) | -0.16  (-0.19 to -0.12) | <0.001 | |
|  | | Qatar | 9.40  (5.98 to 13.31) | 4.98  (3.66 to 6.77) | -2.03  (-2.08 to -1.98) | <0.001 | 685.08  (361.92 to 1140.51) | 660.35  (349.25 to 1108.97) | -0.11  (-0.14 to -0.09) | <0.001 | |
|  | | Republic of Korea | 2.36  (0.00 to 3.82) | 6.78  (3.49 to 11.35) | 3.46  (3.23 to 3.68) | <0.001 | 444.28  (256.85 to 714.65) | 422.16  (240.23 to 684.84) | -0.15  (-0.19 to -0.12) | <0.001 | |
|  | | Republic of Moldova | 8.91  (4.48 to 15.98) | 254.92  (148.34 to 416.96) | 11.51  (11.07 to 11.94) | <0.001 | 322.79  (185.36 to 516.87) | 276.19  (160.68 to 436.56) | -0.63  (-0.68 to -0.58) | <0.001 | |
|  | | Romania | 7.46  (4.61 to 10.45) | 115.68  (66.85 to 184.53) | 9.17  (8.73 to 9.61) | <0.001 | 290.08  (162.92 to 470.41) | 254.79  (140.86 to 412.25) | -0.17  (-0.39 to 0.05) | 0.131 | |
|  | | Russian Federation | 16.76  (11.86 to 24.29) | 869.01  (617.23 to 1257.04) | 13.59  (13.22 to 13.97) | <0.001 | 176.26  (101.14 to 283.10) | 161.48  (93.37 to 258.92) | -0.29  (-0.32 to -0.27) | <0.001 | |
|  | | Rwanda | 2377.42  (579.02 to 5206.87) | 3217.61  (2473.11 to 4028.73) | 0.79  (0.42 to 1.17) | <0.001 | 3011.72  (1863.80 to 4538.44) | 2215.40  (1330.39 to 3429.50) | -0.77  (-1.04 to -0.5) | <0.001 | |
|  | | Saint Kitts and Nevis | 71.72  (42.36 to 108.17) | 464.08  (370.71 to 569.10) | 6.22  (5.88 to 6.57) | <0.001 | 2666.54  (1549.99 to 4144.76) | 2259.06  (1297.48 to 3593.66) | -0.49  (-0.67 to -0.32) | <0.001 | |
|  | | Saint Lucia | 62.03  (45.04 to 81.59) | 88.19  (49.21 to 158.33) | 1.1  (0.88 to 1.31) | <0.001 | 1741.91  (1012.82 to 2762.81) | 1815.02  (1073.97 to 2800.89) | 0.24  (0.07 to 0.41) | 0.006 | |
|  | | Saint Vincent and the Grenadines | 188.70  (124.86 to 269.66) | 325.63  (186.80 to 584.14) | 1.7  (1.3 to 2.1) | <0.001 | 918.93  (535.53 to 1447.00) | 875.99  (498.80 to 1392.67) | -0.15  (-0.19 to -0.12) | <0.001 | |
|  | | Samoa | 7.57  (4.69 to 10.13) | 228.96  (155.09 to 297.82) | 11.58  (11.41 to 11.75) | <0.001 | 514.16  (303.76 to 814.54) | 520.48  (320.04 to 808.62) | 0.07  (-0.07 to 0.21) | 0.302 | |
|  | | San Marino | 9.87  (8.01 to 11.99) | 27.28  (22.53 to 32.19) | 3.33  (3.25 to 3.41) | <0.001 | 268.01  (132.61 to 508.30) | 248.08  (142.79 to 402.78) | -0.23  (-0.27 to -0.2) | <0.001 | |
|  | | Sao Tome and Principe | 3.48  (1.72 to 6.54) | 5.28  (3.32 to 8.34) | 1.29  (0.45 to 2.13) | 0.003 | 1431.53  (864.85 to 2232.00) | 1275.80  (770.60 to 1993.84) | -0.62  (-1.19 to -0.04) | 0.035 | |
|  | | Saudi Arabia | 14.14  (8.34 to 23.64) | 47.01  (20.04 to 116.22) | 3.96  (3.83 to 4.08) | <0.001 | 169.80  (96.78 to 275.35) | 166.85  (95.10 to 269.77) | -0.09  (-0.29 to 0.11) | 0.363 | |
|  | | Senegal | 259.27  (159.47 to 412.38) | 722.86  (498.30 to 1006.03) | 3.26  (2.92 to 3.61) | <0.001 | 1159.65  (697.89 to 1799.86) | 925.41  (547.93 to 1468.36) | -1.97  (-2.79 to -1.15) | <0.001 | |
|  | | Serbia | 5.94  (0.00 to 9.88) | 2.56  (1.40 to 5.63) | -2.86  (-3.65 to -2.07) | <0.001 | 127.20  (71.38 to 206.79) | 125.53  (71.65 to 202.91) | -0.04  (-0.05 to -0.03) | <0.001 | |
|  | | Seychelles | 16.29  (11.07 to 23.45) | 72.48  (42.91 to 127.23) | 4.91  (4.62 to 5.2) | <0.001 | 426.54  (254.16 to 676.50) | 389.32  (237.19 to 598.08) | -0.28  (-0.37 to -0.18) | <0.001 | |
|  | | Sierra Leone | 253.59  (85.57 to 586.47) | 1822.22  (1247.02 to 2566.21) | 6.47  (6.05 to 6.88) | <0.001 | 1702.41  (1031.55 to 2606.16) | 1449.36  (865.54 to 2236.30) | -0.51  (-0.59 to -0.43) | <0.001 | |
|  | | Singapore | 3.70  (1.84 to 6.19) | 29.23  (14.69 to 47.91) | 6.82  (6.56 to 7.08) | <0.001 | 373.11  (213.70 to 603.91) | 343.97  (197.11 to 556.49) | -0.26  (-0.29 to -0.24) | <0.001 | |
|  | | Slovakia | 0.47  (0.33 to 0.64) | 1.79  (1.05 to 2.97) | 4.37  (4.23 to 4.5) | <0.001 | 76.43  (43.61 to 124.58) | 81.05  (47.39 to 131.39) | 0.21  (0.13 to 0.28) | <0.001 | |
|  | | Slovenia | 0.48  (0.00 to 0.79) | 4.32  (2.46 to 7.85) | 7.4  (7.09 to 7.71) | <0.001 | 122.98  (69.48 to 198.49) | 120.23  (67.81 to 194.56) | -0.07  (-0.09 to -0.06) | <0.001 | |
|  | | Solomon Islands | 8.16  (4.72 to 11.04) | 229.21  (156.68 to 296.55) | 11.28  (11.04 to 11.52) | <0.001 | 3976.35  (2352.54 to 6136.50) | 3947.79  (2378.63 to 6099.03) | -0.02  (-0.05 to 0.01) | 0.234 | |
|  | | Somalia | 46.13  (14.53 to 127.44) | 399.49  (172.34 to 816.55) | 7.12  (6.75 to 7.5) | <0.001 | 4830.23  (3011.74 to 7267.41) | 3460.24  (2168.01 to 5205.72) | -1  (-1.44 to -0.55) | <0.001 | |
|  | | South Africa | 681.76  (545.10 to 837.93) | 24877.99  (23281.99 to 26598.81) | 12.17  (11.31 to 13.04) | <0.001 | 9730.34  (6857.76 to 13368.76) | 4635.74  (2779.25 to 7207.58) | -2.38  (-2.61 to -2.15) | <0.001 | |
|  | | South Sudan | 328.25  (77.12 to 974.66) | 2908.26  (1004.83 to 5737.59) | 7.26  (6.8 to 7.74) | <0.001 | 6102.11  (3680.47 to 9354.02) | 5544.13  (3307.25 to 8570.29) | -0.31  (-0.42 to -0.2) | <0.001 | |
|  | | Spain | 156.51  (114.53 to 204.20) | 81.02  (55.69 to 109.56) | -2.13  (-2.24 to -2.02) | <0.001 | 148.72  (84.58 to 243.88) | 143.21  (80.88 to 233.19) | -0.1  (-0.21 to 0.02) | 0.091 | |
|  | | Sri Lanka | 3.37  (1.82 to 5.46) | 14.04  (6.31 to 26.98) | 4.68  (4.5 to 4.85) | <0.001 | 146.31  (84.59 to 234.85) | 130.65  (75.92 to 208.94) | -0.47  (-0.78 to -0.17) | 0.002 | |
|  | | Sudan | 62.06  (8.20 to 245.73) | 518.88  (128.66 to 1459.58) | 7.01  (6.72 to 7.3) | <0.001 | 1581.20  (897.86 to 2529.05) | 1596.18  (896.55 to 2550.84) | -0.12  (-0.58 to 0.34) | 0.61 | |
|  | | Suriname | 150.47  (104.52 to 219.41) | 1241.20  (777.34 to 1927.07) | 7.05  (6.91 to 7.18) | <0.001 | 287.78  (169.05 to 455.19) | 305.13  (186.71 to 469.62) | 0.18  (0.04 to 0.33) | 0.013 | |
|  | | Sweden | 11.82  (6.63 to 18.49) | 62.17  (30.86 to 96.12) | 5.5  (5.25 to 5.75) | <0.001 | 159.91  (89.41 to 260.43) | 151.47  (84.77 to 246.38) | -0.16  (-0.2 to -0.12) | <0.001 | |
|  | | Switzerland | 70.75  (40.16 to 112.57) | 115.24  (56.79 to 191.48) | 1.62  (1.46 to 1.79) | <0.001 | 99.46  (56.17 to 162.04) | 96.04  (54.26 to 157.26) | -0.09  (-0.18 to 0.01) | 0.094 | |
|  | | Syrian Arab Republic | 1.05  (0.77 to 1.41) | 2.73  (1.49 to 4.88) | 3.11  (2.99 to 3.24) | <0.001 | 448.04  (260.20 to 718.93) | 396.92  (229.34 to 632.99) | -0.39  (-0.4 to -0.37) | <0.001 | |
|  | | Taiwan  (Province of China) | 0.93  (0.56 to 1.27) | 5.77  (2.42 to 11.78) | 6.01  (5.78 to 6.25) | <0.001 | 313.28  (181.47 to 497.39) | 275.07  (157.76 to 448.96) | -0.42  (-0.46 to -0.38) | <0.001 | |
|  | | Tajikistan | 19.16  (12.11 to 29.64) | 95.79  (64.00 to 134.35) | 5.31  (5.07 to 5.55) | <0.001 | 138.83  (80.73 to 221.34) | 125.81  (73.93 to 199.80) | -0.25  (-0.76 to 0.26) | 0.337 | |
|  | | Thailand | 71.41  (40.84 to 112.41) | 841.97  (477.62 to 1353.64) | 8.14  (7.76 to 8.52) | <0.001 | 360.04  (209.70 to 577.19) | 325.22  (186.91 to 525.96) | -0.52  (-1.39 to 0.36) | 0.244 | |
|  | | Timor-Leste | 30.55  (22.49 to 40.07) | 280.26  (226.75 to 339.80) | 7.29  (6.79 to 7.8) | <0.001 | 588.20  (340.74 to 933.85) | 552.67  (323.09 to 872.55) | -0.22  (-0.26 to -0.17) | <0.001 | |
|  | | Togo | 1053.17  (581.85 to 1757.13) | 2522.71  (1861.80 to 3279.46) | 2.85  (2.56 to 3.14) | <0.001 | 1959.08  (1182.45 to 2964.27) | 1605.81  (962.68 to 2506.64) | -0.34  (-1.45 to 0.77) | 0.545 | |
|  | | Tokelau | 3.71  (2.23 to 4.98) | 115.85  (79.51 to 152.87) | 11.64  (11.29 to 12) | <0.001 | 2577.44  (1487.41 to 4147.39) | 2551.38  (1561.61 to 3900.35) | -0.02  (-0.1 to 0.06) | 0.595 | |
|  | | Tonga | 8.29  (4.91 to 11.21) | 284.70  (200.96 to 362.71) | 12.06  (11.89 to 12.23) | <0.001 | 1516.44  (880.77 to 2423.22) | 1483.62  (886.86 to 2320.29) | -0.04  (-0.27 to 0.19) | 0.729 | |
|  | | Trinidad and Tobago | 170.56  (93.98 to 293.92) | 1042.77  (815.11 to 1431.30) | 5.98  (5.83 to 6.13) | <0.001 | 552.93  (316.34 to 881.70) | 521.04  (305.66 to 832.15) | -0.26  (-0.38 to -0.15) | <0.001 | |
|  | | Tunisia | 0.31  (0.08 to 0.74) | 18.28  (11.59 to 24.81) | 13.98  (13.36 to 14.62) | <0.001 | 389.38  (222.90 to 624.65) | 374.34  (215.00 to 601.27) | -0.13  (-0.14 to -0.12) | <0.001 | |
|  | | Turkmenistan | 26.40  (19.20 to 34.13) | 44.75  (31.07 to 70.49) | 1.72  (1.62 to 1.82) | <0.001 | 183.52  (104.44 to 296.33) | 159.38  (91.87 to 253.52) | -0.46  (-0.49 to -0.43) | <0.001 | |
|  | | Tuvalu | 3.77  (2.21 to 5.29) | 118.83  (82.38 to 155.87) | 11.74  (11.57 to 11.92) | <0.001 | 1547.16  (856.41 to 2530.84) | 1621.91  (949.89 to 2554.23) | 0.16  (0.08 to 0.23) | <0.001 | |
|  | | Türkiye | 0.41  (0.00 to 0.73) | 7.11  (4.46 to 11.43) | 9.65  (9.47 to 9.82) | <0.001 | 615.17  (339.00 to 994.62) | 582.74  (323.68 to 944.49) | -0.17  (-0.18 to -0.17) | <0.001 | |
|  | | Uganda | 13753.90  (11000.80 to 16675.92) | 8112.25  (6348.03 to 9933.58) | -1.68  (-1.78 to -1.57) | <0.001 | 5104.31  (3048.12 to 7884.66) | 5177.43  (3130.37 to 8021.91) | 0.47  (-0.29 to 1.23) | 0.227 | |
|  | | Ukraine | 28.66  (16.29 to 48.30) | 786.32  (421.93 to 1406.43) | 11.4  (10.86 to 11.94) | <0.001 | 147.76  (83.03 to 239.74) | 143.58  (82.46 to 228.63) | -0.04  (-0.33 to 0.25) | 0.788 | |
|  | | United Arab Emirates | 1.95  (1.30 to 3.05) | 18.79  (13.02 to 24.95) | 7.57  (7.41 to 7.72) | <0.001 | 128.84  (73.90 to 208.60) | 125.22  (72.11 to 201.87) | -0.06  (-0.12 to 0) | 0.052 | |
|  | | United Kingdom | 18.11  (10.41 to 28.50) | 144.62  (85.83 to 206.50) | 6.96  (6.78 to 7.14) | <0.001 | 210.41  (120.36 to 338.06) | 217.10  (128.30 to 341.43) | 0.12  (-0.04 to 0.29) | 0.15 | |
|  | | United Republic of Tanzania | 6757.23  (4853.75 to 8955.07) | 8075.99  (5724.94 to 12023.18) | 0.56  (0.33 to 0.8) | <0.001 | 7238.93  (6092.07 to 8580.75) | 4323.96  (2605.00 to 6678.78) | -1.87  (-2.4 to -1.34) | <0.001 | |
|  | | United States of America | 211.32  (126.84 to 318.55) | 275.29  (139.19 to 428.13) | 0.86  (0.76 to 0.97) | <0.001 | 523.31  (301.27 to 841.59) | 488.68  (281.98 to 787.78) | -0.22  (-0.32 to -0.13) | <0.001 | |
|  | | United States Virgin Islands | 62.95  (40.20 to 95.24) | 160.24  (108.73 to 249.93) | 3.04  (2.9 to 3.18) | <0.001 | 933.27  (543.85 to 1477.88) | 1005.17  (611.82 to 1547.23) | 0.25  (0.22 to 0.28) | <0.001 | |
|  | | Uruguay | 32.12  (14.78 to 59.42) | 307.25  (143.61 to 499.01) | 7.51  (7.42 to 7.6) | <0.001 | 1172.74  (661.91 to 1896.14) | 1142.36  (651.03 to 1826.78) | -0.01  (-0.06 to 0.05) | 0.818 | |
|  | | Uzbekistan | 9.80  (6.16 to 13.86) | 101.22  (47.97 to 180.32) | 7.86  (7.66 to 8.06) | <0.001 | 170.44  (97.33 to 270.78) | 147.55  (83.95 to 238.41) | -0.47  (-0.49 to -0.45) | <0.001 | |
|  | | Vanuatu | 7.71  (4.72 to 10.35) | 227.05  (156.21 to 292.05) | 11.47  (11.23 to 11.72) | <0.001 | 3316.29  (1941.93 to 5195.68) | 3417.00  (2058.78 to 5286.82) | 0.32  (-0.11 to 0.74) | 0.142 | |
|  | | Venezuela  (Bolivarian Republic of) | 21.10  (15.01 to 29.32) | 252.11  (162.76 to 377.76) | 8.31  (8.07 to 8.56) | <0.001 | 1480.86  (837.06 to 2383.70) | 1594.91  (906.89 to 2540.03) | 1.44  (0.45 to 2.44) | 0.004 | |
|  | | Viet Nam | 17.35  (13.31 to 21.31) | 163.69  (107.38 to 245.81) | 7.49  (7.2 to 7.77) | <0.001 | 337.97  (195.07 to 538.18) | 318.42  (181.21 to 513.36) | -0.2  (-0.23 to -0.18) | <0.001 | |
|  | | Yemen | 2.95  (1.28 to 5.59) | 38.26  (23.06 to 55.80) | 8.63  (8.55 to 8.71) | <0.001 | 309.39  (176.33 to 498.13) | 298.07  (168.85 to 484.04) | -0.12  (-0.16 to -0.08) | <0.001 | |
|  | | Zambia | 10857.35  (7204.61 to 14952.53) | 13601.55  (11412.32 to 15749.03) | 0.77  (0.33 to 1.21) | 0.001 | 6513.92  (5121.59 to 8233.49) | 4731.29  (2852.35 to 7259.88) | -1.99  (-3.08 to -0.89) | <0.001 | |
|  | | Zimbabwe | 15032.48  (11816.29 to 18419.91) | 14349.24  (12260.26 to 16314.98) | -0.12  (-0.46 to 0.22) | 0.481 | 4862.09  (2885.76 to 7393.68) | 2751.32  (1651.29 to 4248.27) | -2.17  (-2.5 to -1.84) | <0.001 | |
| Age-standardized Mortality | | Afghanistan | 0.17  (0.03 to 0.37) | 0.46  (0.12 to 0.99) | 3.32  (2.74 to 3.91) | <0.001 | 0.002  (0.000 to 0.014) | 0.002  (0.000 to 0.011) | -0.11  (-0.55 to 0.34) | 0.643 | |
|  | | Albania | 0.02  (0.02 to 0.02) | 0.03  (0.03 to 0.03) | 0.58  (-0.01 to 1.18) | 0.052 | 0.003  (0.002 to 0.006) | 0.001  (0.000 to 0.003) | -3.18  (-3.73 to -2.63) | <0.001 | |
|  | | Algeria | 0.12  (0.06 to 0.20) | 0.55  (0.25 to 1.09) | 4.89  (4.36 to 5.43) | <0.001 | 0.000  (0.000 to 0.003) | 0.000  (0.000 to 0.002) | -1.26  (-1.44 to -1.07) | <0.001 | |
|  | | American Samoa | 0.86  (0.86 to 0.86) | 3.22  (3.21 to 3.23) | 4.02  (0.54 to 7.62) | 0.023 | 0.000  (0.000 to 0.001) | 0.000  (0.000 to 0.000) | -1.07  (-3.33 to 1.25) | 0.365 | |
|  | | Andorra | 0.10  (0.04 to 0.21) | 0.04  (0.02 to 0.09) | -3.01  (-3.55 to -2.46) | <0.001 | 0.002  (0.001 to 0.005) | 0.001  (0.000 to 0.002) | -2.36  (-2.67 to -2.04) | <0.001 | |
|  | | Angola | 4.21  (1.89 to 8.74) | 126.34  (72.89 to 208.54) | 11.35  (10.15 to 12.55) | <0.001 | 0.055  (0.015 to 0.126) | 0.027  (0.009 to 0.067) | -2.12  (-2.89 to -1.36) | <0.001 | |
|  | | Antigua and Barbuda | 8.11  (8.06 to 8.15) | 5.12  (5.10 to 5.13) | -0.8  (-1.56 to -0.03) | 0.043 | 0.006  (0.004 to 0.008) | 0.010  (0.007 to 0.013) | 1.73  (-0.1 to 3.58) | 0.064 | |
|  | | Argentina | 1.19  (1.19 to 1.19) | 2.79  (2.78 to 2.81) | 3.16  (2.19 to 4.14) | <0.001 | 0.006  (0.004 to 0.008) | 0.006  (0.004 to 0.008) | -0.3  (-0.96 to 0.37) | 0.386 | |
|  | | Armenia | 0.11  (0.11 to 0.11) | 0.28  (0.28 to 0.28) | 3.26  (2.37 to 4.17) | <0.001 | 0.015  (0.012 to 0.018) | 0.002  (0.001 to 0.004) | -6.38  (-8.25 to -4.47) | <0.001 | |
|  | | Australia | 0.13  (0.13 to 0.13) | 0.09  (0.09 to 0.09) | -2.15  (-3.72 to -0.56) | 0.008 | 0.003  (0.002 to 0.003) | 0.001  (0.001 to 0.001) | -3.84  (-5.32 to -2.32) | <0.001 | |
|  | | Austria | 0.48  (0.48 to 0.48) | 0.24  (0.24 to 0.24) | -2.52  (-4.04 to -0.97) | 0.001 | 0.001  (0.001 to 0.001) | 0.000  (0.000 to 0.001) | -3.13  (-3.95 to -2.31) | <0.001 | |
|  | | Azerbaijan | 0.29  (0.29 to 0.29) | 0.25  (0.24 to 0.25) | -0.66  (-1.5 to 0.19) | 0.126 | 0.026  (0.017 to 0.038) | 0.012  (0.006 to 0.024) | -2.47  (-3.27 to -1.68) | <0.001 | |
|  | | Bahamas | 24.30  (23.94 to 24.66) | 28.26  (27.77 to 28.76) | 0.37  (-1.17 to 1.94) | 0.637 | 0.011  (0.008 to 0.015) | 0.022  (0.014 to 0.032) | 2.32  (0.09 to 4.59) | 0.041 | |
|  | | Bahrain | 0.27  (0.27 to 0.27) | 1.16  (1.15 to 1.16) | 5.35  (-3.98 to 15.59) | 0.271 | 0.001  (0.000 to 0.001) | 0.001  (0.000 to 0.001) | 0.56  (-0.75 to 1.88) | 0.404 | |
|  | | Bangladesh | NA | 0.33  (0.16 to 0.60) | NA | NA | 0.021  (0.008 to 0.046) | 0.009  (0.003 to 0.021) | -2.76  (-3.5 to -2.01) | <0.001 | |
|  | | Barbados | 7.17  (7.14 to 7.19) | 6.65  (6.63 to 6.68) | 0.08  (-0.69 to 0.85) | 0.838 | 0.015  (0.011 to 0.020) | 0.015  (0.010 to 0.022) | 0.14  (-0.68 to 0.98) | 0.737 | |
|  | | Belarus | 0.75  (0.74 to 0.75) | 4.29  (4.28 to 4.30) | 5.82  (4.09 to 7.58) | <0.001 | 0.009  (0.007 to 0.012) | 0.007  (0.004 to 0.009) | -0.98  (-2.06 to 0.11) | 0.077 | |
|  | | Belgium | 0.82  (0.82 to 0.83) | 0.38  (0.37 to 0.38) | -2.55  (-3.89 to -1.19) | <0.001 | 0.002  (0.001 to 0.002) | 0.001  (0.000 to 0.001) | -3.22  (-3.69 to -2.75) | <0.001 | |
|  | | Belize | 12.90  (12.80 to 13.01) | 19.19  (18.97 to 19.41) | 2.21  (1.3 to 3.13) | <0.001 | 0.004  (0.003 to 0.005) | 0.010  (0.007 to 0.014) | 3.5  (2.03 to 5) | <0.001 | |
|  | | Benin | 0.91  (0.19 to 2.58) | 20.85  (10.34 to 35.41) | 8.91  (5.64 to 12.29) | <0.001 | 0.012  (0.004 to 0.028) | 0.008  (0.003 to 0.020) | -1.24  (-1.43 to -1.04) | <0.001 | |
|  | | Bermuda | 8.60  (8.56 to 8.65) | 4.77  (4.75 to 4.78) | -1.05  (-2.01 to -0.07) | 0.036 | 0.003  (0.002 to 0.004) | 0.004  (0.003 to 0.007) | 0.85  (-0.09 to 1.8) | 0.076 | |
|  | | Bhutan | 0.22  (0.05 to 0.58) | 1.02  (0.35 to 2.38) | 4.78  (3.16 to 6.42) | <0.001 | 0.042  (0.011 to 0.106) | 0.025  (0.009 to 0.060) | -1.71  (-2.12 to -1.29) | <0.001 | |
|  | | Bolivia  (Plurinational State of) | 0.09  (0.01 to 0.41) | 2.49  (0.93 to 5.10) | 10.99  (10.19 to 11.79) | <0.001 | 0.016  (0.006 to 0.036) | 0.008  (0.003 to 0.019) | -2.15  (-2.29 to -2) | <0.001 | |
|  | | Bosnia and Herzegovina | 0.11  (0.11 to 0.11) | 0.16  (0.16 to 0.16) | 1.83  (1.33 to 2.33) | <0.001 | 0.001  (0.001 to 0.002) | 0.001  (0.000 to 0.001) | -2.05  (-3.06 to -1.02) | <0.001 | |
|  | | Botswana | 143.75  (65.79 to 275.48) | 267.61  (130.43 to 480.74) | 1.91  (0.88 to 2.94) | <0.001 | 0.053  (0.019 to 0.121) | 0.020  (0.006 to 0.055) | -3.15  (-3.86 to -2.44) | <0.001 | |
|  | | Brazil | 3.05  (3.04 to 3.05) | 4.50  (4.49 to 4.51) | 1.28  (0.23 to 2.34) | 0.017 | 0.020  (0.018 to 0.023) | 0.017  (0.015 to 0.019) | -0.51  (-0.85 to -0.16) | 0.004 | |
|  | | Brunei Darussalam | 0.10  (0.10 to 0.10) | 0.29  (0.29 to 0.29) | 3.94  (2.91 to 4.97) | <0.001 | 0.003  (0.001 to 0.006) | 0.002  (0.001 to 0.004) | -1.46  (-1.76 to -1.16) | <0.001 | |
|  | | Bulgaria | 0.34  (0.34 to 0.34) | 0.46  (0.46 to 0.46) | 1.1  (-0.17 to 2.38) | 0.091 | 0.002  (0.001 to 0.002) | 0.001  (0.001 to 0.002) | -1.07  (-1.91 to -0.22) | 0.014 | |
|  | | Burkina Faso | 186.88  (96.96 to 319.94) | 13.58  (6.87 to 22.84) | -8.21  (-8.86 to -7.55) | <0.001 | 0.018  (0.006 to 0.040) | 0.011  (0.004 to 0.025) | -1.54  (-1.77 to -1.32) | <0.001 | |
|  | | Burundi | 143.21  (61.38 to 332.34) | 20.31  (9.64 to 36.82) | -6.31  (-7.64 to -4.96) | <0.001 | 0.077  (0.025 to 0.221) | 0.040  (0.010 to 0.140) | -2.16  (-2.42 to -1.9) | <0.001 | |
|  | | Cabo Verde | 11.42  (3.54 to 27.74) | 8.36  (2.26 to 25.18) | -1.18  (-1.79 to -0.56) | <0.001 | 0.007  (0.003 to 0.015) | 0.002  (0.001 to 0.005) | -3.78  (-4.43 to -3.13) | <0.001 | |
|  | | Cambodia | NA | 5.84  (2.04 to 11.79) | NA | NA | 0.005  (0.001 to 0.014) | 0.005  (0.001 to 0.013) | 0.12  (-0.04 to 0.27) | 0.132 | |
|  | | Cameroon | 24.41  (11.56 to 45.63) | 97.27  (58.14 to 148.31) | 4.57  (3.69 to 5.47) | <0.001 | 0.030  (0.005 to 0.076) | 0.016  (0.004 to 0.037) | -2.13  (-2.27 to -1.99) | <0.001 | |
|  | | Canada | 0.51  (0.51 to 0.51) | 0.25  (0.25 to 0.25) | -2.31  (-3.71 to -0.89) | 0.001 | 0.002  (0.002 to 0.003) | 0.001  (0.001 to 0.002) | -1.7  (-2.14 to -1.26) | <0.001 | |
|  | | Central African Republic | 134.47  (49.50 to 273.90) | 140.87  (76.24 to 242.44) | -0.13  (-1.49 to 1.25) | 0.851 | 0.061  (0.016 to 0.137) | 0.051  (0.016 to 0.118) | -0.6  (-0.93 to -0.26) | 0.001 | |
|  | | Chad | 21.92  (10.00 to 44.90) | 32.07  (15.19 to 59.46) | 1.08  (-0.18 to 2.36) | 0.094 | 0.020  (0.005 to 0.049) | 0.016  (0.005 to 0.039) | -0.45  (-0.77 to -0.13) | 0.006 | |
|  | | Chile | 0.47  (0.47 to 0.47) | 1.22  (1.21 to 1.22) | 3.73  (2.24 to 5.25) | <0.001 | 0.005  (0.004 to 0.006) | 0.004  (0.003 to 0.005) | -0.77  (-1.42 to -0.11) | 0.022 | |
|  | | China | 0.09  (0.01 to 0.16) | 0.89  (0.66 to 1.14) | 7.75  (6.8 to 8.7) | <0.001 | 0.005  (0.002 to 0.008) | 0.001  (0.001 to 0.002) | -4.67  (-5.45 to -3.88) | <0.001 | |
|  | | Colombia | 1.11  (1.11 to 1.11) | 3.20  (3.19 to 3.21) | 3.41  (2.22 to 4.61) | <0.001 | 0.018  (0.013 to 0.024) | 0.015  (0.011 to 0.021) | -0.49  (-0.88 to -0.11) | 0.012 | |
|  | | Comoros | 0.04  (0.00 to 0.16) | 0.20  (0.08 to 0.39) | 4.96  (3.69 to 6.25) | <0.001 | 0.110  (0.028 to 0.274) | 0.059  (0.020 to 0.153) | -2.16  (-4.9 to 0.65) | 0.13 | |
|  | | Congo | 136.49  (60.65 to 256.87) | 126.18  (72.35 to 209.58) | -0.27  (-1.42 to 0.89) | 0.647 | 0.045  (0.013 to 0.105) | 0.027  (0.009 to 0.063) | -1.56  (-2.31 to -0.81) | <0.001 | |
|  | | Cook Islands | 0.09  (0.02 to 0.20) | 2.57  (0.89 to 4.99) | 11.31  (10.58 to 12.04) | <0.001 | 0.002  (0.001 to 0.005) | 0.001  (0.000 to 0.002) | -3.18  (-3.39 to -2.98) | <0.001 | |
|  | | Costa Rica | 1.46  (1.46 to 1.46) | 2.42  (2.41 to 2.42) | 1.56  (0.85 to 2.27) | <0.001 | 0.011  (0.008 to 0.014) | 0.013  (0.009 to 0.017) | 0.53  (-0.3 to 1.37) | 0.209 | |
|  | | Coted'Ivoire | 217.72  (102.24 to 413.07) | 61.97  (33.17 to 102.22) | -4.36  (-5.28 to -3.43) | <0.001 | 0.017  (0.005 to 0.039) | 0.012  (0.004 to 0.028) | -1  (-1.6 to -0.4) | 0.001 | |
|  | | Croatia | 0.10  (0.10 to 0.10) | 0.09  (0.09 to 0.10) | -0.07  (-0.64 to 0.5) | 0.8 | 0.001  (0.001 to 0.002) | 0.001  (0.000 to 0.001) | -2.49  (-2.94 to -2.04) | <0.001 | |
|  | | Cuba | 0.77  (0.77 to 0.78) | 0.91  (0.91 to 0.91) | 0.77  (-0.43 to 1.99) | 0.209 | 0.015  (0.012 to 0.020) | 0.032  (0.022 to 0.044) | 2.47  (1.01 to 3.96) | 0.001 | |
|  | | Cyprus | 0.17  (0.17 to 0.17) | 0.29  (0.29 to 0.29) | 1.69  (0.85 to 2.53) | <0.001 | 0.002  (0.001 to 0.004) | 0.000  (0.000 to 0.001) | -3.86  (-5.69 to -2) | <0.001 | |
|  | | Czechia | 0.06  (0.06 to 0.06) | 0.06  (0.06 to 0.06) | 0.38  (-1.39 to 2.18) | 0.674 | 0.003  (0.002 to 0.004) | 0.002  (0.001 to 0.003) | -1.82  (-2.39 to -1.25) | <0.001 | |
|  | | Democratic People's Republic of Korea | 0.01  (0.00 to 0.04) | 1.24  (0.20 to 4.71) | 14.97  (13.26 to 16.7) | <0.001 | 0.002  (0.001 to 0.005) | 0.002  (0.001 to 0.005) | 0.39  (0.24 to 0.55) | <0.001 | |
|  | | Democratic Republic of the Congo | 88.76  (49.49 to 150.87) | 14.54  (7.56 to 25.69) | -6.02  (-6.81 to -5.22) | <0.001 | 0.045  (0.013 to 0.111) | 0.024  (0.008 to 0.061) | -2  (-2.44 to -1.55) | <0.001 | |
|  | | Denmark | 0.41  (0.41 to 0.41) | 0.20  (0.19 to 0.20) | -2.29  (-3.45 to -1.11) | <0.001 | 0.003  (0.002 to 0.005) | 0.001  (0.001 to 0.002) | -2.58  (-3.23 to -1.92) | <0.001 | |
|  | | Djibouti | 0.23  (0.02 to 0.87) | 100.92  (57.50 to 166.05) | 22.29  (20.37 to 24.23) | <0.001 | 0.044  (0.012 to 0.137) | 0.036  (0.010 to 0.121) | -0.52  (-0.94 to -0.09) | 0.017 | |
|  | | Dominica | 5.37  (5.35 to 5.40) | 5.27  (5.26 to 5.29) | 0.32  (-0.48 to 1.12) | 0.436 | 0.001  (0.001 to 0.002) | 0.002  (0.001 to 0.004) | 1.73  (1.17 to 2.28) | <0.001 | |
|  | | Dominican Republic | 5.36  (1.64 to 13.38) | 5.95  (1.49 to 13.82) | 0.27  (-0.64 to 1.19) | 0.56 | 0.048  (0.023 to 0.087) | 0.045  (0.016 to 0.097) | -0.21  (-0.71 to 0.29) | 0.417 | |
|  | | Ecuador | 0.82  (0.82 to 0.83) | 4.05  (4.03 to 4.07) | 5.71  (4.46 to 6.97) | <0.001 | 0.004  (0.003 to 0.005) | 0.004  (0.002 to 0.008) | 0.18  (-1.35 to 1.74) | 0.814 | |
|  | | Egypt | 0.19  (0.19 to 0.19) | 0.50  (0.50 to 0.50) | 3.16  (2.41 to 3.93) | <0.001 | 0.005  (0.002 to 0.012) | 0.002  (0.001 to 0.005) | -2.33  (-3.09 to -1.56) | <0.001 | |
|  | | El Salvador | 2.14  (2.14 to 2.15) | 4.02  (4.00 to 4.03) | 2.04  (0.65 to 3.45) | 0.004 | 0.005  (0.003 to 0.008) | 0.004  (0.002 to 0.007) | -0.75  (-2.02 to 0.53) | 0.249 | |
|  | | Equatorial Guinea | 10.74  (4.83 to 23.98) | 247.11  (117.66 to 479.24) | 10.67  (9.38 to 11.98) | <0.001 | 0.060  (0.016 to 0.146) | 0.024  (0.007 to 0.059) | -2.87  (-3.51 to -2.22) | <0.001 | |
|  | | Eritrea | 27.83  (11.58 to 60.80) | 21.50  (12.73 to 34.17) | -0.93  (-1.6 to -0.26) | 0.007 | 0.082  (0.024 to 0.251) | 0.052  (0.016 to 0.157) | -1.49  (-1.75 to -1.24) | <0.001 | |
|  | | Estonia | 0.47  (0.46 to 0.47) | 2.86  (2.86 to 2.88) | 6.02  (4.98 to 7.07) | <0.001 | 0.015  (0.011 to 0.020) | 0.007  (0.004 to 0.010) | -2.78  (-3.63 to -1.93) | <0.001 | |
|  | | Eswatini | 4.70  (1.48 to 12.94) | 474.62  (213.62 to 913.64) | 15.59  (13.77 to 17.43) | <0.001 | 0.050  (0.018 to 0.109) | 0.029  (0.008 to 0.076) | -1.73  (-2.15 to -1.31) | <0.001 | |
|  | | Ethiopia | 33.23  (16.97 to 62.45) | 25.31  (14.11 to 40.95) | -0.78  (-1.9 to 0.35) | 0.176 | 0.188  (0.058 to 0.392) | 0.055  (0.020 to 0.193) | -3.85  (-4.04 to -3.65) | <0.001 | |
|  | | Fiji | 2.37  (2.36 to 2.37) | 4.10  (4.09 to 4.11) | 1.84  (0.64 to 3.06) | 0.003 | 0.013  (0.005 to 0.032) | 0.008  (0.003 to 0.018) | -1.75  (-2.77 to -0.72) | 0.001 | |
|  | | Finland | 0.19  (0.19 to 0.19) | 0.11  (0.11 to 0.12) | -1.76  (-2.88 to -0.63) | 0.002 | 0.005  (0.004 to 0.007) | 0.002  (0.002 to 0.003) | -2.69  (-3.19 to -2.19) | <0.001 | |
|  | | France | 2.30  (2.30 to 2.31) | 0.41  (0.41 to 0.41) | -5.55  (-7.27 to -3.79) | <0.001 | 0.002  (0.001 to 0.002) | 0.001  (0.000 to 0.001) | -3.06  (-3.48 to -2.64) | <0.001 | |
|  | | Gabon | 33.80  (16.39 to 64.08) | 100.69  (50.97 to 170.64) | 3.48  (2.18 to 4.79) | <0.001 | 0.034  (0.010 to 0.080) | 0.015  (0.005 to 0.040) | -2.48  (-2.78 to -2.17) | <0.001 | |
|  | | Gambia | 4.01  (1.83 to 8.02) | 75.64  (37.20 to 134.81) | 9.77  (8.66 to 10.9) | <0.001 | 0.015  (0.004 to 0.035) | 0.014  (0.004 to 0.033) | -0.26  (-2.05 to 1.56) | 0.775 | |
|  | | Georgia | 0.19  (0.19 to 0.19) | 0.63  (0.62 to 0.63) | 3.78  (3.08 to 4.48) | <0.001 | 0.006  (0.005 to 0.008) | 0.001  (0.001 to 0.002) | -4.78  (-5.72 to -3.83) | <0.001 | |
|  | | Germany | 0.77  (0.76 to 0.77) | 0.25  (0.25 to 0.25) | -3.61  (-5.61 to -1.56) | 0.001 | 0.002  (0.001 to 0.002) | 0.001  (0.000 to 0.001) | -3.21  (-3.72 to -2.7) | <0.001 | |
|  | | Ghana | 32.65  (16.50 to 57.23) | 74.03  (44.24 to 115.89) | 2.78  (1.97 to 3.6) | <0.001 | 0.022  (0.006 to 0.050) | 0.011  (0.004 to 0.025) | -2.18  (-2.46 to -1.9) | <0.001 | |
|  | | Greece | 0.18  (0.18 to 0.18) | 0.12  (0.12 to 0.12) | -1.4  (-2.41 to -0.37) | 0.008 | 0.001  (0.000 to 0.001) | 0.000  (0.000 to 0.000) | -2.22  (-2.82 to -1.61) | <0.001 | |
|  | | Greenland | 2.17  (2.17 to 2.17) | 1.61  (1.60 to 1.61) | -1.32  (-2.72 to 0.09) | 0.067 | 0.000  (0.000 to 0.000) | 0.000  (0.000 to 0.000) | 0.47  (-0.93 to 1.89) | 0.515 | |
|  | | Grenada | 5.66  (5.63 to 5.68) | 4.28  (4.26 to 4.29) | 0.15  (-2.72 to 3.11) | 0.919 | 0.013  (0.009 to 0.018) | 0.023  (0.015 to 0.032) | 1.96  (0.37 to 3.56) | 0.015 | |
|  | | Guam | 0.92  (0.91 to 0.92) | 5.55  (5.52 to 5.57) | 6.88  (3.99 to 9.86) | <0.001 | 0.002  (0.001 to 0.003) | 0.001  (0.000 to 0.001) | -3.3  (-6.29 to -0.21) | 0.037 | |
|  | | Guatemala | 4.87  (4.86 to 4.88) | 3.25  (3.23 to 3.27) | -1.66  (-2.39 to -0.93) | <0.001 | 0.019  (0.014 to 0.025) | 0.015  (0.010 to 0.021) | -0.57  (-1.24 to 0.1) | 0.094 | |
|  | | Guinea | 10.03  (4.88 to 20.09) | 38.56  (21.38 to 62.73) | 4.21  (3.2 to 5.23) | <0.001 | 0.021  (0.006 to 0.050) | 0.016  (0.005 to 0.037) | -0.94  (-1.32 to -0.55) | <0.001 | |
|  | | Guinea-Bissau | 7.36  (3.21 to 14.38) | 87.57  (35.19 to 176.69) | 8.16  (7.18 to 9.14) | <0.001 | 0.046  (0.007 to 0.120) | 0.026  (0.007 to 0.062) | -1.77  (-1.93 to -1.61) | <0.001 | |
|  | | Guyana | 17.71  (17.57 to 17.85) | 28.50  (27.98 to 29.01) | 1.49  (-0.5 to 3.52) | 0.144 | 0.024  (0.016 to 0.034) | 0.046  (0.027 to 0.073) | 2.39  (0.24 to 4.6) | 0.029 | |
|  | | Haiti | 142.91  (67.29 to 261.33) | 44.07  (21.71 to 74.29) | -3.71  (-4.96 to -2.44) | <0.001 | 0.062  (0.021 to 0.142) | 0.060  (0.016 to 0.137) | -0.13  (-0.44 to 0.19) | 0.421 | |
|  | | Honduras | 7.38  (7.35 to 7.40) | 2.37  (2.35 to 2.38) | -3.51  (-3.64 to -3.39) | <0.001 | 0.054  (0.023 to 0.115) | 0.026  (0.008 to 0.061) | -2.46  (-2.81 to -2.11) | <0.001 | |
|  | | Hungary | 0.46  (0.46 to 0.46) | 0.33  (0.33 to 0.33) | -0.84  (-2.3 to 0.64) | 0.264 | 0.010  (0.008 to 0.013) | 0.005  (0.003 to 0.007) | -2.52  (-3.3 to -1.73) | <0.001 | |
|  | | Iceland | 0.38  (0.38 to 0.38) | 0.24  (0.24 to 0.24) | -1.42  (-2.24 to -0.59) | 0.001 | 0.001  (0.001 to 0.002) | 0.001  (0.000 to 0.001) | -2.36  (-3.84 to -0.86) | 0.002 | |
|  | | India | 0.03  (0.01 to 0.06) | 3.45  (2.24 to 4.86) | 17.06  (15.5 to 18.65) | <0.001 | 0.058  (0.025 to 0.082) | 0.028  (0.013 to 0.040) | -2.41  (-2.86 to -1.95) | <0.001 | |
|  | | Indonesia | 0.22  (0.22 to 0.22) | 1.00  (0.95 to 1.08) | 5.03  (2.28 to 7.86) | <0.001 | 0.003  (0.002 to 0.006) | 0.002  (0.001 to 0.003) | -2.29  (-2.41 to -2.16) | <0.001 | |
|  | | Iran  (Islamic Republic of) | 0.04  (0.04 to 0.04) | 0.36  (0.36 to 0.36) | 7.47  (4.39 to 10.65) | <0.001 | 0.000  (0.000 to 0.001) | 0.001  (0.000 to 0.001) | 0.35  (-0.34 to 1.04) | 0.32 | |
|  | | Iraq | 0.07  (0.07 to 0.07) | 0.42  (0.42 to 0.42) | 6.08  (5.73 to 6.43) | <0.001 | 0.003  (0.001 to 0.008) | 0.001  (0.000 to 0.003) | -2.43  (-2.61 to -2.26) | <0.001 | |
|  | | Ireland | 0.28  (0.28 to 0.28) | 0.16  (0.16 to 0.16) | -2.03  (-2.6 to -1.46) | <0.001 | 0.002  (0.001 to 0.002) | 0.001  (0.000 to 0.001) | -3.33  (-3.96 to -2.69) | <0.001 | |
|  | | Israel | 0.56  (0.56 to 0.56) | 0.30  (0.29 to 0.30) | -2.19  (-3.73 to -0.63) | 0.006 | 0.001  (0.001 to 0.001) | 0.000  (0.000 to 0.000) | -3.43  (-4.04 to -2.81) | <0.001 | |
|  | | Italy | 2.10  (2.10 to 2.10) | 0.43  (0.43 to 0.43) | -5.12  (-7.61 to -2.56) | <0.001 | 0.001  (0.001 to 0.001) | 0.000  (0.000 to 0.001) | -2.09  (-2.98 to -1.18) | <0.001 | |
|  | | Jamaica | 11.18  (11.10 to 11.26) | 15.42  (15.27 to 15.57) | 1.09  (0.08 to 2.12) | 0.035 | 0.011  (0.008 to 0.014) | 0.018  (0.011 to 0.027) | 1.87  (-0.04 to 3.82) | 0.055 | |
|  | | Japan | 0.01  (0.01 to 0.01) | 0.03  (0.03 to 0.03) | 3.76  (3.11 to 4.41) | <0.001 | 0.000  (0.000 to 0.000) | 0.001  (0.000 to 0.001) | 1.32  (0.75 to 1.89) | <0.001 | |
|  | | Jordan | 0.07  (0.07 to 0.07) | 0.36  (0.36 to 0.36) | 5.61  (4.81 to 6.41) | <0.001 | 0.000  (0.000 to 0.002) | 0.000  (0.000 to 0.001) | -2.43  (-3.23 to -1.63) | <0.001 | |
|  | | Kazakhstan | 0.47  (0.47 to 0.47) | 1.24  (1.23 to 1.24) | 3.09  (2.62 to 3.56) | <0.001 | 0.014  (0.011 to 0.019) | 0.004  (0.003 to 0.006) | -3.9  (-5.14 to -2.64) | <0.001 | |
|  | | Kenya | 157.88  (83.17 to 270.99) | 83.94  (50.70 to 127.16) | -2.1  (-3.08 to -1.12) | <0.001 | 0.051  (0.024 to 0.093) | 0.036  (0.017 to 0.076) | -1.15  (-1.53 to -0.76) | <0.001 | |
|  | | Kiribati | 2.62  (2.61 to 2.63) | 3.27  (3.25 to 3.29) | 0.72  (-3.03 to 4.62) | 0.709 | 0.006  (0.002 to 0.012) | 0.004  (0.002 to 0.010) | -0.74  (-0.85 to -0.63) | <0.001 | |
|  | | Kuwait | 0.12  (0.12 to 0.12) | 0.10  (0.10 to 0.10) | -0.62  (-1.36 to 0.13) | 0.101 | 0.000  (0.000 to 0.000) | 0.002  (0.002 to 0.003) | 13.06  (8.49 to 17.83) | <0.001 | |
|  | | Kyrgyzstan | 1.04  (1.04 to 1.05) | 2.77  (2.76 to 2.79) | 3.16  (2.08 to 4.24) | <0.001 | 0.018  (0.013 to 0.023) | 0.003  (0.002 to 0.005) | -5.14  (-6.22 to -4.04) | <0.001 | |
|  | | Lao People's Democratic Republic | 0.01  (0.00 to 0.01) | 1.66  (0.61 to 3.56) | 20.29  (18.02 to 22.6) | <0.001 | 0.005  (0.001 to 0.016) | 0.006  (0.002 to 0.016) | 0.66  (0.45 to 0.87) | <0.001 | |
|  | | Latvia | 1.60  (1.59 to 1.61) | 11.11  (11.04 to 11.18) | 6.48  (5.11 to 7.86) | <0.001 | 0.020  (0.015 to 0.026) | 0.009  (0.006 to 0.015) | -2.36  (-2.98 to -1.73) | <0.001 | |
|  | | Lebanon | 0.20  (0.10 to 0.33) | 1.18  (0.66 to 1.89) | 5.91  (5.76 to 6.06) | <0.001 | 0.004  (0.002 to 0.010) | 0.001  (0.001 to 0.003) | -3.49  (-3.86 to -3.12) | <0.001 | |
|  | | Lesotho | 56.81  (26.94 to 103.67) | 514.39  (278.78 to 837.44) | 6.99  (4.61 to 9.43) | <0.001 | 0.029  (0.009 to 0.076) | 0.034  (0.011 to 0.082) | 0.43  (-0.07 to 0.94) | 0.093 | |
|  | | Liberia | 9.18  (3.58 to 22.92) | 36.31  (19.97 to 59.94) | 4.41  (3.28 to 5.56) | <0.001 | 0.027  (0.006 to 0.064) | 0.023  (0.003 to 0.061) | -0.55  (-0.93 to -0.17) | 0.004 | |
|  | | Libya | 0.22  (0.01 to 1.05) | 1.17  (0.01 to 11.04) | 5.57  (5.33 to 5.81) | <0.001 | 0.000  (0.000 to 0.002) | 0.000  (0.000 to 0.002) | 0.23  (-0.36 to 0.83) | 0.44 | |
|  | | Lithuania | 1.71  (1.70 to 1.71) | 10.36  (10.31 to 10.42) | 6.43  (6.14 to 6.73) | <0.001 | 0.007  (0.005 to 0.010) | 0.004  (0.003 to 0.007) | -2.12  (-3.08 to -1.16) | <0.001 | |
|  | | Luxembourg | 0.55  (0.55 to 0.55) | 0.29  (0.29 to 0.29) | -2.22  (-2.98 to -1.46) | <0.001 | 0.002  (0.001 to 0.002) | 0.001  (0.000 to 0.001) | -3.69  (-4.42 to -2.95) | <0.001 | |
|  | | Madagascar | 0.01  (0.00 to 0.04) | 18.98  (9.62 to 33.33) | 26.44  (24.92 to 27.96) | <0.001 | 0.122  (0.035 to 0.279) | 0.090  (0.033 to 0.194) | -1  (-1.46 to -0.55) | <0.001 | |
|  | | Malawi | 256.99  (129.46 to 450.75) | 155.04  (80.72 to 261.65) | -1.7  (-2.34 to -1.06) | <0.001 | 0.082  (0.027 to 0.183) | 0.056  (0.020 to 0.130) | -1.17  (-1.65 to -0.69) | <0.001 | |
|  | | Malaysia | 0.91  (0.91 to 0.91) | 2.62  (2.60 to 2.63) | 3.64  (2.68 to 4.61) | <0.001 | 0.006  (0.002 to 0.013) | 0.003  (0.001 to 0.007) | -1.63  (-2.36 to -0.88) | <0.001 | |
|  | | Maldives | 0.14  (0.14 to 0.14) | 0.88  (0.88 to 0.88) | 6.44  (4.74 to 8.17) | <0.001 | 0.034  (0.011 to 0.075) | 0.009  (0.003 to 0.019) | -4.57  (-4.97 to -4.17) | <0.001 | |
|  | | Mali | 10.18  (4.43 to 21.76) | 33.52  (17.90 to 58.52) | 3.9  (2.43 to 5.39) | <0.001 | 0.033  (0.008 to 0.080) | 0.022  (0.006 to 0.053) | -1.28  (-1.52 to -1.04) | <0.001 | |
|  | | Malta | 0.35  (0.35 to 0.35) | 0.22  (0.22 to 0.22) | -1.46  (-2.69 to -0.22) | 0.021 | 0.001  (0.001 to 0.001) | 0.000  (0.000 to 0.001) | -2.34  (-3.18 to -1.5) | <0.001 | |
|  | | Marshall Islands | 0.20  (0.04 to 0.47) | 7.45  (3.26 to 13.52) | 12.08  (11.05 to 13.12) | <0.001 | 0.004  (0.001 to 0.010) | 0.004  (0.001 to 0.009) | -0.36  (-0.62 to -0.09) | 0.009 | |
|  | | Mauritania | 0.05  (0.02 to 0.08) | 0.05  (0.02 to 0.10) | -0.16  (-0.67 to 0.36) | 0.553 | 0.019  (0.004 to 0.051) | 0.010  (0.003 to 0.023) | -2.21  (-2.4 to -2.02) | <0.001 | |
|  | | Mauritius | 0.25  (0.25 to 0.25) | 3.10  (3.08 to 3.11) | 14.09  (9.85 to 18.5) | <0.001 | 0.000  (0.000 to 0.001) | 0.005  (0.004 to 0.007) | 8.92  (7.5 to 10.35) | <0.001 | |
|  | | Mexico | 1.62  (1.62 to 1.62) | 2.21  (2.21 to 2.21) | 1.11  (0.75 to 1.46) | <0.001 | 0.018  (0.017 to 0.020) | 0.020  (0.015 to 0.029) | 0.33  (-0.04 to 0.69) | 0.078 | |
|  | | Micronesia  (Federated States of) | 0.21  (0.04 to 0.47) | 7.86  (3.37 to 14.28) | 12.22  (11.2 to 13.25) | <0.001 | 0.005  (0.002 to 0.012) | 0.004  (0.001 to 0.009) | -1.1  (-1.24 to -0.97) | <0.001 | |
|  | | Monaco | 0.24  (0.08 to 0.51) | 0.11  (0.04 to 0.23) | -2.51  (-3.1 to -1.92) | <0.001 | 0.000  (0.000 to 0.001) | 0.000  (0.000 to 0.001) | -1.59  (-1.65 to -1.52) | <0.001 | |
|  | | Mongolia | 0.11  (0.11 to 0.11) | 0.24  (0.24 to 0.24) | 2.83  (2.48 to 3.19) | <0.001 | 0.040  (0.014 to 0.096) | 0.015  (0.006 to 0.031) | -3.25  (-3.81 to -2.68) | <0.001 | |
|  | | Montenegro | 0.14  (0.14 to 0.14) | 0.15  (0.15 to 0.15) | 0.23  (-0.49 to 0.94) | 0.536 | 0.001  (0.001 to 0.002) | 0.001  (0.001 to 0.002) | -1.13  (-1.97 to -0.27) | 0.01 | |
|  | | Morocco | 0.45  (0.17 to 0.97) | 0.77  (0.31 to 1.57) | 1.57  (0.97 to 2.16) | <0.001 | 0.001  (0.000 to 0.007) | 0.001  (0.000 to 0.005) | -0.6  (-1.33 to 0.13) | 0.107 | |
|  | | Mozambique | 19.23  (12.10 to 31.13) | 343.17  (307.27 to 388.68) | 9.58  (8.92 to 10.24) | <0.001 | 0.175  (0.053 to 0.407) | 0.085  (0.029 to 0.203) | -2.41  (-2.68 to -2.14) | <0.001 | |
|  | | Myanmar | 0.09  (0.02 to 0.24) | 8.41  (3.50 to 14.86) | 16.18  (14.22 to 18.17) | <0.001 | 0.008  (0.002 to 0.028) | 0.008  (0.003 to 0.018) | -0.14  (-0.29 to 0.01) | 0.077 | |
|  | | Namibia | 34.34  (15.66 to 64.33) | 182.04  (96.25 to 313.65) | 5.22  (3.71 to 6.76) | <0.001 | 0.034  (0.012 to 0.088) | 0.018  (0.005 to 0.052) | -1.94  (-2.66 to -1.22) | <0.001 | |
|  | | Nauru | 0.09  (0.02 to 0.19) | 2.66  (0.92 to 5.33) | 11.62  (10.74 to 12.5) | <0.001 | 0.005  (0.002 to 0.011) | 0.004  (0.001 to 0.008) | -0.88  (-1.05 to -0.7) | <0.001 | |
|  | | Nepal | NA | 0.92  (0.34 to 2.02) | NA | NA | 0.043  (0.015 to 0.103) | 0.023  (0.008 to 0.051) | -2.05  (-2.21 to -1.88) | <0.001 | |
|  | | Netherlands | 0.41  (0.41 to 0.41) | 0.20  (0.20 to 0.20) | -2.08  (-3.46 to -0.69) | 0.004 | 0.001  (0.001 to 0.002) | 0.001  (0.000 to 0.001) | -2.73  (-3.04 to -2.43) | <0.001 | |
|  | | New Zealand | 0.16  (0.16 to 0.16) | 0.07  (0.07 to 0.07) | -2.8  (-3.79 to -1.8) | <0.001 | 0.002  (0.001 to 0.002) | 0.001  (0.001 to 0.001) | -2.66  (-7.34 to 2.25) | 0.283 | |
|  | | Nicaragua | 1.08  (1.08 to 1.08) | 2.12  (2.12 to 2.13) | 2.04  (1.29 to 2.8) | <0.001 | 0.009  (0.005 to 0.015) | 0.005  (0.003 to 0.009) | -1.48  (-2.07 to -0.89) | <0.001 | |
|  | | Niger | 6.04  (2.70 to 13.18) | 6.18  (2.76 to 11.83) | 0.04  (-1.19 to 1.28) | 0.954 | 0.012  (0.004 to 0.033) | 0.007  (0.002 to 0.022) | -1.88  (-2.12 to -1.64) | <0.001 | |
|  | | Nigeria | 16.87  (9.11 to 29.50) | 56.04  (36.85 to 81.62) | 3.94  (2.99 to 4.9) | <0.001 | 0.012  (0.004 to 0.019) | 0.007  (0.003 to 0.015) | -1.43  (-1.57 to -1.29) | <0.001 | |
|  | | Niue | 0.09  (0.02 to 0.21) | 2.19  (0.69 to 4.53) | 10.56  (9.25 to 11.89) | <0.001 | 0.002  (0.001 to 0.006) | 0.002  (0.001 to 0.004) | -0.71  (-0.95 to -0.47) | <0.001 | |
|  | | North Macedonia | 0.03  (0.03 to 0.03) | 0.04  (0.04 to 0.04) | 0.1  (-0.75 to 0.97) | 0.813 | 0.003  (0.001 to 0.007) | 0.001  (0.000 to 0.003) | -2.84  (-3.73 to -1.93) | <0.001 | |
|  | | Northern Mariana Islands | 1.15  (1.15 to 1.16) | 3.49  (3.47 to 3.50) | 4.74  (1.98 to 7.58) | 0.001 | 0.006  (0.002 to 0.015) | 0.001  (0.000 to 0.003) | -7.18  (-9.01 to -5.3) | <0.001 | |
|  | | Norway | 0.10  (0.10 to 0.10) | 0.21  (0.21 to 0.21) | 1.97  (-4.55 to 8.93) | 0.563 | 0.000  (0.000 to 0.000) | 0.000  (0.000 to 0.001) | 1.46  (-1.21 to 4.21) | 0.287 | |
|  | | Oman | 0.23  (0.23 to 0.23) | 1.27  (1.26 to 1.27) | 5.67  (5.41 to 5.93) | <0.001 | 0.001  (0.000 to 0.003) | 0.001  (0.000 to 0.001) | -2.64  (-3.46 to -1.8) | <0.001 | |
|  | | Pakistan | NA | 3.90  (0.02 to 23.28) | NA | NA | 0.018  (0.007 to 0.041) | 0.017  (0.007 to 0.036) | -0.21  (-0.38 to -0.04) | 0.016 | |
|  | | Palau | 0.09  (0.02 to 0.20) | 2.13  (0.70 to 4.29) | 10.65  (9.66 to 11.64) | <0.001 | 0.002  (0.001 to 0.004) | 0.001  (0.000 to 0.003) | -1.37  (-1.59 to -1.15) | <0.001 | |
|  | | Palestine | 0.02  (0.02 to 0.02) | 0.22  (0.21 to 0.22) | 7.88  (6.61 to 9.17) | <0.001 | 0.002  (0.001 to 0.005) | 0.002  (0.000 to 0.004) | -0.28  (-0.82 to 0.27) | 0.315 | |
|  | | Panama | 4.65  (4.63 to 4.67) | 7.95  (7.91 to 7.99) | 1.77  (1.4 to 2.14) | <0.001 | 0.013  (0.010 to 0.018) | 0.011  (0.007 to 0.015) | -0.65  (-1.56 to 0.26) | 0.162 | |
|  | | Papua New Guinea | 0.04  (0.01 to 0.10) | 9.59  (4.18 to 18.37) | 20.24  (17.78 to 22.76) | <0.001 | 0.005  (0.002 to 0.012) | 0.004  (0.001 to 0.009) | -1.18  (-1.64 to -0.72) | <0.001 | |
|  | | Paraguay | 1.11  (1.11 to 1.11) | 3.17  (3.15 to 3.19) | 3.62  (2.44 to 4.82) | <0.001 | 0.025  (0.015 to 0.039) | 0.021  (0.012 to 0.034) | -0.47  (-0.8 to -0.15) | 0.004 | |
|  | | Peru | 1.06  (1.06 to 1.06) | 2.58  (2.57 to 2.60) | 2.73  (2.01 to 3.45) | <0.001 | 0.010  (0.004 to 0.019) | 0.005  (0.002 to 0.011) | -2.11  (-3.51 to -0.7) | 0.003 | |
|  | | Philippines | 0.02  (0.02 to 0.02) | 3.20  (3.10 to 3.33) | 20.63  (13.44 to 28.27) | <0.001 | 0.002  (0.002 to 0.004) | 0.007  (0.002 to 0.010) | 4.01  (3.65 to 4.36) | <0.001 | |
|  | | Poland | 0.02  (0.02 to 0.02) | 0.19  (0.19 to 0.19) | 11.52  (9.52 to 13.54) | <0.001 | 0.003  (0.003 to 0.003) | 0.001  (0.001 to 0.001) | -4.98  (-5.61 to -4.34) | <0.001 | |
|  | | Portugal | 2.11  (2.11 to 2.12) | 1.34  (1.33 to 1.34) | -1.53  (-2.49 to -0.56) | 0.002 | 0.002  (0.001 to 0.003) | 0.001  (0.000 to 0.001) | -3.62  (-4.36 to -2.88) | <0.001 | |
|  | | Puerto Rico | 13.93  (13.86 to 13.99) | 3.49  (3.48 to 3.50) | -4.1  (-5.19 to -2.98) | <0.001 | 0.007  (0.005 to 0.009) | 0.009  (0.006 to 0.013) | 1.11  (-0.42 to 2.68) | 0.156 | |
|  | | Qatar | 0.41  (0.40 to 0.41) | 0.36  (0.36 to 0.36) | -0.33  (-2.97 to 2.38) | 0.808 | 0.005  (0.002 to 0.013) | 0.002  (0.001 to 0.004) | -3.34  (-4.85 to -1.81) | <0.001 | |
|  | | Republic of Korea | 0.01  (0.01 to 0.01) | 0.03  (0.03 to 0.03) | 2.01  (0.63 to 3.41) | 0.004 | 0.001  (0.001 to 0.001) | 0.000  (0.000 to 0.001) | -1.9  (-2.48 to -1.33) | <0.001 | |
|  | | Republic of Moldova | 0.69  (0.69 to 0.69) | 4.46  (4.45 to 4.47) | 6.16  (5.39 to 6.93) | <0.001 | 0.017  (0.013 to 0.023) | 0.009  (0.006 to 0.012) | -2.53  (-2.98 to -2.09) | <0.001 | |
|  | | Romania | 0.54  (0.54 to 0.54) | 0.95  (0.94 to 0.95) | 2.27  (1.17 to 3.39) | <0.001 | 0.003  (0.002 to 0.004) | 0.001  (0.001 to 0.002) | -2.54  (-3.09 to -1.99) | <0.001 | |
|  | | Russian Federation | 1.22  (1.22 to 1.22) | 13.23  (13.20 to 13.25) | 8  (7.52 to 8.48) | <0.001 | 0.014  (0.013 to 0.016) | 0.011  (0.009 to 0.014) | -0.71  (-1.43 to 0.01) | 0.055 | |
|  | | Rwanda | 56.17  (22.44 to 145.60) | 48.26  (25.52 to 80.35) | -0.37  (-1 to 0.27) | 0.257 | 0.140  (0.048 to 0.341) | 0.041  (0.013 to 0.116) | -3.81  (-4.28 to -3.33) | <0.001 | |
|  | | Saint Kitts and Nevis | 1.82  (0.55 to 4.47) | 14.51  (7.48 to 23.62) | 6.7  (5.94 to 7.47) | <0.001 | 0.035  (0.025 to 0.047) | 0.021  (0.014 to 0.031) | -1.41  (-3.26 to 0.47) | 0.142 | |
|  | | Saint Lucia | 5.16  (5.14 to 5.18) | 3.52  (3.51 to 3.53) | -0.65  (-1.39 to 0.09) | 0.083 | 0.025  (0.019 to 0.034) | 0.035  (0.024 to 0.050) | 1.07  (-0.7 to 2.86) | 0.238 | |
|  | | Saint Vincent and the Grenadines | 16.71  (16.55 to 16.88) | 15.35  (15.21 to 15.49) | 0.28  (-1.25 to 1.83) | 0.722 | 0.006  (0.005 to 0.009) | 0.009  (0.006 to 0.013) | 1.08  (-0.67 to 2.86) | 0.227 | |
|  | | Samoa | 0.21  (0.04 to 0.48) | 7.51  (3.21 to 13.65) | 12.07  (11.23 to 12.93) | <0.001 | 0.002  (0.000 to 0.007) | 0.002  (0.000 to 0.006) | -0.53  (-0.77 to -0.3) | <0.001 | |
|  | | San Marino | 0.24  (0.08 to 0.51) | 0.08  (0.03 to 0.18) | -3.36  (-4.02 to -2.69) | <0.001 | 0.000  (0.000 to 0.001) | 0.000  (0.000 to 0.000) | -2.04  (-2.25 to -1.82) | <0.001 | |
|  | | Sao Tome and Principe | 0.04  (0.01 to 0.08) | 0.04  (0.03 to 0.06) | 0.24  (-0.37 to 0.85) | 0.448 | 0.011  (0.003 to 0.029) | 0.007  (0.003 to 0.019) | -1.15  (-2.05 to -0.25) | 0.013 | |
|  | | Saudi Arabia | 0.83  (0.83 to 0.83) | 2.63  (2.62 to 2.63) | 3.76  (2.78 to 4.75) | <0.001 | 0.001  (0.000 to 0.003) | 0.001  (0.000 to 0.002) | -2.04  (-2.43 to -1.65) | <0.001 | |
|  | | Senegal | 6.83  (3.38 to 12.67) | 13.18  (6.52 to 22.84) | 1.99  (1.15 to 2.83) | <0.001 | 0.014  (0.005 to 0.033) | 0.008  (0.003 to 0.019) | -1.86  (-2.42 to -1.29) | <0.001 | |
|  | | Serbia | 0.18  (0.18 to 0.18) | 0.15  (0.15 to 0.15) | -0.57  (-1.69 to 0.56) | 0.318 | 0.002  (0.001 to 0.003) | 0.001  (0.000 to 0.002) | -2.59  (-2.87 to -2.31) | <0.001 | |
|  | | Seychelles | 0.80  (0.80 to 0.80) | 5.17  (5.13 to 5.22) | 6.49  (1.66 to 11.56) | 0.008 | 0.035  (0.017 to 0.057) | 0.012  (0.007 to 0.020) | -3.39  (-3.94 to -2.85) | <0.001 | |
|  | | Sierra Leone | 5.17  (2.21 to 11.80) | 32.85  (16.56 to 55.06) | 6.05  (4.73 to 7.4) | <0.001 | 0.012  (0.004 to 0.028) | 0.011  (0.003 to 0.025) | -0.17  (-0.61 to 0.26) | 0.429 | |
|  | | Singapore | 0.03  (0.03 to 0.03) | 0.04  (0.04 to 0.04) | 0.65  (-0.38 to 1.69) | 0.219 | 0.001  (0.001 to 0.001) | 0.001  (0.000 to 0.001) | -1.78  (-2.69 to -0.86) | <0.001 | |
|  | | Slovakia | 0.03  (0.03 to 0.03) | 0.03  (0.03 to 0.03) | 0.59  (-0.19 to 1.38) | 0.137 | 0.003  (0.002 to 0.005) | 0.002  (0.001 to 0.003) | -1.82  (-2.65 to -0.97) | <0.001 | |
|  | | Slovenia | 0.05  (0.05 to 0.05) | 0.04  (0.04 to 0.04) | -0.03  (-1.3 to 1.26) | 0.967 | 0.002  (0.002 to 0.003) | 0.001  (0.001 to 0.002) | -3.42  (-4.07 to -2.77) | <0.001 | |
|  | | Solomon Islands | 0.21  (0.04 to 0.49) | 7.69  (3.31 to 14.09) | 12.07  (11.02 to 13.13) | <0.001 | 0.005  (0.001 to 0.013) | 0.005  (0.001 to 0.011) | -0.31  (-0.63 to 0.01) | 0.059 | |
|  | | Somalia | 0.44  (0.04 to 1.53) | 23.47  (11.64 to 42.13) | 12.92  (11.62 to 14.24) | <0.001 | 0.113  (0.033 to 0.336) | 0.093  (0.027 to 0.263) | -0.62  (-0.76 to -0.48) | <0.001 | |
|  | | South Africa | 11.18  (9.27 to 13.49) | 223.94  (208.07 to 240.86) | 9.78  (7.72 to 11.87) | <0.001 | 0.104  (0.047 to 0.151) | 0.026  (0.017 to 0.043) | -4.29  (-5.77 to -2.78) | <0.001 | |
|  | | South Sudan | 9.71  (3.71 to 27.35) | 55.89  (23.26 to 128.48) | 5.57  (4.51 to 6.64) | <0.001 | 0.088  (0.027 to 0.243) | 0.096  (0.029 to 0.276) | 0.26  (-0.62 to 1.16) | 0.56 | |
|  | | Spain | 3.42  (3.41 to 3.43) | 0.54  (0.54 to 0.54) | -5.8  (-7.16 to -4.42) | <0.001 | 0.002  (0.002 to 0.003) | 0.001  (0.000 to 0.001) | -3.46  (-4.02 to -2.89) | <0.001 | |
|  | | Sri Lanka | 0.15  (0.15 to 0.15) | 0.55  (0.55 to 0.55) | 4.41  (3.55 to 5.28) | <0.001 | 0.011  (0.006 to 0.018) | 0.005  (0.003 to 0.009) | -2.3  (-2.74 to -1.87) | <0.001 | |
|  | | Sudan | 2.18  (0.77 to 7.01) | 25.96  (11.79 to 61.55) | 8.25  (7.74 to 8.76) | <0.001 | 0.001  (0.000 to 0.007) | 0.001  (0.000 to 0.005) | -0.54  (-0.81 to -0.27) | <0.001 | |
|  | | Suriname | 16.26  (16.13 to 16.40) | 19.35  (19.13 to 19.56) | 1.16  (0.39 to 1.94) | 0.003 | 0.011  (0.006 to 0.020) | 0.012  (0.005 to 0.027) | 0.46  (-0.67 to 1.6) | 0.425 | |
|  | | Sweden | 0.15  (0.15 to 0.15) | 0.15  (0.15 to 0.15) | 0.25  (-3.17 to 3.8) | 0.886 | 0.001  (0.001 to 0.001) | 0.001  (0.001 to 0.001) | -0.94  (-2.5 to 0.64) | 0.242 | |
|  | | Switzerland | 0.86  (0.86 to 0.86) | 0.37  (0.37 to 0.37) | -2.5  (-3.17 to -1.82) | <0.001 | 0.002  (0.002 to 0.003) | 0.001  (0.001 to 0.001) | -3.1  (-3.44 to -2.76) | <0.001 | |
|  | | Syrian Arab Republic | 0.09  (0.09 to 0.09) | 0.30  (0.30 to 0.30) | 4.07  (2.6 to 5.56) | <0.001 | 0.022  (0.011 to 0.036) | 0.010  (0.004 to 0.024) | -2.64  (-3.32 to -1.95) | <0.001 | |
|  | | Taiwan  (Province of China) | 0.02  (0.02 to 0.02) | 0.15  (0.15 to 0.15) | 7.51  (6.67 to 8.35) | <0.001 | 0.002  (0.002 to 0.003) | 0.001  (0.001 to 0.002) | -1.13  (-2.72 to 0.48) | 0.168 | |
|  | | Tajikistan | 2.44  (2.44 to 2.45) | 2.28  (2.27 to 2.30) | -0.01  (-1.36 to 1.36) | 0.99 | 0.023  (0.012 to 0.039) | 0.010  (0.003 to 0.026) | -2.78  (-2.96 to -2.61) | <0.001 | |
|  | | Thailand | 15.85  (15.50 to 16.23) | 10.97  (10.85 to 11.10) | -0.79  (-2.18 to 0.62) | 0.27 | 0.006  (0.002 to 0.013) | 0.004  (0.002 to 0.009) | -0.68  (-1.13 to -0.22) | 0.004 | |
|  | | Timor-Leste | 0.25  (0.07 to 0.64) | 15.97  (9.20 to 24.68) | 14.12  (13.2 to 15.04) | <0.001 | 0.004  (0.001 to 0.011) | 0.006  (0.002 to 0.014) | 1.57  (0.71 to 2.45) | <0.001 | |
|  | | Togo | 23.30  (10.93 to 45.36) | 41.46  (20.69 to 72.26) | 1.76  (0.84 to 2.69) | <0.001 | 0.017  (0.006 to 0.038) | 0.011  (0.004 to 0.026) | -1.46  (-1.77 to -1.15) | <0.001 | |
|  | | Tokelau | 0.09  (0.02 to 0.21) | 2.20  (0.69 to 4.61) | 10.56  (8.94 to 12.21) | <0.001 | 0.003  (0.001 to 0.009) | 0.002  (0.001 to 0.007) | -0.96  (-1.14 to -0.78) | <0.001 | |
|  | | Tonga | 0.54  (0.54 to 0.54) | 0.95  (0.95 to 0.95) | 1.84  (1.59 to 2.1) | <0.001 | 0.002  (0.001 to 0.005) | 0.002  (0.001 to 0.004) | -1.37  (-1.6 to -1.14) | <0.001 | |
|  | | Trinidad and Tobago | 11.38  (11.32 to 11.43) | 13.73  (13.62 to 13.84) | 1.19  (-0.14 to 2.54) | 0.079 | 0.011  (0.008 to 0.014) | 0.013  (0.008 to 0.019) | 0.77  (-0.8 to 2.36) | 0.341 | |
|  | | Tunisia | 0.01  (0.00 to 0.03) | 0.25  (0.08 to 0.58) | 12.05  (10.09 to 14.03) | <0.001 | 0.000  (0.000 to 0.002) | 0.000  (0.000 to 0.001) | -1.07  (-1.28 to -0.87) | <0.001 | |
|  | | Turkmenistan | 1.75  (1.75 to 1.75) | 2.79  (2.77 to 2.81) | 1.49  (-0.13 to 3.13) | 0.072 | 0.020  (0.015 to 0.025) | 0.008  (0.006 to 0.012) | -2.76  (-4.11 to -1.38) | <0.001 | |
|  | | Tuvalu | 0.09  (0.02 to 0.21) | 2.37  (0.74 to 5.06) | 10.72  (9.12 to 12.36) | <0.001 | 0.004  (0.001 to 0.012) | 0.002  (0.001 to 0.006) | -2.04  (-2.16 to -1.93) | <0.001 | |
|  | | Türkiye | 0.03  (0.03 to 0.03) | 0.21  (0.21 to 0.21) | 6.91  (6.03 to 7.8) | <0.001 | 0.001  (0.000 to 0.002) | 0.000  (0.000 yo 0.001) | -0.84  (-1.24 to -0.44) | <0.001 | |
|  | | Uganda | 707.37  (369.59 to 1137.73) | 115.02  (58.60 to 195.20) | -5.76  (-6.59 to -4.92) | <0.001 | 0.049  (0.015 to 0.126) | 0.062  (0.022 to 0.138) | 0.75  (0.16 to 1.34) | 0.013 | |
|  | | Ukraine | 1.56  (1.56 to 1.56) | 13.11  (13.01 to 13.21) | 7.23  (6.26 to 8.21) | <0.001 | 0.006  (0.004 to 0.008) | 0.007  (0.004 to 0.012) | 0.68  (-0.23 to 1.6) | 0.143 | |
|  | | United Arab Emirates | 0.08  (0.03 to 0.18) | 0.21  (0.08 to 0.42) | 2.79  (1.7 to 3.9) | <0.001 | 0.010  (0.003 to 0.030) | 0.005  (0.002 to 0.011) | -2.26  (-3.37 to -1.14) | <0.001 | |
|  | | United Kingdom | 0.16  (0.16 to 0.16) | 0.28  (0.28 to 0.28) | 1.74  (-0.94 to 4.49) | 0.205 | 0.002  (0.002 to 0.002) | 0.002  (0.002 to 0.002) | -1.12  (-1.99 to -0.25) | 0.012 | |
|  | | United Republic of Tanzania | 184.58  (91.64 to 333.43) | 93.94  (48.87 to 157.57) | -2.43  (-3.48 to -1.37) | <0.001 | 0.104  (0.034 to 0.236) | 0.047  (0.017 to 0.115) | -2.55  (-2.86 to -2.24) | <0.001 | |
|  | | United States of America | 3.81  (3.80 to 3.81) | 0.81  (0.81 to 0.81) | -4.9  (-7.04 to -2.72) | <0.001 | 0.003  (0.003 to 0.003) | 0.002  (0.002 to 0.002) | -1.64  (-2.2 to -1.06) | <0.001 | |
|  | | United States Virgin Islands | 5.49  (5.47 to 5.51) | 4.34  (4.33 to 4.36) | -0.72  (-1.42 to -0.02) | 0.045 | 0.014  (0.005 to 0.030) | 0.012  (0.004 to 0.032) | -0.12  (-0.93 to 0.69) | 0.766 | |
|  | | Uruguay | 1.17  (1.17 to 1.17) | 3.89  (3.87 to 3.90) | 4.24  (3.83 to 4.65) | <0.001 | 0.007  (0.005 to 0.010) | 0.007  (0.005 to 0.009) | -0.32  (-0.95 to 0.3) | 0.312 | |
|  | | Uzbekistan | 0.93  (0.93 to 0.93) | 1.89  (1.89 to 1.90) | 2.32  (1.71 to 2.94) | <0.001 | 0.013  (0.009 to 0.017) | 0.003  (0.002 to 0.004) | -4.75  (-5.66 to -3.82) | <0.001 | |
|  | | Vanuatu | 0.21  (0.04 to 0.47) | 7.60  (3.27 to 14.05) | 12.17  (11.16 to 13.19) | <0.001 | 0.003  (0.001 to 0.010) | 0.003  (0.001 to 0.008) | -0.59  (-1.04 to -0.13) | 0.011 | |
|  | | Venezuela  (Bolivarian Republic of) | 1.77  (1.77 to 1.78) | 4.54  (4.52 to 4.56) | 3.24  (2.74 to 3.74) | <0.001 | 0.012  (0.009 to 0.016) | 0.015  (0.009 to 0.022) | 0.7  (-0.04 to 1.44) | 0.065 | |
|  | | Viet Nam | 0.21  (0.07 to 0.41) | 2.52  (1.51 to 4.30) | 8.68  (7.36 to 10.01) | <0.001 | 0.001  (0.000 to 0.003) | 0.001  (0.000 to 0.002) | -2.18  (-2.28 to -2.08) | <0.001 | |
|  | | Yemen | 0.11  (0.02 to 0.29) | 1.21  (0.39 to 2.44) | 8.22  (7.66 to 8.78) | <0.001 | 0.000  (0.000 to 0.002) | 0.000  (0.000 to 0.002) | 0.65  (-0.27 to 1.57) | 0.167 | |
|  | | Zambia | 287.27  (139.52 to 515.51) | 197.14  (98.69 to 349.99) | -1.42  (-2.42 to -0.42) | 0.006 | 0.132  (0.038 to 0.293) | 0.081  (0.025 to 0.227) | -1.55  (-1.75 to -1.35) | <0.001 | |
|  | | Zimbabwe | 327.05  (155.66 to 584.54) | 205.07  (106.24 to 347.37) | -1.62  (-2.12 to -1.12) | <0.001 | 0.024  (0.006 to 0.084) | 0.034  (0.010 to 0.100) | 1.13  (0.29 to 1.98) | 0.009 | |
| Age-standardized DALYs | | Afghanistan | 9.75  (1.71 to 21.30) | 27.51  (7.49 to 59.47) | 3.39  (2.82 to 3.97) | <0.001 | 0.99  (0.63 to 1.72) | 0.70  (0.44 to 1.23) | -1.13  (-1.2 to -1.07) | <0.001 | |
|  | | Albania | 1.58  (1.49 to 1.68) | 2.02  (1.90 to 2.29) | 0.91  (0.33 to 1.5) | 0.002 | 0.24  (0.13 to 0.42) | 0.12  (0.06 to 0.24) | -2.34  (-2.76 to -1.91) | <0.001 | |
|  | | Algeria | 7.23  (3.77 to 11.96) | 37.01  (19.41 to 67.90) | 5.35  (4.85 to 5.85) | <0.001 | 0.20  (0.09 to 0.45) | 0.14  (0.04 to 0.36) | -1.03  (-1.62 to -0.43) | 0.001 | |
|  | | American Samoa | 49.08  (48.83 to 49.33) | 191.70  (186.66 to 197.85) | 4.02  (1.83 to 6.26) | <0.001 | 0.89  (0.40 to 1.85) | 3.13  (0.62 to 6.93) | 4.17  (3.73 to 4.6) | <0.001 | |
|  | | Andorra | 6.48  (3.11 to 12.65) | 3.47  (1.76 to 6.38) | -2.02  (-2.54 to -1.5) | <0.001 | 0.31  (0.12 to 0.69) | 0.24  (0.08 to 0.55) | -0.83  (-1 to -0.66) | <0.001 | |
|  | | Angola | 276.80  (127.28 to 554.36) | 7578.15  (4493.49 to 12280.66) | 11.01  (9.83 to 12.2) | <0.001 | 13.39  (7.10 to 21.44) | 6.74  (2.88 to 11.85) | -2.17  (-2.37 to -1.97) | <0.001 | |
|  | | Antigua and Barbuda | 465.64  (460.47 to 472.81) | 311.62  (296.10 to 339.53) | -1.2  (-2.19 to -0.2) | 0.019 | 0.57  (0.37 to 0.97) | 0.81  (0.56 to 1.22) | 1.12  (-0.19 to 2.45) | 0.095 | |
|  | | Argentina | 78.10  (73.19 to 85.17) | 173.18  (165.81 to 183.27) | 2.94  (2.08 to 3.8) | <0.001 | 0.69  (0.40 to 1.26) | 0.74  (0.44 to 1.33) | 0.35  (-0.34 to 1.05) | 0.317 | |
|  | | Armenia | 6.24  (6.18 to 6.30) | 19.94  (17.76 to 23.22) | 4.06  (2.98 to 5.15) | <0.001 | 0.90  (0.73 to 1.10) | 0.13  (0.09 to 0.23) | -6.02  (-7.85 to -4.15) | <0.001 | |
|  | | Australia | 8.79  (8.12 to 9.70) | 8.11  (6.12 to 11.69) | -1.3  (-2.67 to 0.1) | 0.069 | 0.22  (0.14 to 0.39) | 0.12  (0.05 to 0.26) | -1.99  (-2.65 to -1.33) | <0.001 | |
|  | | Austria | 28.84  (28.13 to 29.86) | 20.84  (15.94 to 29.27) | -1.31  (-2.27 to -0.35) | 0.008 | 0.15  (0.11 to 0.22) | 0.10  (0.06 to 0.15) | -1.44  (-1.62 to -1.25) | <0.001 | |
|  | | Azerbaijan | 16.91  (16.63 to 17.27) | 15.92  (14.67 to 17.92) | -0.36  (-0.73 to 0) | 0.053 | 1.60  (1.08 to 2.33) | 0.78  (0.43 to 1.48) | -2.3  (-3.02 to -1.57) | <0.001 | |
|  | | Bahamas | 1424.40  (1381.27 to 1480.89) | 1708.50  (1631.48 to 1821.39) | 0.44  (-0.69 to 1.58) | 0.449 | 1.02  (0.66 to 1.65) | 1.81  (1.23 to 2.69) | 1.9  (0.16 to 3.66) | 0.032 | |
|  | | Bahrain | 15.81  (15.56 to 16.26) | 66.64  (65.98 to 67.83) | 4.75  (1.75 to 7.84) | 0.002 | 0.22  (0.07 to 0.57) | 0.24  (0.08 to 0.58) | 0.13  (-0.14 to 0.4) | 0.341 | |
|  | | Bangladesh | 0.05  (0.02 to 0.10) | 20.41  (10.42 to 35.85) | 21.26  (20.8 to 21.72) | <0.001 | 4.54  (1.89 to 8.27) | 1.53  (0.96 to 2.46) | -3.49  (-3.62 to -3.35) | <0.001 | |
|  | | Barbados | 413.49  (405.60 to 426.36) | 420.19  (402.20 to 445.76) | 0.27  (-0.55 to 1.11) | 0.516 | 1.01  (0.76 to 1.36) | 1.04  (0.72 to 1.47) | 0.25  (-0.48 to 0.97) | 0.505 | |
|  | | Belarus | 42.82  (41.52 to 43.55) | 259.55  (248.00 to 279.57) | 6.3  (5.04 to 7.58) | <0.001 | 0.53  (0.40 to 0.70) | 0.39  (0.27 to 0.54) | -1.07  (-2.2 to 0.07) | 0.065 | |
|  | | Belgium | 49.10  (48.03 to 50.64) | 33.15  (26.75 to 42.08) | -1.37  (-2.34 to -0.38) | 0.007 | 0.13  (0.09 to 0.20) | 0.08  (0.05 to 0.13) | -1.91  (-2.14 to -1.69) | <0.001 | |
|  | | Belize | 752.29  (741.74 to 765.54) | 1161.56  (1112.98 to 1250.02) | 1.94  (0.98 to 2.9) | <0.001 | 0.48  (0.31 to 0.77) | 0.83  (0.58 to 1.18) | 1.91  (0.93 to 2.89) | <0.001 | |
|  | | Benin | 73.44  (24.02 to 178.03) | 1300.36  (701.08 to 2127.20) | 8.36  (5.37 to 11.43) | <0.001 | 3.28  (2.21 to 4.82) | 2.15  (1.44 to 3.28) | -1.38  (-1.64 to -1.13) | <0.001 | |
|  | | Bermuda | 483.88  (478.39 to 491.21) | 279.17  (273.81 to 287.64) | -1.26  (-2.57 to 0.06) | 0.062 | 0.47  (0.26 to 0.90) | 0.49  (0.29 to 0.86) | 0.17  (-0.35 to 0.69) | 0.522 | |
|  | | Bhutan | 13.64  (3.38 to 34.53) | 67.89  (27.12 to 146.76) | 5.07  (3.62 to 6.54) | <0.001 | 8.33  (3.87 to 14.47) | 4.05  (2.53 to 6.56) | -2.36  (-2.77 to -1.94) | <0.001 | |
|  | | Bolivia  (Plurinational State of) | 6.09  (0.64 to 25.42) | 154.60  (63.07 to 306.78) | 10.75  (9.99 to 11.51) | <0.001 | 4.64  (1.76 to 8.87) | 2.00  (1.34 to 3.09) | -2.69  (-2.89 to -2.49) | <0.001 | |
|  | | Bosnia and Herzegovina | 6.46  (6.43 to 6.50) | 9.63  (9.58 to 9.69) | 1.41  (0.94 to 1.9) | <0.001 | 0.10  (0.07 to 0.17) | 0.08  (0.05 to 0.15) | -0.7  (-1.12 to -0.29) | 0.001 | |
|  | | Botswana | 9614.75  (5006.32 to 17483.96) | 16696.04  (8958.67 to 28835.31) | 1.7  (0.77 to 2.64) | <0.001 | 11.10  (5.86 to 18.04) | 4.56  (2.48 to 7.85) | -2.83  (-3.19 to -2.47) | <0.001 | |
|  | | Brazil | 189.90  (184.90 to 196.73) | 280.72  (265.51 to 302.80) | 1.09  (0.61 to 1.56) | <0.001 | 1.92  (1.54 to 2.55) | 1.95  (1.47 to 2.75) | 0.1  (-0.08 to 0.28) | 0.293 | |
|  | | Brunei Darussalam | 6.56  (6.05 to 7.50) | 21.92  (18.36 to 27.52) | 3.96  (3.36 to 4.58) | <0.001 | 0.33  (0.18 to 0.58) | 0.38  (0.23 to 0.61) | 0.46  (0.35 to 0.58) | <0.001 | |
|  | | Bulgaria | 20.96  (20.73 to 21.21) | 30.41  (28.55 to 33.18) | 1.48  (0.4 to 2.58) | 0.007 | 0.13  (0.10 to 0.17) | 0.10  (0.07 to 0.14) | -0.94  (-1.61 to -0.26) | 0.007 | |
|  | | Burkina Faso | 11456.97  (6209.18 to 19294.09) | 853.28  (463.62 to 1384.82) | -8.13  (-8.75 to -7.5) | <0.001 | 3.30  (2.18 to 5.12) | 2.63  (1.76 to 3.98) | -0.72  (-0.81 to -0.62) | <0.001 | |
|  | | Burundi | 9026.47  (4068.69 to 20000.70) | 1286.40  (677.42 to 2221.46) | -6.3  (-7.69 to -4.9) | <0.001 | 7.56  (3.01 to 17.05) | 4.38  (2.25 to 10.80) | -1.77  (-2.03 to -1.51) | <0.001 | |
|  | | Cabo Verde | 728.69  (240.98 to 1708.91) | 544.45  (163.23 to 1618.04) | -1.1  (-1.69 to -0.51) | <0.001 | 1.13  (0.67 to 1.89) | 0.79  (0.48 to 1.34) | -1.15  (-1.37 to -0.93) | <0.001 | |
|  | | Cambodia | 0.17  (0.09 to 0.29) | 374.59  (154.78 to 709.11) | 23.67  (18.61 to 28.94) | <0.001 | 0.67  (0.41 to 1.22) | 0.46  (0.25 to 0.97) | -1.21  (-1.37 to -1.05) | <0.001 | |
|  | | Cameroon | 1599.19  (819.11 to 2867.76) | 5917.82  (3732.52 to 8892.68) | 4.33  (3.48 to 5.18) | <0.001 | 11.09  (5.41 to 18.69) | 4.64  (1.67 to 8.83) | -2.78  (-2.95 to -2.61) | <0.001 | |
|  | | Canada | 32.79  (30.27 to 36.92) | 26.56  (18.45 to 39.52) | -1.06  (-1.72 to -0.39) | 0.002 | 0.19  (0.14 to 0.25) | 0.14  (0.10 to 0.19) | -1.06  (-1.28 to -0.83) | <0.001 | |
|  | | Central African Republic | 8862.21  (3791.69 to 17192.84) | 8607.89  (4880.12 to 14495.53) | -0.36  (-1.63 to 0.93) | 0.584 | 16.29  (9.10 to 26.15) | 16.57  (9.43 to 26.30) | 0.05  (-0.23 to 0.33) | 0.748 | |
|  | | Chad | 1431.84  (699.37 to 2796.28) | 2007.10  (1058.99 to 3574.83) | 0.94  (-0.24 to 2.13) | 0.119 | 8.29  (3.77 to 14.63) | 6.30  (2.50 to 11.64) | -0.88  (-1.05 to -0.71) | <0.001 | |
|  | | Chile | 29.01  (28.10 to 30.19) | 80.74  (72.41 to 96.45) | 3.76  (3.26 to 4.27) | <0.001 | 0.45  (0.29 to 0.73) | 0.38  (0.23 to 0.64) | -0.44  (-0.87 to -0.02) | 0.042 | |
|  | | China | 5.42  (0.68 to 9.62) | 53.06  (39.13 to 67.96) | 7.57  (6.7 to 8.46) | <0.001 | 0.62  (0.41 to 0.89) | 0.25  (0.15 to 0.43) | -2.99  (-3.2 to -2.79) | <0.001 | |
|  | | Colombia | 65.41  (64.59 to 67.30) | 198.52  (189.51 to 214.09) | 3.56  (1.99 to 5.16) | <0.001 | 1.36  (1.04 to 1.81) | 1.11  (0.78 to 1.54) | -0.58  (-0.93 to -0.22) | 0.001 | |
|  | | Comoros | 2.56  (0.24 to 9.34) | 12.27  (5.27 to 23.10) | 4.98  (3.73 to 6.24) | <0.001 | 13.91  (6.59 to 25.64) | 7.10  (3.02 to 14.03) | -2.33  (-3.75 to -0.89) | 0.002 | |
|  | | Congo | 8569.95  (4018.55 to 15647.61) | 7597.78  (4421.35 to 12569.98) | -0.42  (-1.54 to 0.72) | 0.471 | 8.28  (3.81 to 14.61) | 5.09  (2.02 to 9.20) | -1.57  (-1.87 to -1.26) | <0.001 | |
|  | | Cook Islands | 5.50  (1.26 to 12.07) | 156.53  (59.87 to 294.59) | 11.29  (10.58 to 12) | <0.001 | 0.84  (0.32 to 1.93) | 1.01  (0.38 to 2.20) | 0.59  (0.51 to 0.67) | <0.001 | |
|  | | Costa Rica | 84.12  (83.03 to 86.46) | 143.90  (139.71 to 150.70) | 1.77  (1.34 to 2.21) | <0.001 | 0.80  (0.57 to 1.12) | 0.91  (0.66 to 1.24) | 0.41  (-0.13 to 0.95) | 0.141 | |
|  | | Coted'Ivoire | 13415.18  (6631.76 to 25020.78) | 3783.17  (2163.93 to 6058.83) | -4.34  (-5.18 to -3.5) | <0.001 | 5.57  (2.15 to 10.34) | 3.44  (2.29 to 5.15) | -1.53  (-1.77 to -1.28) | <0.001 | |
|  | | Croatia | 6.13  (6.03 to 6.24) | 6.42  (6.01 to 7.09) | 0.4  (-0.32 to 1.13) | 0.272 | 0.11  (0.08 to 0.17) | 0.07  (0.04 to 0.12) | -1.6  (-1.86 to -1.35) | <0.001 | |
|  | | Cuba | 45.51  (45.02 to 46.44) | 61.06  (54.43 to 75.08) | 1.87  (-0.46 to 4.26) | 0.116 | 1.09  (0.79 to 1.51) | 1.99  (1.41 to 2.76) | 2.21  (1.13 to 3.3) | <0.001 | |
|  | | Cyprus | 10.03  (9.95 to 10.14) | 17.94  (17.11 to 19.31) | 2  (1.49 to 2.52) | <0.001 | 0.15  (0.07 to 0.30) | 0.09  (0.04 to 0.16) | -1.63  (-2.06 to -1.2) | <0.001 | |
|  | | Czechia | 3.53  (3.45 to 3.65) | 4.78  (4.03 to 5.94) | 1.34  (0.03 to 2.68) | 0.045 | 0.22  (0.16 to 0.29) | 0.13  (0.09 to 0.21) | -1.49  (-2.17 to -0.79) | <0.001 | |
|  | | Democratic People's Republic of Korea | 0.97  (0.04 to 2.80) | 77.25  (15.06 to 276.73) | 14.67  (13.18 to 16.18) | <0.001 | 0.28  (0.15 to 0.53) | 0.34  (0.19 to 0.59) | 0.6  (0.52 to 0.67) | <0.001 | |
|  | | Democratic Republic of the Congo | 5458.55  (3154.92 to 9055.67) | 928.68  (523.32 to 1579.17) | -5.9  (-6.73 to -5.06) | <0.001 | 13.04  (7.07 to 21.27) | 7.06  (3.17 to 12.61) | -2  (-2.24 to -1.75) | <0.001 | |
|  | | Denmark | 24.80  (24.15 to 25.63) | 12.45  (11.53 to 13.76) | -2.41  (-2.84 to -1.97) | <0.001 | 0.22  (0.16 to 0.30) | 0.11  (0.08 to 0.15) | -2.17  (-2.62 to -1.73) | <0.001 | |
|  | | Djibouti | 17.93  (1.73 to 60.47) | 5902.22  (3328.34 to 9820.49) | 20.95  (19.34 to 22.58) | <0.001 | 7.31  (3.06 to 14.57) | 6.12  (2.47 to 12.71) | -0.57  (-0.84 to -0.29) | <0.001 | |
|  | | Dominica | 309.29  (305.24 to 314.68) | 316.36  (307.24 to 335.37) | 0.13  (-0.66 to 0.92) | 0.749 | 0.85  (0.42 to 1.64) | 2.92  (0.59 to 6.62) | 4.29  (4.06 to 4.53) | <0.001 | |
|  | | Dominican Republic | 356.29  (124.45 to 839.08) | 388.40  (122.32 to 840.76) | 0.19  (-0.66 to 1.05) | 0.667 | 3.62  (2.10 to 5.91) | 3.48  (1.77 to 6.52) | -0.17  (-0.49 to 0.16) | 0.315 | |
|  | | Ecuador | 50.53  (48.97 to 52.98) | 252.22  (240.27 to 270.95) | 5.12  (4.02 to 6.24) | <0.001 | 1.36  (0.93 to 2.14) | 0.85  (0.51 to 1.53) | -1.46  (-2.01 to -0.9) | <0.001 | |
|  | | Egypt | 11.13  (11.03 to 11.25) | 29.57  (28.78 to 31.02) | 3.78  (2.94 to 4.63) | <0.001 | 0.54  (0.30 to 1.01) | 0.26  (0.14 to 0.47) | -2.28  (-2.62 to -1.94) | <0.001 | |
|  | | El Salvador | 127.29  (125.82 to 129.83) | 247.01  (236.09 to 264.81) | 2.01  (0.89 to 3.15) | <0.001 | 0.55  (0.39 to 0.76) | 0.40  (0.25 to 0.60) | -1.2  (-1.44 to -0.95) | <0.001 | |
|  | | Equatorial Guinea | 706.65  (326.16 to 1538.38) | 15307.30  (7472.35 to 29401.22) | 10.43  (9.14 to 11.72) | <0.001 | 18.00  (10.24 to 28.34) | 5.56  (2.35 to 10.00) | -3.7  (-3.96 to -3.44) | <0.001 | |
|  | | Eritrea | 1801.76  (775.95 to 3819.53) | 1300.18  (780.01 to 2052.60) | -1.16  (-1.8 to -0.51) | <0.001 | 9.44  (4.07 to 20.92) | 5.90  (3.07 to 12.44) | -1.57  (-1.75 to -1.38) | <0.001 | |
|  | | Estonia | 26.78  (26.62 to 26.94) | 186.41  (173.22 to 207.12) | 7.63  (7.23 to 8.02) | <0.001 | 0.91  (0.68 to 1.20) | 0.42  (0.29 to 0.63) | -2.62  (-3.44 to -1.79) | <0.001 | |
|  | | Eswatini | 337.83  (107.66 to 912.54) | 30206.90  (14935.16 to 56264.30) | 15.27  (13.58 to 16.98) | <0.001 | 9.83  (4.96 to 16.24) | 5.46  (2.35 to 9.84) | -1.9  (-2.06 to -1.74) | <0.001 | |
|  | | Ethiopia | 2101.08  (1106.70 to 3855.71) | 1545.61  (894.03 to 2449.24) | -0.89  (-2.01 to 0.24) | 0.124 | 25.22  (15.01 to 39.25) | 7.71  (4.14 to 16.60) | -3.75  (-3.87 to -3.62) | <0.001 | |
|  | | Fiji | 137.54  (136.47 to 138.70) | 242.77  (237.75 to 250.21) | 1.96  (0.39 to 3.56) | 0.014 | 3.78  (2.29 to 6.52) | 3.79  (1.12 to 7.71) | 0.02  (-0.14 to 0.19) | 0.781 | |
|  | | Finland | 11.53  (11.27 to 11.84) | 8.48  (7.14 to 10.46) | -0.93  (-1.36 to -0.5) | <0.001 | 0.36  (0.26 to 0.47) | 0.17  (0.13 to 0.25) | -2.21  (-2.6 to -1.81) | <0.001 | |
|  | | France | 142.06  (137.50 to 148.49) | 35.54  (30.05 to 42.33) | -5.03  (-6.02 to -4.04) | <0.001 | 0.15  (0.10 to 0.25) | 0.09  (0.05 to 0.18) | -1.62  (-1.86 to -1.38) | <0.001 | |
|  | | Gabon | 2182.57  (1134.12 to 3994.95) | 6178.76  (3335.93 to 10303.75) | 3.31  (2.06 to 4.58) | <0.001 | 7.78  (3.70 to 13.60) | 3.79  (2.45 to 6.10) | -2.36  (-2.66 to -2.06) | <0.001 | |
|  | | Gambia | 267.10  (124.25 to 523.29) | 4610.48  (2328.76 to 8164.93) | 9.43  (8.31 to 10.57) | <0.001 | 4.61  (1.62 to 8.81) | 4.03  (1.36 to 7.91) | -0.49  (-0.75 to -0.22) | <0.001 | |
|  | | Georgia | 10.74  (10.64 to 10.95) | 38.84  (36.26 to 44.02) | 4.3  (4.04 to 4.56) | <0.001 | 0.46  (0.33 to 0.66) | 0.20  (0.11 to 0.38) | -2.69  (-3.48 to -1.89) | <0.001 | |
|  | | Germany | 47.73  (46.20 to 49.86) | 19.62  (15.89 to 25.45) | -3.4  (-4.23 to -2.56) | <0.001 | 0.12  (0.08 to 0.17) | 0.06  (0.04 to 0.11) | -2.11  (-2.43 to -1.79) | <0.001 | |
|  | | Ghana | 2068.74  (1109.12 to 3535.46) | 4460.80  (2722.67 to 6916.20) | 2.6  (1.81 to 3.41) | <0.001 | 4.34  (1.59 to 8.18) | 3.14  (2.08 to 4.75) | -1.05  (-1.33 to -0.76) | <0.001 | |
|  | | Greece | 11.18  (10.85 to 11.58) | 10.18  (8.17 to 13.20) | -0.45  (-0.92 to 0.02) | 0.062 | 0.10  (0.07 to 0.15) | 0.10  (0.07 to 0.17) | 0.15  (-0.14 to 0.45) | 0.31 | |
|  | | Greenland | 130.91  (123.67 to 141.06) | 107.44  (93.52 to 129.94) | -0.26  (-2.18 to 1.7) | 0.793 | 0.17  (0.05 to 0.43) | 0.28  (0.11 to 0.59) | 1.58  (1.47 to 1.7) | <0.001 | |
|  | | Grenada | 331.66  (328.34 to 336.07) | 246.49  (242.66 to 252.15) | -0.46  (-1.48 to 0.57) | 0.379 | 1.53  (1.02 to 2.41) | 2.17  (1.46 to 3.21) | 1.35  (0.43 to 2.27) | 0.004 | |
|  | | Guam | 52.28  (52.09 to 52.51) | 318.96  (315.91 to 323.48) | 6.7  (4.09 to 9.37) | <0.001 | 0.67  (0.25 to 1.54) | 0.95  (0.49 to 1.91) | 1.15  (0.8 to 1.5) | <0.001 | |
|  | | Guatemala | 284.10  (278.42 to 292.85) | 197.32  (190.30 to 209.26) | -1.81  (-2.44 to -1.17) | <0.001 | 1.76  (1.40 to 2.25) | 1.24  (0.92 to 1.66) | -0.99  (-1.4 to -0.57) | <0.001 | |
|  | | Guinea | 667.79  (336.43 to 1298.37) | 2398.15  (1398.28 to 3813.38) | 4.08  (3.15 to 5.02) | <0.001 | 7.03  (2.86 to 12.58) | 5.11  (1.85 to 9.56) | -1.03  (-1.23 to -0.82) | <0.001 | |
|  | | Guinea-Bissau | 493.84  (209.88 to 950.59) | 5521.21  (2275.68 to 11019.48) | 7.98  (7.08 to 8.89) | <0.001 | 8.01  (3.18 to 15.08) | 4.70  (1.60 to 9.13) | -1.65  (-1.84 to -1.46) | <0.001 | |
|  | | Guyana | 1042.08  (1027.16 to 1061.71) | 1704.53  (1646.37 to 1787.23) | 1.74  (1.02 to 2.46) | <0.001 | 1.77  (1.24 to 2.43) | 3.02  (1.91 to 4.62) | 1.95  (0.11 to 3.81) | 0.037 | |
|  | | Haiti | 8774.22  (4225.46 to 15845.34) | 2754.89  (1406.44 to 4552.93) | -3.66  (-4.89 to -2.41) | <0.001 | 10.82  (5.49 to 18.33) | 7.85  (3.42 to 13.87) | -0.96  (-1.28 to -0.64) | <0.001 | |
|  | | Honduras | 439.52  (433.63 to 448.12) | 141.44  (139.08 to 144.90) | -3.56  (-3.65 to -3.47) | <0.001 | 3.57  (1.72 to 7.08) | 1.80  (0.78 to 3.83) | -2.28  (-2.61 to -1.95) | <0.001 | |
|  | | Hungary | 27.86  (27.28 to 28.87) | 19.82  (19.62 to 20.11) | -0.66  (-1.5 to 0.19) | 0.13 | 0.66  (0.49 to 0.88) | 0.34  (0.23 to 0.53) | -2.19  (-3.05 to -1.33) | <0.001 | |
|  | | Iceland | 23.35  (22.34 to 24.88) | 20.81  (16.49 to 27.40) | -0.56  (-1.03 to -0.08) | 0.023 | 0.11  (0.08 to 0.18) | 0.07  (0.04 to 0.12) | -1.51  (-1.84 to -1.19) | <0.001 | |
|  | | India | 2.27  (1.04 to 4.45) | 208.86  (139.90 to 291.24) | 15.74  (14.03 to 17.48) | <0.001 | 6.04  (3.95 to 8.05) | 2.97  (1.99 to 4.04) | -2.27  (-2.71 to -1.83) | <0.001 | |
|  | | Indonesia | 12.69  (12.61 to 12.79) | 63.85  (60.21 to 69.41) | 5.4  (4.97 to 5.83) | <0.001 | 2.36  (1.39 to 3.65) | 2.47  (1.52 to 3.70) | 0.16  (0.08 to 0.24) | <0.001 | |
|  | | Iran  (Islamic Republic of) | 2.29  (2.22 to 2.42) | 23.36  (22.22 to 24.78) | 7.54  (5.51 to 9.61) | <0.001 | 0.14  (0.08 to 0.27) | 0.13  (0.06 to 0.26) | -0.22  (-0.41 to -0.04) | 0.016 | |
|  | | Iraq | 3.87  (3.77 to 4.09) | 25.23  (24.05 to 28.40) | 6.16  (5.17 to 7.17) | <0.001 | 0.33  (0.16 to 0.67) | 0.21  (0.09 to 0.42) | -1.57  (-1.86 to -1.28) | <0.001 | |
|  | | Ireland | 17.15  (16.61 to 17.85) | 15.25  (11.40 to 20.84) | -0.5  (-0.92 to -0.07) | 0.021 | 0.14  (0.10 to 0.20) | 0.07  (0.04 to 0.13) | -1.92  (-2.41 to -1.43) | <0.001 | |
|  | | Israel | 33.04  (32.32 to 34.22) | 24.83  (20.09 to 31.21) | -1.13  (-1.75 to -0.49) | <0.001 | 0.09  (0.05 to 0.14) | 0.06  (0.03 to 0.11) | -1.34  (-1.54 to -1.14) | <0.001 | |
|  | | Italy | 135.38  (130.37 to 142.23) | 33.64  (29.24 to 39.85) | -4.89  (-5.94 to -3.83) | <0.001 | 0.12  (0.08 to 0.19) | 0.09  (0.06 to 0.15) | -0.99  (-1.29 to -0.69) | <0.001 | |
|  | | Jamaica | 640.62  (631.15 to 654.32) | 912.65  (880.21 to 962.22) | 1.42  (0.4 to 2.44) | 0.006 | 1.46  (0.79 to 2.74) | 1.70  (1.10 to 2.61) | 0.64  (-0.71 to 2.01) | 0.354 | |
|  | | Japan | 0.71  (0.61 to 0.89) | 2.80  (2.13 to 3.73) | 4.55  (4.01 to 5.1) | <0.001 | 0.16  (0.08 to 0.33) | 0.16  (0.09 to 0.31) | 0.02  (-0.15 to 0.18) | 0.828 | |
|  | | Jordan | 4.01  (3.95 to 4.11) | 20.67  (20.56 to 20.83) | 5.5  (4.76 to 6.24) | <0.001 | 0.18  (0.04 to 0.46) | 0.16  (0.04 to 0.43) | -0.36  (-0.46 to -0.26) | <0.001 | |
|  | | Kazakhstan | 27.31  (26.96 to 27.75) | 79.04  (73.18 to 88.98) | 3.94  (3.48 to 4.4) | <0.001 | 0.90  (0.69 to 1.15) | 0.29  (0.22 to 0.39) | -3.77  (-5.06 to -2.47) | <0.001 | |
|  | | Kenya | 9904.90  (5489.95 to 16614.62) | 5214.57  (3295.66 to 7673.35) | -1.97  (-2.96 to -0.97) | <0.001 | 8.45  (5.61 to 12.31) | 5.93  (3.91 to 9.09) | -1.12  (-1.23 to -1.01) | <0.001 | |
|  | | Kiribati | 152.40  (151.32 to 153.67) | 188.73  (186.93 to 191.21) | 0.37  (-1.1 to 1.87) | 0.621 | 7.02  (2.61 to 13.10) | 7.60  (3.04 to 13.85) | 0.26  (0.08 to 0.43) | 0.005 | |
|  | | Kuwait | 6.88  (6.84 to 6.94) | 5.62  (5.54 to 5.75) | 0.49  (-1.97 to 3.01) | 0.698 | 0.11  (0.02 to 0.30) | 0.24  (0.13 to 0.44) | 2.52  (1.64 to 3.4) | <0.001 | |
|  | | Kyrgyzstan | 58.70  (58.27 to 59.21) | 167.06  (159.05 to 178.98) | 3.77  (2.77 to 4.77) | <0.001 | 1.08  (0.80 to 1.41) | 0.23  (0.17 to 0.31) | -4.91  (-6.06 to -3.75) | <0.001 | |
|  | | Lao People's Democratic Republic | 0.37  (0.15 to 0.75) | 109.12  (48.09 to 217.02) | 20.06  (18.56 to 21.58) | <0.001 | 1.30  (0.87 to 2.00) | 0.88  (0.55 to 1.51) | -1.24  (-1.34 to -1.13) | <0.001 | |
|  | | Latvia | 93.19  (91.81 to 94.55) | 650.52  (634.56 to 672.34) | 6.51  (5.75 to 7.28) | <0.001 | 1.17  (0.87 to 1.53) | 0.56  (0.37 to 0.89) | -2.34  (-2.89 to -1.79) | <0.001 | |
|  | | Lebanon | 11.86  (6.28 to 19.64) | 68.77  (40.01 to 109.38) | 5.84  (5.69 to 5.98) | <0.001 | 0.39  (0.17 to 0.80) | 0.22  (0.09 to 0.46) | -1.9  (-2.14 to -1.65) | <0.001 | |
|  | | Lesotho | 3886.86  (2047.64 to 6715.24) | 32047.85  (18615.97 to 50424.95) | 6.71  (4.52 to 8.95) | <0.001 | 8.11  (3.68 to 14.18) | 7.92  (3.63 to 13.52) | -0.11  (-0.38 to 0.17) | 0.451 | |
|  | | Liberia | 590.31  (253.90 to 1403.76) | 2204.03  (1271.36 to 3565.34) | 4.24  (3.19 to 5.29) | <0.001 | 11.62  (6.05 to 19.01) | 8.35  (3.74 to 14.39) | -1.05  (-1.33 to -0.76) | <0.001 | |
|  | | Libya | 13.13  (0.79 to 61.71) | 69.27  (0.55 to 626.93) | 5.53  (5.3 to 5.76) | <0.001 | 0.13  (0.03 to 0.34) | 0.16  (0.06 to 0.36) | 0.62  (0.33 to 0.91) | <0.001 | |
|  | | Lithuania | 97.83  (95.95 to 101.15) | 584.81  (579.82 to 592.39) | 6.08  (5.09 to 7.08) | <0.001 | 0.45  (0.34 to 0.59) | 0.25  (0.16 to 0.43) | -2  (-2.85 to -1.14) | <0.001 | |
|  | | Luxembourg | 34.58  (32.67 to 37.29) | 24.45  (19.55 to 31.63) | -1.39  (-1.8 to -0.99) | <0.001 | 0.15  (0.10 to 0.22) | 0.07  (0.04 to 0.12) | -2.47  (-3.05 to -1.9) | <0.001 | |
|  | | Madagascar | 1.07  (0.26 to 2.70) | 1106.79  (588.72 to 1933.32) | 25.38  (24 to 26.79) | <0.001 | 18.84  (10.26 to 31.06) | 11.58  (5.77 to 19.94) | -1.59  (-1.9 to -1.28) | <0.001 | |
|  | | Malawi | 16456.08  (9079.53 to 27708.75) | 9857.36  (5657.29 to 15901.22) | -1.7  (-2.6 to -0.79) | <0.001 | 10.68  (5.16 to 18.44) | 6.03  (3.46 to 10.93) | -1.83  (-2.05 to -1.61) | <0.001 | |
|  | | Malaysia | 53.98  (52.70 to 55.58) | 162.09  (154.77 to 173.33) | 3.69  (2.84 to 4.56) | <0.001 | 0.44  (0.23 to 0.86) | 0.30  (0.17 to 0.52) | -1.27  (-1.62 to -0.93) | <0.001 | |
|  | | Maldives | 8.78  (8.40 to 9.27) | 52.62  (52.05 to 53.97) | 5.87  (5.53 to 6.21) | <0.001 | 2.53  (1.15 to 4.89) | 0.68  (0.35 to 1.26) | -4.18  (-4.34 to -4.01) | <0.001 | |
|  | | Mali | 670.07  (318.05 to 1349.43) | 2033.08  (1138.37 to 3472.34) | 3.61  (2.28 to 4.96) | <0.001 | 9.35  (4.22 to 15.96) | 6.88  (2.95 to 12.27) | -0.94  (-1.24 to -0.63) | <0.001 | |
|  | | Malta | 21.14  (20.50 to 22.10) | 20.87  (15.70 to 28.34) | 0.09  (-0.42 to 0.6) | 0.744 | 0.09  (0.06 to 0.14) | 0.06  (0.03 to 0.11) | -1.1  (-1.36 to -0.85) | <0.001 | |
|  | | Marshall Islands | 12.72  (3.04 to 27.71) | 445.76  (207.67 to 797.51) | 11.95  (10.96 to 12.94) | <0.001 | 4.11  (1.08 to 8.79) | 6.74  (2.30 to 12.94) | 1.65  (1.39 to 1.91) | <0.001 | |
|  | | Mauritania | 3.93  (1.64 to 6.64) | 5.63  (2.38 to 9.71) | 1.14  (0.77 to 1.5) | <0.001 | 6.77  (2.84 to 11.98) | 2.84  (1.82 to 4.45) | -2.86  (-3.12 to -2.6) | <0.001 | |
|  | | Mauritius | 15.41  (15.09 to 15.78) | 190.68  (183.11 to 202.50) | 8.75  (5.13 to 12.49) | <0.001 | 0.25  (0.10 to 0.56) | 0.58  (0.39 to 0.91) | 2.81  (2.06 to 3.58) | <0.001 | |
|  | | Mexico | 94.66  (93.58 to 96.05) | 133.42  (129.03 to 139.70) | 1.19  (0.73 to 1.64) | <0.001 | 1.39  (1.21 to 1.68) | 1.36  (1.06 to 1.89) | -0.05  (-0.37 to 0.27) | 0.771 | |
|  | | Micronesia  (Federated States of) | 13.47  (3.24 to 28.91) | 470.75  (214.08 to 841.01) | 11.92  (10.93 to 12.92) | <0.001 | 4.33  (1.22 to 8.86) | 5.12  (1.62 to 10.47) | 0.53  (0.31 to 0.76) | <0.001 | |
|  | | Monaco | 14.61  (5.63 to 29.93) | 8.15  (4.19 to 15.22) | -1.87  (-2.32 to -1.41) | <0.001 | 0.21  (0.05 to 0.57) | 0.16  (0.04 to 0.40) | -0.86  (-1.11 to -0.61) | <0.001 | |
|  | | Mongolia | 6.20  (6.20 to 6.20) | 14.09  (13.45 to 15.55) | 3.14  (2.58 to 3.7) | <0.001 | 3.49  (1.88 to 6.80) | 2.05  (1.32 to 3.10) | -1.76  (-2.55 to -0.97) | <0.001 | |
|  | | Montenegro | 8.40  (8.30 to 8.60) | 9.65  (9.09 to 10.55) | 0.56  (-0.06 to 1.18) | 0.077 | 0.14  (0.09 to 0.22) | 0.12  (0.07 to 0.21) | -0.2  (-0.8 to 0.42) | 0.53 | |
|  | | Morocco | 27.53  (11.22 to 58.00) | 49.83  (22.89 to 95.57) | 1.76  (1.19 to 2.33) | <0.001 | 1.99  (1.26 to 3.15) | 1.25  (0.71 to 2.28) | -1.55  (-1.98 to -1.12) | <0.001 | |
|  | | Mozambique | 1361.30  (874.12 to 2139.53) | 21350.55  (18872.62 to 24469.00) | 9.13  (8.5 to 9.76) | <0.001 | 29.26  (17.19 to 47.12) | 11.61  (5.74 to 20.51) | -3  (-3.49 to -2.51) | <0.001 | |
|  | | Myanmar | 11.21  (5.54 to 20.78) | 542.83  (255.13 to 920.26) | 13.61  (11.81 to 15.45) | <0.001 | 1.72  (1.11 to 2.88) | 1.25  (0.80 to 1.92) | -1  (-1.14 to -0.85) | <0.001 | |
|  | | Namibia | 2339.75  (1213.82 to 4100.66) | 11464.92  (6616.72 to 18871.86) | 5.1  (3.75 to 6.45) | <0.001 | 6.06  (2.74 to 10.78) | 3.96  (2.52 to 6.61) | -1.31  (-1.83 to -0.79) | <0.001 | |
|  | | Nauru | 5.54  (1.38 to 11.91) | 163.60  (62.29 to 316.76) | 11.45  (10.61 to 12.31) | <0.001 | 5.72  (1.64 to 11.77) | 12.51  (5.52 to 22.41) | 2.63  (2.31 to 2.95) | <0.001 | |
|  | | Nepal | NA | 59.31  (25.94 to 120.45) | NA | NA | 5.99  (2.66 to 11.13) | 2.78  (1.70 to 4.59) | -2.42  (-2.56 to -2.29) | <0.001 | |
|  | | Netherlands | 25.97  (24.87 to 27.63) | 17.78  (13.92 to 23.70) | -1.27  (-1.93 to -0.61) | <0.001 | 0.13  (0.09 to 0.19) | 0.08  (0.05 to 0.13) | -1.62  (-1.78 to -1.47) | <0.001 | |
|  | | New Zealand | 10.29  (9.92 to 10.90) | 6.75  (5.22 to 9.43) | -1.28  (-2.73 to 0.19) | 0.085 | 0.20  (0.11 to 0.37) | 0.13  (0.06 to 0.28) | -1.4  (-2.34 to -0.44) | 0.004 | |
|  | | Nicaragua | 62.97  (62.51 to 63.65) | 131.46  (124.55 to 142.42) | 2.27  (1.59 to 2.96) | <0.001 | 0.76  (0.51 to 1.16) | 0.51  (0.32 to 0.78) | -1.23  (-1.53 to -0.93) | <0.001 | |
|  | | Niger | 397.40  (184.24 to 823.57) | 384.13  (186.48 to 716.30) | -0.17  (-1.3 to 0.97) | 0.768 | 2.01  (1.27 to 3.41) | 1.09  (0.66 to 2.16) | -1.98  (-2.15 to -1.82) | <0.001 | |
|  | | Nigeria | 1065.93  (614.18 to 1808.15) | 3344.07  (2255.25 to 4801.59) | 3.81  (2.82 to 4.82) | <0.001 | 4.13  (2.53 to 6.15) | 3.16  (1.98 to 4.69) | -0.86  (-0.98 to -0.73) | <0.001 | |
|  | | Niue | 5.87  (1.44 to 12.70) | 136.14  (48.93 to 270.52) | 10.51  (9.28 to 11.76) | <0.001 | 2.83  (1.19 to 5.59) | 5.89  (1.85 to 12.30) | 2.47  (2.23 to 2.71) | <0.001 | |
|  | | North Macedonia | 1.84  (1.82 to 1.87) | 2.21  (2.17 to 2.28) | 0.73  (-0.56 to 2.04) | 0.27 | 0.20  (0.09 to 0.41) | 0.11  (0.05 to 0.26) | -1.8  (-2.35 to -1.25) | <0.001 | |
|  | | Northern Mariana Islands | 66.72  (66.24 to 67.28) | 205.51  (202.21 to 210.58) | 4.85  (2.34 to 7.42) | <0.001 | 1.01  (0.42 to 2.17) | 2.16  (1.18 to 3.69) | 2.56  (1.88 to 3.24) | <0.001 | |
|  | | Norway | 6.76  (6.45 to 7.16) | 22.04  (16.78 to 29.82) | 3.15  (1.15 to 5.19) | 0.002 | 0.11  (0.07 to 0.18) | 0.12  (0.08 to 0.18) | 0.17  (-0.52 to 0.87) | 0.629 | |
|  | | Oman | 13.16  (12.96 to 13.42) | 76.29  (73.69 to 79.99) | 5.82  (5.55 to 6.09) | <0.001 | 0.11  (0.05 to 0.22) | 0.07  (0.03 to 0.13) | -1.53  (-1.95 to -1.1) | <0.001 | |
|  | | Pakistan | 0.01  (0.00 to 0.06) | 229.91  (1.86 to 1347.71) | 38.21  (36.15 to 40.3) | <0.001 | 3.67  (1.68 to 6.66) | 3.61  (2.21 to 5.65) | 0  (-0.16 to 0.15) | 0.957 | |
|  | | Palau | 5.57  (1.37 to 12.11) | 132.12  (48.59 to 256.41) | 10.61  (9.67 to 11.56) | <0.001 | 0.87  (0.40 to 1.65) | 2.90  (0.49 to 7.04) | 4.17  (3.92 to 4.42) | <0.001 | |
|  | | Palestine | 1.21  (1.19 to 1.24) | 13.15  (12.44 to 14.30) | 9.06  (3.64 to 14.77) | 0.001 | 0.24  (0.10 to 0.50) | 0.24  (0.09 to 0.47) | -0.01  (-0.3 to 0.29) | 0.97 | |
|  | | Panama | 271.30  (266.24 to 279.02) | 498.28  (478.06 to 529.02) | 2  (1.75 to 2.27) | <0.001 | 1.06  (0.75 to 1.53) | 0.97  (0.65 to 1.45) | -0.3  (-0.9 to 0.3) | 0.326 | |
|  | | Papua New Guinea | 2.94  (0.89 to 6.90) | 636.72  (326.35 to 1125.75) | 19.09  (17.09 to 21.12) | <0.001 | 4.20  (1.25 to 8.41) | 4.02  (1.13 to 8.08) | -0.15  (-0.46 to 0.16) | 0.347 | |
|  | | Paraguay | 66.16  (64.50 to 68.58) | 193.21  (185.12 to 206.04) | 3.72  (2.99 to 4.45) | <0.001 | 2.52  (1.65 to 3.86) | 2.64  (1.75 to 4.01) | 0.27  (-0.12 to 0.66) | 0.173 | |
|  | | Peru | 66.26  (63.93 to 69.32) | 159.71  (153.27 to 169.74) | 2.95  (1.64 to 4.29) | <0.001 | 1.55  (1.04 to 2.32) | 0.62  (0.30 to 1.22) | -3.03  (-3.65 to -2.41) | <0.001 | |
|  | | Philippines | 1.11  (1.09 to 1.15) | 203.81  (193.14 to 226.26) | 19.03  (17.55 to 20.53) | <0.001 | 0.84  (0.52 to 1.35) | 1.94  (1.27 to 2.79) | 2.81  (2.49 to 3.13) | <0.001 | |
|  | | Poland | 1.30  (1.23 to 1.43) | 12.78  (11.62 to 14.48) | 7.54  (4.22 to 10.96) | <0.001 | 0.27  (0.23 to 0.34) | 0.13  (0.09 to 0.19) | -2.47  (-2.76 to -2.18) | <0.001 | |
|  | | Portugal | 125.81  (124.60 to 127.22) | 83.52  (79.25 to 89.01) | -1.28  (-3.14 to 0.61) | 0.182 | 0.16  (0.11 to 0.23) | 0.08  (0.05 to 0.14) | -2.26  (-2.7 to -1.81) | <0.001 | |
|  | | Puerto Rico | 816.06  (804.13 to 832.98) | 200.65  (197.31 to 205.61) | -4.32  (-6.35 to -2.25) | <0.001 | 0.63  (0.40 to 1.06) | 0.76  (0.48 to 1.22) | 0.71  (-0.43 to 1.87) | 0.224 | |
|  | | Qatar | 24.09  (23.42 to 25.13) | 20.65  (20.47 to 20.89) | -0.29  (-4.48 to 4.09) | 0.895 | 0.51  (0.22 to 1.05) | 0.30  (0.11 to 0.63) | -1.48  (-2.28 to -0.67) | <0.001 | |
|  | | Republic of Korea | 1.21  (0.84 to 1.46) | 2.06  (1.76 to 2.50) | 1.89  (1.08 to 2.69) | <0.001 | 0.23  (0.12 to 0.44) | 0.20  (0.09 to 0.40) | -0.55  (-0.79 to -0.3) | <0.001 | |
|  | | Republic of Moldova | 39.59  (39.10 to 40.53) | 270.16  (259.18 to 287.15) | 6.66  (6.17 to 7.16) | <0.001 | 1.07  (0.79 to 1.42) | 0.57  (0.39 to 0.81) | -2.24  (-2.56 to -1.91) | <0.001 | |
|  | | Romania | 33.23  (32.66 to 33.86) | 67.16  (61.77 to 75.95) | 2.86  (2.28 to 3.43) | <0.001 | 0.23  (0.15 to 0.39) | 0.14  (0.07 to 0.28) | -1.69  (-2.21 to -1.16) | <0.001 | |
|  | | Russian Federation | 72.50  (71.54 to 74.53) | 816.84  (781.29 to 869.23) | 8.15  (7.27 to 9.05) | <0.001 | 0.84  (0.78 to 0.96) | 0.68  (0.57 to 0.84) | -0.62  (-1.95 to 0.73) | 0.366 | |
|  | | Rwanda | 3613.52  (1506.74 to 9011.26) | 2999.96  (1691.23 to 4821.40) | -0.48  (-1.06 to 0.1) | 0.102 | 15.07  (7.37 to 28.61) | 5.05  (2.92 to 9.80) | -3.45  (-3.89 to -3.01) | <0.001 | |
|  | | Saint Kitts and Nevis | 112.35  (38.28 to 267.36) | 845.38  (460.96 to 1360.72) | 6.6  (5.85 to 7.36) | <0.001 | 3.99  (2.81 to 5.68) | 2.07  (1.35 to 3.20) | -2.07  (-3.02 to -1.1) | <0.001 | |
|  | | Saint Lucia | 300.00  (295.67 to 305.49) | 206.31  (200.36 to 217.89) | -0.97  (-1.76 to -0.18) | 0.016 | 2.24  (1.64 to 3.13) | 3.11  (2.21 to 4.33) | 1.1  (-0.25 to 2.46) | 0.112 | |
|  | | Saint Vincent and the Grenadines | 969.06  (954.44 to 986.16) | 899.29  (876.63 to 940.31) | 0.02  (-1.14 to 1.2) | 0.972 | 0.76  (0.50 to 1.21) | 0.90  (0.60 to 1.37) | 0.44  (-0.51 to 1.4) | 0.361 | |
|  | | Samoa | 13.27  (3.18 to 29.10) | 449.05  (201.19 to 805.34) | 11.88  (11.07 to 12.7) | <0.001 | 0.64  (0.33 to 1.17) | 0.95  (0.58 to 1.53) | 1.3  (1.13 to 1.47) | <0.001 | |
|  | | San Marino | 14.61  (5.64 to 29.87) | 6.74  (3.46 to 12.51) | -2.49  (-3.07 to -1.91) | <0.001 | 0.21  (0.05 to 0.60) | 0.18  (0.04 to 0.45) | -0.5  (-0.61 to -0.38) | <0.001 | |
|  | | Sao Tome and Principe | 2.85  (1.39 to 5.28) | 3.91  (2.74 to 5.34) | 1.04  (0.48 to 1.59) | <0.001 | 2.25  (1.34 to 3.77) | 1.98  (1.24 to 3.09) | -0.44  (-1.1 to 0.23) | 0.2 | |
|  | | Saudi Arabia | 49.30  (48.34 to 50.60) | 150.53  (146.29 to 163.23) | 4  (2.49 to 5.53) | <0.001 | 0.16  (0.09 to 0.28) | 0.09  (0.04 to 0.18) | -1.85  (-2.12 to -1.58) | <0.001 | |
|  | | Senegal | 439.66  (231.26 to 785.46) | 811.69  (434.20 to 1354.14) | 1.98  (0.94 to 3.03) | <0.001 | 2.55  (1.69 to 4.00) | 1.42  (0.92 to 2.29) | -1.92  (-2.3 to -1.55) | <0.001 | |
|  | | Serbia | 11.52  (10.39 to 12.58) | 9.49  (9.25 to 10.06) | -0.2  (-1.05 to 0.66) | 0.648 | 0.16  (0.10 to 0.25) | 0.09  (0.05 to 0.16) | -1.71  (-1.95 to -1.47) | <0.001 | |
|  | | Seychelles | 46.79  (46.08 to 47.74) | 302.59  (294.00 to 321.43) | 6.56  (4.11 to 9.07) | <0.001 | 2.44  (1.36 to 3.78) | 1.36  (0.90 to 2.02) | -1.75  (-2.34 to -1.17) | <0.001 | |
|  | | Sierra Leone | 339.95  (154.87 to 730.91) | 2023.36  (1109.74 to 3318.74) | 5.83  (4.61 to 7.06) | <0.001 | 3.12  (2.11 to 4.65) | 2.44  (1.59 to 3.79) | -0.79  (-0.89 to -0.69) | <0.001 | |
|  | | Singapore | 2.29  (2.12 to 2.55) | 4.82  (3.24 to 7.22) | 2.5  (2.09 to 2.91) | <0.001 | 0.21  (0.12 to 0.42) | 0.19  (0.09 to 0.36) | -0.48  (-0.73 to -0.22) | <0.001 | |
|  | | Slovakia | 1.77  (1.75 to 1.80) | 2.01  (1.93 to 2.13) | 0.23  (-0.95 to 1.43) | 0.704 | 0.25  (0.17 to 0.36) | 0.17  (0.11 to 0.26) | -1.34  (-1.82 to -0.86) | <0.001 | |
|  | | Slovenia | 3.02  (2.96 to 3.07) | 2.89  (2.71 to 3.22) | 0.11  (-0.68 to 0.9) | 0.788 | 0.18  (0.13 to 0.24) | 0.08  (0.05 to 0.14) | -2.49  (-2.93 to -2.05) | <0.001 | |
|  | | Solomon Islands | 13.74  (3.25 to 29.87) | 462.10  (209.93 to 834.62) | 11.78  (10.78 to 12.79) | <0.001 | 5.46  (1.91 to 10.58) | 4.95  (1.64 to 9.61) | -0.32  (-0.46 to -0.18) | <0.001 | |
|  | | Somalia | 33.77  (6.10 to 98.39) | 1359.73  (698.65 to 2392.26) | 12.12  (11.05 to 13.19) | <0.001 | 16.71  (8.36 to 31.95) | 13.88  (6.82 to 26.07) | -0.58  (-0.76 to -0.4) | <0.001 | |
|  | | South Africa | 776.90  (644.20 to 937.78) | 15603.90  (14284.79 to 16952.72) | 9.77  (8.04 to 11.52) | <0.001 | 11.69  (7.37 to 16.97) | 5.92  (3.82 to 9.00) | -2.14  (-2.78 to -1.51) | <0.001 | |
|  | | South Sudan | 614.81  (250.37 to 1649.79) | 3434.05  (1567.86 to 7505.72) | 5.57  (4.53 to 6.61) | <0.001 | 14.66  (7.63 to 26.28) | 13.92  (6.90 to 26.96) | -0.22  (-0.61 to 0.18) | 0.285 | |
|  | | Spain | 219.91  (212.96 to 229.79) | 35.99  (32.77 to 40.35) | -6.72  (-7.78 to -5.64) | <0.001 | 0.16  (0.12 to 0.23) | 0.08  (0.05 to 0.14) | -2.3  (-2.58 to -2.03) | <0.001 | |
|  | | Sri Lanka | 8.90  (8.70 to 9.19) | 33.34  (32.49 to 34.90) | 4.62  (2.22 to 7.07) | <0.001 | 0.75  (0.48 to 1.17) | 0.40  (0.23 to 0.64) | -2.02  (-2.39 to -1.64) | <0.001 | |
|  | | Sudan | 133.34  (49.56 to 419.94) | 1508.16  (694.75 to 3576.69) | 8.05  (7.58 to 8.53) | <0.001 | 2.01  (1.31 to 3.00) | 1.16  (0.71 to 1.91) | -1.82  (-2.1 to -1.54) | <0.001 | |
|  | | Suriname | 949.73  (937.12 to 966.02) | 1209.62  (1156.14 to 1294.91) | 0.83  (-0.27 to 1.95) | 0.14 | 0.96  (0.59 to 1.47) | 1.25  (0.75 to 2.14) | 0.91  (0.32 to 1.5) | 0.003 | |
|  | | Sweden | 9.58  (9.10 to 10.32) | 13.19  (10.44 to 16.92) | 1.17  (-0.91 to 3.29) | 0.273 | 0.10  (0.07 to 0.17) | 0.08  (0.05 to 0.15) | -0.59  (-1.47 to 0.31) | 0.198 | |
|  | | Switzerland | 55.61  (51.96 to 61.42) | 29.74  (24.38 to 37.35) | -2.25  (-2.94 to -1.55) | <0.001 | 0.16  (0.12 to 0.22) | 0.08  (0.06 to 0.13) | -2.16  (-2.55 to -1.77) | <0.001 | |
|  | | Syrian Arab Republic | 5.00  (4.94 to 5.08) | 17.18  (16.98 to 17.54) | 3.83  (3.14 to 4.53) | <0.001 | 1.45  (0.83 to 2.32) | 0.69  (0.33 to 1.59) | -2.44  (-3.04 to -1.83) | <0.001 | |
|  | | Taiwan  (Province of China) | 1.25  (1.19 to 1.31) | 8.81  (8.46 to 9.44) | 4.31  (2.65 to 5.99) | <0.001 | 0.24  (0.15 to 0.40) | 0.19  (0.12 to 0.33) | -0.71  (-1.49 to 0.08) | 0.079 | |
|  | | Tajikistan | 136.33  (135.23 to 137.75) | 134.16  (130.12 to 139.58) | -0.06  (-0.53 to 0.42) | 0.818 | 1.44  (0.79 to 2.39) | 0.68  (0.29 to 1.60) | -2.46  (-2.65 to -2.27) | <0.001 | |
|  | | Thailand | 937.34  (915.15 to 961.85) | 712.84  (663.89 to 796.48) | -0.45  (-2.05 to 1.18) | 0.588 | 0.49  (0.22 to 0.95) | 0.39  (0.21 to 0.68) | -0.77  (-1.19 to -0.35) | <0.001 | |
|  | | Timor-Leste | 17.85  (6.62 to 41.38) | 921.05  (542.04 to 1425.33) | 13.4  (12.56 to 14.24) | <0.001 | 1.29  (0.87 to 1.91) | 1.12  (0.74 to 1.73) | -0.44  (-0.61 to -0.27) | <0.001 | |
|  | | Togo | 1500.89  (766.04 to 2786.28) | 2573.38  (1386.09 to 4326.33) | 1.71  (0.85 to 2.58) | <0.001 | 3.80  (2.00 to 6.53) | 2.51  (1.66 to 3.93) | -1.29  (-1.62 to -0.97) | <0.001 | |
|  | | Tokelau | 6.05  (1.50 to 12.99) | 137.09  (48.64 to 276.57) | 10.44  (8.9 to 12.01) | <0.001 | 8.67  (2.19 to 19.19) | 7.52  (2.66 to 14.26) | -0.43  (-0.62 to -0.23) | <0.001 | |
|  | | Tonga | 32.12  (31.39 to 32.89) | 77.64  (66.84 to 90.85) | 2.87  (2.67 to 3.07) | <0.001 | 0.83  (0.44 to 1.60) | 1.73  (1.05 to 2.73) | 2.42  (2.25 to 2.58) | <0.001 | |
|  | | Trinidad and Tobago | 664.36  (652.40 to 686.19) | 861.69  (827.35 to 909.22) | 1.09  (0.12 to 2.07) | 0.027 | 0.84  (0.61 to 1.19) | 0.98  (0.66 to 1.45) | 0.51  (-0.8 to 1.84) | 0.445 | |
|  | | Tunisia | 0.44  (0.03 to 1.62) | 16.06  (5.62 to 34.62) | 11.91  (10.05 to 13.81) | <0.001 | 0.13  (0.03 to 0.34) | 0.12  (0.03 to 0.32) | -0.29  (-0.41 to -0.17) | <0.001 | |
|  | | Turkmenistan | 100.43  (99.19 to 101.93) | 159.60  (157.21 to 163.07) | 1.48  (0.02 to 2.97) | 0.047 | 1.23  (0.93 to 1.57) | 0.57  (0.40 to 0.82) | -2.46  (-3.7 to -1.19) | <0.001 | |
|  | | Tuvalu | 6.13  (1.51 to 13.12) | 148.38  (52.82 to 305.62) | 10.63  (9.15 to 12.12) | <0.001 | 5.08  (0.98 to 11.77) | 6.85  (2.05 to 13.83) | 1.06  (0.82 to 1.31) | <0.001 | |
|  | | Türkiye | 1.54  (1.47 to 1.62) | 12.26  (11.87 to 13.00) | 6.95  (6.41 to 7.48) | <0.001 | 0.33  (0.17 to 0.65) | 0.19  (0.05 to 0.48) | -1.8  (-1.96 to -1.63) | <0.001 | |
|  | | Uganda | 42424.33  (23052.22 to 67294.66) | 7342.71  (4108.19 to 11974.54) | -5.55  (-6.11 to -4.97) | <0.001 | 9.06  (4.30 to 15.69) | 10.79  (5.48 to 18.29) | 0.55  (0.26 to 0.84) | <0.001 | |
|  | | Ukraine | 88.41  (86.78 to 91.84) | 778.11  (743.41 to 836.25) | 7.43  (6.54 to 8.33) | <0.001 | 0.38  (0.29 to 0.50) | 0.45  (0.26 to 0.73) | 0.49  (-0.36 to 1.36) | 0.26 | |
|  | | United Arab Emirates | 4.95  (2.16 to 10.38) | 13.35  (5.87 to 25.94) | 3.1  (2.1 to 4.12) | <0.001 | 0.68  (0.24 to 1.83) | 0.35  (0.16 to 0.71) | -2.08  (-3.14 to -1.01) | <0.001 | |
|  | | United Kingdom | 10.78  (10.00 to 11.94) | 26.51  (21.33 to 33.44) | 3.01  (1.94 to 4.1) | <0.001 | 0.21  (0.16 to 0.30) | 0.18  (0.13 to 0.28) | -0.52  (-1.12 to 0.09) | 0.096 | |
|  | | United Republic of Tanzania | 11646.85  (6234.63 to 20236.71) | 6062.32  (3452.89 to 9749.46) | -2.34  (-3.36 to -1.32) | <0.001 | 14.50  (7.45 to 24.29) | 6.81  (2.93 to 12.41) | -2.43  (-2.76 to -2.1) | <0.001 | |
|  | | United States of America | 233.93  (224.50 to 247.26) | 65.37  (53.86 to 80.90) | -4.26  (-4.9 to -3.61) | <0.001 | 0.39  (0.28 to 0.60) | 0.31  (0.20 to 0.51) | -0.79  (-1.04 to -0.54) | <0.001 | |
|  | | United States Virgin Islands | 313.06  (309.36 to 318.87) | 254.18  (247.52 to 264.06) | -0.97  (-1.72 to -0.22) | 0.011 | 1.08  (0.50 to 2.07) | 1.46  (0.78 to 2.75) | 1.25  (0.56 to 1.95) | <0.001 | |
|  | | Uruguay | 69.59  (67.95 to 72.44) | 244.87  (226.67 to 271.61) | 4.36  (3.95 to 4.78) | <0.001 | 0.75  (0.47 to 1.27) | 0.70  (0.42 to 1.24) | -0.32  (-0.94 to 0.31) | 0.314 | |
|  | | Uzbekistan | 52.77  (52.12 to 53.48) | 112.70  (107.25 to 121.61) | 2.46  (1.37 to 3.55) | <0.001 | 0.83  (0.60 to 1.10) | 0.25  (0.18 to 0.35) | -3.89  (-4.72 to -3.05) | <0.001 | |
|  | | Vanuatu | 13.16  (3.23 to 28.66) | 456.83  (208.12 to 824.80) | 11.91  (10.94 to 12.89) | <0.001 | 3.79  (0.99 to 7.71) | 5.17  (1.80 to 10.09) | 1.02  (0.81 to 1.23) | <0.001 | |
|  | | Venezuela  (Bolivarian Republic of) | 104.05  (103.08 to 105.26) | 275.02  (264.55 to 289.84) | 3.24  (2.61 to 3.87) | <0.001 | 1.31  (0.91 to 2.01) | 1.65  (1.12 to 2.43) | 0.79  (-0.03 to 1.62) | 0.059 | |
|  | | Viet Nam | 15.37  (7.21 to 26.86) | 163.68  (104.65 to 265.00) | 8.19  (7.08 to 9.31) | <0.001 | 0.31  (0.19 to 0.54) | 0.19  (0.10 to 0.35) | -1.56  (-1.69 to -1.43) | <0.001 | |
|  | | Yemen | 6.60  (1.44 to 17.29) | 72.84  (25.00 to 143.95) | 8.04  (7.79 to 8.29) | <0.001 | 0.40  (0.26 to 0.63) | 0.31  (0.19 to 0.50) | -0.83  (-0.98 to -0.68) | <0.001 | |
|  | | Zambia | 18509.18  (9844.51 to 31930.60) | 12500.67  (6742.64 to 21272.59) | -1.46  (-2.42 to -0.49) | 0.003 | 17.43  (9.08 to 29.43) | 9.82  (4.42 to 19.76) | -1.88  (-2.1 to -1.66) | <0.001 | |
|  | | Zimbabwe | 21332.88  (11354.56 to 36372.40) | 12867.26  (7331.81 to 20926.80) | -1.73  (-2.2 to -1.26) | <0.001 | 4.95  (2.99 to 9.11) | 5.29  (2.19 to 10.50) | 0.13  (-0.18 to 0.45) | 0.409 | |

**Abbreviations:** DALYs, Disability-Adjusted Life Years; AAPC, average annual percent change; CI, confidence interval.
