# Supplementary material for: Global, regional, and national burden of HIV and other sexually transmitted infections among women of childbearing age from 1990 to 2021
Source: Microbiol Spectr. 2025 Oct 24;13(12):e00488-25. doi: 10.1128/spectrum.00488-25 (PMC12671144; doi:10.1128/spectrum.00488-25)
Supplement: Table S4 — The age-standardized incidence, prevalence, mortality, and DALY rates of 204 countries and territories. [file spectrum.00488-25-s0005.docx]

**Table 4**: The age-standardized incidence, prevalence, mortality and DALYs rates of 204 countries and territories.

|  |  | Chlamydial infection | | | | Gonococcal infection | | | |
| --- | --- | --- | --- | --- | --- | --- | --- | --- | --- |
|  |  | 1990 | 2021 | AAPC (95% CI) | P | 1990 | 2021 | AAPC (95% CI) | P |
| Age-standardized Incidence | Afghanistan | 5509.54  (3073.06 to 8896.34) | 5341.73  (2959.88 to 8783.56) | -0.09  (-0.13 to -0.06) | <0.001 | 1647.21  (827.17 to 2962.42) | 1614.18  (798.87 to 2957.51) | -0.05  (-0.12 to 0.02) | 0.132 |
|  | Albania | 5204.42  (2896.05 to 8548.39) | 5103.99  (2859.24 to 8465.75) | -0.07  (-0.14 to 0.01) | 0.084 | 4231.05  (2126.05 to 7561.29) | 3886.29  (1947.78 to 6977.10) | -0.28  (-0.32 to -0.24) | <0.001 |
|  | Algeria | 6687.03  (3860.71 to 10760.63) | 5018.36  (2780.27 to 8188.72) | -0.88  (-0.95 to -0.81) | <0.001 | 342.84  (169.32 to 606.73) | 331.10  (166.84 to 588.62) | -0.12  (-0.21 to -0.04) | 0.003 |
|  | American Samoa | 10544.30  (5920.39 to 17040.77) | 10401.13  (5900.55 to 17061.70) | -0.05  (-0.05 to -0.04) | <0.001 | 2373.97  (1164.39 to 4264.11) | 2213.60  (1120.03 to 4006.65) | -0.22  (-0.33 to -0.1) | <0.001 |
|  | Andorra | 535.74  (287.14 to 893.87) | 531.28  (282.72 to 893.71) | -0.03  (-0.06 to 0) | 0.068 | 139.27  (66.59 to 257.69) | 133.53  (63.75 to 246.80) | -0.16  (-0.21 to -0.11) | <0.001 |
|  | Angola | 3152.66  (1728.30 to 5249.11) | 3082.73  (1679.29 to 5149.88) | -0.08  (-0.11 to -0.05) | <0.001 | 1447.19  (724.60 to 2626.35) | 1325.14  (647.72 to 2412.81) | -0.26  (-0.38 to -0.15) | <0.001 |
|  | Antigua and Barbuda | 9695.14  (5530.34 to 15568.53) | 9704.36  (5572.39 to 15775.03) | 0  (-0.03 to 0.04) | 0.794 | 1735.06  (864.93 to 3125.28) | 1601.23  (780.66 to 2909.79) | -0.27  (-0.32 to -0.21) | <0.001 |
|  | Argentina | 1217.83  (713.13 to 1958.96) | 1333.37  (723.48 to 2242.24) | 0.29  (0.2 to 0.38) | <0.001 | 427.06  (211.61 to 779.54) | 432.69  (212.66 to 796.20) | 0.02  (-0.05 to 0.09) | 0.54 |
|  | Armenia | 12928.62  (7391.21 to 20677.09) | 12949.97  (7432.69 to 20566.77) | 0  (-0.09 to 0.09) | 0.95 | 5168.51  (2582.28 to 9201.55) | 4210.95  (2133.26 to 7598.88) | -0.66  (-0.72 to -0.6) | <0.001 |
|  | Australia | 1123.27  (612.96 to 1872.54) | 1121.28  (601.44 to 1874.83) | 0  (-0.04 to 0.04) | 0.989 | 211.33  (102.83 to 389.00) | 192.73  (94.99 to 351.26) | -0.31  (-0.34 to -0.29) | <0.001 |
|  | Austria | 533.91  (284.40 to 899.73) | 531.86  (283.47 to 896.98) | -0.01  (-0.02 to 0) | 0.104 | 139.17  (66.94 to 256.60) | 125.50  (60.22 to 234.21) | -0.35  (-0.41 to -0.28) | <0.001 |
|  | Azerbaijan | 13213.06  (7601.58 to 20946.64) | 13080.29  (7458.68 to 20927.41) | -0.03  (-0.05 to -0.02) | <0.001 | 5229.79  (2694.16 to 9315.06) | 4456.34  (2281.02 to 7933.13) | -0.52  (-0.55 to -0.48) | <0.001 |
|  | Bahamas | 9755.83  (5517.12 to 15853.91) | 9708.96  (5393.74 to 15886.55) | -0.01  (-0.03 to 0) | 0.02 | 1692.58  (846.61 to 3061.10) | 1571.27  (782.99 to 2885.03) | -0.25  (-0.28 to -0.22) | <0.001 |
|  | Bahrain | 5289.26  (2920.32 to 8661.88) | 5071.62  (2831.37 to 8296.09) | -0.1  (-0.12 to -0.07) | <0.001 | 1524.33  (761.94 to 2788.23) | 1480.45  (728.75 to 2682.53) | -0.09  (-0.15 to -0.02) | 0.011 |
|  | Bangladesh | 4095.02  (2284.81 to 6744.56) | 4066.12  (2245.11 to 6662.70) | -0.03  (-0.04 to -0.01) | <0.001 | 916.44  (444.14 to 1686.76) | 737.96  (361.65 to 1355.84) | -0.69  (-0.73 to -0.66) | <0.001 |
|  | Barbados | 9754.45  (5570.18 to 15867.44) | 9698.16  (5539.29 to 15566.25) | -0.01  (-0.06 to 0.04) | 0.667 | 1733.90  (859.49 to 3096.15) | 1571.26  (779.44 to 2844.17) | -0.32  (-0.39 to -0.26) | <0.001 |
|  | Belarus | 7816.92  (4407.91 to 12838.89) | 7866.04  (4452.99 to 12922.89) | 0.02  (0.02 to 0.03) | <0.001 | 4390.55  (2217.87 to 7801.81) | 3648.43  (1829.37 to 6516.52) | -0.64  (-0.74 to -0.54) | <0.001 |
|  | Belgium | 908.90  (486.40 to 1507.94) | 903.11  (487.51 to 1525.02) | -0.03  (-0.15 to 0.09) | 0.611 | 142.60  (67.86 to 265.61) | 124.52  (60.32 to 232.48) | -0.46  (-0.61 to -0.31) | <0.001 |
|  | Belize | 9783.19  (5543.66 to 15881.22) | 9752.23  (5614.53 to 15824.30) | 0  (-0.02 to 0.02) | 0.736 | 1607.96  (804.28 to 2970.78) | 1519.70  (752.70 to 2762.61) | -0.19  (-0.23 to -0.14) | <0.001 |
|  | Benin | 2514.44  (1391.72 to 4121.35) | 2495.94  (1375.16 to 4148.28) | 0.01  (-0.15 to 0.17) | 0.916 | 1560.30  (772.93 to 2826.98) | 1501.86  (740.52 to 2736.83) | -0.12  (-0.2 to -0.03) | 0.008 |
|  | Bermuda | 9647.55  (5469.77 to 15726.60) | 9621.58  (5513.31 to 15651.09) | -0.01  (-0.01 to 0) | 0.055 | 1637.18  (806.88 to 2952.08) | 1490.45  (735.93 to 2719.47) | -0.3  (-0.35 to -0.24) | <0.001 |
|  | Bhutan | 4068.11  (2266.09 to 6740.33) | 3981.27  (2226.52 to 6517.07) | -0.07  (-0.08 to -0.07) | <0.001 | 796.36  (389.06 to 1453.53) | 687.52  (335.68 to 1263.80) | -0.47  (-0.5 to -0.43) | <0.001 |
|  | Bolivia (Plurinational State of) | 5064.92  (2848.47 to 8208.17) | 4997.27  (2747.87 to 8126.38) | 0.04  (-0.19 to 0.27) | 0.751 | 255.39  (124.75 to 464.99) | 230.77  (112.40 to 428.28) | -0.33  (-0.41 to -0.26) | <0.001 |
|  | Bosnia and Herzegovina | 5133.55  (2869.99 to 8435.53) | 5093.89  (2827.05 to 8457.82) | -0.03  (-0.04 to -0.01) | <0.001 | 4077.82  (2069.47 to 7308.27) | 3787.15  (1874.21 to 6889.56) | -0.25  (-0.29 to -0.2) | <0.001 |
|  | Botswana | 8082.98  (4563.02 to 13022.32) | 8006.19  (4467.06 to 13028.94) | -0.01  (-0.12 to 0.09) | 0.809 | 4453.53  (2294.37 to 7866.49) | 4130.29  (2097.63 to 7295.31) | -0.24  (-0.32 to -0.17) | <0.001 |
|  | Brazil | 10469.62  (5999.81 to 17165.13) | 10529.82  (6029.46 to 17133.67) | 0.01  (-0.18 to 0.2) | 0.926 | 1737.69  (812.63 to 3220.69) | 1666.91  (782.87 to 3068.05) | -0.13  (-0.28 to 0.03) | 0.12 |
|  | Brunei Darussalam | 1445.06  (787.59 to 2422.83) | 1450.73  (790.25 to 2425.26) | 0.01  (-0.03 to 0.05) | 0.716 | 483.61  (239.19 to 880.01) | 438.30  (213.44 to 800.39) | -0.33  (-0.41 to -0.25) | <0.001 |
|  | Bulgaria | 6624.61  (3715.93 to 10843.16) | 6654.92  (3684.29 to 10867.39) | 0  (-0.05 to 0.05) | 0.996 | 3984.75  (2011.03 to 7217.68) | 3710.88  (1865.50 to 6763.11) | -0.23  (-0.25 to -0.21) | <0.001 |
|  | Burkina Faso | 3686.52  (2055.49 to 6060.32) | 3624.28  (1989.40 to 6012.54) | -0.06  (-0.08 to -0.05) | <0.001 | 2542.84  (1238.53 to 4705.53) | 2314.21  (1114.10 to 4304.27) | -0.33  (-0.49 to -0.18) | <0.001 |
|  | Burundi | 4975.86  (2754.67 to 8063.29) | 4905.70  (2711.87 to 8043.36) | -0.04  (-0.06 to -0.03) | <0.001 | 2503.01  (1251.37 to 4535.89) | 2387.04  (1165.46 to 4263.16) | -0.2  (-0.23 to -0.17) | <0.001 |
|  | Cabo Verde | 3616.80  (1989.42 to 5978.97) | 3571.75  (1967.54 to 5926.18) | -0.04  (-0.05 to -0.02) | <0.001 | 1912.87  (951.62 to 3451.00) | 1853.24  (936.43 to 3354.93) | -0.11  (-0.12 to -0.09) | <0.001 |
|  | Cambodia | 6283.76  (3503.47 to 10263.66) | 6147.99  (3427.28 to 10127.96) | -0.07  (-0.1 to -0.04) | <0.001 | 696.83  (337.50 to 1267.25) | 605.75  (293.91 to 1108.01) | -0.44  (-0.5 to -0.38) | <0.001 |
|  | Cameroon | 3574.00  (1972.56 to 5915.68) | 3494.46  (1944.76 to 5778.98) | -0.06  (-0.08 to -0.04) | <0.001 | 2828.34  (1407.61 to 5100.26) | 2716.85  (1362.63 to 4909.86) | -0.13  (-0.23 to -0.03) | 0.009 |
|  | Canada | 491.88  (258.20 to 822.95) | 486.92  (259.02 to 808.16) | -0.02  (-0.13 to 0.08) | 0.651 | 153.24  (74.35 to 283.02) | 144.06  (70.30 to 265.71) | -0.2  (-0.25 to -0.14) | <0.001 |
|  | Central African Republic | 3621.08  (1987.92 to 6038.28) | 3588.13  (1985.69 to 5979.47) | -0.03  (-0.05 to -0.01) | 0.001 | 2018.75  (885.34 to 3897.29) | 2003.06  (883.82 to 3890.29) | -0.02  (-0.03 to 0) | 0.128 |
|  | Chad | 3716.73  (2058.03 to 6118.40) | 3653.07  (2010.74 to 6057.93) | -0.05  (-0.06 to -0.05) | <0.001 | 2218.68  (1115.49 to 3990.16) | 2055.24  (1025.35 to 3707.89) | -0.26  (-0.29 to -0.23) | <0.001 |
|  | Chile | 1350.85  (735.55 to 2272.40) | 1320.05  (720.78 to 2194.41) | -0.07  (-0.09 to -0.04) | <0.001 | 456.36  (222.19 to 830.59) | 417.14  (204.16 to 765.11) | -0.31  (-0.48 to -0.14) | <0.001 |
|  | China | 6199.26  (3514.34 to 10231.96) | 6915.34  (3910.56 to 11413.92) | 0.52  (-0.43 to 1.47) | 0.286 | 2073.37  (958.72 to 3838.29) | 1728.30  (799.57 to 3182.65) | -0.58  (-0.78 to -0.39) | <0.001 |
|  | Colombia | 5452.88  (3295.26 to 8557.25) | 6160.95  (3436.01 to 10075.87) | 0.4  (0.33 to 0.48) | <0.001 | 515.21  (260.79 to 911.10) | 488.20  (241.82 to 876.22) | -0.17  (-0.18 to -0.15) | <0.001 |
|  | Comoros | 4921.21  (2715.93 to 8086.74) | 4877.68  (2690.82 to 8013.91) | -0.03  (-0.04 to -0.01) | 0.004 | 2447.80  (1219.47 to 4486.57) | 2347.17  (1187.63 to 4133.62) | -0.15  (-0.16 to -0.13) | <0.001 |
|  | Congo | 3102.64  (1698.17 to 5159.28) | 3063.82  (1672.34 to 5187.93) | -0.05  (-0.1 to 0) | 0.058 | 1397.98  (688.54 to 2544.84) | 1304.32  (648.40 to 2354.22) | -0.22  (-0.29 to -0.14) | <0.001 |
|  | Cook Islands | 10355.16  (5920.83 to 16748.55) | 10330.14  (5894.07 to 16511.28) | -0.02  (-0.06 to 0.03) | 0.451 | 2211.97  (1085.48 to 3977.50) | 2145.98  (1067.71 to 3847.85) | -0.08  (-0.13 to -0.03) | 0.001 |
|  | Costa Rica | 8305.07  (4674.44 to 13434.40) | 8339.09  (4590.26 to 13737.09) | 0.01  (-0.01 to 0.03) | 0.189 | 1291.25  (619.74 to 2319.73) | 1184.32  (591.38 to 2124.64) | -0.28  (-0.33 to -0.24) | <0.001 |
|  | Coted'Ivoire | 4738.25  (2625.65 to 7903.32) | 4666.03  (2579.25 to 7631.80) | -0.08  (-0.15 to 0) | 0.038 | 2659.62  (1320.66 to 4769.53) | 2381.40  (1170.02 to 4318.99) | -0.38  (-0.47 to -0.29) | <0.001 |
|  | Croatia | 5611.50  (3116.08 to 9281.67) | 5641.77  (3136.46 to 9425.83) | 0.01  (-0.04 to 0.06) | 0.718 | 3943.98  (2011.56 to 7112.08) | 3644.02  (1832.48 to 6564.09) | -0.25  (-0.29 to -0.21) | <0.001 |
|  | Cuba | 9697.03  (5543.31 to 15792.85) | 9652.17  (5466.32 to 15742.19) | 0  (-0.02 to 0.01) | 0.344 | 1560.76  (768.92 to 2866.83) | 1465.64  (718.96 to 2666.96) | -0.2  (-0.21 to -0.18) | <0.001 |
|  | Cyprus | 537.93  (285.99 to 900.28) | 537.00  (287.67 to 905.03) | 0  (-0.02 to 0.01) | 0.862 | 141.06  (68.00 to 263.80) | 132.92  (64.16 to 246.37) | -0.2  (-0.27 to -0.14) | <0.001 |
|  | Czechia | 5209.11  (2872.68 to 8666.56) | 5187.58  (2880.20 to 8571.34) | -0.01  (-0.05 to 0.03) | 0.563 | 4070.76  (2043.75 to 7292.10) | 3697.59  (1876.19 to 6669.52) | -0.32  (-0.34 to -0.3) | <0.001 |
|  | Democratic People's Republic of Korea | 9081.68  (5097.76 to 14683.67) | 8914.43  (5067.22 to 14526.99) | -0.06  (-0.07 to -0.05) | <0.001 | 1361.09  (665.16 to 2458.36) | 1321.92  (665.20 to 2420.35) | -0.09  (-0.14 to -0.03) | 0.001 |
|  | Democratic Republic of the Congo | 2728.68  (1510.48 to 4533.98) | 2675.68  (1491.98 to 4473.87) | -0.07  (-0.08 to -0.06) | <0.001 | 947.53  (469.68 to 1719.10) | 888.84  (431.08 to 1613.82) | -0.19  (-0.28 to -0.09) | <0.001 |
|  | Denmark | 706.71  (377.83 to 1182.98) | 697.07  (373.54 to 1176.32) | -0.04  (-0.16 to 0.08) | 0.517 | 138.07  (66.79 to 256.37) | 127.62  (60.92 to 238.76) | -0.27  (-0.39 to -0.14) | <0.001 |
|  | Djibouti | 4990.83  (2777.20 to 8145.79) | 4916.99  (2729.79 to 8073.45) | -0.05  (-0.06 to -0.04) | <0.001 | 2423.63  (1191.14 to 4401.01) | 2330.71  (1162.62 to 4209.38) | -0.13  (-0.15 to -0.12) | <0.001 |
|  | Dominica | 9724.27  (5582.41 to 15798.40) | 9757.35  (5518.84 to 15830.04) | 0.02  (-0.01 to 0.05) | 0.278 | 1748.55  (871.65 to 3158.19) | 1636.17  (822.25 to 2984.43) | -0.22  (-0.29 to -0.15) | <0.001 |
|  | Dominican Republic | 9812.15  (5549.06 to 15869.06) | 9723.06  (5527.18 to 15758.75) | -0.02  (-0.06 to 0.02) | 0.287 | 1801.40  (903.43 to 3172.54) | 1652.57  (834.34 to 2974.96) | -0.31  (-0.45 to -0.17) | <0.001 |
|  | Ecuador | 3561.80  (1983.34 to 5870.93) | 3510.00  (1927.41 to 5860.22) | 0.04  (-0.27 to 0.36) | 0.783 | 238.06  (114.63 to 439.51) | 216.94  (104.72 to 400.81) | -0.31  (-0.33 to -0.28) | <0.001 |
|  | Egypt | 6369.84  (3581.27 to 10284.65) | 5590.66  (3099.93 to 9125.34) | -0.41  (-0.55 to -0.28) | <0.001 | 2490.60  (1255.23 to 4499.29) | 2324.87  (1161.28 to 4221.35) | -0.25  (-0.46 to -0.04) | 0.018 |
|  | El Salvador | 8499.60  (4722.36 to 13721.67) | 8333.35  (4673.76 to 13567.91) | -0.06  (-0.08 to -0.05) | <0.001 | 1299.80  (635.46 to 2339.08) | 1180.02  (587.57 to 2157.11) | -0.32  (-0.38 to -0.26) | <0.001 |
|  | Equatorial Guinea | 3147.81  (1727.27 to 5207.19) | 3057.76  (1678.63 to 5096.48) | -0.09  (-0.1 to -0.08) | <0.001 | 1454.18  (714.27 to 2652.85) | 1287.39  (633.57 to 2366.60) | -0.38  (-0.47 to -0.3) | <0.001 |
|  | Eritrea | 4983.27  (2806.82 to 8192.03) | 4864.32  (2713.68 to 7941.23) | -0.07  (-0.09 to -0.05) | <0.001 | 2562.38  (1284.65 to 4620.98) | 2395.01  (1196.15 to 4359.49) | -0.24  (-0.3 to -0.19) | <0.001 |
|  | Estonia | 7761.29  (4398.95 to 12667.98) | 7860.33  (4361.65 to 12971.42) | 0.03  (0.01 to 0.06) | 0.005 | 4573.52  (2288.13 to 8156.08) | 3577.79  (1779.45 to 6480.94) | -0.83  (-0.87 to -0.78) | <0.001 |
|  | Eswatini | 7491.67  (4207.72 to 12052.42) | 7394.40  (4115.83 to 12160.48) | -0.02  (-0.04 to -0.01) | 0.007 | 4210.69  (2130.48 to 7435.89) | 3877.83  (1967.06 to 6990.18) | -0.26  (-0.29 to -0.23) | <0.001 |
|  | Ethiopia | 5729.46  (3276.94 to 9205.93) | 5315.74  (3006.46 to 8710.35) | -0.24  (-0.26 to -0.22) | <0.001 | 1444.42  (665.39 to 2693.99) | 1262.16  (583.26 to 2324.46) | -0.5  (-0.68 to -0.32) | <0.001 |
|  | Fiji | 21527.84  (13489.33 to 31705.50) | 17317.70  (10149.13 to 27142.43) | -0.69  (-0.82 to -0.56) | <0.001 | 3113.57  (1685.57 to 5156.97) | 2717.20  (1342.62 to 4905.20) | -0.43  (-0.46 to -0.4) | <0.001 |
|  | Finland | 746.63  (402.65 to 1256.18) | 747.65  (399.84 to 1246.49) | 0.02  (-0.02 to 0.05) | 0.298 | 142.15  (68.67 to 262.90) | 123.78  (59.26 to 229.09) | -0.46  (-0.49 to -0.43) | <0.001 |
|  | France | 536.63  (287.03 to 892.98) | 533.80  (284.26 to 903.20) | -0.02  (-0.05 to 0.01) | 0.166 | 141.71  (68.52 to 263.31) | 128.04  (61.87 to 238.13) | -0.33  (-0.36 to -0.3) | <0.001 |
|  | Gabon | 3084.44  (1698.91 to 5141.80) | 3056.81  (1667.73 to 5113.18) | -0.02  (-0.04 to 0) | 0.037 | 1393.74  (685.03 to 2554.77) | 1282.94  (620.30 to 2345.30) | -0.25  (-0.35 to -0.15) | <0.001 |
|  | Gambia | 2773.40  (1510.18 to 4577.63) | 2716.38  (1488.68 to 4491.54) | -0.05  (-0.1 to 0) | 0.037 | 2081.93  (1040.58 to 3804.52) | 1961.36  (974.67 to 3602.02) | -0.19  (-0.21 to -0.17) | <0.001 |
|  | Georgia | 12937.22  (7374.93 to 20833.98) | 12907.03  (7401.77 to 20509.05) | 0  (-0.05 to 0.04) | 0.854 | 4808.58  (2434.20 to 8571.76) | 4268.72  (2169.30 to 7623.39) | -0.39  (-0.45 to -0.34) | <0.001 |
|  | Germany | 479.41  (254.76 to 804.12) | 477.88  (257.08 to 796.58) | 0.01  (-0.06 to 0.07) | 0.867 | 143.73  (68.18 to 265.03) | 129.94  (62.05 to 242.55) | -0.35  (-0.4 to -0.3) | <0.001 |
|  | Ghana | 4832.24  (2662.54 to 7925.60) | 4769.18  (2669.80 to 7817.44) | -0.03  (-0.11 to 0.05) | 0.425 | 4836.27  (2415.83 to 8524.69) | 4698.99  (2413.57 to 8325.70) | -0.09  (-0.26 to 0.07) | 0.274 |
|  | Greece | 695.00  (367.78 to 1165.48) | 694.18  (370.21 to 1160.63) | -0.01  (-0.04 to 0.02) | 0.456 | 134.76  (64.07 to 249.55) | 124.51  (59.73 to 229.78) | -0.28  (-0.38 to -0.17) | <0.001 |
|  | Greenland | 629.78  (339.13 to 1053.56) | 630.30  (337.33 to 1060.85) | 0  (0 to 0.01) | 0.023 | 155.65  (75.21 to 288.39) | 149.44  (72.58 to 280.69) | -0.13  (-0.21 to -0.06) | <0.001 |
|  | Grenada | 9768.12  (5581.90 to 15763.46) | 9724.66  (5535.04 to 15890.18) | 0  (-0.01 to 0.01) | 0.822 | 1793.44  (884.63 to 3222.98) | 1643.07  (820.07 to 2978.52) | -0.29  (-0.39 to -0.2) | <0.001 |
|  | Guam | 10361.25  (5737.19 to 16822.44) | 10244.92  (5823.92 to 16514.02) | -0.05  (-0.07 to -0.02) | 0.001 | 2245.78  (1115.74 to 4105.87) | 2113.00  (1046.20 to 3845.69) | -0.19  (-0.26 to -0.11) | <0.001 |
|  | Guatemala | 8114.34  (5271.54 to 12000.72) | 7887.90  (4469.48 to 12800.56) | -0.09  (-0.11 to -0.07) | <0.001 | 2247.47  (1127.82 to 4015.02) | 1849.24  (889.91 to 3413.17) | -0.65  (-0.71 to -0.59) | <0.001 |
|  | Guinea | 3698.89  (2057.70 to 6148.79) | 3633.62  (2023.93 to 5977.11) | -0.03  (-0.05 to -0.02) | <0.001 | 2152.34  (1070.18 to 3937.47) | 2017.22  (982.08 to 3643.60) | -0.2  (-0.22 to -0.18) | <0.001 |
|  | Guinea-Bissau | 3671.30  (2024.96 to 6060.47) | 3606.94  (1967.65 to 5915.23) | -0.05  (-0.07 to -0.03) | <0.001 | 2207.43  (1089.14 to 3999.34) | 2025.36  (1015.51 to 3669.90) | -0.29  (-0.3 to -0.28) | <0.001 |
|  | Guyana | 9852.66  (5594.55 to 15903.30) | 9796.18  (5603.28 to 15916.41) | -0.01  (-0.06 to 0.04) | 0.694 | 1798.22  (896.39 to 3223.08) | 1663.28  (814.86 to 3024.65) | -0.25  (-0.29 to -0.22) | <0.001 |
|  | Haiti | 10046.65  (5692.54 to 16552.66) | 9958.15  (5716.51 to 16088.58) | -0.01  (-0.05 to 0.03) | 0.656 | 1916.97  (949.61 to 3428.08) | 1771.91  (873.45 to 3239.76) | -0.29  (-0.37 to -0.2) | <0.001 |
|  | Honduras | 8526.56  (4823.10 to 13881.04) | 8379.10  (4678.97 to 13561.62) | -0.05  (-0.07 to -0.02) | 0.001 | 1376.77  (672.64 to 2524.60) | 1279.73  (642.20 to 2346.82) | -0.24  (-0.29 to -0.19) | <0.001 |
|  | Hungary | 5074.33  (2819.20 to 8333.35) | 5057.53  (2798.10 to 8372.60) | -0.01  (-0.04 to 0.01) | 0.267 | 4203.35  (2137.63 to 7591.29) | 3740.43  (1898.41 to 6776.76) | -0.38  (-0.4 to -0.35) | <0.001 |
|  | Iceland | 536.14  (286.48 to 907.33) | 533.88  (283.52 to 888.55) | -0.01  (-0.02 to 0) | 0.099 | 143.08  (69.57 to 261.09) | 126.97  (61.27 to 235.01) | -0.41  (-0.45 to -0.36) | <0.001 |
|  | India | 2236.46  (1237.75 to 3735.00) | 2222.37  (1227.86 to 3742.05) | -0.06  (-0.14 to 0.03) | 0.206 | 1103.87  (507.36 to 2083.76) | 974.48  (446.64 to 1837.40) | -0.4  (-0.5 to -0.3) | <0.001 |
|  | Indonesia | 6021.10  (3408.70 to 9871.89) | 5866.16  (3316.18 to 9617.23) | -0.08  (-0.09 to -0.07) | <0.001 | 737.03  (339.63 to 1360.19) | 684.66  (317.45 to 1269.63) | -0.23  (-0.25 to -0.22) | <0.001 |
|  | Iran (Islamic Republic of) | 8657.41  (4929.09 to 14007.56) | 7982.57  (4500.01 to 12920.20) | -0.29  (-0.53 to -0.04) | 0.023 | 1623.98  (743.42 to 3069.01) | 1600.60  (728.90 to 3025.88) | -0.06  (-0.19 to 0.07) | 0.351 |
|  | Iraq | 9210.23  (6067.77 to 13336.58) | 6850.19  (3850.88 to 11099.91) | -0.94  (-1.12 to -0.77) | <0.001 | 1563.01  (764.36 to 2877.17) | 1451.51  (731.39 to 2645.66) | -0.24  (-0.29 to -0.18) | <0.001 |
|  | Ireland | 757.25  (409.75 to 1262.62) | 755.11  (408.81 to 1269.27) | 0  (-0.06 to 0.07) | 0.961 | 138.79  (66.22 to 258.48) | 125.92  (60.96 to 233.17) | -0.33  (-0.39 to -0.27) | <0.001 |
|  | Israel | 1210.60  (676.98 to 1954.15) | 963.95  (522.44 to 1598.62) | -0.76  (-0.84 to -0.68) | <0.001 | 142.12  (68.06 to 264.60) | 128.73  (60.65 to 236.71) | -0.34  (-0.44 to -0.23) | <0.001 |
|  | Italy | 786.83  (427.82 to 1319.28) | 776.37  (420.95 to 1294.44) | 0.01  (-0.03 to 0.05) | 0.597 | 144.88  (68.16 to 271.19) | 131.12  (61.97 to 244.61) | -0.33  (-0.4 to -0.27) | <0.001 |
|  | Jamaica | 9750.92  (5480.70 to 15788.24) | 9733.75  (5479.06 to 15777.03) | 0  (-0.01 to 0.01) | 0.911 | 1680.90  (834.47 to 3022.45) | 1597.44  (804.28 to 2932.23) | -0.2  (-0.36 to -0.05) | 0.011 |
|  | Japan | 1411.54  (772.59 to 2361.31) | 1426.82  (783.99 to 2363.64) | 0  (-0.04 to 0.04) | 0.867 | 481.54  (223.36 to 893.04) | 422.15  (197.42 to 772.48) | -0.44  (-0.5 to -0.38) | <0.001 |
|  | Jordan | 1962.51  (1080.71 to 3277.78) | 1884.72  (1036.83 to 3145.58) | -0.12  (-0.19 to -0.05) | 0.001 | 1264.04  (627.59 to 2296.82) | 1251.13  (617.41 to 2294.89) | -0.1  (-0.35 to 0.16) | 0.449 |
|  | Kazakhstan | 12898.11  (7328.05 to 20686.19) | 12959.23  (7387.08 to 20840.38) | 0.02  (-0.03 to 0.07) | 0.501 | 4947.59  (2477.00 to 8981.98) | 4300.73  (2127.57 to 7754.99) | -0.46  (-0.5 to -0.42) | <0.001 |
|  | Kenya | 5082.75  (2858.29 to 8355.03) | 5120.16  (2877.90 to 8423.27) | 0  (-0.16 to 0.16) | 0.975 | 2398.51  (1120.93 to 4410.44) | 2351.21  (1110.99 to 4303.34) | -0.06  (-0.24 to 0.12) | 0.505 |
|  | Kiribati | 11270.45  (6415.41 to 18269.85) | 11148.01  (6384.20 to 17768.71) | -0.04  (-0.09 to 0.01) | 0.093 | 684.90  (319.07 to 1267.01) | 680.65  (316.97 to 1277.92) | -0.01  (-0.14 to 0.12) | 0.901 |
|  | Kuwait | 3457.66  (1887.99 to 5802.10) | 3373.62  (1863.67 to 5547.70) | -0.1  (-0.34 to 0.14) | 0.413 | 2273.94  (1126.38 to 4101.68) | 2053.76  (1016.50 to 3714.14) | -0.29  (-0.43 to -0.15) | <0.001 |
|  | Kyrgyzstan | 12898.67  (7458.27 to 20915.60) | 12979.04  (7420.70 to 20963.46) | 0.02  (0 to 0.04) | 0.021 | 4827.36  (2470.20 to 8558.68) | 4387.33  (2215.26 to 7808.73) | -0.32  (-0.36 to -0.28) | <0.001 |
|  | Lao People's Democratic Republic | 6319.46  (3544.79 to 10440.82) | 6122.29  (3466.28 to 10101.38) | -0.09  (-0.12 to -0.07) | <0.001 | 709.61  (341.33 to 1304.23) | 627.58  (306.42 to 1147.69) | -0.39  (-0.43 to -0.34) | <0.001 |
|  | Latvia | 7813.53  (4399.27 to 12810.53) | 7853.99  (4413.65 to 12942.07) | 0.02  (0 to 0.03) | 0.005 | 4491.69  (2264.85 to 8186.87) | 3719.61  (1862.27 to 6725.59) | -0.67  (-0.75 to -0.59) | <0.001 |
|  | Lebanon | 2943.90  (1615.32 to 4953.73) | 2610.62  (1419.24 to 4313.75) | -0.15  (-0.31 to 0.01) | 0.072 | 1474.30  (726.70 to 2705.33) | 1432.69  (713.60 to 2602.29) | -0.08  (-0.16 to 0) | 0.064 |
|  | Lesotho | 7542.81  (4214.77 to 12206.48) | 7451.36  (4124.86 to 12111.08) | -0.02  (-0.03 to -0.01) | <0.001 | 4213.60  (2104.54 to 7582.14) | 3962.95  (1973.71 to 7027.22) | -0.19  (-0.25 to -0.13) | <0.001 |
|  | Liberia | 3639.95  (2005.63 to 6068.16) | 3588.82  (1993.89 to 5900.69) | -0.04  (-0.07 to -0.01) | 0.006 | 2140.71  (1068.41 to 3908.77) | 1995.49  (972.56 to 3610.89) | -0.23  (-0.26 to -0.2) | <0.001 |
|  | Libya | 5352.30  (2954.39 to 8753.48) | 5114.73  (2843.99 to 8432.46) | -0.1  (-0.13 to -0.07) | <0.001 | 1506.78  (737.96 to 2799.86) | 1469.13  (724.37 to 2642.03) | -0.07  (-0.13 to -0.02) | 0.008 |
|  | Lithuania | 7775.98  (4387.10 to 12695.61) | 7855.39  (4408.51 to 12963.48) | 0.03  (0.02 to 0.04) | <0.001 | 4381.93  (2203.28 to 7762.17) | 3763.90  (1905.71 to 6796.28) | -0.52  (-0.55 to -0.49) | <0.001 |
|  | Luxembourg | 439.50  (233.48 to 733.62) | 440.41  (235.18 to 749.06) | 0  (-0.08 to 0.09) | 0.931 | 146.62  (71.33 to 269.98) | 134.18  (63.97 to 248.10) | -0.3  (-0.35 to -0.25) | <0.001 |
|  | Madagascar | 4919.47  (2726.42 to 8040.98) | 4896.96  (2689.37 to 8078.74) | -0.01  (-0.03 to 0) | 0.096 | 2443.97  (1228.87 to 4398.19) | 2404.65  (1204.92 to 4381.10) | -0.08  (-0.13 to -0.04) | <0.001 |
|  | Malawi | 5922.00  (3484.76 to 9307.88) | 5564.10  (3050.85 to 9132.28) | -0.2  (-0.28 to -0.12) | <0.001 | 4472.94  (2243.85 to 8021.43) | 4445.42  (2235.30 to 7804.32) | -0.07  (-0.15 to 0.01) | 0.086 |
|  | Malaysia | 6084.28  (3382.31 to 9929.23) | 5994.33  (3283.88 to 9795.24) | -0.04  (-0.08 to 0.01) | 0.097 | 646.85  (309.92 to 1176.78) | 594.77  (288.55 to 1101.81) | -0.26  (-0.31 to -0.22) | <0.001 |
|  | Maldives | 6130.01  (3440.31 to 10144.56) | 5979.11  (3370.94 to 9844.79) | -0.07  (-0.09 to -0.05) | <0.001 | 690.61  (336.48 to 1263.65) | 604.48  (292.19 to 1093.94) | -0.42  (-0.47 to -0.37) | <0.001 |
|  | Mali | 4160.08  (2311.61 to 6882.44) | 4091.09  (2264.41 to 6689.39) | -0.04  (-0.16 to 0.08) | 0.527 | 1925.06  (939.20 to 3483.81) | 1836.79  (924.35 to 3333.96) | -0.17  (-0.2 to -0.14) | <0.001 |
|  | Malta | 538.69  (290.33 to 904.70) | 535.50  (284.41 to 899.41) | -0.01  (-0.04 to 0.02) | 0.365 | 142.47  (68.41 to 266.62) | 129.82  (62.55 to 242.93) | -0.32  (-0.41 to -0.22) | <0.001 |
|  | Marshall Islands | 2254.54  (1235.21 to 3737.65) | 2232.92  (1209.11 to 3719.41) | 0.01  (-0.22 to 0.24) | 0.934 | 2409.08  (1181.46 to 4396.64) | 2309.16  (1151.54 to 4181.97) | -0.14  (-0.23 to -0.04) | 0.006 |
|  | Mauritania | 3707.12  (2033.10 to 6148.33) | 3604.99  (1997.62 to 5976.22) | -0.09  (-0.09 to -0.08) | <0.001 | 2194.12  (1085.37 to 3938.15) | 1981.66  (992.37 to 3548.21) | -0.32  (-0.35 to -0.29) | <0.001 |
|  | Mauritius | 6062.27  (3404.08 to 10006.37) | 5985.82  (3365.13 to 9852.82) | -0.04  (-0.05 to -0.03) | <0.001 | 643.38  (312.70 to 1171.85) | 560.51  (273.72 to 1031.36) | -0.44  (-0.47 to -0.41) | <0.001 |
|  | Mexico | 11165.45  (6458.27 to 17935.32) | 10971.37  (6336.07 to 17669.32) | -0.06  (-0.11 to -0.01) | 0.026 | 1396.72  (652.15 to 2591.74) | 1207.70  (568.22 to 2241.25) | -0.47  (-0.49 to -0.44) | <0.001 |
|  | Micronesia (Federated States of) | 10586.77  (5990.19 to 16879.79) | 10467.93  (5997.33 to 16878.52) | -0.04  (-0.08 to 0) | 0.028 | 2486.14  (1226.79 to 4460.38) | 2339.19  (1168.48 to 4334.43) | -0.21  (-0.25 to -0.16) | <0.001 |
|  | Monaco | 533.61  (287.55 to 889.81) | 531.52  (282.52 to 883.15) | -0.01  (-0.03 to 0.01) | 0.449 | 134.25  (65.16 to 249.58) | 128.58  (61.84 to 241.04) | -0.16  (-0.23 to -0.08) | <0.001 |
|  | Mongolia | 13078.51  (7512.34 to 20830.30) | 12994.59  (7475.62 to 20570.73) | -0.01  (-0.1 to 0.08) | 0.869 | 4865.84  (2438.21 to 8734.86) | 4471.13  (2242.78 to 7988.98) | -0.27  (-0.32 to -0.22) | <0.001 |
|  | Montenegro | 5110.30  (2827.89 to 8446.89) | 5068.73  (2820.28 to 8455.74) | -0.03  (-0.08 to 0.02) | 0.205 | 4160.28  (2094.36 to 7558.39) | 3931.00  (1976.00 to 7059.84) | -0.19  (-0.22 to -0.17) | <0.001 |
|  | Morocco | 7611.83  (4403.89 to 12168.42) | 5369.26  (2980.80 to 8855.91) | -1.05  (-1.38 to -0.72) | <0.001 | 1820.72  (905.86 to 3291.76) | 1701.77  (829.15 to 3093.35) | -0.23  (-0.33 to -0.13) | <0.001 |
|  | Mozambique | 5581.35  (3109.43 to 9215.00) | 5583.16  (3140.62 to 9029.68) | -0.01  (-0.09 to 0.07) | 0.786 | 3482.69  (1727.49 to 6166.87) | 3341.48  (1665.54 to 6020.89) | -0.15  (-0.2 to -0.11) | <0.001 |
|  | Myanmar | 6211.06  (3465.59 to 10051.06) | 6081.76  (3417.53 to 10059.89) | -0.05  (-0.08 to -0.02) | 0.001 | 703.79  (345.56 to 1283.43) | 631.41  (305.97 to 1147.88) | -0.34  (-0.4 to -0.28) | <0.001 |
|  | Namibia | 7521.80  (4177.73 to 12402.12) | 7393.36  (4074.62 to 12078.77) | -0.05  (-0.07 to -0.02) | <0.001 | 4192.82  (2148.82 to 7471.55) | 3773.85  (1910.09 to 6694.74) | -0.34  (-0.36 to -0.31) | <0.001 |
|  | Nauru | 10575.20  (6042.89 to 16989.41) | 10453.60  (5889.70 to 16891.10) | -0.05  (-0.07 to -0.02) | <0.001 | 2245.60  (1127.86 to 4100.38) | 2176.25  (1084.17 to 3940.72) | -0.11  (-0.19 to -0.04) | 0.004 |
|  | Nepal | 4100.83  (2260.77 to 6800.50) | 4026.22  (2213.59 to 6652.99) | -0.06  (-0.07 to -0.05) | <0.001 | 813.57  (395.97 to 1484.18) | 692.32  (337.29 to 1283.57) | -0.52  (-0.58 to -0.45) | <0.001 |
|  | Netherlands | 1086.13  (619.58 to 1771.67) | 788.68  (422.52 to 1320.42) | -1.02  (-1.12 to -0.91) | <0.001 | 120.50  (57.50 to 220.23) | 116.15  (53.74 to 218.16) | -0.14  (-0.25 to -0.02) | 0.016 |
|  | New Zealand | 4145.89  (2297.65 to 6801.44) | 4177.77  (2329.76 to 6814.52) | -0.01  (-0.12 to 0.1) | 0.842 | 224.09  (102.64 to 420.86) | 197.19  (92.86 to 370.23) | -0.44  (-0.54 to -0.34) | <0.001 |
|  | Nicaragua | 9189.75  (5525.20 to 14404.41) | 8661.49  (4867.06 to 14062.63) | -0.19  (-0.21 to -0.18) | <0.001 | 2329.30  (1119.42 to 4297.15) | 1964.06  (924.03 to 3680.81) | -0.56  (-0.62 to -0.51) | <0.001 |
|  | Niger | 3696.09  (1970.55 to 6233.92) | 3564.11  (1924.04 to 5978.11) | -0.09  (-0.12 to -0.05) | <0.001 | 3027.52  (1483.96 to 5353.32) | 2592.01  (1251.02 to 4698.53) | -0.49  (-0.53 to -0.46) | <0.001 |
|  | Nigeria | 5843.80  (3297.19 to 9562.96) | 6099.40  (3454.99 to 9972.35) | 0.14  (-0.04 to 0.32) | 0.132 | 1824.87  (865.68 to 3354.84) | 1718.21  (828.72 to 3139.53) | -0.21  (-0.24 to -0.17) | <0.001 |
|  | Niue | 10427.57  (5877.05 to 16947.64) | 10362.52  (5904.11 to 16842.02) | -0.02  (-0.04 to 0) | 0.055 | 2218.20  (1102.02 to 4075.38) | 2153.39  (1069.09 to 3953.34) | -0.09  (-0.14 to -0.03) | 0.002 |
|  | North Macedonia | 5078.75  (2810.93 to 8455.68) | 5069.28  (2821.26 to 8352.06) | -0.01  (-0.02 to 0) | 0.042 | 3976.39  (2005.33 to 7183.63) | 3809.68  (1923.09 to 6797.44) | -0.14  (-0.16 to -0.12) | <0.001 |
|  | Northern Mariana Islands | 10367.68  (5912.09 to 16813.41) | 10215.04  (5776.29 to 16439.09) | -0.05  (-0.06 to -0.03) | <0.001 | 2306.89  (1149.31 to 4114.03) | 2173.53  (1077.87 to 3952.43) | -0.2  (-0.28 to -0.12) | <0.001 |
|  | Norway | 906.83  (496.58 to 1506.01) | 895.70  (488.86 to 1490.77) | -0.03  (-0.06 to 0.01) | 0.208 | 131.41  (60.42 to 250.04) | 148.95  (69.18 to 279.31) | 0.41  (0.38 to 0.45) | <0.001 |
|  | Oman | 5289.29  (2912.10 to 8674.68) | 5085.71  (2826.00 to 8471.38) | -0.09  (-0.12 to -0.07) | <0.001 | 1510.19  (736.02 to 2776.71) | 1441.68  (711.59 to 2588.25) | -0.14  (-0.21 to -0.07) | <0.001 |
|  | Pakistan | 7506.40  (4230.23 to 12263.06) | 6938.50  (3895.88 to 11364.44) | -0.23  (-0.3 to -0.16) | <0.001 | 527.25  (254.78 to 968.93) | 458.54  (219.29 to 837.44) | -0.45  (-0.5 to -0.39) | <0.001 |
|  | Palau | 10430.37  (5892.77 to 16988.36) | 10344.01  (5888.81 to 16642.70) | -0.03  (-0.04 to -0.02) | <0.001 | 2227.68  (1115.01 to 3994.86) | 2161.77  (1070.96 to 3945.93) | -0.08  (-0.16 to -0.01) | 0.035 |
|  | Palestine | 5305.15  (2948.75 to 8732.18) | 5127.02  (2858.11 to 8467.82) | -0.08  (-0.1 to -0.06) | <0.001 | 1533.74  (752.96 to 2807.71) | 1489.63  (748.76 to 2706.15) | -0.09  (-0.14 to -0.05) | <0.001 |
|  | Panama | 8382.04  (4659.16 to 13382.33) | 8241.15  (4627.20 to 13232.44) | -0.05  (-0.06 to -0.04) | <0.001 | 1212.09  (589.74 to 2241.40) | 1167.97  (574.57 to 2116.68) | -0.12  (-0.13 to -0.11) | <0.001 |
|  | Papua New Guinea | 16087.49  (9647.38 to 24717.27) | 13624.74  (7864.64 to 21736.63) | -0.54  (-0.62 to -0.46) | <0.001 | 10586.43  (5403.26 to 18246.43) | 10495.59  (5402.89 to 18253.83) | -0.03  (-0.1 to 0.04) | 0.41 |
|  | Paraguay | 11358.27  (6495.95 to 18260.27) | 11341.50  (6413.09 to 18216.29) | -0.01  (-0.03 to 0.01) | 0.36 | 1719.42  (842.77 to 3126.04) | 1651.98  (822.92 to 2970.92) | -0.13  (-0.17 to -0.09) | <0.001 |
|  | Peru | 7100.23  (4023.34 to 11522.98) | 6907.08  (3833.34 to 11433.72) | -0.06  (-0.17 to 0.06) | 0.355 | 245.58  (119.06 to 452.58) | 224.46  (109.37 to 409.80) | -0.33  (-0.46 to -0.19) | <0.001 |
|  | Philippines | 6978.44  (3984.65 to 11429.85) | 6834.31  (3889.16 to 11186.58) | -0.05  (-0.1 to 0) | 0.042 | 621.19  (294.15 to 1130.92) | 602.69  (286.66 to 1091.79) | -0.09  (-0.14 to -0.05) | <0.001 |
|  | Poland | 5158.21  (2912.99 to 8541.01) | 5099.05  (2882.94 to 8489.60) | -0.04  (-0.08 to 0) | 0.052 | 3785.33  (1822.55 to 6877.28) | 3225.61  (1571.54 to 5807.34) | -0.51  (-0.59 to -0.42) | <0.001 |
|  | Portugal | 917.84  (491.20 to 1531.25) | 918.67  (493.82 to 1539.09) | -0.01  (-0.1 to 0.08) | 0.873 | 145.27  (69.81 to 268.77) | 133.31  (63.91 to 245.89) | -0.3  (-0.4 to -0.2) | <0.001 |
|  | Puerto Rico | 9593.03  (5464.75 to 15585.48) | 9635.40  (5513.85 to 15694.88) | 0.01  (0 to 0.03) | 0.145 | 1638.95  (803.57 to 2995.77) | 1544.64  (766.95 to 2837.50) | -0.19  (-0.25 to -0.12) | <0.001 |
|  | Qatar | 6182.56  (3454.19 to 10111.61) | 6003.77  (3355.82 to 9768.30) | -0.08  (-0.12 to -0.05) | <0.001 | 1484.99  (739.54 to 2688.31) | 1447.12  (711.53 to 2655.69) | -0.08  (-0.13 to -0.03) | 0.003 |
|  | Republic of Korea | 1208.68  (674.41 to 1943.78) | 1436.59  (786.91 to 2397.81) | 0.57  (0.51 to 0.62) | <0.001 | 441.27  (217.57 to 800.46) | 394.97  (194.81 to 725.47) | -0.38  (-0.46 to -0.31) | <0.001 |
|  | Republic of Moldova | 7854.84  (4397.26 to 12931.27) | 7906.45  (4420.02 to 12973.37) | 0.02  (0 to 0.04) | 0.028 | 4455.34  (2259.26 to 8004.29) | 3850.44  (1933.70 to 6910.00) | -0.49  (-0.55 to -0.43) | <0.001 |
|  | Romania | 5151.76  (2880.44 to 8517.11) | 5108.12  (2851.31 to 8429.25) | -0.02  (-0.04 to -0.01) | 0.004 | 4108.13  (2051.81 to 7416.09) | 3763.30  (1860.54 to 6768.29) | -0.28  (-0.34 to -0.23) | <0.001 |
|  | Russian Federation | 7522.07  (4316.89 to 12321.77) | 7560.66  (4322.93 to 12379.21) | 0.01  (-0.01 to 0.03) | 0.242 | 4313.40  (2025.63 to 7860.41) | 3930.16  (1840.17 to 7246.02) | -0.29  (-0.34 to -0.24) | <0.001 |
|  | Rwanda | 4430.78  (2443.55 to 7270.06) | 4297.16  (2384.04 to 7147.94) | -0.08  (-0.11 to -0.05) | <0.001 | 2503.28  (1255.22 to 4495.48) | 2335.21  (1158.28 to 4199.89) | -0.3  (-0.34 to -0.25) | <0.001 |
|  | Saint Kitts and Nevis | 9703.66  (5486.52 to 15772.31) | 9670.96  (5483.58 to 15573.75) | 0  (-0.02 to 0.03) | 0.84 | 1629.00  (809.35 to 2972.24) | 1542.72  (759.50 to 2832.79) | -0.17  (-0.18 to -0.16) | <0.001 |
|  | Saint Lucia | 9777.90  (5516.51 to 15945.57) | 9726.06  (5555.35 to 15805.40) | -0.01  (-0.06 to 0.04) | 0.685 | 1796.30  (903.44 to 3227.07) | 1632.62  (797.07 to 2973.84) | -0.31  (-0.38 to -0.24) | <0.001 |
|  | Saint Vincent and the Grenadines | 9764.63  (5547.83 to 15931.56) | 9720.39  (5482.08 to 15725.75) | -0.01  (-0.03 to 0) | 0.142 | 1803.72  (886.64 to 3216.16) | 1660.38  (821.18 to 3076.22) | -0.27  (-0.34 to -0.2) | <0.001 |
|  | Samoa | 16219.66  (9533.83 to 25490.29) | 16035.99  (9368.30 to 25245.07) | -0.03  (-0.42 to 0.35) | 0.867 | 3212.31  (1613.15 to 5729.21) | 3140.75  (1576.87 to 5657.15) | -0.07  (-0.1 to -0.03) | <0.001 |
|  | San Marino | 533.45  (286.18 to 889.82) | 530.07  (281.81 to 876.97) | -0.01  (-0.04 to 0.02) | 0.399 | 134.78  (64.63 to 248.37) | 128.23  (61.37 to 237.98) | -0.18  (-0.29 to -0.07) | 0.002 |
|  | Sao Tome and Principe | 3619.71  (1990.20 to 6024.23) | 3564.30  (1972.68 to 5905.94) | -0.04  (-0.06 to -0.02) | <0.001 | 2054.20  (1002.19 to 3705.92) | 1947.94  (990.41 to 3502.92) | -0.17  (-0.19 to -0.15) | <0.001 |
|  | Saudi Arabia | 14468.99  (8702.51 to 22345.67) | 11674.44  (6699.86 to 18498.03) | -0.63  (-0.67 to -0.59) | <0.001 | 711.22  (305.01 to 1398.80) | 718.16  (308.38 to 1401.27) | 0.01  (-0.21 to 0.23) | 0.919 |
|  | Senegal | 3264.05  (1890.19 to 5258.76) | 2244.12  (1227.82 to 3711.40) | -1.23  (-1.31 to -1.15) | <0.001 | 890.21  (472.57 to 1510.55) | 687.62  (347.29 to 1240.07) | -0.94  (-1.14 to -0.74) | <0.001 |
|  | Serbia | 5126.34  (2839.50 to 8494.55) | 5085.18  (2815.67 to 8426.49) | -0.02  (-0.04 to 0) | 0.039 | 4123.25  (2090.78 to 7292.49) | 3894.01  (1968.44 to 7000.54) | -0.19  (-0.21 to -0.17) | <0.001 |
|  | Seychelles | 5402.51  (3024.54 to 8918.11) | 5201.98  (2905.27 to 8559.62) | -0.09  (-0.18 to 0) | 0.05 | 672.35  (323.49 to 1238.60) | 635.92  (307.62 to 1166.45) | -0.18  (-0.22 to -0.15) | <0.001 |
|  | Sierra Leone | 3657.36  (2004.95 to 6098.79) | 3582.00  (2002.34 to 5831.98) | -0.06  (-0.07 to -0.04) | <0.001 | 2092.68  (1057.28 to 3754.47) | 1973.09  (974.04 to 3606.05) | -0.19  (-0.23 to -0.15) | <0.001 |
|  | Singapore | 1443.98  (784.45 to 2423.57) | 1439.58  (781.25 to 2392.97) | -0.01  (-0.03 to 0.01) | 0.259 | 444.15  (216.95 to 812.74) | 374.64  (186.08 to 688.27) | -0.57  (-0.71 to -0.43) | <0.001 |
|  | Slovakia | 5072.74  (2820.82 to 8347.07) | 5050.18  (2802.68 to 8309.26) | -0.02  (-0.04 to 0) | 0.013 | 4049.95  (2003.83 to 7273.08) | 3911.49  (1969.07 to 7058.35) | -0.15  (-0.21 to -0.09) | <0.001 |
|  | Slovenia | 3343.23  (1860.44 to 5589.42) | 3339.78  (1839.22 to 5546.95) | 0.01  (-0.06 to 0.09) | 0.75 | 3922.25  (1976.59 to 7018.26) | 3485.24  (1737.79 to 6365.37) | -0.41  (-0.45 to -0.36) | <0.001 |
|  | Solomon Islands | 8635.35  (4814.17 to 13917.25) | 8412.28  (4618.15 to 13750.36) | -0.11  (-0.15 to -0.06) | <0.001 | 1062.30  (524.24 to 1925.56) | 972.10  (481.59 to 1774.36) | -0.25  (-0.41 to -0.09) | 0.002 |
|  | Somalia | 5597.56  (3218.19 to 8967.40) | 3923.40  (2168.86 to 6574.31) | -1.19  (-1.26 to -1.11) | <0.001 | 1492.42  (745.05 to 2648.06) | 1505.89  (748.23 to 2730.46) | -0.03  (-0.24 to 0.18) | 0.784 |
|  | South Africa | 12768.20  (7424.68 to 20151.39) | 11790.36  (6803.62 to 18767.15) | -0.25  (-0.33 to -0.16) | <0.001 | 9399.22  (4935.80 to 15713.12) | 8861.12  (4458.40 to 15301.24) | -0.26  (-0.48 to -0.04) | 0.018 |
|  | South Sudan | 4979.10  (2766.01 to 8243.13) | 4950.84  (2750.73 to 8112.78) | -0.02  (-0.05 to 0.01) | 0.117 | 2591.86  (1294.94 to 4661.80) | 2509.68  (1260.67 to 4543.06) | -0.16  (-0.19 to -0.12) | <0.001 |
|  | Spain | 421.29  (228.10 to 705.29) | 438.46  (234.07 to 739.24) | 0.12  (0.08 to 0.15) | <0.001 | 285.73  (140.51 to 526.11) | 269.66  (127.69 to 506.61) | -0.17  (-0.38 to 0.04) | 0.109 |
|  | Sri Lanka | 6099.73  (3381.57 to 10128.98) | 5984.97  (3332.08 to 9881.09) | -0.06  (-0.07 to -0.04) | <0.001 | 1114.53  (559.95 to 1975.06) | 1252.62  (611.81 to 2273.42) | 0.38  (0.28 to 0.48) | <0.001 |
|  | Sudan | 8415.00  (4731.46 to 13609.43) | 8801.61  (5222.44 to 13760.03) | 0.08  (-0.28 to 0.44) | 0.661 | 2655.80  (1331.01 to 4791.42) | 2517.83  (1260.19 to 4553.66) | -0.15  (-0.22 to -0.08) | <0.001 |
|  | Suriname | 9915.14  (5664.68 to 15906.10) | 9835.30  (5574.80 to 16044.42) | -0.02  (-0.05 to 0.01) | 0.29 | 1765.60  (870.69 to 3132.70) | 1607.88  (795.39 to 2928.03) | -0.31  (-0.38 to -0.24) | <0.001 |
|  | Sweden | 1697.99  (923.76 to 2865.79) | 1642.51  (905.22 to 2705.83) | -0.12  (-0.2 to -0.04) | 0.002 | 149.71  (66.83 to 284.09) | 126.21  (58.18 to 237.81) | -0.56  (-0.61 to -0.5) | <0.001 |
|  | Switzerland | 489.07  (261.53 to 813.12) | 486.09  (258.82 to 814.10) | -0.03  (-0.07 to 0.01) | 0.108 | 138.94  (66.23 to 260.37) | 129.21  (61.65 to 239.06) | -0.25  (-0.34 to -0.16) | <0.001 |
|  | Syrian Arab Republic | 5374.85  (2992.64 to 8843.11) | 5193.80  (2911.84 to 8497.67) | -0.07  (-0.1 to -0.03) | <0.001 | 1540.17  (761.62 to 2799.88) | 1452.77  (715.52 to 2625.68) | -0.21  (-0.27 to -0.14) | <0.001 |
|  | Taiwan (Province of China) | 12598.02  (7530.35 to 19589.99) | 11123.27  (6296.49 to 18030.04) | -0.4  (-0.42 to -0.38) | <0.001 | 1321.09  (649.24 to 2416.12) | 1270.17  (636.49 to 2302.60) | -0.14  (-0.17 to -0.1) | <0.001 |
|  | Tajikistan | 13146.47  (7427.62 to 21156.42) | 13107.51  (7524.29 to 20828.23) | -0.01  (-0.06 to 0.04) | 0.634 | 5348.61  (2678.02 to 9530.47) | 4948.30  (2468.85 to 8800.60) | -0.26  (-0.3 to -0.21) | <0.001 |
|  | Thailand | 8736.31  (5239.20 to 13577.68) | 7880.07  (4414.21 to 12893.72) | -0.31  (-0.36 to -0.26) | <0.001 | 657.51  (327.23 to 1173.13) | 708.98  (341.42 to 1288.83) | 0.24  (0.2 to 0.29) | <0.001 |
|  | Timor-Leste | 6305.68  (3520.49 to 10397.78) | 6113.45  (3435.20 to 9990.37) | -0.1  (-0.11 to -0.08) | <0.001 | 693.88  (336.71 to 1262.98) | 628.36  (308.29 to 1144.56) | -0.33  (-0.36 to -0.3) | <0.001 |
|  | Togo | 3642.08  (1995.33 to 6074.23) | 3614.49  (2030.55 to 5992.26) | -0.03  (-0.05 to 0) | 0.068 | 2128.98  (1072.33 to 3848.95) | 1967.69  (966.62 to 3562.79) | -0.25  (-0.27 to -0.23) | <0.001 |
|  | Tokelau | 10476.64  (5897.20 to 17067.72) | 10415.52  (5867.08 to 16846.40) | -0.04  (-0.05 to -0.03) | <0.001 | 2285.87  (1117.88 to 4167.89) | 2188.67  (1091.58 to 3962.69) | -0.13  (-0.19 to -0.07) | <0.001 |
|  | Tonga | 12315.42  (7064.93 to 19793.10) | 12264.58  (6980.41 to 19262.69) | -0.01  (-0.05 to 0.03) | 0.582 | 3018.97  (1503.24 to 5403.01) | 2863.75  (1437.07 to 5208.03) | -0.17  (-0.24 to -0.1) | <0.001 |
|  | Trinidad and Tobago | 9783.73  (5511.48 to 15867.58) | 9682.28  (5500.54 to 15768.34) | -0.02  (-0.04 to 0.01) | 0.159 | 1603.26  (787.98 to 2900.93) | 1514.14  (764.91 to 2779.91) | -0.19  (-0.25 to -0.13) | <0.001 |
|  | Tunisia | 5356.05  (2941.02 to 8852.70) | 5124.14  (2842.70 to 8479.63) | -0.13  (-0.26 to 0.01) | 0.066 | 3250.63  (1799.12 to 5233.84) | 2389.12  (1186.37 to 4319.04) | -0.98  (-1.13 to -0.83) | <0.001 |
|  | Turkmenistan | 12977.40  (7407.15 to 20974.65) | 12990.13  (7465.44 to 20809.64) | 0  (-0.07 to 0.06) | 0.946 | 5094.12  (2587.54 to 9089.67) | 4055.78  (2067.92 to 7280.27) | -0.74  (-0.84 to -0.65) | <0.001 |
|  | Tuvalu | 10467.18  (5970.92 to 16857.64) | 10398.43  (5890.56 to 16788.94) | -0.02  (-0.05 to 0) | 0.058 | 2299.77  (1135.84 to 4151.81) | 2206.95  (1093.11 to 3980.02) | -0.12  (-0.19 to -0.05) | 0.001 |
|  | Türkiye | 7162.77  (4409.07 to 10898.74) | 5425.86  (3032.93 to 8878.06) | -0.9 (-1.01 to -0.79) | <0.001 | 4996.41  (2753.85 to 8280.00) | 3545.37  (1765.32 to 6430.94) | -1.06 (-1.12 to -1.01) | <0.001 |
|  | Uganda | 7069.38  (4043.32 to 11397.24) | 5995.53  (3372.28 to 9771.50) | -0.54  (-0.6 to -0.48) | <0.001 | 3587.52  (1840.44 to 6227.13) | 3793.34  (1929.24 to 6767.75) | 0.13  (-0.05 to 0.3) | 0.15 |
|  | Ukraine | 7508.19  (4224.36 to 12261.41) | 7610.02  (4327.52 to 12411.90) | 0.04  (0 to 0.08) | 0.033 | 4573.90  (2175.47 to 8403.56) | 3858.28  (1819.99 to 7093.67) | -0.55  (-0.62 to -0.48) | <0.001 |
|  | United Arab Emirates | 4151.39  (2281.45 to 6805.11) | 4072.43  (2234.46 to 6667.69) | -0.06  (-0.12 to -0.01) | 0.028 | 1490.77  (729.68 to 2704.69) | 1444.96  (716.01 to 2643.36) | -0.11  (-0.22 to -0.01) | 0.039 |
|  | United Kingdom | 457.78  (245.72 to 766.93) | 462.40  (248.22 to 775.02) | 0.03  (-0.01 to 0.07) | 0.16 | 111.59  (60.08 to 191.77) | 103.05  (54.79 to 181.08) | -0.27  (-0.33 to -0.22) | <0.001 |
|  | United Republic of Tanzania | 6014.77  (3621.90 to 9217.14) | 5145.36  (2843.62 to 8532.99) | -0.59  (-0.96 to -0.23) | 0.001 | 2311.56  (1146.37 to 4239.58) | 1888.52  (932.19 to 3460.11) | -0.76  (-1.28 to -0.24) | 0.004 |
|  | United States of America | 845.56  (453.70 to 1396.76) | 1032.59  (561.43 to 1704.65) | 0.63  (0.44 to 0.81) | <0.001 | 292.08  (136.21 to 543.70) | 281.77  (130.95 to 525.21) | -0.12  (-0.57 to 0.33) | 0.588 |
|  | United States Virgin Islands | 9700.88  (5545.36 to 15669.14) | 9651.96  (5496.81 to 15660.33) | 0  (-0.01 to 0) | 0.286 | 1772.95  (874.41 to 3253.13) | 1648.11  (812.57 to 2961.94) | -0.23  (-0.32 to -0.15) | <0.001 |
|  | Uruguay | 1345.66  (736.58 to 2260.16) | 1326.26  (727.02 to 2200.98) | -0.05  (-0.06 to -0.03) | <0.001 | 467.85  (230.63 to 864.15) | 426.73  (207.57 to 780.51) | -0.32  (-0.37 to -0.26) | <0.001 |
|  | Uzbekistan | 12919.78  (7398.22 to 20607.08) | 12902.97  (7385.09 to 20543.05) | 0  (-0.04 to 0.04) | 0.86 | 5134.54  (2590.28 to 9257.18) | 4270.57  (2159.90 to 7670.81) | -0.6  (-0.64 to -0.56) | <0.001 |
|  | Vanuatu | 15372.41  (9412.97 to 23353.80) | 13082.34  (7541.39 to 20838.94) | -0.54  (-0.58 to -0.5) | <0.001 | 3195.11  (1582.84 to 5735.94) | 3061.89  (1529.88 to 5587.62) | -0.13  (-0.19 to -0.06) | <0.001 |
|  | Venezuela (Bolivarian Republic of) | 8427.62  (4747.08 to 13635.08) | 8322.04  (4705.92 to 13457.44) | -0.03  (-0.06 to -0.01) | 0.02 | 1291.07  (628.37 to 2377.56) | 1203.22  (591.32 to 2187.50) | -0.24  (-0.27 to -0.2) | <0.001 |
|  | Viet Nam | 4106.01  (2455.48 to 6460.17) | 4660.72  (2594.62 to 7810.00) | 0.41  (0.37 to 0.45) | <0.001 | 235.37  (118.12 to 411.89) | 250.07  (123.55 to 451.18) | 0.19  (0.06 to 0.31) | 0.003 |
|  | Yemen | 5473.36  (3025.24 to 9009.11) | 5218.55  (2897.73 to 8556.88) | -0.13  (-0.16 to -0.11) | <0.001 | 1620.69  (792.10 to 2963.71) | 1567.81  (776.22 to 2863.63) | -0.08  (-0.18 to 0.01) | 0.083 |
|  | Zambia | 3668.04  (2019.82 to 6071.98) | 3672.29  (2026.31 to 6061.02) | 0.01  (-0.02 to 0.05) | 0.448 | 2678.78  (1356.35 to 4848.32) | 2611.00  (1307.97 to 4701.88) | -0.14  (-0.3 to 0.03) | 0.11 |
|  | Zimbabwe | 4809.17  (2637.65 to 7891.89) | 4764.86  (2614.15 to 7886.72) | -0.02  (-0.04 to 0) | 0.025 | 2260.44  (1138.42 to 4078.78) | 2172.09  (1090.90 to 3926.43) | -0.13  (-0.22 to -0.04) | 0.006 |
| Age-standardized Prevalence | Afghanistan | 4746.43  (2705.26 to 7565.48) | 4609.42  (2622.31 to 7475.97) | -0.09  (-0.12 to -0.05) | <0.001 | 1141.13  (593.75 to 2019.27) | 1121.10  (578.45 to 2016.99) | -0.04  (-0.11 to 0.02) | 0.189 |
|  | Albania | 4584.11  (2656.99 to 7375.19) | 4480.08  (2606.34 to 7272.90) | -0.08  (-0.14 to -0.01) | 0.024 | 2877.11  (1473.79 to 5096.58) | 2637.49  (1345.78 to 4698.86) | -0.28  (-0.32 to -0.25) | <0.001 |
|  | Algeria | 5733.15  (3376.34 to 9132.91) | 4341.82  (2478.88 to 6983.54) | -0.85  (-0.92 to -0.78) | <0.001 | 267.57  (149.98 to 443.71) | 259.60  (149.51 to 430.77) | -0.11  (-0.2 to -0.03) | 0.007 |
|  | American Samoa | 8972.81  (5118.80 to 14379.74) | 8849.67  (5101.68 to 14399.05) | -0.05  (-0.06 to -0.04) | <0.001 | 1619.21  (812.92 to 2880.55) | 1508.89  (780.60 to 2703.50) | -0.22  (-0.33 to -0.11) | <0.001 |
|  | Andorra | 529.06  (317.29 to 826.68) | 519.67  (310.10 to 823.90) | -0.06  (-0.1 to -0.03) | 0.001 | 113.99  (64.68 to 194.00) | 107.31  (60.23 to 183.27) | -0.2  (-0.24 to -0.16) | <0.001 |
|  | Angola | 3130.43  (1929.12 to 4887.70) | 2959.08  (1778.95 to 4678.86) | -0.18  (-0.19 to -0.18) | <0.001 | 1163.72  (675.56 to 1953.45) | 1021.99  (568.65 to 1749.43) | -0.41  (-0.51 to -0.31) | <0.001 |
|  | Antigua and Barbuda | 8308.14  (4839.04 to 13205.78) | 8315.06  (4865.61 to 13372.47) | 0  (-0.03 to 0.04) | 0.827 | 1230.23  (648.50 to 2158.60) | 1131.47  (584.11 to 2002.30) | -0.28  (-0.33 to -0.22) | <0.001 |
|  | Argentina | 1361.63  (927.59 to 1983.11) | 1443.75  (930.70 to 2201.89) | 0.19  (0.17 to 0.2) | <0.001 | 452.54  (292.70 to 696.56) | 434.19  (275.95 to 678.99) | -0.16  (-0.23 to -0.08) | <0.001 |
|  | Armenia | 11063.68  (6474.09 to 17528.66) | 11079.68  (6471.24 to 17417.36) | 0.01  (-0.06 to 0.07) | 0.865 | 3540.00  (1815.76 to 6229.55) | 2870.32  (1486.29 to 5127.37) | -0.68  (-0.75 to -0.61) | <0.001 |
|  | Australia | 1419.22  (850.56 to 2132.80) | 1395.72  (822.64 to 2117.57) | -0.03  (-0.17 to 0.1) | 0.614 | 333.55  (170.46 to 539.57) | 287.42  (146.72 to 460.88) | -0.48  (-0.5 to -0.46) | <0.001 |
|  | Austria | 529.44  (318.37 to 833.71) | 523.38  (314.15 to 828.96) | -0.04  (-0.05 to -0.02) | <0.001 | 113.84  (64.46 to 191.88) | 100.38  (56.77 to 172.73) | -0.41  (-0.48 to -0.35) | <0.001 |
|  | Azerbaijan | 11314.66  (6633.79 to 17754.74) | 11210.82  (6531.36 to 17742.10) | -0.03  (-0.05 to -0.01) | 0.006 | 3584.33  (1895.82 to 6310.60) | 3045.41  (1596.33 to 5361.91) | -0.53  (-0.56 to -0.49) | <0.001 |
|  | Bahamas | 8363.15  (4835.86 to 13442.12) | 8320.57  (4724.02 to 13461.75) | -0.01  (-0.02 to 0) | 0.003 | 1198.73  (633.78 to 2106.97) | 1107.60  (582.82 to 1983.46) | -0.26  (-0.29 to -0.23) | <0.001 |
|  | Bahrain | 4569.11  (2585.75 to 7377.62) | 4385.59  (2519.11 to 7070.76) | -0.09  (-0.12 to -0.07) | <0.001 | 1058.57  (551.15 to 1898.84) | 1028.84  (527.99 to 1830.47) | -0.08  (-0.15 to -0.02) | 0.009 |
|  | Bangladesh | 3907.76  (2393.54 to 6129.17) | 3697.17  (2177.08 to 5852.99) | -0.18  (-0.21 to -0.16) | <0.001 | 781.38  (451.93 to 1305.55) | 572.41  (319.87 to 988.11) | -1  (-1.04 to -0.95) | <0.001 |
|  | Barbados | 8361.94  (4870.72 to 13475.77) | 8310.24  (4848.24 to 13201.74) | -0.01  (-0.06 to 0.04) | 0.602 | 1229.97  (647.58 to 2139.73) | 1109.80  (580.99 to 1959.46) | -0.34  (-0.4 to -0.28) | <0.001 |
|  | Belarus | 6755.95  (3905.67 to 10938.54) | 6803.73  (3961.50 to 11014.13) | 0.03  (0.02 to 0.03) | <0.001 | 2992.35  (1545.64 to 5263.77) | 2482.05  (1269.11 to 4393.03) | -0.64  (-0.74 to -0.54) | <0.001 |
|  | Belgium | 847.81  (492.29 to 1343.95) | 841.01  (492.20 to 1357.01) | -0.04  (-0.29 to 0.21) | 0.757 | 118.70  (67.96 to 200.21) | 100.54  (57.41 to 172.77) | -0.55  (-0.59 to -0.51) | <0.001 |
|  | Belize | 8384.81  (4859.21 to 13462.33) | 8355.39  (4914.14 to 13419.64) | 0  (-0.02 to 0.01) | 0.656 | 1134.13  (597.19 to 2044.92) | 1068.53  (556.44 to 1895.90) | -0.2  (-0.23 to -0.16) | <0.001 |
|  | Benin | 2892.08  (1907.05 to 4273.20) | 2777.48  (1830.31 to 4157.16) | -0.1  (-0.17 to -0.03) | 0.004 | 1262.77  (716.16 to 2110.91) | 1171.92  (662.25 to 1996.13) | -0.24  (-0.31 to -0.18) | <0.001 |
|  | Bermuda | 8271.23  (4789.09 to 13341.00) | 8247.39  (4816.55 to 13267.84) | -0.01  (-0.01 to 0) | 0.032 | 1158.50  (603.55 to 2032.59) | 1049.20  (544.62 to 1868.18) | -0.31  (-0.37 to -0.26) | <0.001 |
|  | Bhutan | 3747.91  (2250.35 to 5984.74) | 3665.30  (2195.79 to 5753.11) | -0.08  (-0.09 to -0.07) | <0.001 | 621.81  (347.12 to 1062.26) | 536.46  (299.94 to 920.37) | -0.47  (-0.52 to -0.42) | <0.001 |
|  | Bolivia (Plurinational State of) | 4598.17  (2742.77 to 7212.90) | 4533.43  (2640.90 to 7162.57) | 0  (-0.18 to 0.19) | 0.97 | 291.72  (187.52 to 436.91) | 255.65  (146.37 to 398.97) | -0.41  (-0.49 to -0.33) | <0.001 |
|  | Bosnia and Herzegovina | 4526.92  (2640.25 to 7284.24) | 4498.97  (2606.57 to 7307.74) | -0.02  (-0.03 to -0.01) | <0.001 | 2773.10  (1432.58 to 4927.27) | 2574.80  (1300.93 to 4643.95) | -0.25  (-0.29 to -0.21) | <0.001 |
|  | Botswana | 7171.29  (4244.34 to 11271.02) | 7099.22  (4145.45 to 11280.67) | -0.02  (-0.12 to 0.09) | 0.766 | 3131.82  (1692.77 to 5409.96) | 2891.38  (1538.81 to 5000.87) | -0.26  (-0.34 to -0.18) | <0.001 |
|  | Brazil | 8839.94  (5118.87 to 14421.43) | 9184.12  (5429.61 to 14672.65) | 0.16  (-0.06 to 0.38) | 0.165 | 1187.53  (572.62 to 2176.34) | 1193.71  (606.40 to 2128.76) | 0.02  (-0.14 to 0.19) | 0.791 |
|  | Brunei Darussalam | 1609.65  (1001.45 to 2468.42) | 1595.15  (974.65 to 2436.04) | -0.03  (-0.04 to -0.02) | <0.001 | 496.14  (279.98 to 791.78) | 439.03  (240.75 to 701.30) | -0.4  (-0.42 to -0.37) | <0.001 |
|  | Bulgaria | 5770.46  (3329.27 to 9285.47) | 5799.72  (3319.39 to 9314.11) | 0  (-0.04 to 0.04) | 0.926 | 2709.79  (1396.55 to 4864.90) | 2521.18  (1290.21 to 4556.62) | -0.23  (-0.26 to -0.21) | <0.001 |
|  | Burkina Faso | 4219.07  (2794.75 to 6234.46) | 3703.93  (2318.46 to 5675.43) | -0.42  (-0.45 to -0.39) | <0.001 | 2043.14  (1135.25 to 3496.70) | 1717.07  (910.67 to 3041.92) | -0.59  (-0.74 to -0.43) | <0.001 |
|  | Burundi | 4538.46  (2682.23 to 7088.74) | 4429.50  (2592.71 to 7033.13) | -0.08  (-0.08 to -0.07) | <0.001 | 1814.01  (976.82 to 3166.03) | 1706.57  (893.50 to 2959.89) | -0.24  (-0.27 to -0.21) | <0.001 |
|  | Cabo Verde | 3710.52  (2318.21 to 5666.15) | 3699.60  (2357.71 to 5630.40) | 0  (-0.01 to 0.01) | 0.611 | 1443.19  (794.68 to 2463.55) | 1397.22  (783.09 to 2397.46) | -0.11  (-0.12 to -0.09) | <0.001 |
|  | Cambodia | 5340.37  (3023.12 to 8656.44) | 5216.39  (2947.04 to 8532.69) | -0.08  (-0.1 to -0.05) | <0.001 | 482.37  (242.89 to 862.63) | 418.54  (210.01 to 752.57) | -0.45  (-0.5 to -0.39) | <0.001 |
|  | Cameroon | 4103.16  (2716.35 to 6056.15) | 3616.03  (2314.29 to 5518.89) | -0.4  (-0.43 to -0.36) | <0.001 | 2224.91  (1254.68 to 3746.24) | 1989.88  (1083.62 to 3451.66) | -0.4  (-0.52 to -0.29) | <0.001 |
|  | Canada | 588.90  (370.64 to 880.13) | 582.15  (372.77 to 862.36) | -0.02  (-0.13 to 0.09) | 0.689 | 206.95  (126.10 to 308.92) | 189.90  (113.35 to 279.26) | -0.27  (-0.31 to -0.22) | <0.001 |
|  | Central African Republic | 3438.07  (2069.85 to 5453.17) | 3376.90  (2041.81 to 5362.90) | -0.06  (-0.07 to -0.04) | <0.001 | 1512.43  (752.89 to 2761.53) | 1487.62  (741.13 to 2747.74) | -0.05  (-0.11 to 0.01) | 0.102 |
|  | Chad | 4014.73  (2592.31 to 6045.34) | 3728.42  (2353.70 to 5722.96) | -0.24  (-0.26 to -0.22) | <0.001 | 1764.39  (997.16 to 2961.62) | 1547.38  (858.86 to 2648.59) | -0.44  (-0.46 to -0.41) | <0.001 |
|  | Chile | 1458.06  (934.83 to 2238.75) | 1431.80  (914.88 to 2164.57) | -0.05  (-0.07 to -0.03) | <0.001 | 463.88  (291.40 to 723.06) | 410.41  (260.36 to 645.64) | -0.4  (-0.5 to -0.31) | <0.001 |
|  | China | 5279.62  (3038.31 to 8643.39) | 5883.34  (3378.97 to 9628.97) | 0.51  (-0.42 to 1.45) | 0.283 | 1405.57  (663.09 to 2583.46) | 1171.96  (553.44 to 2142.27) | -0.58  (-0.77 to -0.39) | <0.001 |
|  | Colombia | 4767.77  (2960.55 to 7348.22) | 5331.07  (3059.90 to 8590.56) | 0.37  (0.3 to 0.44) | <0.001 | 405.98  (232.90 to 671.23) | 372.25  (206.13 to 631.82) | -0.27  (-0.29 to -0.25) | <0.001 |
|  | Comoros | 4462.73  (2612.02 to 7106.03) | 4415.03  (2589.45 to 7024.71) | -0.04  (-0.06 to -0.02) | 0.001 | 1764.98  (943.55 to 3128.77) | 1683.19  (910.13 to 2872.88) | -0.16  (-0.18 to -0.15) | <0.001 |
|  | Congo | 3033.11  (1857.11 to 4765.60) | 2948.99  (1792.52 to 4719.35) | -0.1  (-0.17 to -0.04) | 0.002 | 1100.98  (621.21 to 1872.52) | 1006.91  (569.46 to 1703.18) | -0.28  (-0.36 to -0.2) | <0.001 |
|  | Cook Islands | 8816.08  (5128.35 to 14133.36) | 8791.25  (5092.25 to 13940.93) | -0.02  (-0.06 to 0.02) | 0.416 | 1509.84  (758.25 to 2685.13) | 1462.35  (744.81 to 2596.21) | -0.09  (-0.13 to -0.04) | <0.001 |
|  | Costa Rica | 7143.23  (4110.02 to 11414.24) | 7168.52  (4048.12 to 11666.00) | 0.01  (-0.01 to 0.02) | 0.273 | 920.71  (472.30 to 1609.93) | 841.44  (446.29 to 1468.90) | -0.29  (-0.33 to -0.25) | <0.001 |
|  | Coted'Ivoire | 4925.30  (3151.17 to 7529.63) | 4586.40  (2833.17 to 7055.78) | -0.24  (-0.32 to -0.15) | <0.001 | 2055.10  (1148.88 to 3466.96) | 1765.45  (954.02 to 3055.88) | -0.52  (-0.59 to -0.44) | <0.001 |
|  | Croatia | 4937.41  (2859.49 to 7997.43) | 5004.39  (2920.56 to 8163.13) | 0.04  (0.02 to 0.06) | <0.001 | 2685.55  (1398.16 to 4799.05) | 2485.22  (1278.30 to 4431.44) | -0.25  (-0.29 to -0.2) | <0.001 |
|  | Cuba | 8314.48  (4853.52 to 13378.12) | 8285.85  (4796.55 to 13359.59) | 0  (-0.02 to 0.02) | 0.736 | 1101.53  (573.58 to 1972.48) | 1032.75  (534.49 to 1831.92) | -0.2  (-0.22 to -0.19) | <0.001 |
|  | Cyprus | 511.81  (299.35 to 814.00) | 503.27  (291.57 to 806.99) | -0.05  (-0.07 to -0.03) | <0.001 | 111.42  (61.58 to 194.53) | 102.71  (56.27 to 178.54) | -0.28  (-0.4 to -0.16) | <0.001 |
|  | Czechia | 4590.73  (2652.29 to 7469.74) | 4579.34  (2648.31 to 7392.26) | -0.01  (-0.04 to 0.03) | 0.632 | 2771.62  (1421.34 to 4919.27) | 2513.47  (1300.21 to 4496.01) | -0.32  (-0.35 to -0.3) | <0.001 |
|  | Democratic People's Republic of Korea | 7671.19  (4347.32 to 12343.74) | 7531.93  (4326.52 to 12212.78) | -0.06  (-0.07 to -0.05) | <0.001 | 929.49  (465.14 to 1660.65) | 902.82  (465.25 to 1635.06) | -0.09  (-0.14 to -0.03) | 0.001 |
|  | Democratic Republic of the Congo | 2706.56  (1690.00 to 4209.23) | 2620.02  (1631.52 to 4109.00) | -0.11  (-0.12 to -0.1) | <0.001 | 797.43  (465.00 to 1313.17) | 734.47  (426.15 to 1219.38) | -0.25  (-0.33 to -0.17) | <0.001 |
|  | Denmark | 659.90  (381.39 to 1055.12) | 653.26  (379.16 to 1051.27) | -0.04  (-0.13 to 0.05) | 0.367 | 109.75  (60.65 to 189.47) | 100.37  (54.94 to 174.57) | -0.29  (-0.42 to -0.16) | <0.001 |
|  | Djibouti | 4512.70  (2655.73 to 7126.32) | 4447.18  (2619.08 to 7071.67) | -0.04  (-0.06 to -0.03) | <0.001 | 1738.81  (916.43 to 3058.37) | 1667.40  (888.06 to 2921.03) | -0.14  (-0.16 to -0.13) | <0.001 |
|  | Dominica | 8331.76  (4878.95 to 13389.94) | 8358.27  (4824.13 to 13421.78) | 0.01  (-0.01 to 0.03) | 0.226 | 1239.53  (655.22 to 2180.92) | 1157.20  (614.03 to 2054.88) | -0.23  (-0.3 to -0.16) | <0.001 |
|  | Dominican Republic | 8412.38  (4874.41 to 13466.36) | 8328.70  (4833.02 to 13354.81) | -0.02  (-0.06 to 0.01) | 0.214 | 1283.90  (684.39 to 2198.38) | 1168.25  (623.82 to 2049.11) | -0.33  (-0.47 to -0.2) | <0.001 |
|  | Ecuador | 3319.56  (1980.68 to 5237.51) | 3327.27  (1965.57 to 5339.56) | 0.12  (-0.14 to 0.37) | 0.36 | 258.17  (159.48 to 400.72) | 240.66  (132.72 to 372.48) | -0.23  (-0.28 to -0.17) | <0.001 |
|  | Egypt | 5467.52  (3139.11 to 8725.08) | 4819.12  (2740.17 to 7765.80) | -0.4  (-0.53 to -0.27) | <0.001 | 1699.51  (875.08 to 3040.01) | 1588.49  (812.70 to 2851.93) | -0.25  (-0.45 to -0.04) | 0.019 |
|  | El Salvador | 7307.77  (4151.35 to 11658.16) | 7156.79  (4108.12 to 11526.23) | -0.07  (-0.08 to -0.05) | <0.001 | 925.66  (481.37 to 1619.82) | 834.41  (438.67 to 1487.25) | -0.35  (-0.4 to -0.3) | <0.001 |
|  | Equatorial Guinea | 3041.05  (1853.84 to 4751.71) | 2942.33  (1792.56 to 4639.46) | -0.1  (-0.11 to -0.1) | <0.001 | 1135.70  (634.68 to 1938.38) | 991.81  (556.01 to 1713.24) | -0.43  (-0.5 to -0.36) | <0.001 |
|  | Eritrea | 4520.89  (2698.87 to 7209.14) | 4396.82  (2598.18 to 6953.51) | -0.08  (-0.08 to -0.07) | <0.001 | 1853.42  (997.36 to 3225.11) | 1717.12  (917.48 to 3024.18) | -0.27  (-0.3 to -0.24) | <0.001 |
|  | Estonia | 6712.25  (3919.50 to 10806.13) | 6800.59  (3879.43 to 11055.41) | 0.04  (0.01 to 0.06) | 0.009 | 3122.85  (1597.57 to 5512.28) | 2433.49  (1235.99 to 4369.23) | -0.84  (-0.88 to -0.79) | <0.001 |
|  | Eswatini | 6669.36  (3929.88 to 10424.18) | 6581.21  (3837.08 to 10555.30) | -0.03  (-0.04 to -0.01) | <0.001 | 2968.55  (1576.22 to 5118.58) | 2725.50  (1452.38 to 4801.24) | -0.27  (-0.3 to -0.24) | <0.001 |
|  | Ethiopia | 5280.97  (3239.04 to 8187.89) | 4740.53  (2811.17 to 7567.83) | -0.35  (-0.38 to -0.32) | <0.001 | 1136.64  (603.59 to 1978.21) | 923.22  (469.49 to 1632.65) | -0.68  (-0.78 to -0.59) | <0.001 |
|  | Fiji | 18147.30  (11440.46 to 26627.35) | 14617.66  (8645.71 to 22791.91) | -0.69  (-0.82 to -0.56) | <0.001 | 2117.14  (1165.58 to 3484.22) | 1848.07  (932.25 to 3305.80) | -0.43  (-0.46 to -0.4) | <0.001 |
|  | Finland | 747.30  (454.42 to 1171.77) | 766.79  (478.91 to 1188.05) | 0.09  (0.02 to 0.16) | 0.018 | 147.70  (87.45 to 232.08) | 132.20  (85.89 to 203.75) | -0.38  (-0.44 to -0.31) | <0.001 |
|  | France | 531.32  (319.50 to 830.46) | 524.92  (314.13 to 834.00) | -0.04  (-0.07 to -0.02) | 0.001 | 116.07  (66.46 to 198.01) | 102.63  (57.89 to 175.83) | -0.4  (-0.43 to -0.37) | <0.001 |
|  | Gabon | 2974.75  (1807.39 to 4682.73) | 2942.49  (1786.53 to 4643.68) | -0.03  (-0.05 to -0.01) | <0.001 | 1078.28  (600.92 to 1849.64) | 987.82  (546.23 to 1695.80) | -0.27  (-0.35 to -0.19) | <0.001 |
|  | Gambia | 3043.94  (1958.68 to 4584.99) | 2970.70  (1947.31 to 4456.46) | -0.07  (-0.09 to -0.04) | <0.001 | 1591.77  (887.86 to 2737.86) | 1484.35  (824.99 to 2577.31) | -0.23  (-0.26 to -0.19) | <0.001 |
|  | Georgia | 11099.05  (6481.14 to 17688.16) | 11190.86  (6591.85 to 17506.22) | 0.03  (-0.01 to 0.07) | 0.161 | 3295.38  (1713.22 to 5801.80) | 2941.90  (1541.91 to 5178.67) | -0.37  (-0.43 to -0.31) | <0.001 |
|  | Germany | 476.91  (285.24 to 748.03) | 473.66  (285.23 to 740.29) | -0.02  (-0.06 to 0.03) | 0.411 | 116.50  (64.24 to 198.41) | 103.23  (57.26 to 178.49) | -0.41  (-0.45 to -0.37) | <0.001 |
|  | Ghana | 4749.27  (2928.72 to 7316.71) | 4665.86  (2911.89 to 7195.73) | -0.05  (-0.1 to 0.01) | 0.106 | 3434.81  (1816.30 to 5894.53) | 3321.13  (1796.71 to 5732.05) | -0.11  (-0.26 to 0.05) | 0.172 |
|  | Greece | 662.63  (386.64 to 1061.24) | 714.27  (441.40 to 1105.01) | 0.23  (0.14 to 0.32) | <0.001 | 109.66  (61.21 to 186.74) | 108.53  (64.26 to 179.73) | -0.05  (-0.1 to 0) | 0.069 |
|  | Greenland | 703.72  (441.71 to 1071.23) | 701.60  (442.54 to 1061.74) | -0.01  (-0.03 to 0) | 0.131 | 207.37  (124.94 to 310.54) | 196.56  (118.64 to 292.68) | -0.17  (-0.2 to -0.14) | <0.001 |
|  | Grenada | 8371.05  (4884.95 to 13365.96) | 8331.66  (4844.95 to 13461.87) | 0  (-0.01 to 0.01) | 0.696 | 1272.53  (666.53 to 2230.09) | 1161.01  (611.79 to 2050.50) | -0.31  (-0.4 to -0.22) | <0.001 |
|  | Guam | 8821.58  (4966.12 to 14193.74) | 8720.30  (5034.81 to 13941.09) | -0.05  (-0.07 to -0.02) | <0.001 | 1531.96  (777.42 to 2770.40) | 1439.66  (729.90 to 2595.31) | -0.2  (-0.27 to -0.13) | <0.001 |
|  | Guatemala | 6997.18  (4625.24 to 10243.12) | 6796.01  (3946.37 to 10889.19) | -0.09  (-0.11 to -0.08) | <0.001 | 1569.17  (822.19 to 2748.78) | 1283.22  (643.55 to 2325.14) | -0.67  (-0.74 to -0.61) | <0.001 |
|  | Guinea | 4068.87  (2648.57 to 6091.92) | 3722.76  (2379.02 to 5677.44) | -0.28  (-0.32 to -0.24) | <0.001 | 1731.03  (985.40 to 2923.29) | 1523.55  (833.01 to 2611.79) | -0.41  (-0.46 to -0.35) | <0.001 |
|  | Guinea-Bissau | 3987.24  (2548.68 to 6014.78) | 3709.34  (2328.70 to 5633.35) | -0.23  (-0.25 to -0.21) | <0.001 | 1762.27  (982.89 to 2972.85) | 1535.58  (856.98 to 2635.92) | -0.46  (-0.48 to -0.44) | <0.001 |
|  | Guyana | 8445.00  (4900.94 to 13482.06) | 8392.73  (4891.93 to 13486.75) | -0.01  (-0.06 to 0.04) | 0.669 | 1278.39  (677.99 to 2228.83) | 1176.36  (611.48 to 2082.42) | -0.27  (-0.3 to -0.24) | <0.001 |
|  | Haiti | 8654.52  (5024.29 to 14068.74) | 8527.82  (4993.48 to 13638.64) | -0.04  (-0.05 to -0.03) | <0.001 | 1383.12  (734.87 to 2391.11) | 1256.87  (657.07 to 2235.29) | -0.34  (-0.43 to -0.25) | <0.001 |
|  | Honduras | 7337.45  (4245.47 to 11798.75) | 7203.91  (4121.98 to 11525.72) | -0.05  (-0.07 to -0.02) | <0.001 | 985.61  (516.55 to 1748.41) | 911.99  (487.35 to 1624.09) | -0.26  (-0.31 to -0.2) | <0.001 |
|  | Hungary | 4483.11  (2600.23 to 7200.45) | 4470.13  (2589.53 to 7232.14) | -0.01  (-0.03 to 0.01) | 0.267 | 2863.90  (1489.72 to 5123.40) | 2543.26  (1315.43 to 4565.49) | -0.38  (-0.4 to -0.36) | <0.001 |
|  | Iceland | 503.11  (292.81 to 814.05) | 495.03  (285.44 to 791.71) | -0.05  (-0.07 to -0.03) | <0.001 | 112.04  (61.60 to 191.37) | 97.16  (52.81 to 169.62) | -0.49  (-0.55 to -0.44) | <0.001 |
|  | India | 2210.39  (1385.57 to 3468.19) | 2171.43  (1347.20 to 3444.37) | -0.07  (-0.23 to 0.1) | 0.413 | 797.07  (398.76 to 1449.82) | 701.42  (348.91 to 1277.54) | -0.41  (-0.53 to -0.3) | <0.001 |
|  | Indonesia | 5090.88  (2913.90 to 8300.54) | 4950.67  (2826.33 to 8076.98) | -0.08  (-0.1 to -0.07) | <0.001 | 504.85  (239.84 to 919.85) | 465.73  (221.47 to 856.22) | -0.26  (-0.28 to -0.24) | <0.001 |
|  | Iran (Islamic Republic of) | 7380.08  (4279.22 to 11835.59) | 6821.22  (3918.28 to 10932.76) | -0.28  (-0.53 to -0.04) | 0.025 | 1116.07  (529.02 to 2079.18) | 1103.05  (522.83 to 2054.30) | -0.06  (-0.19 to 0.08) | 0.419 |
|  | Iraq | 7842.84  (5217.98 to 11279.33) | 5868.59  (3368.22 to 9411.40) | -0.92  (-1.1 to -0.75) | <0.001 | 1086.24  (554.21 to 1959.55) | 1006.07  (526.61 to 1803.52) | -0.25  (-0.29 to -0.21) | <0.001 |
|  | Ireland | 714.02  (423.81 to 1136.57) | 707.74  (418.91 to 1137.24) | -0.02  (-0.08 to 0.04) | 0.518 | 113.26  (63.44 to 193.11) | 100.03  (56.40 to 171.89) | -0.41  (-0.47 to -0.36) | <0.001 |
|  | Israel | 1091.45  (646.48 to 1710.33) | 880.36  (509.98 to 1409.81) | -0.72  (-0.79 to -0.64) | <0.001 | 115.95  (64.98 to 198.44) | 102.28  (56.38 to 174.63) | -0.41  (-0.51 to -0.31) | <0.001 |
|  | Italy | 858.47  (526.63 to 1311.65) | 1024.23  (679.99 to 1477.95) | 0.59  (0.46 to 0.73) | <0.001 | 147.50  (83.80 to 238.98) | 150.32  (92.50 to 231.73) | 0.06  (0.01 to 0.11) | 0.028 |
|  | Jamaica | 8356.85  (4806.20 to 13387.37) | 8339.15  (4794.16 to 13378.67) | 0  (-0.01 to 0.01) | 0.671 | 1188.82  (622.28 to 2082.42) | 1126.77  (597.86 to 2015.88) | -0.21  (-0.36 to -0.06) | 0.007 |
|  | Japan | 1572.19  (980.94 to 2380.56) | 1659.71  (1058.24 to 2473.51) | 0.16  (0.11 to 0.21) | <0.001 | 451.62  (249.65 to 737.42) | 385.81  (215.90 to 627.52) | -0.51  (-0.53 to -0.49) | <0.001 |
|  | Jordan | 1758.52  (1019.77 to 2850.69) | 1682.05  (974.03 to 2731.24) | -0.13  (-0.18 to -0.09) | <0.001 | 874.44  (448.75 to 1565.44) | 860.76  (438.47 to 1555.86) | -0.11  (-0.36 to 0.14) | 0.382 |
|  | Kazakhstan | 11058.28  (6415.18 to 17543.82) | 11109.41  (6464.94 to 17672.87) | 0.02  (-0.03 to 0.07) | 0.495 | 3388.05  (1737.69 to 6079.03) | 2936.33  (1488.93 to 5237.01) | -0.47  (-0.51 to -0.43) | <0.001 |
|  | Kenya | 4552.58  (2701.81 to 7290.75) | 4585.29  (2722.85 to 7351.90) | 0  (-0.16 to 0.17) | 0.979 | 1685.80  (835.01 to 3026.78) | 1647.15  (820.15 to 2947.14) | -0.07  (-0.23 to 0.1) | 0.434 |
|  | Kiribati | 9585.71  (5538.83 to 15429.01) | 9474.70  (5502.72 to 14991.35) | -0.04  (-0.09 to 0.01) | 0.079 | 495.05  (250.12 to 882.53) | 490.27  (247.90 to 888.48) | -0.02  (-0.14 to 0.1) | 0.738 |
|  | Kuwait | 3046.84  (1730.60 to 4996.43) | 2974.58  (1710.50 to 4786.52) | -0.1  (-0.33 to 0.13) | 0.407 | 1554.18  (789.68 to 2774.12) | 1401.14  (709.92 to 2507.68) | -0.3  (-0.42 to -0.17) | <0.001 |
|  | Kyrgyzstan | 11056.60  (6535.08 to 17741.43) | 11127.45  (6496.38 to 17772.99) | 0.02  (0 to 0.04) | 0.014 | 3303.44  (1735.15 to 5790.90) | 2997.34  (1550.12 to 5277.35) | -0.32  (-0.36 to -0.28) | <0.001 |
|  | Lao People's Democratic Republic | 5374.29  (3056.85 to 8804.72) | 5194.91  (2980.17 to 8509.41) | -0.1  (-0.12 to -0.08) | <0.001 | 492.37  (246.56 to 888.38) | 433.88  (219.88 to 780.35) | -0.4  (-0.44 to -0.36) | <0.001 |
|  | Latvia | 6758.49  (3910.86 to 10909.03) | 6793.83  (3925.60 to 11027.16) | 0.02  (0 to 0.03) | 0.01 | 3064.94  (1582.17 to 5528.96) | 2530.87  (1293.32 to 4534.59) | -0.68  (-0.76 to -0.59) | <0.001 |
|  | Lebanon | 2613.71  (1501.23 to 4289.38) | 2336.22  (1337.99 to 3753.59) | -0.14  (-0.29 to 0.02) | 0.078 | 1022.16  (524.59 to 1841.02) | 994.43  (514.54 to 1772.76) | -0.07  (-0.15 to 0) | 0.063 |
|  | Lesotho | 6714.71  (3937.65 to 10594.64) | 6635.63  (3862.18 to 10524.33) | -0.02  (-0.03 to -0.01) | <0.001 | 2971.33  (1562.26 to 5213.97) | 2791.64  (1462.48 to 4831.99) | -0.19  (-0.25 to -0.14) | <0.001 |
|  | Liberia | 3960.65  (2566.42 to 6010.11) | 3690.18  (2369.08 to 5602.45) | -0.23  (-0.26 to -0.21) | <0.001 | 1706.21  (980.92 to 2888.87) | 1508.07  (822.64 to 2586.55) | -0.4  (-0.44 to -0.35) | <0.001 |
|  | Libya | 4620.18  (2612.75 to 7451.20) | 4421.92  (2528.24 to 7190.68) | -0.09  (-0.12 to -0.07) | <0.001 | 1044.14  (531.44 to 1905.06) | 1020.19  (523.22 to 1802.44) | -0.07  (-0.12 to -0.02) | 0.009 |
|  | Lithuania | 6724.01  (3890.67 to 10823.28) | 6794.71  (3921.99 to 11048.85) | 0.03  (0.02 to 0.05) | <0.001 | 2988.15  (1535.07 to 5237.40) | 2563.35  (1325.26 to 4584.01) | -0.52  (-0.54 to -0.49) | <0.001 |
|  | Luxembourg | 476.92  (298.46 to 724.18) | 479.98  (305.10 to 736.79) | 0  (-0.07 to 0.08) | 0.907 | 128.36  (75.83 to 211.19) | 115.29  (67.63 to 191.95) | -0.36  (-0.4 to -0.32) | <0.001 |
|  | Madagascar | 4518.95  (2704.51 to 7126.15) | 4429.31  (2593.56 to 7077.42) | -0.06  (-0.08 to -0.05) | <0.001 | 1784.17  (974.72 to 3091.44) | 1725.89  (926.24 to 3044.69) | -0.14  (-0.18 to -0.09) | <0.001 |
|  | Malawi | 5347.39  (3307.31 to 8174.75) | 4943.72  (2850.54 to 7928.55) | -0.26  (-0.33 to -0.18) | <0.001 | 3133.90  (1644.53 to 5503.20) | 3067.67  (1595.38 to 5310.26) | -0.12  (-0.2 to -0.04) | 0.004 |
|  | Malaysia | 5175.70  (2927.03 to 8375.98) | 5089.91  (2828.70 to 8255.45) | -0.04  (-0.09 to 0) | 0.044 | 448.05  (223.27 to 799.76) | 411.41  (207.36 to 749.71) | -0.27  (-0.32 to -0.23) | <0.001 |
|  | Maldives | 5205.64  (2965.22 to 8547.06) | 5075.22  (2901.52 to 8296.67) | -0.08  (-0.1 to -0.06) | <0.001 | 478.28  (241.81 to 860.41) | 418.12  (210.04 to 743.71) | -0.43  (-0.47 to -0.38) | <0.001 |
|  | Mali | 4860.70  (3189.33 to 7182.31) | 4087.06  (2552.67 to 6262.80) | -0.56  (-0.62 to -0.49) | <0.001 | 1722.24  (966.94 to 2784.82) | 1400.37  (788.23 to 2402.57) | -0.68  (-0.74 to -0.62) | <0.001 |
|  | Malta | 512.59  (302.84 to 820.69) | 503.67  (293.72 to 806.37) | -0.04  (-0.13 to 0.04) | 0.3 | 112.88  (62.29 to 195.93) | 100.46  (54.99 to 176.49) | -0.41  (-0.51 to -0.3) | <0.001 |
|  | Marshall Islands | 2069.08  (1213.82 to 3311.83) | 2044.86  (1190.13 to 3283.76) | -0.08  (-0.34 to 0.18) | 0.563 | 1644.84  (827.63 to 2969.42) | 1576.54  (804.34 to 2825.10) | -0.14  (-0.23 to -0.04) | 0.004 |
|  | Mauritania | 3984.21  (2542.25 to 6055.27) | 3705.97  (2365.71 to 5681.38) | -0.23  (-0.25 to -0.21) | <0.001 | 1735.34  (972.91 to 2907.01) | 1498.17  (836.75 to 2545.50) | -0.47  (-0.51 to -0.44) | <0.001 |
|  | Mauritius | 5148.91  (2931.23 to 8432.43) | 5082.04  (2902.25 to 8306.07) | -0.04  (-0.05 to -0.03) | <0.001 | 444.79  (223.52 to 796.31) | 386.90  (195.72 to 700.56) | -0.45  (-0.48 to -0.42) | <0.001 |
|  | Mexico | 9524.69  (5594.38 to 15157.68) | 9404.54  (5543.99 to 14984.83) | -0.04  (-0.07 to -0.01) | 0.003 | 985.42  (489.41 to 1783.53) | 848.48  (420.81 to 1537.49) | -0.48  (-0.5 to -0.46) | <0.001 |
|  | Micronesia (Federated States of) | 9011.81  (5189.91 to 14262.99) | 8906.35  (5174.35 to 14242.87) | -0.04  (-0.08 to -0.01) | 0.02 | 1698.02  (858.04 to 3013.50) | 1596.66  (816.68 to 2927.36) | -0.21  (-0.25 to -0.16) | <0.001 |
|  | Monaco | 526.99  (318.35 to 825.99) | 520.69  (309.81 to 813.85) | -0.03  (-0.06 to -0.01) | 0.002 | 110.11  (62.04 to 188.19) | 102.80  (57.81 to 177.98) | -0.24  (-0.29 to -0.19) | <0.001 |
|  | Mongolia | 11205.08  (6567.79 to 17639.11) | 11136.49  (6540.62 to 17456.70) | -0.01  (-0.09 to 0.08) | 0.875 | 3328.63  (1708.14 to 5909.32) | 3055.29  (1571.12 to 5398.35) | -0.27  (-0.33 to -0.22) | <0.001 |
|  | Montenegro | 4509.41  (2613.39 to 7269.01) | 4476.52  (2595.02 to 7298.04) | -0.03  (-0.08 to 0.02) | 0.226 | 2831.59  (1457.32 to 5097.21) | 2675.80  (1373.32 to 4759.93) | -0.19  (-0.21 to -0.17) | <0.001 |
|  | Morocco | 6499.30  (3822.61 to 10289.60) | 4634.18  (2644.96 to 7533.46) | -1.02  (-1.34 to -0.71) | <0.001 | 1255.18  (644.62 to 2235.65) | 1175.32  (593.63 to 2101.95) | -0.23  (-0.32 to -0.13) | <0.001 |
|  | Mozambique | 5109.01  (3045.92 to 8158.58) | 4999.44  (2959.91 to 7867.91) | -0.08  (-0.16 to -0.01) | 0.035 | 2506.23  (1334.31 to 4293.02) | 2349.37  (1233.83 to 4136.22) | -0.22  (-0.27 to -0.18) | <0.001 |
|  | Myanmar | 5300.05  (3007.97 to 8507.56) | 5161.78  (2942.71 to 8474.25) | -0.06  (-0.08 to -0.04) | <0.001 | 491.92  (252.55 to 877.70) | 436.96  (220.60 to 782.06) | -0.37  (-0.44 to -0.31) | <0.001 |
|  | Namibia | 6709.98  (3914.55 to 10773.57) | 6581.64  (3803.40 to 10468.24) | -0.06  (-0.06 to -0.05) | <0.001 | 2963.85  (1599.34 to 5153.99) | 2650.48  (1407.91 to 4599.20) | -0.36  (-0.38 to -0.33) | <0.001 |
|  | Nauru | 8998.91  (5214.19 to 14345.20) | 8893.79  (5087.13 to 14262.86) | -0.05  (-0.07 to -0.02) | <0.001 | 1533.13  (787.87 to 2768.23) | 1484.21  (757.11 to 2658.74) | -0.12  (-0.19 to -0.04) | 0.002 |
|  | Nepal | 3810.80  (2271.13 to 6070.66) | 3658.71  (2138.99 to 5855.11) | -0.13  (-0.18 to -0.07) | <0.001 | 645.36  (363.60 to 1095.08) | 528.43  (291.89 to 924.12) | -0.64  (-0.7 to -0.59) | <0.001 |
|  | Netherlands | 989.60  (598.29 to 1558.07) | 735.71  (429.22 to 1178.88) | -0.95  (-1.04 to -0.87) | <0.001 | 102.77  (59.10 to 170.78) | 94.77  (52.66 to 162.82) | -0.29  (-0.38 to -0.21) | <0.001 |
|  | New Zealand | 3886.97  (2298.05 to 6110.39) | 4152.44  (2509.05 to 6365.21) | 0.18  (0.09 to 0.27) | <0.001 | 289.43  (149.15 to 492.98) | 289.93  (152.78 to 447.58) | 0.01  (-0.03 to 0.04) | 0.722 |
|  | Nicaragua | 7882.20  (4829.68 to 12240.35) | 7434.02  (4259.76 to 11933.90) | -0.19  (-0.21 to -0.18) | <0.001 | 1611.12  (803.72 to 2919.75) | 1356.39  (663.32 to 2502.82) | -0.57  (-0.62 to -0.51) | <0.001 |
|  | Niger | 4063.47  (2599.84 to 6197.22) | 3664.94  (2288.92 to 5663.55) | -0.3  (-0.4 to -0.21) | <0.001 | 2310.56  (1268.33 to 3866.32) | 1900.56  (1004.60 to 3306.83) | -0.62  (-0.69 to -0.56) | <0.001 |
|  | Nigeria | 5717.50  (3576.45 to 8849.83) | 5675.50  (3461.11 to 8928.91) | -0.03  (-0.2 to 0.15) | 0.76 | 1389.31  (735.78 to 2414.41) | 1246.46  (652.01 to 2192.06) | -0.36  (-0.39 to -0.33) | <0.001 |
|  | Niue | 8876.09  (5083.46 to 14307.37) | 8818.54  (5109.89 to 14214.49) | -0.02  (-0.04 to -0.01) | 0.013 | 1514.18  (770.52 to 2752.42) | 1467.60  (745.52 to 2668.44) | -0.09  (-0.15 to -0.04) | 0.001 |
|  | North Macedonia | 4479.90  (2589.94 to 7289.97) | 4478.79  (2604.26 to 7211.04) | 0  (-0.01 to 0) | 0.259 | 2703.65  (1390.07 to 4838.68) | 2590.12  (1334.22 to 4579.57) | -0.14  (-0.16 to -0.12) | <0.001 |
|  | Northern Mariana Islands | 8830.75  (5116.44 to 14198.39) | 8696.27  (4992.29 to 13874.23) | -0.05  (-0.06 to -0.04) | <0.001 | 1575.84  (804.19 to 2782.10) | 1482.11  (751.10 to 2667.72) | -0.2  (-0.28 to -0.12) | <0.001 |
|  | Norway | 1050.63  (690.31 to 1562.44) | 1031.89  (687.30 to 1522.66) | -0.13  (-0.44 to 0.18) | 0.414 | 145.37  (89.70 to 229.76) | 160.76  (104.04 to 248.25) | 0.34  (0.2 to 0.48) | <0.001 |
|  | Oman | 4571.04  (2590.15 to 7382.96) | 4398.71  (2510.73 to 7216.38) | -0.09  (-0.12 to -0.07) | <0.001 | 1048.46  (531.97 to 1895.70) | 1000.77  (514.58 to 1764.26) | -0.14  (-0.2 to -0.08) | <0.001 |
|  | Pakistan | 6565.77  (3857.21 to 10538.25) | 6105.16  (3570.16 to 9809.84) | -0.22  (-0.29 to -0.16) | <0.001 | 413.13  (226.52 to 710.50) | 360.34  (197.37 to 613.85) | -0.43  (-0.47 to -0.39) | <0.001 |
|  | Palau | 8878.01  (5104.75 to 14334.85) | 8803.17  (5092.04 to 14052.03) | -0.03  (-0.04 to -0.02) | <0.001 | 1520.35  (778.79 to 2698.67) | 1473.24  (745.57 to 2663.31) | -0.09  (-0.16 to -0.01) | 0.018 |
|  | Palestine | 4582.20  (2622.89 to 7440.75) | 4432.81  (2538.23 to 7214.04) | -0.08  (-0.1 to -0.06) | <0.001 | 1065.25  (544.45 to 1912.30) | 1035.00  (541.99 to 1845.06) | -0.09  (-0.13 to -0.05) | <0.001 |
|  | Panama | 7209.54  (4106.65 to 11357.94) | 7091.13  (4081.44 to 11242.36) | -0.05  (-0.06 to -0.04) | <0.001 | 861.06  (446.00 to 1547.41) | 828.38  (432.79 to 1459.01) | -0.13  (-0.14 to -0.11) | <0.001 |
|  | Papua New Guinea | 13731.25  (8361.79 to 20928.64) | 11560.99  (6755.23 to 18314.81) | -0.56  (-0.67 to -0.45) | <0.001 | 7127.82  (3673.39 to 12238.20) | 7041.67  (3647.84 to 12214.27) | -0.03  (-0.1 to 0.05) | 0.454 |
|  | Paraguay | 9638.74  (5583.65 to 15396.60) | 9599.96  (5494.60 to 15327.41) | -0.02  (-0.04 to 0) | 0.111 | 1195.25  (611.77 to 2132.22) | 1141.45  (589.88 to 2019.80) | -0.15  (-0.19 to -0.11) | <0.001 |
|  | Peru | 6203.82  (3629.45 to 9898.16) | 6141.60  (3558.95 to 9894.35) | 0  (-0.16 to 0.16) | 0.982 | 249.88  (152.02 to 399.73) | 251.58  (147.62 to 383.92) | 0.05  (-0.16 to 0.27) | 0.646 |
|  | Philippines | 5937.04  (3444.87 to 9648.25) | 5854.11  (3400.40 to 9476.60) | -0.04  (-0.06 to -0.01) | 0.007 | 430.54  (213.48 to 771.29) | 424.31  (213.02 to 750.48) | -0.05  (-0.06 to -0.03) | <0.001 |
|  | Poland | 4554.69  (2691.33 to 7369.98) | 4555.24  (2701.36 to 7384.06) | 0  (-0.04 to 0.04) | 0.95 | 2579.72  (1269.84 to 4640.99) | 2192.27  (1088.87 to 3912.71) | -0.52  (-0.6 to -0.43) | <0.001 |
|  | Portugal | 815.46  (457.01 to 1325.13) | 830.40  (473.60 to 1346.55) | 0.04  (-0.05 to 0.14) | 0.35 | 112.82  (60.85 to 196.62) | 104.41  (58.17 to 180.14) | -0.27  (-0.38 to -0.16) | <0.001 |
|  | Puerto Rico | 8231.04  (4785.50 to 13242.55) | 8257.80  (4824.08 to 13303.52) | 0.01  (-0.01 to 0.03) | 0.202 | 1161.50  (602.32 to 2064.25) | 1090.14  (572.00 to 1951.41) | -0.2  (-0.26 to -0.14) | <0.001 |
|  | Qatar | 5312.58  (3030.08 to 8586.23) | 5162.32  (2956.94 to 8296.48) | -0.08  (-0.12 to -0.05) | <0.001 | 1030.32  (532.33 to 1833.21) | 1004.42  (515.04 to 1809.90) | -0.08  (-0.13 to -0.03) | 0.002 |
|  | Republic of Korea | 1423.88  (904.74 to 2074.94) | 1610.66  (996.01 to 2431.24) | 0.41  (0.36 to 0.46) | <0.001 | 453.28  (255.26 to 717.69) | 384.05  (213.63 to 622.45) | -0.55  (-0.62 to -0.47) | <0.001 |
|  | Republic of Moldova | 6816.29  (3923.91 to 11042.01) | 6837.41  (3937.70 to 11057.22) | 0.01  (-0.01 to 0.03) | 0.342 | 3044.15  (1581.49 to 5408.46) | 2621.91  (1345.68 to 4661.45) | -0.5  (-0.54 to -0.46) | <0.001 |
|  | Romania | 4547.91  (2658.05 to 7339.18) | 4510.51  (2630.98 to 7273.71) | -0.03  (-0.03 to -0.02) | <0.001 | 2795.99  (1425.04 to 4998.22) | 2556.76  (1287.80 to 4557.72) | -0.29  (-0.35 to -0.23) | <0.001 |
|  | Russian Federation | 6529.75  (3855.22 to 10539.26) | 6542.02  (3841.52 to 10561.08) | 0  (-0.01 to 0.01) | 0.713 | 2933.20  (1411.22 to 5299.50) | 2662.91  (1271.04 to 4872.96) | -0.3  (-0.35 to -0.25) | <0.001 |
|  | Rwanda | 4092.23  (2431.67 to 6475.37) | 3909.03  (2303.27 to 6294.08) | -0.14  (-0.16 to -0.11) | <0.001 | 1819.46  (976.26 to 3146.78) | 1662.64  (872.79 to 2909.69) | -0.35  (-0.41 to -0.3) | <0.001 |
|  | Saint Kitts and Nevis | 8318.20  (4807.24 to 13378.00) | 8288.26  (4801.75 to 13203.16) | 0  (-0.02 to 0.03) | 0.858 | 1155.88  (608.26 to 2047.75) | 1088.22  (566.42 to 1947.11) | -0.19  (-0.2 to -0.18) | <0.001 |
|  | Saint Lucia | 8379.07  (4830.82 to 13508.16) | 8332.92  (4856.54 to 13393.14) | -0.01  (-0.06 to 0.04) | 0.637 | 1276.45  (680.39 to 2229.76) | 1154.24  (596.51 to 2049.56) | -0.32  (-0.4 to -0.25) | <0.001 |
|  | Saint Vincent and the Grenadines | 8364.71  (4847.76 to 13491.03) | 8326.63  (4800.23 to 13331.02) | -0.01  (-0.03 to 0) | 0.112 | 1282.85  (670.03 to 2225.05) | 1174.97  (614.19 to 2116.99) | -0.29  (-0.36 to -0.22) | <0.001 |
|  | Samoa | 13705.18  (8126.82 to 21413.08) | 13545.49  (7986.43 to 21216.07) | -0.03  (-0.41 to 0.35) | 0.86 | 2179.14  (1113.66 to 3858.72) | 2128.57  (1087.40 to 3805.83) | -0.07  (-0.1 to -0.03) | <0.001 |
|  | San Marino | 526.97  (318.08 to 825.79) | 519.75  (310.79 to 807.48) | -0.04  (-0.06 to -0.02) | 0.001 | 110.55  (61.89 to 187.74) | 102.85  (57.91 to 176.40) | -0.25  (-0.35 to -0.15) | <0.001 |
|  | Sao Tome and Principe | 3703.89  (2292.83 to 5755.45) | 3662.39  (2294.16 to 5609.77) | -0.03  (-0.05 to -0.01) | 0.002 | 1558.12  (831.84 to 2661.17) | 1471.64  (816.78 to 2507.77) | -0.18  (-0.2 to -0.17) | <0.001 |
|  | Saudi Arabia | 12220.83  (7408.57 to 18795.71) | 9889.83  (5748.78 to 15572.94) | -0.62  (-0.66 to -0.58) | <0.001 | 513.87  (242.07 to 973.89) | 517.33  (244.01 to 971.79) | 0  (-0.2 to 0.2) | 0.973 |
|  | Senegal | 3813.08  (2586.11 to 5496.51) | 2569.60  (1717.30 to 3789.34) | -1.31  (-1.4 to -1.23) | <0.001 | 924.07  (608.03 to 1353.72) | 637.53  (403.67 to 1006.71) | -1.29  (-1.5 to -1.08) | <0.001 |
|  | Serbia | 4517.82  (2617.48 to 7326.90) | 4483.87  (2590.82 to 7266.80) | -0.02  (-0.04 to 0.01) | 0.129 | 2804.00  (1450.38 to 4919.99) | 2647.74  (1364.06 to 4716.80) | -0.19  (-0.21 to -0.17) | <0.001 |
|  | Seychelles | 4597.55  (2618.15 to 7525.34) | 4427.54  (2512.48 to 7223.92) | -0.09  (-0.18 to 0) | 0.045 | 464.84  (231.63 to 841.88) | 440.98  (222.18 to 794.45) | -0.17  (-0.21 to -0.14) | <0.001 |
|  | Sierra Leone | 3862.28  (2446.19 to 5934.06) | 3681.52  (2361.02 to 5547.87) | -0.15  (-0.17 to -0.14) | <0.001 | 1625.51  (922.99 to 2737.15) | 1490.88  (820.44 to 2576.84) | -0.28  (-0.31 to -0.24) | <0.001 |
|  | Singapore | 1615.71  (985.51 to 2454.18) | 1612.60  (991.89 to 2433.57) | 0  (-0.02 to 0.01) | 0.589 | 444.15  (249.75 to 718.36) | 355.93  (199.00 to 579.57) | -0.72  (-0.82 to -0.63) | <0.001 |
|  | Slovakia | 4462.20  (2593.77 to 7190.88) | 4442.78  (2567.63 to 7151.83) | -0.02  (-0.05 to 0.01) | 0.121 | 2752.41  (1390.07 to 4900.03) | 2657.71  (1361.43 to 4756.81) | -0.15  (-0.21 to -0.08) | <0.001 |
|  | Slovenia | 3032.99  (1790.26 to 4902.56) | 3035.11  (1780.20 to 4871.65) | 0.02  (-0.06 to 0.09) | 0.683 | 2667.98  (1371.34 to 4731.23) | 2365.27  (1200.24 to 4284.23) | -0.41  (-0.46 to -0.37) | <0.001 |
|  | Solomon Islands | 7401.28  (4220.68 to 11793.61) | 7196.61  (4027.12 to 11643.69) | -0.11  (-0.16 to -0.06) | <0.001 | 752.90  (394.14 to 1329.63) | 687.99  (361.17 to 1221.95) | -0.27  (-0.42 to -0.12) | 0.001 |
|  | Somalia | 5057.76  (3068.91 to 7847.28) | 3613.41  (2147.84 to 5810.36) | -1.13  (-1.2 to -1.06) | <0.001 | 1145.71  (642.04 to 1914.85) | 1132.39  (628.17 to 1947.84) | -0.09  (-0.26 to 0.07) | 0.278 |
|  | South Africa | 11170.80  (6694.65 to 17337.78) | 10195.08  (6023.68 to 16022.54) | -0.29  (-0.38 to -0.2) | <0.001 | 6448.74  (3466.77 to 10654.51) | 6010.27  (3074.01 to 10299.08) | -0.3  (-0.53 to -0.08) | 0.009 |
|  | South Sudan | 4579.86  (2730.29 to 7279.57) | 4472.90  (2633.51 to 7113.96) | -0.07  (-0.09 to -0.06) | <0.001 | 1904.95  (1035.01 to 3283.02) | 1806.93  (972.89 to 3163.76) | -0.23  (-0.27 to -0.19) | <0.001 |
|  | Spain | 409.76  (244.07 to 648.71) | 484.92  (304.36 to 735.12) | 0.52  (0.41 to 0.62) | <0.001 | 207.57  (110.12 to 368.19) | 206.21  (110.56 to 364.78) | -0.01  (-0.18 to 0.17) | 0.941 |
|  | Sri Lanka | 5181.46  (2917.81 to 8538.49) | 5080.48  (2869.30 to 8324.04) | -0.06  (-0.07 to -0.05) | <0.001 | 759.01  (389.29 to 1331.85) | 849.99  (423.06 to 1530.51) | 0.37  (0.27 to 0.47) | <0.001 |
|  | Sudan | 7169.26  (4093.92 to 11495.69) | 7493.54  (4513.53 to 11623.31) | 0.08  (-0.27 to 0.43) | 0.658 | 1812.24  (929.61 to 3235.98) | 1720.74  (882.52 to 3079.15) | -0.15  (-0.22 to -0.08) | <0.001 |
|  | Suriname | 8494.26  (4949.54 to 13485.38) | 8424.27  (4873.00 to 13584.68) | -0.02  (-0.05 to 0.01) | 0.256 | 1251.43  (654.05 to 2161.69) | 1133.77  (592.31 to 2011.27) | -0.32  (-0.4 to -0.25) | <0.001 |
|  | Sweden | 1474.38  (831.92 to 2443.96) | 1435.42  (820.97 to 2321.64) | -0.09  (-0.19 to 0) | 0.061 | 125.85  (69.32 to 216.05) | 119.52  (72.90 to 193.71) | -0.18  (-0.22 to -0.13) | <0.001 |
|  | Switzerland | 521.64  (324.32 to 802.62) | 522.70  (330.09 to 793.51) | 0  (-0.06 to 0.06) | 0.897 | 120.21  (70.00 to 201.84) | 110.63  (64.70 to 184.13) | -0.29  (-0.36 to -0.21) | <0.001 |
|  | Syrian Arab Republic | 4644.11  (2660.84 to 7536.81) | 4488.96  (2587.26 to 7238.61) | -0.07  (-0.1 to -0.04) | <0.001 | 1069.31  (551.88 to 1907.75) | 1007.75  (516.94 to 1789.44) | -0.21  (-0.26 to -0.15) | <0.001 |
|  | Taiwan (Province of China) | 10601.52  (6375.68 to 16428.07) | 9372.34  (5348.37 to 15124.00) | -0.4  (-0.41 to -0.38) | <0.001 | 901.86  (454.32 to 1632.91) | 866.68  (444.47 to 1555.25) | -0.14  (-0.17 to -0.11) | <0.001 |
|  | Tajikistan | 11268.38  (6528.87 to 17933.17) | 11198.90  (6539.32 to 17627.95) | -0.02  (-0.07 to 0.03) | 0.377 | 3671.30  (1894.28 to 6460.45) | 3383.89  (1732.12 to 5955.37) | -0.27  (-0.3 to -0.24) | <0.001 |
|  | Thailand | 7383.50  (4464.23 to 11416.86) | 6661.77  (3772.46 to 10840.02) | -0.31  (-0.36 to -0.26) | <0.001 | 454.90  (234.32 to 799.91) | 487.52  (242.06 to 874.34) | 0.22  (0.17 to 0.27) | <0.001 |
|  | Timor-Leste | 5352.82  (3032.42 to 8761.34) | 5188.13  (2958.24 to 8421.12) | -0.1  (-0.11 to -0.08) | <0.001 | 479.67  (242.08 to 860.06) | 434.36  (221.20 to 777.94) | -0.33  (-0.36 to -0.3) | <0.001 |
|  | Togo | 3931.90  (2542.10 to 5981.39) | 3704.45  (2369.67 to 5670.47) | -0.19  (-0.23 to -0.15) | <0.001 | 1677.46  (953.15 to 2831.93) | 1483.92  (810.91 to 2549.52) | -0.39  (-0.43 to -0.36) | <0.001 |
|  | Tokelau | 8916.82  (5099.61 to 14399.67) | 8862.36  (5071.50 to 14219.25) | -0.04  (-0.05 to -0.03) | <0.001 | 1560.91  (782.59 to 2814.67) | 1492.58  (760.75 to 2673.77) | -0.13  (-0.19 to -0.08) | <0.001 |
|  | Tonga | 10450.60  (6070.32 to 16670.08) | 10404.11  (5997.77 to 16240.47) | -0.01  (-0.05 to 0.03) | 0.534 | 2049.16  (1040.38 to 3637.73) | 1942.80  (991.64 to 3505.07) | -0.17  (-0.24 to -0.1) | <0.001 |
|  | Trinidad and Tobago | 8385.62  (4826.72 to 13460.00) | 8298.88  (4818.70 to 13370.42) | -0.02  (-0.04 to 0) | 0.101 | 1130.95  (586.40 to 1995.36) | 1064.38  (564.23 to 1908.71) | -0.2  (-0.27 to -0.12) | <0.001 |
|  | Tunisia | 4622.66  (2612.63 to 7534.85) | 4430.47  (2526.15 to 7221.29) | -0.12  (-0.25 to 0.01) | 0.069 | 2205.60  (1238.62 to 3529.90) | 1631.26  (829.49 to 2918.00) | -0.96  (-1.11 to -0.81) | <0.001 |
|  | Turkmenistan | 11124.70  (6478.57 to 17784.64) | 11138.55  (6531.91 to 17650.20) | 0  (-0.07 to 0.07) | 0.97 | 3492.82  (1823.23 to 6159.82) | 2764.97  (1438.98 to 4916.32) | -0.76  (-0.85 to -0.67) | <0.001 |
|  | Tuvalu | 8908.88  (5158.47 to 14222.11) | 8848.14  (5093.16 to 14170.65) | -0.02  (-0.05 to 0) | 0.056 | 1570.16  (791.60 to 2805.03) | 1504.99  (763.01 to 2686.26) | -0.13  (-0.19 to -0.06) | <0.001 |
|  | Türkiye | 6167.00  (3866.59 to 9266.55) | 4731.00  (2737.48 to 7610.60) | -0.86 (-0.95 to -0.77) | <0.001 | 3379.22  (1885.05 to 5567.26) | 2411.81  (1224.74 to 4337.20) | -1.04 (-1.1 to -0.98) | <0.001 |
|  | Uganda | 6261.80  (3742.49 to 9869.10) | 5330.86  (3141.27 to 8470.05) | -0.52  (-0.58 to -0.47) | <0.001 | 2521.69  (1352.11 to 4279.65) | 2638.88  (1395.48 to 4622.61) | 0.14  (-0.03 to 0.32) | 0.114 |
|  | Ukraine | 6496.64  (3771.68 to 10458.30) | 6577.20  (3845.45 to 10569.07) | 0.03  (-0.01 to 0.07) | 0.123 | 3116.48  (1520.20 to 5667.42) | 2617.23  (1256.74 to 4774.23) | -0.56  (-0.64 to -0.49) | <0.001 |
|  | United Arab Emirates | 3619.88  (2060.20 to 5837.00) | 3553.78  (2019.21 to 5713.47) | -0.06  (-0.11 to -0.01) | 0.024 | 1033.33  (526.71 to 1842.01) | 1002.75  (516.53 to 1802.37) | -0.11  (-0.21 to 0) | 0.041 |
|  | United Kingdom | 647.31  (438.94 to 921.16) | 845.97  (616.71 to 1140.89) | 0.85  (0.74 to 0.96) | <0.001 | 132.93  (88.82 to 193.89) | 150.00  (104.83 to 207.67) | 0.33  (0.16 to 0.5) | <0.001 |
|  | United Republic of Tanzania | 5398.85  (3404.49 to 8071.60) | 4635.80  (2716.04 to 7460.54) | -0.58  (-0.91 to -0.24) | 0.001 | 1682.62  (903.83 to 2968.47) | 1371.97  (733.60 to 2419.52) | -0.76  (-1.22 to -0.29) | 0.001 |
|  | United States of America | 897.93  (548.14 to 1378.38) | 1012.25  (613.13 to 1578.64) | 0.39  (0.2 to 0.58) | <0.001 | 305.14  (173.37 to 490.87) | 259.43  (150.49 to 425.71) | -0.6  (-0.89 to -0.3) | <0.001 |
|  | United States Virgin Islands | 8313.23  (4858.05 to 13291.65) | 8268.55  (4808.28 to 13270.87) | 0  (-0.01 to 0) | 0.206 | 1258.88  (659.50 to 2245.77) | 1166.95  (608.67 to 2045.25) | -0.24  (-0.32 to -0.16) | <0.001 |
|  | Uruguay | 1454.54  (940.00 to 2216.36) | 1437.95  (929.98 to 2169.85) | -0.04  (-0.06 to -0.01) | 0.001 | 485.69  (310.02 to 759.91) | 423.71  (268.05 to 661.76) | -0.45  (-0.51 to -0.39) | <0.001 |
|  | Uzbekistan | 11070.73  (6471.42 to 17483.79) | 11062.27  (6464.42 to 17424.13) | 0  (-0.04 to 0.04) | 0.907 | 3519.67  (1823.97 to 6272.51) | 2915.26  (1509.49 to 5182.03) | -0.61  (-0.66 to -0.56) | <0.001 |
|  | Vanuatu | 13001.52  (8044.21 to 19652.55) | 11084.86  (6458.62 to 17553.16) | -0.53  (-0.57 to -0.49) | <0.001 | 2170.65  (1096.81 to 3864.77) | 2080.33  (1060.76 to 3763.98) | -0.13  (-0.19 to -0.06) | <0.001 |
|  | Venezuela (Bolivarian Republic of) | 7249.71  (4191.00 to 11610.61) | 7154.86  (4139.72 to 11430.49) | -0.04  (-0.06 to -0.01) | 0.005 | 920.61  (479.16 to 1646.49) | 853.27  (445.08 to 1508.88) | -0.25  (-0.28 to -0.22) | <0.001 |
|  | Viet Nam | 3513.34  (2135.44 to 5470.72) | 3996.13  (2274.60 to 6619.05) | 0.42  (0.38 to 0.46) | <0.001 | 171.87  (93.21 to 288.92) | 182.70  (97.90 to 316.30) | 0.19  (0.04 to 0.34) | 0.013 |
|  | Yemen | 4717.92  (2681.16 to 7662.23) | 4507.78  (2577.33 to 7291.76) | -0.13  (-0.15 to -0.11) | <0.001 | 1122.94  (570.78 to 2017.40) | 1089.53  (561.77 to 1952.95) | -0.07  (-0.17 to 0.02) | 0.113 |
|  | Zambia | 3521.39  (2144.01 to 5505.17) | 3407.06  (2029.56 to 5386.61) | -0.1  (-0.13 to -0.07) | <0.001 | 1959.37  (1073.94 to 3407.17) | 1856.14  (986.63 to 3249.50) | -0.23  (-0.39 to -0.07) | 0.004 |
|  | Zimbabwe | 4427.47  (2610.68 to 6974.25) | 4393.80  (2586.18 to 6995.06) | -0.02  (-0.03 to -0.01) | 0.005 | 1659.99  (906.54 to 2870.99) | 1594.34  (871.09 to 2762.08) | -0.13  (-0.2 to -0.06) | <0.001 |
| Age-standardized Mortality | Afghanistan | 0.000  (0.000 to 0.020) | 0.000  (0.000 to 0.020) | -0.07  (-0.55 to 0.42) | 0.787 | 0.001  (0.000 to 0.008) | 0.001  (0.000 to 0.006) | -0.11  (-0.58 to 0.36) | 0.657 |
|  | Albania | 0.010  (0.000 to 0.020) | 0.000  (0.000 to 0.010) | -3.08  (-3.6 to -2.55) | <0.001 | 0.004  (0.002 to 0.007) | 0.001  (0.001 to 0.004) | -3.14  (-3.68 to -2.6) | <0.001 |
|  | Algeria | 0.000  (0.000 to 0.010) | 0.000  (0.000 to 0.000) | -1.25  (-1.45 to -1.05) | <0.001 | 0.000  (0.000 to 0.002) | 0.000  (0.000 to 0.001) | -1.28  (-1.48 to -1.08) | <0.001 |
|  | American Samoa | 0.000  (0.000 to 0.000) | 0.000  (0.000 to 0.000) | -1.09  (-3.35 to 1.22) | 0.353 | 0.000  (0.000 to 0.000) | 0.000  (0.000 to 0.000) | -1.08  (-3.34 to 1.23) | 0.357 |
|  | Andorra | 0.010  (0.000 to 0.010) | 0.000  (0.000 to 0.010) | -2.36  (-2.68 to -2.03) | <0.001 | 0.002  (0.001 to 0.005) | 0.001  (0.000 to 0.003) | -2.35  (-2.67 to -2.03) | <0.001 |
|  | Angola | 0.090  (0.020 to 0.220) | 0.050  (0.020 to 0.120) | -2.09  (-2.85 to -1.31) | <0.001 | 0.032  (0.008 to 0.073) | 0.016  (0.005 to 0.039) | -2.08  (-2.86 to -1.3) | <0.001 |
|  | Antigua and Barbuda | 0.010  (0.010 to 0.010) | 0.020  (0.010 to 0.020) | 1.8  (0.06 to 3.57) | 0.042 | 0.003  (0.002 to 0.004) | 0.006  (0.004 to 0.008) | 1.7  (-0.17 to 3.6) | 0.076 |
|  | Argentina | 0.010  (0.010 to 0.020) | 0.010  (0.010 to 0.020) | -0.33  (-1.03 to 0.38) | 0.357 | 0.006  (0.005 to 0.008) | 0.006  (0.004 to 0.008) | -0.37  (-1.03 to 0.3) | 0.283 |
|  | Armenia | 0.040  (0.030 to 0.050) | 0.010  (0.000 to 0.010) | -6.23  (-8.12 to -4.3) | <0.001 | 0.016  (0.013 to 0.020) | 0.002  (0.001 to 0.004) | -6.42  (-8.26 to -4.54) | <0.001 |
|  | Australia | 0.010  (0.000 to 0.010) | 0.000  (0.000 to 0.000) | -3.91  (-5.44 to -2.36) | <0.001 | 0.003  (0.002 to 0.004) | 0.001  (0.001 to 0.001) | -3.98  (-5.51 to -2.43) | <0.001 |
|  | Austria | 0.000  (0.000 to 0.000) | 0.000  (0.000 to 0.000) | -3.27  (-4.14 to -2.38) | <0.001 | 0.001  (0.001 to 0.001) | 0.000  (0.000 to 0.001) | -3.12  (-3.91 to -2.33) | <0.001 |
|  | Azerbaijan | 0.070  (0.040 to 0.100) | 0.030  (0.020 to 0.060) | -2.51  (-3.33 to -1.68) | <0.001 | 0.028  (0.019 to 0.041) | 0.013  (0.006 to 0.026) | -2.51  (-3.35 to -1.67) | <0.001 |
|  | Bahamas | 0.020  (0.010 to 0.030) | 0.040  (0.020 to 0.050) | 2.34  (0.09 to 4.64) | 0.041 | 0.006  (0.004 to 0.008) | 0.012  (0.008 to 0.018) | 2.32  (0.04 to 4.66) | 0.046 |
|  | Bahrain | 0.000  (0.000 to 0.000) | 0.000  (0.000 to 0.000) | 0.64  (-0.96 to 2.25) | 0.436 | 0.000  (0.000 to 0.001) | 0.000  (0.000 to 0.001) | 0.63  (-1 to 2.29) | 0.45 |
|  | Bangladesh | 0.040  (0.010 to 0.080) | 0.010  (0.010 to 0.040) | -2.75  (-3.49 to -2.02) | <0.001 | 0.012  (0.005 to 0.026) | 0.005  (0.002 to 0.012) | -2.73  (-3.47 to -1.99) | <0.001 |
|  | Barbados | 0.030  (0.020 to 0.040) | 0.030  (0.020 to 0.040) | 0.14  (-0.66 to 0.95) | 0.73 | 0.009  (0.006 to 0.012) | 0.009  (0.006 to 0.013) | 0.14  (-0.66 to 0.94) | 0.738 |
|  | Belarus | 0.020  (0.020 to 0.030) | 0.020  (0.010 to 0.030) | -0.87  (-2.05 to 0.33) | 0.155 | 0.009  (0.007 to 0.012) | 0.007  (0.005 to 0.010) | -0.95  (-2.02 to 0.13) | 0.085 |
|  | Belgium | 0.000  (0.000 to 0.010) | 0.000  (0.000 to 0.000) | -3.17  (-3.63 to -2.71) | <0.001 | 0.002  (0.001 to 0.002) | 0.001  (0.000 to 0.001) | -3.21  (-3.68 to -2.74) | <0.001 |
|  | Belize | 0.010  (0.000 to 0.010) | 0.020  (0.010 to 0.020) | 3.19  (1.78 to 4.62) | <0.001 | 0.002  (0.002 to 0.003) | 0.005  (0.004 to 0.008) | 3.21  (1.82 to 4.63) | <0.001 |
|  | Benin | 0.020  (0.010 to 0.050) | 0.010  (0.000 to 0.030) | -1.25  (-1.44 to -1.05) | <0.001 | 0.008  (0.003 to 0.017) | 0.005  (0.002 to 0.012) | -1.24  (-1.44 to -1.04) | <0.001 |
|  | Bermuda | 0.010  (0.000 to 0.010) | 0.010  (0.010 to 0.010) | 1.05  (-0.01 to 2.12) | 0.053 | 0.002  (0.001 to 0.003) | 0.003  (0.002 to 0.004) | 1.03  (-0.02 to 2.09) | 0.055 |
|  | Bhutan | 0.070  (0.020 to 0.180) | 0.040  (0.010 to 0.100) | -1.75  (-2.17 to -1.32) | <0.001 | 0.025  (0.007 to 0.062) | 0.015  (0.005 to 0.035) | -1.72  (-2.15 to -1.29) | <0.001 |
|  | Bolivia (Plurinational State of) | 0.030  (0.010 to 0.060) | 0.010  (0.010 to 0.030) | -2.17  (-2.35 to -1.99) | <0.001 | 0.010  (0.004 to 0.022) | 0.005  (0.002 to 0.012) | -2.18  (-2.36 to -2) | <0.001 |
|  | Bosnia and Herzegovina | 0.000  (0.000 to 0.000) | 0.000  (0.000 to 0.000) | -2.08  (-3.06 to -1.09) | <0.001 | 0.001  (0.001 to 0.002) | 0.001  (0.000 to 0.001) | -2.03  (-3 to -1.06) | <0.001 |
|  | Botswana | 0.090  (0.030 to 0.210) | 0.040  (0.010 to 0.100) | -3.13  (-3.84 to -2.41) | <0.001 | 0.031  (0.011 to 0.071) | 0.012  (0.004 to 0.033) | -3.11  (-3.84 to -2.39) | <0.001 |
|  | Brazil | 0.040  (0.030 to 0.040) | 0.030  (0.030 to 0.030) | -0.46  (-0.85 to -0.08) | 0.019 | 0.012  (0.011 to 0.013) | 0.010  (0.009 to 0.011) | -0.48  (-0.84 to -0.12) | 0.01 |
|  | Brunei Darussalam | 0.010  (0.000 to 0.020) | 0.000  (0.000 to 0.010) | -1.41  (-1.7 to -1.13) | <0.001 | 0.003  (0.001 to 0.006) | 0.002  (0.001 to 0.004) | -1.53  (-1.82 to -1.25) | <0.001 |
|  | Bulgaria | 0.000  (0.000 to 0.010) | 0.000  (0.000 to 0.010) | -1.01  (-1.85 to -0.16) | 0.021 | 0.002  (0.001 to 0.003) | 0.001  (0.001 to 0.002) | -1.09  (-1.91 to -0.26) | 0.01 |
|  | Burkina Faso | 0.030  (0.010 to 0.070) | 0.020  (0.010 to 0.040) | -1.47  (-1.7 to -1.23) | <0.001 | 0.011  (0.003 to 0.025) | 0.007  (0.002 to 0.016) | -1.48  (-1.71 to -1.24) | <0.001 |
|  | Burundi | 0.150  (0.050 to 0.420) | 0.080  (0.020 to 0.270) | -2.14  (-2.4 to -1.87) | <0.001 | 0.050  (0.017 to 0.143) | 0.026  (0.007 to 0.091) | -2.13  (-2.39 to -1.86) | <0.001 |
|  | Cabo Verde | 0.010  (0.000 to 0.030) | 0.000  (0.000 to 0.010) | -3.76  (-4.41 to -3.11) | <0.001 | 0.005  (0.002 to 0.010) | 0.001  (0.001 to 0.003) | -3.71  (-4.33 to -3.08) | <0.001 |
|  | Cambodia | 0.010  (0.000 to 0.020) | 0.010  (0.000 to 0.020) | 0.08  (-0.07 to 0.24) | 0.303 | 0.003  (0.001 to 0.008) | 0.003  (0.001 to 0.008) | 0.07  (-0.08 to 0.23) | 0.356 |
|  | Cameroon | 0.050  (0.010 to 0.130) | 0.030  (0.010 to 0.060) | -2.09  (-2.24 to -1.95) | <0.001 | 0.019  (0.003 to 0.047) | 0.010  (0.003 to 0.023) | -2.1  (-2.24 to -1.96) | <0.001 |
|  | Canada | 0.010  (0.000 to 0.010) | 0.000  (0.000 to 0.000) | -1.71  (-2.16 to -1.26) | <0.001 | 0.002  (0.002 to 0.003) | 0.001  (0.001 to 0.002) | -1.68  (-2.13 to -1.23) | <0.001 |
|  | Central African Republic | 0.100  (0.030 to 0.230) | 0.090  (0.030 to 0.200) | -0.57  (-0.91 to -0.23) | 0.001 | 0.035  (0.009 to 0.078) | 0.030  (0.010 to 0.068) | -0.56  (-0.9 to -0.22) | 0.001 |
|  | Chad | 0.030  (0.010 to 0.080) | 0.030  (0.010 to 0.070) | -0.4  (-0.72 to -0.08) | 0.013 | 0.012  (0.003 to 0.031) | 0.010  (0.003 to 0.024) | -0.41  (-0.73 to -0.09) | 0.012 |
|  | Chile | 0.010  (0.010 to 0.020) | 0.010  (0.010 to 0.010) | -0.85  (-1.43 to -0.25) | 0.005 | 0.005  (0.004 to 0.007) | 0.004  (0.003 to 0.005) | -0.82  (-1.38 to -0.25) | 0.005 |
|  | China | 0.010  (0.000 to 0.010) | 0.000  (0.000 to 0.000) | -4.65  (-5.44 to -3.85) | <0.001 | 0.003  (0.001 to 0.005) | 0.001  (0.000 to 0.001) | -4.64  (-5.55 to -3.72) | <0.001 |
|  | Colombia | 0.030  (0.020 to 0.040) | 0.030  (0.020 to 0.040) | -0.52  (-0.91 to -0.12) | 0.011 | 0.011  (0.008 to 0.014) | 0.009  (0.006 to 0.013) | -0.52  (-0.91 to -0.12) | 0.012 |
|  | Comoros | 0.210  (0.050 to 0.520) | 0.110  (0.040 to 0.290) | -2.13  (-5.1 to 0.94) | 0.172 | 0.071  (0.018 to 0.176) | 0.038  (0.013 to 0.099) | -2.13  (-5.02 to 0.84) | 0.158 |
|  | Congo | 0.080  (0.020 to 0.180) | 0.050  (0.020 to 0.110) | -1.51  (-2.25 to -0.76) | <0.001 | 0.025  (0.007 to 0.059) | 0.016  (0.005 to 0.036) | -1.49  (-2.24 to -0.73) | <0.001 |
|  | Cook Islands | 0.000  (0.000 to 0.010) | 0.000  (0.000 to 0.000) | -3.18  (-3.38 to -2.98) | <0.001 | 0.001  (0.000 to 0.003) | 0.000  (0.000 to 0.001) | -3.18  (-3.38 to -2.98) | <0.001 |
|  | Costa Rica | 0.020  (0.010 to 0.020) | 0.020  (0.020 to 0.030) | 0.54  (-0.28 to 1.38) | 0.198 | 0.006  (0.005 to 0.008) | 0.007  (0.005 to 0.010) | 0.59  (-0.22 to 1.41) | 0.152 |
|  | Coted'Ivoire | 0.030  (0.010 to 0.070) | 0.020  (0.010 to 0.050) | -0.97  (-1.57 to -0.36) | 0.002 | 0.010  (0.003 to 0.024) | 0.008  (0.002 to 0.017) | -0.97  (-1.57 to -0.36) | 0.002 |
|  | Croatia | 0.000  (0.000 to 0.000) | 0.000  (0.000 to 0.000) | -2.46  (-2.93 to -1.98) | <0.001 | 0.001  (0.001 to 0.002) | 0.001  (0.000 to 0.001) | -2.48  (-2.94 to -2.02) | <0.001 |
|  | Cuba | 0.030  (0.020 to 0.040) | 0.050  (0.040 to 0.080) | 2.42  (0.96 to 3.9) | 0.001 | 0.009  (0.007 to 0.012) | 0.018  (0.013 to 0.025) | 2.39  (0.92 to 3.87) | 0.001 |
|  | Cyprus | 0.000  (0.000 to 0.010) | 0.000  (0.000 to 0.000) | -3.75  (-4.58 to -2.92) | <0.001 | 0.002  (0.001 to 0.004) | 0.001  (0.000 to 0.001) | -3.82  (-5.55 to -2.05) | <0.001 |
|  | Czechia | 0.010  (0.010 to 0.010) | 0.000  (0.000 to 0.010) | -1.85  (-2.39 to -1.3) | <0.001 | 0.003  (0.003 to 0.004) | 0.002  (0.001 to 0.003) | -1.84  (-2.37 to -1.3) | <0.001 |
|  | Democratic People's Republic of Korea | 0.000  (0.000 to 0.010) | 0.000  (0.000 to 0.010) | 0.37  (0.21 to 0.53) | <0.001 | 0.001  (0.000 to 0.003) | 0.001  (0.000 to 0.003) | 0.37  (0.22 to 0.52) | <0.001 |
|  | Democratic Republic of the Congo | 0.080  (0.020 to 0.190) | 0.040  (0.010 to 0.100) | -1.98  (-2.43 to -1.53) | <0.001 | 0.026  (0.008 to 0.064) | 0.014  (0.005 to 0.035) | -1.98  (-2.43 to -1.53) | <0.001 |
|  | Denmark | 0.010  (0.010 to 0.010) | 0.000  (0.000 to 0.010) | -2.65  (-3.3 to -2) | <0.001 | 0.004  (0.003 to 0.005) | 0.002  (0.001 to 0.002) | -2.57  (-3.43 to -1.7) | <0.001 |
|  | Djibouti | 0.080  (0.020 to 0.250) | 0.070  (0.020 to 0.230) | -0.44  (-0.9 to 0.02) | 0.059 | 0.028  (0.008 to 0.085) | 0.023  (0.006 to 0.078) | -0.45  (-0.9 to 0) | 0.049 |
|  | Dominica | 0.000  (0.000 to 0.000) | 0.000  (0.000 to 0.010) | 1.74  (1.24 to 2.25) | <0.001 | 0.001  (0.000 to 0.001) | 0.001  (0.000 to 0.002) | 1.78  (1.23 to 2.33) | <0.001 |
|  | Dominican Republic | 0.080  (0.040 to 0.150) | 0.080  (0.030 to 0.170) | -0.25  (-0.79 to 0.3) | 0.369 | 0.027  (0.013 to 0.050) | 0.026  (0.009 to 0.055) | -0.22  (-0.72 to 0.28) | 0.389 |
|  | Ecuador | 0.010  (0.010 to 0.010) | 0.010  (0.000 to 0.010) | 0.19  (-1.38 to 1.78) | 0.816 | 0.002  (0.002 to 0.003) | 0.003  (0.001 to 0.005) | 0.18  (-1.38 to 1.77) | 0.82 |
|  | Egypt | 0.010  (0.000 to 0.020) | 0.000  (0.000 to 0.010) | -2.32  (-3.08 to -1.55) | <0.001 | 0.003  (0.001 to 0.007) | 0.001  (0.001 to 0.003) | -2.35  (-3.11 to -1.58) | <0.001 |
|  | El Salvador | 0.010  (0.010 to 0.010) | 0.010  (0.000 to 0.010) | -0.77  (-2.05 to 0.52) | 0.239 | 0.003  (0.002 to 0.005) | 0.002  (0.001 to 0.004) | -0.75  (-2.01 to 0.52) | 0.247 |
|  | Equatorial Guinea | 0.100  (0.030 to 0.250) | 0.040  (0.010 to 0.100) | -2.86  (-3.5 to -2.2) | <0.001 | 0.035  (0.009 to 0.084) | 0.014  (0.004 to 0.034) | -2.84  (-3.49 to -2.19) | <0.001 |
|  | Eritrea | 0.150  (0.040 to 0.460) | 0.100  (0.030 to 0.290) | -1.47  (-1.76 to -1.18) | <0.001 | 0.051  (0.015 to 0.156) | 0.033  (0.010 to 0.099) | -1.46  (-1.74 to -1.17) | <0.001 |
|  | Estonia | 0.040  (0.030 to 0.050) | 0.020  (0.010 to 0.030) | -2.85  (-3.67 to -2.02) | <0.001 | 0.016  (0.012 to 0.021) | 0.007  (0.005 to 0.011) | -2.83  (-3.74 to -1.91) | <0.001 |
|  | Eswatini | 0.090  (0.030 to 0.190) | 0.050  (0.010 to 0.130) | -1.74  (-2.16 to -1.32) | <0.001 | 0.030  (0.011 to 0.065) | 0.017  (0.005 to 0.045) | -1.75  (-2.17 to -1.33) | <0.001 |
|  | Ethiopia | 0.350  (0.110 to 0.730) | 0.100  (0.040 to 0.360) | -3.81  (-4.01 to -3.62) | <0.001 | 0.120  (0.037 to 0.248) | 0.035  (0.013 to 0.124) | -3.82  (-4.01 to -3.63) | <0.001 |
|  | Fiji | 0.020  (0.010 to 0.050) | 0.010  (0.000 to 0.030) | -1.75  (-2.77 to -0.71) | 0.001 | 0.007  (0.003 to 0.018) | 0.004  (0.001 to 0.010) | -1.74  (-2.77 to -0.7) | 0.001 |
|  | Finland | 0.010  (0.010 to 0.020) | 0.010  (0.000 to 0.010) | -2.75  (-3.3 to -2.2) | <0.001 | 0.006  (0.004 to 0.007) | 0.002  (0.002 to 0.003) | -2.7  (-3.22 to -2.17) | <0.001 |
|  | France | 0.000  (0.000 to 0.010) | 0.000  (0.000 to 0.000) | -2.91  (-3.41 to -2.4) | <0.001 | 0.002  (0.001 to 0.002) | 0.001  (0.000 to 0.001) | -3.05  (-3.5 to -2.61) | <0.001 |
|  | Gabon | 0.060  (0.020 to 0.140) | 0.030  (0.010 to 0.070) | -2.46  (-2.76 to -2.16) | <0.001 | 0.019  (0.006 to 0.046) | 0.009  (0.003 to 0.023) | -2.48  (-2.78 to -2.17) | <0.001 |
|  | Gambia | 0.030  (0.010 to 0.060) | 0.020  (0.010 to 0.060) | -0.24  (-2.03 to 1.59) | 0.797 | 0.009  (0.002 to 0.022) | 0.009  (0.003 to 0.021) | -0.24  (-2.03 to 1.59) | 0.798 |
|  | Georgia | 0.020  (0.010 to 0.020) | 0.000  (0.000 to 0.010) | -4.77  (-5.75 to -3.77) | <0.001 | 0.007  (0.005 to 0.009) | 0.002  (0.001 to 0.003) | -4.79  (-5.74 to -3.84) | <0.001 |
|  | Germany | 0.000  (0.000 to 0.010) | 0.000  (0.000 to 0.000) | -3.22  (-3.76 to -2.67) | <0.001 | 0.002  (0.001 to 0.002) | 0.001  (0.000 to 0.001) | -3.22  (-3.74 to -2.69) | <0.001 |
|  | Ghana | 0.040  (0.010 to 0.090) | 0.020  (0.010 to 0.040) | -2.18  (-2.46 to -1.9) | <0.001 | 0.014  (0.004 to 0.031) | 0.007  (0.002 to 0.016) | -2.21  (-2.53 to -1.88) | <0.001 |
|  | Greece | 0.000  (0.000 to 0.000) | 0.000  (0.000 to 0.000) | -2.18  (-2.75 to -1.59) | <0.001 | 0.001  (0.000 to 0.001) | 0.000  (0.000 to 0.000) | -2.24  (-2.85 to -1.63) | <0.001 |
|  | Greenland | 0.000  (0.000 to 0.000) | 0.000  (0.000 to 0.000) | 0.59  (-0.88 to 2.08) | 0.434 | 0.000  (0.000 to 0.000) | 0.000  (0.000 to 0.000) | 0.48  (-0.9 to 1.87) | 0.497 |
|  | Grenada | 0.020  (0.020 to 0.030) | 0.040  (0.030 to 0.060) | 1.94  (0.36 to 3.55) | 0.016 | 0.008  (0.005 to 0.011) | 0.013  (0.009 to 0.018) | 1.88  (0.28 to 3.5) | 0.021 |
|  | Guam | 0.000  (0.000 to 0.000) | 0.000  (0.000 to 0.000) | -3.27  (-6.34 to -0.11) | 0.043 | 0.001  (0.000 to 0.002) | 0.000  (0.000 to 0.001) | -3.22  (-6.35 to 0.01) | 0.051 |
|  | Guatemala | 0.030  (0.030 to 0.040) | 0.030  (0.020 to 0.040) | -0.54  (-1.21 to 0.13) | 0.113 | 0.011  (0.008 to 0.015) | 0.009  (0.006 to 0.012) | -0.83  (-1.89 to 0.25) | 0.133 |
|  | Guinea | 0.040  (0.010 to 0.090) | 0.030  (0.010 to 0.060) | -0.9  (-1.28 to -0.51) | <0.001 | 0.013  (0.004 to 0.032) | 0.010  (0.003 to 0.023) | -0.91  (-1.3 to -0.51) | <0.001 |
|  | Guinea-Bissau | 0.080  (0.010 to 0.210) | 0.040  (0.010 to 0.110) | -1.75  (-1.91 to -1.59) | <0.001 | 0.029  (0.004 to 0.075) | 0.016  (0.005 to 0.039) | -1.76  (-1.91 to -1.6) | <0.001 |
|  | Guyana | 0.040  (0.030 to 0.060) | 0.080  (0.050 to 0.120) | 2.38  (0.19 to 4.61) | 0.033 | 0.014  (0.009 to 0.020) | 0.026  (0.015 to 0.042) | 2.21  (0.2 to 4.27) | 0.031 |
|  | Haiti | 0.110  (0.040 to 0.250) | 0.100  (0.030 to 0.240) | -0.08  (-0.41 to 0.25) | 0.649 | 0.036  (0.012 to 0.083) | 0.035  (0.009 to 0.081) | 0.04  (-0.18 to 0.26) | 0.727 |
|  | Honduras | 0.090  (0.040 to 0.200) | 0.040  (0.010 to 0.110) | -2.49  (-2.83 to -2.14) | <0.001 | 0.032  (0.013 to 0.068) | 0.015  (0.005 to 0.036) | -2.52  (-2.87 to -2.18) | <0.001 |
|  | Hungary | 0.030  (0.020 to 0.040) | 0.010  (0.010 to 0.020) | -2.57  (-3.34 to -1.8) | <0.001 | 0.011  (0.008 to 0.014) | 0.005  (0.003 to 0.007) | -2.54  (-3.31 to -1.77) | <0.001 |
|  | Iceland | 0.000  (0.000 to 0.000) | 0.000  (0.000 to 0.000) | -2.16  (-3.78 to -0.51) | 0.011 | 0.001  (0.001 to 0.002) | 0.001  (0.000 to 0.001) | -2.29  (-3.17 to -1.41) | <0.001 |
|  | India | 0.100  (0.040 to 0.140) | 0.050  (0.020 to 0.070) | -2.43  (-2.93 to -1.92) | <0.001 | 0.034  (0.015 to 0.048) | 0.016  (0.008 to 0.023) | -2.44  (-2.95 to -1.93) | <0.001 |
|  | Indonesia | 0.010  (0.000 to 0.010) | 0.000  (0.000 to 0.010) | -2.33  (-2.43 to -2.23) | <0.001 | 0.002  (0.001 to 0.003) | 0.001  (0.001 to 0.002) | -2.32  (-2.41 to -2.22) | <0.001 |
|  | Iran (Islamic Republic of) | 0.000  (0.000 to 0.000) | 0.000  (0.000 to 0.000) | 0.6  (0.07 to 1.13) | 0.026 | 0.000  (0.000 to 0.001) | 0.000  (0.000 to 0.001) | 0.44  (-0.25 to 1.14) | 0.21 |
|  | Iraq | 0.010  (0.000 to 0.010) | 0.000  (0.000 to 0.010) | -2.42  (-2.59 to -2.24) | <0.001 | 0.002  (0.001 to 0.005) | 0.001  (0.000 to 0.002) | -2.5  (-2.68 to -2.31) | <0.001 |
|  | Ireland | 0.000  (0.000 to 0.010) | 0.000  (0.000 to 0.000) | -3.23  (-3.8 to -2.66) | <0.001 | 0.002  (0.001 to 0.002) | 0.001  (0.000 to 0.001) | -3.31  (-3.96 to -2.67) | <0.001 |
|  | Israel | 0.000  (0.000 to 0.000) | 0.000  (0.000 to 0.000) | -3.38  (-4.06 to -2.7) | <0.001 | 0.001  (0.001 to 0.001) | 0.000  (0.000 to 0.000) | -3.4  (-4.01 to -2.78) | <0.001 |
|  | Italy | 0.000  (0.000 to 0.000) | 0.000  (0.000 to 0.000) | -2.02  (-2.91 to -1.13) | <0.001 | 0.001  (0.001 to 0.001) | 0.000  (0.000 to 0.001) | -2.08  (-2.97 to -1.18) | <0.001 |
|  | Jamaica | 0.020  (0.010 to 0.020) | 0.030  (0.020 to 0.050) | 1.82  (-0.12 to 3.79) | 0.066 | 0.006  (0.004 to 0.008) | 0.010  (0.006 to 0.015) | 1.85  (-0.14 to 3.89) | 0.069 |
|  | Japan | 0.000  (0.000 to 0.000) | 0.000  (0.000 to 0.000) | 1.28  (0.71 to 1.86) | <0.001 | 0.000  (0.000 to 0.000) | 0.001  (0.001 to 0.001) | 1.34  (0.77 to 1.91) | <0.001 |
|  | Jordan | 0.000  (0.000 to 0.000) | 0.000  (0.000 to 0.000) | -2.46  (-3.45 to -1.46) | <0.001 | 0.000  (0.000 to 0.001) | 0.000  (0.000 to 0.001) | -2.45  (-3.25 to -1.64) | <0.001 |
|  | Kazakhstan | 0.040  (0.030 to 0.050) | 0.010  (0.010 to 0.020) | -3.67  (-4.91 to -2.43) | <0.001 | 0.015  (0.012 to 0.020) | 0.005  (0.004 to 0.006) | -3.86  (-5.13 to -2.57) | <0.001 |
|  | Kenya | 0.090  (0.050 to 0.170) | 0.070  (0.030 to 0.140) | -1.17  (-1.57 to -0.77) | <0.001 | 0.033  (0.015 to 0.059) | 0.023  (0.011 to 0.049) | -1.16  (-1.56 to -0.76) | <0.001 |
|  | Kiribati | 0.010  (0.000 to 0.020) | 0.010  (0.000 to 0.020) | -0.74  (-0.84 to -0.63) | <0.001 | 0.003  (0.001 to 0.007) | 0.002  (0.001 to 0.006) | -0.73  (-0.84 to -0.63) | <0.001 |
|  | Kuwait | 0.000  (0.000 to 0.000) | 0.000  (0.000 to 0.010) | 11.72  (6.94 to 16.71) | <0.001 | 0.000  (0.000 to 0.000) | 0.001  (0.001 to 0.002) | 13.12  (8.54 to 17.89) | <0.001 |
|  | Kyrgyzstan | 0.050  (0.030 to 0.060) | 0.010  (0.010 to 0.010) | -5.12  (-6.16 to -4.08) | <0.001 | 0.019  (0.014 to 0.025) | 0.004  (0.003 to 0.005) | -5.15  (-6.41 to -3.87) | <0.001 |
|  | Lao People's Democratic Republic | 0.010  (0.000 to 0.030) | 0.010  (0.000 to 0.030) | 0.71  (0.49 to 0.93) | <0.001 | 0.003  (0.001 to 0.010) | 0.004  (0.001 to 0.010) | 0.72  (0.5 to 0.94) | <0.001 |
|  | Latvia | 0.050  (0.040 to 0.070) | 0.030  (0.020 to 0.040) | -2.31  (-2.96 to -1.65) | <0.001 | 0.021  (0.016 to 0.028) | 0.010  (0.006 to 0.016) | -2.38  (-3.05 to -1.71) | <0.001 |
|  | Lebanon | 0.010  (0.000 to 0.020) | 0.000  (0.000 to 0.010) | -3.45  (-3.75 to -3.14) | <0.001 | 0.003  (0.001 to 0.006) | 0.001  (0.000 to 0.002) | -3.47  (-3.82 to -3.12) | <0.001 |
|  | Lesotho | 0.050  (0.020 to 0.130) | 0.060  (0.020 to 0.140) | 0.45  (-0.06 to 0.96) | 0.082 | 0.017  (0.006 to 0.044) | 0.020  (0.007 to 0.048) | 0.44  (-0.06 to 0.95) | 0.087 |
|  | Liberia | 0.050  (0.010 to 0.110) | 0.040  (0.010 to 0.110) | -0.52  (-0.91 to -0.13) | 0.009 | 0.017  (0.004 to 0.041) | 0.014  (0.002 to 0.039) | -0.54  (-0.93 to -0.15) | 0.006 |
|  | Libya | 0.000  (0.000 to 0.000) | 0.000  (0.000 to 0.000) | 0.25  (-0.32 to 0.83) | 0.39 | 0.000  (0.000 to 0.001) | 0.000  (0.000 to 0.001) | 0.24  (-0.22 to 0.71) | 0.299 |
|  | Lithuania | 0.020  (0.020 to 0.030) | 0.010  (0.010 to 0.020) | -2.1  (-3.13 to -1.06) | <0.001 | 0.008  (0.006 to 0.010) | 0.004  (0.003 to 0.008) | -2.14  (-3.11 to -1.15) | <0.001 |
|  | Luxembourg | 0.000  (0.000 to 0.010) | 0.000  (0.000 to 0.000) | -3.83  (-4.66 to -3) | <0.001 | 0.002  (0.001 to 0.003) | 0.001  (0.000 to 0.001) | -3.72  (-4.49 to -2.94) | <0.001 |
|  | Madagascar | 0.230  (0.060 to 0.520) | 0.170  (0.060 to 0.370) | -0.96  (-1.29 to -0.62) | <0.001 | 0.078  (0.022 to 0.179) | 0.058  (0.021 to 0.125) | -0.97  (-1.42 to -0.51) | <0.001 |
|  | Malawi | 0.150  (0.050 to 0.340) | 0.110  (0.040 to 0.250) | -1.15  (-1.63 to -0.67) | <0.001 | 0.053  (0.018 to 0.119) | 0.036  (0.013 to 0.085) | -1.15  (-1.64 to -0.67) | <0.001 |
|  | Malaysia | 0.010  (0.000 to 0.020) | 0.010  (0.000 to 0.010) | -1.68  (-2.44 to -0.91) | <0.001 | 0.003  (0.001 to 0.007) | 0.002  (0.001 to 0.004) | -1.61  (-2.13 to -1.09) | <0.001 |
|  | Maldives | 0.060  (0.020 to 0.130) | 0.020  (0.010 to 0.030) | -4.53  (-4.94 to -4.12) | <0.001 | 0.020  (0.007 to 0.044) | 0.005  (0.002 to 0.011) | -4.55  (-4.96 to -4.14) | <0.001 |
|  | Mali | 0.060  (0.010 to 0.140) | 0.040  (0.010 to 0.090) | -1.25  (-1.49 to -1.01) | <0.001 | 0.021  (0.005 to 0.050) | 0.014  (0.004 to 0.034) | -1.26  (-1.51 to -1.02) | <0.001 |
|  | Malta | 0.000  (0.000 to 0.000) | 0.000  (0.000 to 0.000) | -2.27  (-3.14 to -1.4) | <0.001 | 0.001  (0.001 to 0.001) | 0.000  (0.000 to 0.001) | -2.09  (-3.54 to -0.62) | 0.005 |
|  | Marshall Islands | 0.010  (0.000 to 0.020) | 0.010  (0.000 to 0.010) | -0.35  (-0.62 to -0.08) | 0.012 | 0.002  (0.001 to 0.005) | 0.002  (0.001 to 0.005) | -0.34  (-0.61 to -0.07) | 0.013 |
|  | Mauritania | 0.030  (0.010 to 0.090) | 0.020  (0.000 to 0.040) | -2.23  (-2.42 to -2.04) | <0.001 | 0.012  (0.003 to 0.032) | 0.006  (0.002 to 0.015) | -2.23  (-2.42 to -2.04) | <0.001 |
|  | Mauritius | 0.000  (0.000 to 0.000) | 0.010  (0.010 to 0.010) | 8.85  (7.44 to 10.28) | <0.001 | 0.000  (0.000 to 0.000) | 0.003  (0.002 to 0.004) | 8.87  (7.44 to 10.31) | <0.001 |
|  | Mexico | 0.030  (0.030 to 0.030) | 0.030  (0.030 to 0.050) | 0.31  (-0.05 to 0.67) | 0.096 | 0.011  (0.010 to 0.012) | 0.011  (0.009 to 0.017) | 0.33  (-0.02 to 0.69) | 0.067 |
|  | Micronesia (Federated States of) | 0.010  (0.000 to 0.020) | 0.010  (0.000 to 0.010) | -1.1  (-1.24 to -0.96) | <0.001 | 0.003  (0.001 to 0.007) | 0.002  (0.001 to 0.005) | -1.1  (-1.24 to -0.96) | <0.001 |
|  | Monaco | 0.000  (0.000 to 0.000) | 0.000  (0.000 to 0.000) | -1.59  (-1.66 to -1.53) | <0.001 | 0.000  (0.000 to 0.001) | 0.000  (0.000 to 0.001) | -1.57  (-1.65 to -1.5) | <0.001 |
|  | Mongolia | 0.110  (0.040 to 0.250) | 0.040  (0.020 to 0.080) | -3.26  (-3.8 to -2.73) | <0.001 | 0.043  (0.015 to 0.102) | 0.016  (0.007 to 0.032) | -3.26  (-3.81 to -2.7) | <0.001 |
|  | Montenegro | 0.000  (0.000 to 0.010) | 0.000  (0.000 to 0.000) | -1.07  (-1.9 to -0.24) | 0.012 | 0.002  (0.001 to 0.003) | 0.001  (0.001 to 0.002) | -1.08  (-1.92 to -0.23) | 0.012 |
|  | Morocco | 0.000  (0.000 to 0.010) | 0.000  (0.000 to 0.010) | -0.67  (-1.39 to 0.05) | 0.068 | 0.001  (0.000 to 0.004) | 0.001  (0.000 to 0.003) | -0.67  (-1.39 to 0.05) | 0.069 |
|  | Mozambique | 0.320  (0.100 to 0.750) | 0.160  (0.050 to 0.380) | -2.35  (-2.62 to -2.09) | <0.001 | 0.112  (0.034 to 0.259) | 0.055  (0.019 to 0.131) | -2.38  (-2.65 to -2.11) | <0.001 |
|  | Myanmar | 0.020  (0.000 to 0.050) | 0.010  (0.010 to 0.030) | -0.13  (-0.29 to 0.03) | 0.099 | 0.005  (0.001 to 0.017) | 0.005  (0.002 to 0.011) | -0.15  (-0.31 to 0) | 0.058 |
|  | Namibia | 0.060  (0.020 to 0.160) | 0.030  (0.010 to 0.090) | -1.97  (-2.68 to -1.26) | <0.001 | 0.020  (0.007 to 0.052) | 0.011  (0.003 to 0.031) | -1.98  (-2.71 to -1.25) | <0.001 |
|  | Nauru | 0.010  (0.000 to 0.020) | 0.010  (0.000 to 0.010) | -0.88  (-1.05 to -0.7) | <0.001 | 0.003  (0.001 to 0.006) | 0.002  (0.001 to 0.004) | -0.87  (-1.04 to -0.7) | <0.001 |
|  | Nepal | 0.080  (0.030 to 0.180) | 0.040  (0.010 to 0.090) | -2.11  (-2.27 to -1.95) | <0.001 | 0.025  (0.009 to 0.061) | 0.013  (0.005 to 0.029) | -2.11  (-2.25 to -1.98) | <0.001 |
|  | Netherlands | 0.000  (0.000 to 0.000) | 0.000  (0.000 to 0.000) | -2.79  (-3.03 to -2.54) | <0.001 | 0.002  (0.001 to 0.002) | 0.001  (0.000 to 0.001) | -2.75  (-3.06 to -2.44) | <0.001 |
|  | New Zealand | 0.010  (0.000 to 0.010) | 0.000  (0.000 to 0.000) | -2.65  (-7.31 to 2.24) | 0.283 | 0.002  (0.002 to 0.003) | 0.001  (0.001 to 0.001) | -2.66  (-7.36 to 2.27) | 0.285 |
|  | Nicaragua | 0.010  (0.010 to 0.030) | 0.010  (0.000 to 0.020) | -1.51  (-2.07 to -0.96) | <0.001 | 0.005  (0.003 to 0.009) | 0.003  (0.002 to 0.005) | -1.5  (-2.07 to -0.93) | <0.001 |
|  | Niger | 0.020  (0.010 to 0.060) | 0.010  (0.000 to 0.040) | -1.85  (-2.09 to -1.62) | <0.001 | 0.007  (0.002 to 0.020) | 0.004  (0.001 to 0.014) | -1.85  (-2.09 to -1.61) | <0.001 |
|  | Nigeria | 0.020  (0.010 to 0.030) | 0.010  (0.000 to 0.030) | -1.41  (-1.55 to -1.27) | <0.001 | 0.007  (0.003 to 0.012) | 0.005  (0.002 to 0.009) | -1.41  (-1.55 to -1.26) | <0.001 |
|  | Niue | 0.000  (0.000 to 0.010) | 0.000  (0.000 to 0.010) | -0.68  (-0.93 to -0.44) | <0.001 | 0.001  (0.000 to 0.003) | 0.001  (0.000 to 0.002) | -0.65  (-0.91 to -0.4) | <0.001 |
|  | North Macedonia | 0.010  (0.000 to 0.020) | 0.000  (0.000 to 0.010) | -2.86  (-3.7 to -2.01) | <0.001 | 0.003  (0.001 to 0.007) | 0.001  (0.000 to 0.004) | -2.89  (-3.77 to -2) | <0.001 |
|  | Northern Mariana Islands | 0.010  (0.000 to 0.020) | 0.000  (0.000 to 0.000) | -7.17  (-9.09 to -5.2) | <0.001 | 0.003  (0.001 to 0.008) | 0.000  (0.000 to 0.002) | -7.11  (-8.84 to -5.35) | <0.001 |
|  | Norway | 0.000  (0.000 to 0.000) | 0.000  (0.000 to 0.000) | 1.38  (-1.3 to 4.14) | 0.316 | 0.000  (0.000 to 0.000) | 0.000  (0.000 to 0.001) | 1.41  (-1.31 to 4.2) | 0.313 |
|  | Oman | 0.000  (0.000 to 0.010) | 0.000  (0.000 to 0.000) | -2.62  (-3.42 to -1.8) | <0.001 | 0.001  (0.000 to 0.002) | 0.000  (0.000 to 0.001) | -2.61  (-3.39 to -1.81) | <0.001 |
|  | Pakistan | 0.030  (0.010 to 0.070) | 0.030  (0.010 to 0.060) | -0.18  (-0.35 to -0.01) | 0.033 | 0.010  (0.004 to 0.022) | 0.009  (0.004 to 0.020) | -0.14  (-0.31 to 0.03) | 0.112 |
|  | Palau | 0.000  (0.000 to 0.010) | 0.000  (0.000 to 0.000) | -1.34  (-1.56 to -1.12) | <0.001 | 0.001  (0.000 to 0.002) | 0.001  (0.000 to 0.002) | -1.33  (-1.54 to -1.12) | <0.001 |
|  | Palestine | 0.000  (0.000 to 0.010) | 0.000  (0.000 to 0.010) | -0.26  (-0.86 to 0.34) | 0.397 | 0.001  (0.000 to 0.003) | 0.001  (0.000 to 0.003) | -0.27  (-0.87 to 0.33) | 0.377 |
|  | Panama | 0.020  (0.020 to 0.030) | 0.020  (0.010 to 0.030) | -0.68  (-1.56 to 0.2) | 0.129 | 0.008  (0.006 to 0.010) | 0.006  (0.004 to 0.009) | -0.68  (-1.6 to 0.24) | 0.146 |
|  | Papua New Guinea | 0.010  (0.000 to 0.020) | 0.010  (0.000 to 0.010) | -1.17  (-1.63 to -0.71) | <0.001 | 0.003  (0.001 to 0.007) | 0.002  (0.001 to 0.005) | -1.17  (-1.63 to -0.7) | <0.001 |
|  | Paraguay | 0.040  (0.030 to 0.070) | 0.040  (0.020 to 0.060) | -0.42  (-1.09 to 0.26) | 0.225 | 0.015  (0.009 to 0.023) | 0.012  (0.007 to 0.020) | -0.46  (-1.17 to 0.25) | 0.206 |
|  | Peru | 0.020  (0.010 to 0.030) | 0.010  (0.000 to 0.020) | -2.11  (-3.48 to -0.73) | 0.003 | 0.006  (0.003 to 0.011) | 0.003  (0.001 to 0.007) | -2.14  (-3.52 to -0.75) | 0.003 |
|  | Philippines | 0.000  (0.000 to 0.010) | 0.010  (0.000 to 0.020) | 3.99  (3.63 to 4.34) | <0.001 | 0.001  (0.001 to 0.003) | 0.004  (0.001 to 0.006) | 3.96  (3.61 to 4.31) | <0.001 |
|  | Poland | 0.010  (0.010 to 0.010) | 0.000  (0.000 to 0.000) | -5.01  (-5.63 to -4.38) | <0.001 | 0.003  (0.003 to 0.004) | 0.001  (0.001 to 0.001) | -4.99  (-5.62 to -4.35) | <0.001 |
|  | Portugal | 0.000  (0.000 to 0.010) | 0.000  (0.000 to 0.000) | -3.56  (-4.39 to -2.73) | <0.001 | 0.002  (0.002 to 0.003) | 0.001  (0.000 to 0.001) | -3.62  (-4.44 to -2.79) | <0.001 |
|  | Puerto Rico | 0.010  (0.010 to 0.010) | 0.020  (0.010 to 0.020) | 1.12  (-0.42 to 2.67) | 0.154 | 0.004  (0.003 to 0.005) | 0.005  (0.003 to 0.007) | 1.06  (-0.49 to 2.63) | 0.18 |
|  | Qatar | 0.010  (0.000 to 0.020) | 0.000  (0.000 to 0.010) | -3.32  (-4.87 to -1.74) | <0.001 | 0.003  (0.001 to 0.008) | 0.001  (0.000 to 0.003) | -3.31  (-4.81 to -1.79) | <0.001 |
|  | Republic of Korea | 0.000  (0.000 to 0.000) | 0.000  (0.000 to 0.000) | -1.88  (-2.37 to -1.38) | <0.001 | 0.001  (0.001 to 0.002) | 0.001  (0.000 to 0.001) | -1.9  (-2.46 to -1.34) | <0.001 |
|  | Republic of Moldova | 0.050  (0.030 to 0.060) | 0.020  (0.020 to 0.030) | -2.47  (-2.92 to -2.01) | <0.001 | 0.018  (0.014 to 0.024) | 0.009  (0.007 to 0.013) | -2.51  (-2.95 to -2.06) | <0.001 |
|  | Romania | 0.010  (0.010 to 0.010) | 0.000  (0.000 to 0.010) | -2.38  (-2.95 to -1.81) | <0.001 | 0.003  (0.002 to 0.004) | 0.001  (0.001 to 0.002) | -2.55  (-3.29 to -1.8) | <0.001 |
|  | Russian Federation | 0.040  (0.030 to 0.040) | 0.030  (0.030 to 0.040) | -0.62  (-1.37 to 0.13) | 0.103 | 0.015  (0.014 to 0.017) | 0.012  (0.010 to 0.015) | -0.72  (-1.44 to 0.01) | 0.054 |
|  | Rwanda | 0.260  (0.090 to 0.640) | 0.080  (0.020 to 0.220) | -3.78  (-4.26 to -3.3) | <0.001 | 0.090  (0.031 to 0.218) | 0.027  (0.008 to 0.075) | -3.78  (-4.26 to -3.31) | <0.001 |
|  | Saint Kitts and Nevis | 0.060  (0.040 to 0.080) | 0.040  (0.020 to 0.050) | -1.27  (-3.1 to 0.6) | 0.182 | 0.020  (0.014 to 0.027) | 0.012  (0.008 to 0.018) | -1.41  (-3.26 to 0.48) | 0.143 |
|  | Saint Lucia | 0.040  (0.030 to 0.060) | 0.060  (0.040 to 0.090) | 1.06  (-0.72 to 2.87) | 0.244 | 0.014  (0.011 to 0.019) | 0.020  (0.014 to 0.029) | 1.31  (-0.39 to 3.05) | 0.133 |
|  | Saint Vincent and the Grenadines | 0.010  (0.010 to 0.010) | 0.020  (0.010 to 0.020) | 1.11  (-0.6 to 2.85) | 0.205 | 0.004  (0.003 to 0.005) | 0.005  (0.004 to 0.007) | 0.96  (-0.57 to 2.51) | 0.218 |
|  | Samoa | 0.000  (0.000 to 0.010) | 0.000  (0.000 to 0.010) | -0.54  (-0.78 to -0.3) | <0.001 | 0.001  (0.000 to 0.004) | 0.001  (0.000 to 0.003) | -0.54  (-0.78 to -0.31) | <0.001 |
|  | San Marino | 0.000  (0.000 to 0.000) | 0.000  (0.000 to 0.000) | -2.1  (-2.31 to -1.89) | <0.001 | 0.000  (0.000 to 0.001) | 0.000  (0.000 to 0.000) | -2.08  (-2.3 to -1.86) | <0.001 |
|  | Sao Tome and Principe | 0.020  (0.000 to 0.050) | 0.010  (0.000 to 0.030) | -1.71  (-3.8 to 0.42) | 0.115 | 0.007  (0.002 to 0.018) | 0.005  (0.002 to 0.012) | -1.74  (-3.79 to 0.37) | 0.105 |
|  | Saudi Arabia | 0.000  (0.000 to 0.010) | 0.000  (0.000 to 0.000) | -2.09  (-2.57 to -1.61) | <0.001 | 0.001  (0.000 to 0.002) | 0.000  (0.000 to 0.001) | -2.09  (-2.56 to -1.61) | <0.001 |
|  | Senegal | 0.020  (0.010 to 0.060) | 0.010  (0.000 to 0.030) | -1.84  (-2.4 to -1.27) | <0.001 | 0.009  (0.003 to 0.021) | 0.005  (0.002 to 0.012) | -1.85  (-2.42 to -1.28) | <0.001 |
|  | Serbia | 0.010  (0.000 to 0.010) | 0.000  (0.000 to 0.000) | -2.51  (-2.79 to -2.22) | <0.001 | 0.002  (0.001 to 0.004) | 0.001  (0.001 to 0.002) | -2.55  (-2.83 to -2.27) | <0.001 |
|  | Seychelles | 0.060  (0.030 to 0.100) | 0.020  (0.010 to 0.030) | -3.42  (-3.97 to -2.86) | <0.001 | 0.021  (0.010 to 0.034) | 0.007  (0.004 to 0.012) | -3.41  (-3.96 to -2.85) | <0.001 |
|  | Sierra Leone | 0.020  (0.010 to 0.050) | 0.020  (0.010 to 0.040) | -0.15  (-0.6 to 0.31) | 0.528 | 0.007  (0.002 to 0.017) | 0.007  (0.002 to 0.016) | -0.15  (-0.6 to 0.3) | 0.516 |
|  | Singapore | 0.000  (0.000 to 0.000) | 0.000  (0.000 to 0.000) | -1.94  (-2.8 to -1.07) | <0.001 | 0.001  (0.001 to 0.001) | 0.001  (0.000 to 0.001) | -1.84  (-2.69 to -0.99) | <0.001 |
|  | Slovakia | 0.010  (0.010 to 0.010) | 0.010  (0.000 to 0.010) | -1.87  (-2.68 to -1.05) | <0.001 | 0.004  (0.002 to 0.006) | 0.002  (0.001 to 0.004) | -1.8  (-2.65 to -0.94) | <0.001 |
|  | Slovenia | 0.010  (0.000 to 0.010) | 0.000  (0.000 to 0.000) | -3.47  (-4.99 to -1.93) | <0.001 | 0.003  (0.002 to 0.003) | 0.001  (0.001 to 0.002) | -3.45  (-4.1 to -2.79) | <0.001 |
|  | Solomon Islands | 0.010  (0.000 to 0.020) | 0.010  (0.000 to 0.020) | -0.3  (-0.63 to 0.02) | 0.064 | 0.003  (0.001 to 0.007) | 0.002  (0.001 to 0.006) | -0.29  (-0.61 to 0.03) | 0.077 |
|  | Somalia | 0.210  (0.060 to 0.620) | 0.170  (0.050 to 0.490) | -0.6  (-0.75 to -0.45) | <0.001 | 0.072  (0.021 to 0.213) | 0.059  (0.018 to 0.168) | -0.6  (-0.74 to -0.45) | <0.001 |
|  | South Africa | 0.180  (0.080 to 0.260) | 0.040  (0.030 to 0.070) | -4.32  (-5.75 to -2.87) | <0.001 | 0.061  (0.028 to 0.089) | 0.015  (0.010 to 0.024) | -4.42  (-5.93 to -2.89) | <0.001 |
|  | South Sudan | 0.160  (0.050 to 0.450) | 0.180  (0.060 to 0.520) | 0.31  (-0.58 to 1.22) | 0.496 | 0.056  (0.017 to 0.155) | 0.063  (0.019 to 0.179) | 0.32  (-0.58 to 1.22) | 0.489 |
|  | Spain | 0.010  (0.000 to 0.010) | 0.000  (0.000 to 0.000) | -3.34  (-3.86 to -2.81) | <0.001 | 0.002  (0.002 to 0.003) | 0.001  (0.001 to 0.001) | -3.42  (-3.97 to -2.86) | <0.001 |
|  | Sri Lanka | 0.020  (0.010 to 0.030) | 0.010  (0.000 to 0.020) | -2.31  (-2.77 to -1.85) | <0.001 | 0.007  (0.004 to 0.011) | 0.003  (0.002 to 0.006) | -2.34  (-2.8 to -1.88) | <0.001 |
|  | Sudan | 0.000  (0.000 to 0.010) | 0.000  (0.000 to 0.010) | -0.52  (-0.78 to -0.26) | <0.001 | 0.001  (0.000 to 0.004) | 0.001  (0.000 to 0.003) | -0.52  (-0.78 to -0.26) | <0.001 |
|  | Suriname | 0.020  (0.010 to 0.030) | 0.020  (0.010 to 0.050) | 0.39  (-0.78 to 1.57) | 0.513 | 0.007  (0.003 to 0.012) | 0.007  (0.003 to 0.016) | 0.34  (-0.83 to 1.53) | 0.568 |
|  | Sweden | 0.000  (0.000 to 0.000) | 0.000  (0.000 to 0.000) | -1.01  (-2.53 to 0.54) | 0.201 | 0.001  (0.001 to 0.001) | 0.001  (0.001 to 0.001) | -0.96  (-2.47 to 0.56) | 0.215 |
|  | Switzerland | 0.010  (0.000 to 0.010) | 0.000  (0.000 to 0.000) | -3.2  (-3.9 to -2.5) | <0.001 | 0.002  (0.002 to 0.003) | 0.001  (0.001 to 0.001) | -3.12  (-3.48 to -2.76) | <0.001 |
|  | Syrian Arab Republic | 0.040  (0.020 to 0.060) | 0.020  (0.010 to 0.040) | -2.59  (-3.23 to -1.94) | <0.001 | 0.013  (0.007 to 0.022) | 0.006  (0.003 to 0.015) | -2.63  (-3.3 to -1.96) | <0.001 |
|  | Taiwan (Province of China) | 0.000  (0.000 to 0.000) | 0.000  (0.000 to 0.000) | -1.14  (-2.74 to 0.49) | 0.169 | 0.001  (0.001 to 0.002) | 0.001  (0.001 to 0.001) | -1.18  (-2.76 to 0.42) | 0.147 |
|  | Tajikistan | 0.060  (0.030 to 0.100) | 0.030  (0.010 to 0.070) | -2.82  (-2.99 to -2.64) | <0.001 | 0.025  (0.013 to 0.042) | 0.011  (0.004 to 0.028) | -2.79  (-2.97 to -2.61) | <0.001 |
|  | Thailand | 0.010  (0.000 to 0.020) | 0.010  (0.000 to 0.010) | -0.9  (-1.32 to -0.48) | <0.001 | 0.003  (0.001 to 0.008) | 0.003  (0.001 to 0.005) | -0.76  (-1.21 to -0.32) | 0.001 |
|  | Timor-Leste | 0.010  (0.000 to 0.020) | 0.010  (0.000 to 0.030) | 1.57  (0.59 to 2.57) | 0.002 | 0.002  (0.001 to 0.007) | 0.004  (0.001 to 0.009) | 1.57  (0.6 to 2.55) | 0.001 |
|  | Togo | 0.030  (0.010 to 0.060) | 0.020  (0.010 to 0.050) | -1.44  (-1.75 to -1.13) | <0.001 | 0.011  (0.004 to 0.024) | 0.007  (0.002 to 0.016) | -1.44  (-1.75 to -1.13) | <0.001 |
|  | Tokelau | 0.010  (0.000 to 0.020) | 0.000  (0.000 to 0.010) | -0.92  (-1.1 to -0.74) | <0.001 | 0.002  (0.001 to 0.005) | 0.001  (0.000 to 0.004) | -0.89  (-1.08 to -0.7) | <0.001 |
|  | Tonga | 0.000  (0.000 to 0.010) | 0.000  (0.000 to 0.010) | -1.37  (-1.59 to -1.14) | <0.001 | 0.001  (0.000 to 0.003) | 0.001  (0.000 to 0.002) | -1.37  (-1.61 to -1.13) | <0.001 |
|  | Trinidad and Tobago | 0.020  (0.010 to 0.020) | 0.020  (0.010 to 0.030) | 0.74  (-0.89 to 2.4) | 0.376 | 0.006  (0.004 to 0.008) | 0.007  (0.005 to 0.011) | 0.74  (-0.83 to 2.33) | 0.359 |
|  | Tunisia | 0.000  (0.000 to 0.000) | 0.000  (0.000 to 0.000) | -1.04  (-1.38 to -0.7) | <0.001 | 0.000  (0.000 to 0.001) | 0.000  (0.000 to 0.001) | -1.09  (-1.3 to -0.88) | <0.001 |
|  | Turkmenistan | 0.050  (0.040 to 0.060) | 0.020  (0.010 to 0.030) | -2.79  (-4.12 to -1.44) | <0.001 | 0.021  (0.016 to 0.027) | 0.009  (0.006 to 0.013) | -2.77  (-4.09 to -1.43) | <0.001 |
|  | Tuvalu | 0.010  (0.000 to 0.020) | 0.000  (0.000 to 0.010) | -2.04  (-2.16 to -1.93) | <0.001 | 0.002  (0.001 to 0.007) | 0.001  (0.000 to 0.003) | -2.04  (-2.16 to -1.92) | <0.001 |
|  | Türkiye | 0.001  (0.000 to 0.003) | 0.001  (0.000 to 0.002) | -0.83 (-1.24 to -0.43) | <0.001 | 0.000  (0.00 to 0.001) | 0.000  (0.00 to 0.001) | -0.84 (-1.25 to -0.43) | <0.001 |
|  | Uganda | 0.090  (0.030 to 0.230) | 0.120  (0.040 to 0.260) | 0.8  (0.21 to 1.39) | 0.007 | 0.031  (0.010 to 0.080) | 0.040  (0.014 to 0.090) | 0.8  (0.21 to 1.39) | 0.008 |
|  | Ukraine | 0.020  (0.010 to 0.020) | 0.020  (0.010 to 0.030) | 0.83  (-0.06 to 1.74) | 0.068 | 0.006  (0.005 to 0.008) | 0.008  (0.004 to 0.013) | 0.72  (-0.19 to 1.64) | 0.123 |
|  | United Arab Emirates | 0.020  (0.010 to 0.050) | 0.010  (0.000 to 0.020) | -2.49  (-3.59 to -1.38) | <0.001 | 0.006  (0.002 to 0.018) | 0.003  (0.001 to 0.007) | -2.3  (-3.51 to -1.07) | <0.001 |
|  | United Kingdom | 0.010  (0.010 to 0.010) | 0.000  (0.000 to 0.010) | -1.04  (-1.88 to -0.19) | 0.017 | 0.002  (0.002 to 0.003) | 0.002  (0.002 to 0.002) | -1.1  (-1.96 to -0.23) | 0.013 |
|  | United Republic of Tanzania | 0.190  (0.060 to 0.440) | 0.090  (0.030 to 0.220) | -2.49  (-2.8 to -2.18) | <0.001 | 0.066  (0.021 to 0.151) | 0.030  (0.011 to 0.074) | -2.5  (-2.81 to -2.19) | <0.001 |
|  | United States of America | 0.010  (0.010 to 0.010) | 0.000  (0.000 to 0.010) | -1.6  (-2.21 to -0.98) | <0.001 | 0.003  (0.003 to 0.004) | 0.002  (0.002 to 0.002) | -1.58  (-2.16 to -1) | <0.001 |
|  | United States Virgin Islands | 0.020  (0.010 to 0.050) | 0.020  (0.010 to 0.050) | -0.15  (-1 to 0.7) | 0.726 | 0.008  (0.003 to 0.017) | 0.007  (0.002 to 0.018) | -0.12  (-0.91 to 0.69) | 0.778 |
|  | Uruguay | 0.020  (0.010 to 0.020) | 0.020  (0.010 to 0.020) | -0.28  (-0.86 to 0.31) | 0.353 | 0.008  (0.006 to 0.010) | 0.007  (0.005 to 0.010) | -0.28  (-0.84 to 0.28) | 0.328 |
|  | Uzbekistan | 0.030  (0.020 to 0.040) | 0.010  (0.010 to 0.010) | -4.74  (-5.64 to -3.84) | <0.001 | 0.014  (0.010 to 0.019) | 0.003  (0.002 to 0.005) | -4.72  (-5.62 to -3.81) | <0.001 |
|  | Vanuatu | 0.010  (0.000 to 0.020) | 0.000  (0.000 to 0.010) | -0.58  (-1.03 to -0.12) | 0.013 | 0.002  (0.001 to 0.005) | 0.002  (0.000 to 0.004) | -0.57  (-1.02 to -0.11) | 0.015 |
|  | Venezuela (Bolivarian Republic of) | 0.020  (0.020 to 0.030) | 0.030  (0.020 to 0.040) | 0.72  (-0.01 to 1.46) | 0.055 | 0.007  (0.005 to 0.010) | 0.009  (0.006 to 0.013) | 0.74  (-0.01 to 1.49) | 0.054 |
|  | Viet Nam | 0.000  (0.000 to 0.010) | 0.000  (0.000 to 0.000) | -2.18  (-2.29 to -2.08) | <0.001 | 0.001  (0.000 to 0.002) | 0.000  (0.000 to 0.001) | -2.19  (-2.3 to -2.08) | <0.001 |
|  | Yemen | 0.000  (0.000 to 0.000) | 0.000  (0.000 to 0.000) | 0.74  (-0.21 to 1.71) | 0.128 | 0.000  (0.000 to 0.001) | 0.000  (0.000 to 0.001) | 0.75  (-0.2 to 1.72) | 0.123 |
|  | Zambia | 0.240  (0.070 to 0.540) | 0.150  (0.050 to 0.420) | -1.52  (-1.72 to -1.32) | <0.001 | 0.084  (0.024 to 0.187) | 0.052  (0.016 to 0.145) | -1.53  (-1.73 to -1.33) | <0.001 |
|  | Zimbabwe | 0.040  (0.010 to 0.150) | 0.060  (0.020 to 0.180) | 1.15  (0.08 to 2.22) | 0.035 | 0.014  (0.004 to 0.050) | 0.021  (0.006 to 0.060) | 1.15  (0.27 to 2.04) | 0.01 |
| Age-standardized DALYs | Afghanistan | 2.07  (1.12 to 3.75) | 2.14  (1.18 to 3.70) | 0.1  (0.04 to 0.16) | 0.001 | 0.64  (0.33 to 1.22) | 0.67  (0.36 to 1.17) | 0.11  (0.01 to 0.2) | 0.03 |
|  | Albania | 3.23  (1.71 to 5.39) | 2.93  (1.44 to 5.33) | -0.33  (-0.46 to -0.21) | <0.001 | 1.01  (0.59 to 1.71) | 0.79  (0.42 to 1.39) | -0.81  (-1.01 to -0.62) | <0.001 |
|  | Algeria | 2.05  (1.15 to 3.42) | 1.99  (1.04 to 3.44) | -0.09  (-0.11 to -0.06) | <0.001 | 0.49  (0.26 to 0.88) | 0.48  (0.25 to 0.84) | -0.07  (-0.1 to -0.03) | <0.001 |
|  | American Samoa | 2.52  (1.17 to 4.58) | 2.47  (1.16 to 4.45) | -0.04  (-0.19 to 0.11) | 0.585 | 0.56  (0.28 to 1.03) | 0.50  (0.25 to 0.89) | -0.33  (-0.4 to -0.26) | <0.001 |
|  | Andorra | 1.23  (0.74 to 1.98) | 1.04  (0.59 to 1.74) | -0.55  (-0.66 to -0.44) | <0.001 | 0.39  (0.22 to 0.65) | 0.29  (0.16 to 0.50) | -0.98  (-1.1 to -0.86) | <0.001 |
|  | Angola | 11.28  (5.91 to 19.72) | 7.24  (3.88 to 12.72) | -1.4  (-1.82 to -0.99) | <0.001 | 4.26  (2.21 to 7.46) | 2.60  (1.52 to 4.50) | -1.57  (-1.98 to -1.15) | <0.001 |
|  | Antigua and Barbuda | 3.32  (1.79 to 5.67) | 3.76  (2.19 to 6.18) | 0.4  (-0.02 to 0.82) | 0.06 | 1.13  (0.68 to 1.87) | 1.13  (0.73 to 1.76) | -0.02  (-0.56 to 0.52) | 0.937 |
|  | Argentina | 4.77  (2.59 to 7.96) | 4.57  (2.59 to 7.68) | -0.15  (-0.29 to 0) | 0.049 | 2.38  (1.38 to 3.90) | 2.09  (1.29 to 3.39) | -0.43  (-0.56 to -0.3) | <0.001 |
|  | Armenia | 5.72  (3.76 to 8.57) | 4.16  (2.16 to 7.22) | -1.05  (-1.53 to -0.57) | <0.001 | 2.27  (1.61 to 3.38) | 1.09  (0.62 to 1.83) | -2.42  (-2.92 to -1.91) | <0.001 |
|  | Australia | 6.76  (3.12 to 12.57) | 6.58  (2.86 to 12.08) | -0.11  (-0.23 to 0.01) | 0.078 | 2.95  (1.28 to 5.48) | 2.37  (0.97 to 4.61) | -0.72  (-0.78 to -0.67) | <0.001 |
|  | Austria | 1.06  (0.62 to 1.76) | 0.94  (0.53 to 1.58) | -0.38  (-0.5 to -0.27) | <0.001 | 0.31  (0.19 to 0.51) | 0.21  (0.12 to 0.37) | -1.18  (-1.27 to -1.1) | <0.001 |
|  | Azerbaijan | 7.50  (4.94 to 10.84) | 5.57  (3.18 to 9.15) | -0.98  (-1.16 to -0.79) | <0.001 | 3.02  (2.10 to 4.28) | 1.80  (1.12 to 2.92) | -1.68  (-2.08 to -1.27) | <0.001 |
|  | Bahamas | 3.86  (2.28 to 6.35) | 4.93  (3.15 to 7.41) | 0.79  (-0.15 to 1.74) | 0.098 | 1.25  (0.82 to 1.93) | 1.47  (1.01 to 2.14) | 0.58  (-0.5 to 1.67) | 0.293 |
|  | Bahrain | 2.05  (1.15 to 3.36) | 2.04  (1.15 to 3.38) | -0.03  (-0.07 to 0.01) | 0.165 | 0.59  (0.31 to 1.05) | 0.58  (0.31 to 1.03) | -0.06  (-0.16 to 0.05) | 0.275 |
|  | Bangladesh | 8.15  (4.62 to 13.22) | 4.72  (2.49 to 8.01) | -1.76  (-1.99 to -1.53) | <0.001 | 2.98  (1.53 to 5.02) | 1.36  (0.80 to 2.24) | -2.54  (-2.71 to -2.38) | <0.001 |
|  | Barbados | 4.36  (2.69 to 6.78) | 4.27  (2.66 to 6.70) | 0.01  (-0.42 to 0.44) | 0.965 | 1.46  (0.98 to 2.19) | 1.28  (0.86 to 1.93) | -0.35  (-0.74 to 0.05) | 0.084 |
|  | Belarus | 4.17  (2.49 to 6.52) | 3.98  (2.32 to 6.34) | -0.21  (-0.63 to 0.21) | 0.328 | 1.49  (0.98 to 2.32) | 1.11  (0.71 to 1.74) | -0.98  (-1.37 to -0.59) | <0.001 |
|  | Belgium | 1.24  (0.75 to 2.01) | 1.10  (0.63 to 1.82) | -0.4  (-0.83 to 0.03) | 0.071 | 0.38  (0.24 to 0.62) | 0.24  (0.14 to 0.41) | -1.51  (-1.85 to -1.16) | <0.001 |
|  | Belize | 3.19  (1.61 to 5.59) | 3.75  (2.13 to 6.19) | 0.54  (0.16 to 0.93) | 0.005 | 0.91  (0.53 to 1.53) | 1.02  (0.66 to 1.60) | 0.39  (-0.08 to 0.85) | 0.103 |
|  | Benin | 10.12  (5.65 to 16.63) | 8.50  (4.41 to 14.64) | -0.61  (-0.73 to -0.49) | <0.001 | 3.06  (1.62 to 5.45) | 2.25  (1.10 to 4.07) | -1.04  (-1.23 to -0.85) | <0.001 |
|  | Bermuda | 3.12  (1.56 to 5.50) | 3.25  (1.72 to 5.65) | 0.12  (-0.04 to 0.28) | 0.133 | 0.97  (0.56 to 1.65) | 0.85  (0.51 to 1.39) | -0.41  (-0.57 to -0.25) | <0.001 |
|  | Bhutan | 8.57  (4.25 to 15.86) | 6.66  (3.61 to 11.51) | -0.83  (-1.06 to -0.6) | <0.001 | 2.63  (1.31 to 4.94) | 1.85  (1.03 to 3.25) | -1.17  (-1.43 to -0.91) | <0.001 |
|  | Bolivia (Plurinational State of) | 6.24  (3.34 to 10.56) | 5.39  (2.69 to 9.59) | -0.48  (-0.61 to -0.35) | <0.001 | 2.09  (1.20 to 3.43) | 1.56  (0.89 to 2.55) | -0.98  (-1.11 to -0.85) | <0.001 |
|  | Bosnia and Herzegovina | 2.86  (1.38 to 5.09) | 2.89  (1.41 to 5.23) | 0.06  (0 to 0.13) | 0.06 | 0.82  (0.45 to 1.48) | 0.73  (0.39 to 1.32) | -0.39  (-0.45 to -0.32) | <0.001 |
|  | Botswana | 10.39  (5.53 to 18.63) | 6.97  (3.56 to 12.14) | -1.33  (-1.55 to -1.11) | <0.001 | 3.87  (2.06 to 6.72) | 2.42  (1.37 to 4.20) | -1.55  (-1.87 to -1.22) | <0.001 |
|  | Brazil | 3.56  (2.81 to 4.57) | 6.38  (4.30 to 9.29) | 1.9  (1.72 to 2.09) | <0.001 | 1.08  (0.87 to 1.40) | 1.57  (1.14 to 2.28) | 1.24  (1.04 to 1.45) | <0.001 |
|  | Brunei Darussalam | 5.19  (2.23 to 9.63) | 5.03  (2.09 to 9.69) | -0.11  (-0.16 to -0.06) | <0.001 | 2.42  (1.07 to 4.52) | 2.03  (1.09 to 3.49) | -0.58  (-0.67 to -0.48) | <0.001 |
|  | Bulgaria | 3.02  (1.53 to 5.35) | 3.01  (1.51 to 5.41) | 0.01  (-0.06 to 0.08) | 0.805 | 0.85  (0.48 to 1.49) | 0.74  (0.40 to 1.35) | -0.46  (-0.61 to -0.31) | <0.001 |
|  | Burkina Faso | 15.44  (8.55 to 25.45) | 8.81  (4.62 to 15.26) | -1.85  (-2 to -1.7) | <0.001 | 5.02  (2.63 to 8.76) | 2.46  (1.25 to 4.38) | -2.32  (-2.54 to -2.1) | <0.001 |
|  | Burundi | 13.92  (6.49 to 32.27) | 8.93  (3.84 to 22.34) | -1.51  (-1.74 to -1.27) | <0.001 | 5.01  (2.46 to 11.13) | 3.10  (1.44 to 7.69) | -1.61  (-1.82 to -1.39) | <0.001 |
|  | Cabo Verde | 8.39  (4.42 to 14.39) | 8.07  (4.18 to 14.54) | -0.11  (-0.21 to 0) | 0.045 | 2.17  (1.03 to 4.00) | 1.90  (0.88 to 3.53) | -0.45  (-0.61 to -0.29) | <0.001 |
|  | Cambodia | 1.79  (0.99 to 3.20) | 1.65  (0.91 to 2.93) | -0.26  (-0.34 to -0.19) | <0.001 | 0.41  (0.22 to 0.79) | 0.36  (0.18 to 0.70) | -0.45  (-0.56 to -0.33) | <0.001 |
|  | Cameroon | 16.14  (8.98 to 26.58) | 9.45  (5.02 to 15.86) | -1.77  (-1.96 to -1.57) | <0.001 | 5.32  (2.75 to 9.00) | 2.71  (1.40 to 4.76) | -2.2  (-2.38 to -2.02) | <0.001 |
|  | Canada | 2.37  (1.36 to 3.83) | 2.23  (1.24 to 3.73) | -0.18  (-0.22 to -0.14) | <0.001 | 1.41  (0.80 to 2.39) | 1.22  (0.67 to 2.11) | -0.47  (-0.58 to -0.36) | <0.001 |
|  | Central African Republic | 10.80  (5.46 to 19.02) | 9.60  (4.92 to 17.36) | -0.44  (-0.63 to -0.25) | <0.001 | 4.04  (2.12 to 6.93) | 3.58  (1.90 to 6.25) | -0.47  (-0.66 to -0.28) | <0.001 |
|  | Chad | 12.40  (7.00 to 20.58) | 9.36  (5.11 to 15.94) | -0.91  (-1.11 to -0.71) | <0.001 | 4.12  (2.21 to 6.97) | 2.70  (1.39 to 4.79) | -1.39  (-1.55 to -1.22) | <0.001 |
|  | Chile | 4.41  (2.39 to 7.48) | 4.30  (2.32 to 7.24) | -0.06  (-0.16 to 0.04) | 0.209 | 2.19  (1.24 to 3.73) | 1.80  (1.07 to 2.95) | -0.65  (-0.75 to -0.54) | <0.001 |
|  | China | 1.97  (1.23 to 2.98) | 1.70  (1.01 to 2.75) | -0.49  (-0.62 to -0.36) | <0.001 | 0.52  (0.32 to 0.83) | 0.34  (0.20 to 0.58) | -1.41  (-1.53 to -1.29) | <0.001 |
|  | Colombia | 4.57  (3.00 to 6.76) | 4.04  (2.57 to 6.14) | -0.4  (-0.57 to -0.22) | <0.001 | 1.41  (1.00 to 2.04) | 1.13  (0.79 to 1.64) | -0.7  (-0.94 to -0.47) | <0.001 |
|  | Comoros | 17.32  (6.52 to 37.83) | 11.25  (5.29 to 24.14) | -1.54  (-3.38 to 0.33) | 0.106 | 6.16  (2.48 to 13.21) | 3.92  (1.97 to 8.13) | -1.6  (-3.36 to 0.19) | 0.079 |
|  | Congo | 9.36  (4.93 to 16.36) | 7.13  (3.85 to 12.17) | -0.77  (-0.97 to -0.58) | <0.001 | 3.43  (1.77 to 6.06) | 2.52  (1.46 to 4.25) | -0.91  (-1.1 to -0.73) | <0.001 |
|  | Cook Islands | 2.66  (1.33 to 4.73) | 2.51  (1.23 to 4.58) | -0.19  (-0.29 to -0.09) | <0.001 | 0.57  (0.31 to 1.03) | 0.49  (0.25 to 0.90) | -0.48  (-0.59 to -0.38) | <0.001 |
|  | Costa Rica | 3.67  (2.23 to 5.78) | 3.91  (2.43 to 6.12) | 0.19  (0.04 to 0.35) | 0.012 | 1.10  (0.72 to 1.70) | 1.08  (0.73 to 1.62) | -0.05  (-0.39 to 0.29) | 0.763 |
|  | Coted'Ivoire | 12.65  (7.38 to 20.31) | 9.03  (4.75 to 15.27) | -1.09  (-1.19 to -1) | <0.001 | 3.95  (2.16 to 6.62) | 2.53  (1.27 to 4.54) | -1.48  (-1.69 to -1.26) | <0.001 |
|  | Croatia | 3.07  (1.52 to 5.40) | 3.50  (1.69 to 6.26) | 0.43  (0.31 to 0.55) | <0.001 | 0.85  (0.47 to 1.54) | 0.80  (0.43 to 1.47) | -0.19  (-0.32 to -0.06) | 0.003 |
|  | Cuba | 4.40  (2.72 to 6.87) | 5.96  (3.97 to 8.65) | 1.13  (0.6 to 1.67) | <0.001 | 1.28  (0.88 to 1.88) | 1.71  (1.22 to 2.36) | 1.06  (0.45 to 1.68) | 0.001 |
|  | Cyprus | 0.90  (0.49 to 1.56) | 0.70  (0.37 to 1.22) | -0.84  (-1.18 to -0.5) | <0.001 | 0.29  (0.16 to 0.50) | 0.19  (0.10 to 0.33) | -1.4  (-1.68 to -1.13) | <0.001 |
|  | Czechia | 3.17  (1.73 to 5.39) | 3.04  (1.54 to 5.35) | -0.13  (-0.24 to -0.01) | 0.03 | 0.99  (0.59 to 1.64) | 0.77  (0.43 to 1.34) | -0.78  (-0.93 to -0.63) | <0.001 |
|  | Democratic People's Republic of Korea | 1.58  (0.91 to 2.62) | 1.62  (0.93 to 2.64) | 0.07  (0.04 to 0.1) | <0.001 | 0.38  (0.20 to 0.67) | 0.38  (0.21 to 0.67) | -0.02  (-0.11 to 0.06) | 0.588 |
|  | Democratic Republic of the Congo | 9.38  (4.85 to 16.41) | 6.86  (3.61 to 11.98) | -1.02  (-1.23 to -0.81) | <0.001 | 3.48  (1.83 to 6.08) | 2.49  (1.42 to 4.24) | -1.11  (-1.28 to -0.93) | <0.001 |
|  | Denmark | 1.38  (0.92 to 2.09) | 1.09  (0.65 to 1.81) | -0.71  (-0.96 to -0.46) | <0.001 | 0.43  (0.30 to 0.65) | 0.28  (0.17 to 0.43) | -1.4  (-1.72 to -1.08) | <0.001 |
|  | Djibouti | 9.08  (4.12 to 20.39) | 8.25  (3.82 to 19.77) | -0.26  (-0.54 to 0.02) | 0.068 | 3.25  (1.60 to 7.25) | 2.83  (1.39 to 6.77) | -0.39  (-0.66 to -0.13) | 0.003 |
|  | Dominica | 2.88  (1.37 to 5.25) | 2.95  (1.44 to 5.35) | 0.09  (-0.07 to 0.25) | 0.286 | 0.98  (0.53 to 1.70) | 0.90  (0.50 to 1.57) | -0.27  (-0.35 to -0.19) | <0.001 |
|  | Dominican Republic | 7.64  (4.39 to 12.26) | 7.19  (3.75 to 12.80) | -0.22  (-0.55 to 0.12) | 0.207 | 2.68  (1.62 to 4.16) | 2.33  (1.24 to 4.14) | -0.49  (-0.78 to -0.21) | 0.001 |
|  | Ecuador | 4.66  (2.27 to 8.28) | 5.30  (2.77 to 9.15) | 0.44  (0.3 to 0.58) | <0.001 | 1.39  (0.81 to 2.30) | 1.34  (0.79 to 2.20) | -0.1  (-0.27 to 0.08) | 0.268 |
|  | Egypt | 2.50  (1.47 to 4.05) | 2.22  (1.24 to 3.71) | -0.39  (-0.48 to -0.3) | <0.001 | 0.73  (0.40 to 1.25) | 0.63  (0.33 to 1.11) | -0.5  (-0.62 to -0.38) | <0.001 |
|  | El Salvador | 3.19  (1.76 to 5.19) | 3.03  (1.60 to 5.21) | -0.18  (-0.36 to 0.01) | 0.061 | 0.92  (0.55 to 1.51) | 0.75  (0.44 to 1.21) | -0.67  (-0.85 to -0.48) | <0.001 |
|  | Equatorial Guinea | 10.67  (5.29 to 19.39) | 6.86  (3.58 to 11.91) | -1.41  (-1.68 to -1.14) | <0.001 | 4.01  (2.04 to 7.32) | 2.37  (1.38 to 4.05) | -1.65  (-1.92 to -1.39) | <0.001 |
|  | Eritrea | 13.75  (6.07 to 33.51) | 10.15  (4.69 to 23.49) | -1.03  (-1.22 to -0.85) | <0.001 | 5.04  (2.36 to 11.75) | 3.60  (1.78 to 8.00) | -1.15  (-1.33 to -0.97) | <0.001 |
|  | Estonia | 5.15  (3.37 to 7.69) | 3.97  (2.21 to 6.54) | -0.89  (-1.22 to -0.56) | <0.001 | 2.00  (1.42 to 2.90) | 1.10  (0.70 to 1.73) | -2  (-2.35 to -1.65) | <0.001 |
|  | Eswatini | 10.21  (5.55 to 17.75) | 7.89  (4.06 to 14.27) | -0.85  (-1.05 to -0.66) | <0.001 | 3.83  (2.06 to 6.60) | 2.80  (1.47 to 4.98) | -1.02  (-1.27 to -0.76) | <0.001 |
|  | Ethiopia | 27.86  (12.12 to 52.48) | 10.22  (5.12 to 27.07) | -3.15  (-3.34 to -2.97) | <0.001 | 9.59  (4.19 to 17.89) | 3.25  (1.61 to 8.96) | -3.41  (-3.6 to -3.22) | <0.001 |
|  | Fiji | 4.21  (2.24 to 7.20) | 3.29  (1.73 to 5.74) | -0.79  (-1.17 to -0.41) | <0.001 | 1.05  (0.57 to 1.91) | 0.78  (0.43 to 1.38) | -0.97  (-1.34 to -0.6) | <0.001 |
|  | Finland | 2.36  (1.60 to 3.48) | 2.06  (1.23 to 3.28) | -0.42  (-0.81 to -0.03) | 0.036 | 1.12  (0.72 to 1.70) | 0.77  (0.46 to 1.27) | -1.18  (-1.32 to -1.04) | <0.001 |
|  | France | 1.18  (0.71 to 1.90) | 1.01  (0.57 to 1.68) | -0.52  (-0.58 to -0.46) | <0.001 | 0.36  (0.22 to 0.59) | 0.24  (0.14 to 0.39) | -1.32  (-1.43 to -1.21) | <0.001 |
|  | Gabon | 7.83  (4.06 to 13.75) | 6.01  (3.20 to 10.64) | -0.85  (-1.03 to -0.67) | <0.001 | 2.89  (1.61 to 4.92) | 2.08  (1.19 to 3.58) | -1.07  (-1.27 to -0.87) | <0.001 |
|  | Gambia | 9.63  (5.18 to 15.97) | 9.22  (4.90 to 15.46) | -0.14  (-0.23 to -0.04) | 0.004 | 2.95  (1.51 to 5.17) | 2.56  (1.33 to 4.60) | -0.44  (-0.75 to -0.13) | 0.005 |
|  | Georgia | 4.78  (2.62 to 7.61) | 5.66  (2.89 to 9.71) | 0.53  (0.3 to 0.77) | <0.001 | 1.64  (1.03 to 2.64) | 1.43  (0.84 to 2.39) | -0.52  (-0.82 to -0.22) | 0.001 |
|  | Germany | 1.14  (0.70 to 1.83) | 0.96  (0.53 to 1.62) | -0.57  (-0.65 to -0.48) | <0.001 | 0.36  (0.22 to 0.58) | 0.23  (0.13 to 0.40) | -1.43  (-1.53 to -1.33) | <0.001 |
|  | Ghana | 10.06  (5.61 to 16.86) | 8.72  (4.70 to 14.62) | -0.48  (-0.57 to -0.39) | <0.001 | 3.36  (1.83 to 5.79) | 2.67  (1.32 to 4.69) | -0.76  (-0.89 to -0.64) | <0.001 |
|  | Greece | 1.02  (0.57 to 1.69) | 1.63  (0.92 to 2.68) | 1.57  (1.4 to 1.75) | <0.001 | 0.27  (0.15 to 0.45) | 0.33  (0.18 to 0.56) | 0.63  (0.49 to 0.77) | <0.001 |
|  | Greenland | 2.04  (1.05 to 3.51) | 2.05  (1.06 to 3.59) | 0.01  (-0.03 to 0.05) | 0.733 | 1.27  (0.66 to 2.23) | 1.17  (0.61 to 2.06) | -0.25  (-0.3 to -0.21) | <0.001 |
|  | Grenada | 4.12  (2.45 to 6.47) | 4.98  (3.19 to 7.56) | 0.68  (0.3 to 1.06) | <0.001 | 1.44  (0.95 to 2.22) | 1.57  (1.08 to 2.28) | 0.35  (-0.04 to 0.75) | 0.08 |
|  | Guam | 2.67  (1.32 to 4.68) | 2.52  (1.16 to 4.62) | -0.17  (-0.35 to 0.02) | 0.073 | 0.56  (0.30 to 1.00) | 0.48  (0.25 to 0.88) | -0.51  (-0.75 to -0.28) | <0.001 |
|  | Guatemala | 4.70  (3.17 to 6.94) | 4.09  (2.65 to 6.34) | -0.38  (-1.15 to 0.4) | 0.343 | 1.58  (1.11 to 2.32) | 1.15  (0.79 to 1.70) | -0.99  (-1.22 to -0.76) | <0.001 |
|  | Guinea | 13.36  (7.61 to 21.79) | 9.31  (5.04 to 15.81) | -1.19  (-1.42 to -0.97) | <0.001 | 4.32  (2.23 to 7.29) | 2.67  (1.38 to 4.70) | -1.57  (-1.76 to -1.37) | <0.001 |
|  | Guinea-Bissau | 15.44  (7.92 to 27.57) | 10.50  (5.68 to 18.14) | -1.26  (-1.37 to -1.16) | <0.001 | 5.31  (2.63 to 9.60) | 3.14  (1.65 to 5.54) | -1.71  (-1.88 to -1.55) | <0.001 |
|  | Guyana | 5.13  (3.31 to 7.64) | 7.22  (4.63 to 10.83) | 1.2  (-0.07 to 2.49) | 0.064 | 1.81  (1.24 to 2.64) | 2.35  (1.56 to 3.48) | 0.95  (-0.43 to 2.35) | 0.178 |
|  | Haiti | 9.82  (4.81 to 18.16) | 8.94  (3.86 to 17.02) | -0.3  (-0.55 to -0.06) | 0.017 | 3.54  (1.87 to 6.43) | 3.03  (1.37 to 5.81) | -0.44  (-0.76 to -0.13) | 0.006 |
|  | Honduras | 8.25  (4.49 to 14.75) | 5.14  (2.76 to 9.37) | -1.56  (-1.76 to -1.36) | <0.001 | 2.74  (1.50 to 4.95) | 1.58  (0.87 to 2.90) | -1.75  (-1.93 to -1.56) | <0.001 |
|  | Hungary | 4.31  (2.75 to 6.65) | 3.49  (1.94 to 5.77) | -0.66  (-0.88 to -0.44) | <0.001 | 1.49  (1.04 to 2.21) | 0.97  (0.60 to 1.60) | -1.41  (-1.65 to -1.16) | <0.001 |
|  | Iceland | 0.73  (0.43 to 1.19) | 0.63  (0.34 to 1.10) | -0.49  (-0.68 to -0.29) | <0.001 | 0.24  (0.15 to 0.41) | 0.16  (0.09 to 0.29) | -1.26  (-1.45 to -1.06) | <0.001 |
|  | India | 9.90  (6.29 to 13.40) | 6.46  (4.23 to 9.23) | -1.38  (-1.68 to -1.08) | <0.001 | 2.79  (1.63 to 3.78) | 1.60  (1.03 to 2.24) | -1.77  (-2.21 to -1.32) | <0.001 |
|  | Indonesia | 1.28  (0.79 to 1.91) | 0.96  (0.58 to 1.51) | -0.94  (-1 to -0.88) | <0.001 | 0.29  (0.18 to 0.44) | 0.18  (0.11 to 0.29) | -1.51  (-1.56 to -1.45) | <0.001 |
|  | Iran (Islamic Republic of) | 2.21  (1.26 to 3.58) | 2.25  (1.26 to 3.70) | 0.1  (-0.13 to 0.33) | 0.393 | 0.49  (0.27 to 0.86) | 0.52  (0.29 to 0.90) | 0.27  (0.17 to 0.37) | <0.001 |
|  | Iraq | 2.46  (1.36 to 4.19) | 2.14  (1.22 to 3.53) | -0.46  (-0.51 to -0.41) | <0.001 | 0.70  (0.39 to 1.20) | 0.55  (0.30 to 0.98) | -0.78  (-0.91 to -0.65) | <0.001 |
|  | Ireland | 1.16  (0.71 to 1.85) | 0.98  (0.55 to 1.67) | -0.52  (-0.6 to -0.44) | <0.001 | 0.34  (0.22 to 0.55) | 0.22  (0.13 to 0.37) | -1.44  (-1.61 to -1.27) | <0.001 |
|  | Israel | 1.04  (0.60 to 1.73) | 0.93  (0.50 to 1.59) | -0.38  (-0.42 to -0.33) | <0.001 | 0.30  (0.17 to 0.51) | 0.21  (0.11 to 0.37) | -1.21  (-1.34 to -1.08) | <0.001 |
|  | Italy | 2.60  (1.38 to 4.37) | 4.42  (2.51 to 7.23) | 1.7  (1.46 to 1.94) | <0.001 | 0.71  (0.40 to 1.21) | 0.80  (0.46 to 1.33) | 0.38  (0.15 to 0.6) | 0.001 |
|  | Jamaica | 3.83  (2.27 to 6.33) | 4.49  (2.77 to 7.10) | 0.52  (-0.23 to 1.28) | 0.176 | 1.21  (0.79 to 1.87) | 1.35  (0.91 to 1.99) | 0.39  (-0.5 to 1.29) | 0.393 |
|  | Japan | 4.65  (2.45 to 7.97) | 5.54  (2.93 to 9.37) | 0.57  (0.54 to 0.59) | <0.001 | 1.61  (0.85 to 2.82) | 1.31  (0.72 to 2.26) | -0.67  (-0.72 to -0.63) | <0.001 |
|  | Jordan | 1.47  (0.81 to 2.48) | 1.32  (0.74 to 2.25) | -0.36  (-0.42 to -0.3) | <0.001 | 0.41  (0.20 to 0.76) | 0.35  (0.18 to 0.63) | -0.56  (-0.65 to -0.47) | <0.001 |
|  | Kazakhstan | 5.89  (3.80 to 9.05) | 4.45  (2.39 to 7.52) | -0.88  (-1.35 to -0.4) | <0.001 | 2.17  (1.52 to 3.17) | 1.24  (0.76 to 2.04) | -1.81  (-2.36 to -1.26) | <0.001 |
|  | Kenya | 9.75  (5.85 to 15.19) | 7.88  (4.66 to 13.75) | -0.68  (-0.88 to -0.48) | <0.001 | 3.18  (1.91 to 5.02) | 2.45  (1.43 to 4.37) | -0.83  (-1.01 to -0.64) | <0.001 |
|  | Kiribati | 3.07  (1.56 to 5.27) | 2.89  (1.47 to 5.09) | -0.2  (-0.25 to -0.14) | <0.001 | 0.67  (0.38 to 1.11) | 0.61  (0.34 to 1.02) | -0.31  (-0.38 to -0.24) | <0.001 |
|  | Kuwait | 2.00  (1.02 to 3.46) | 2.20  (1.24 to 3.74) | 0.31  (0.2 to 0.42) | <0.001 | 0.53  (0.27 to 1.00) | 0.53  (0.29 to 0.92) | -0.02  (-0.24 to 0.21) | 0.889 |
|  | Kyrgyzstan | 6.27  (4.13 to 9.29) | 4.30  (2.28 to 7.46) | -1.26  (-1.72 to -0.79) | <0.001 | 2.29  (1.64 to 3.25) | 1.22  (0.72 to 2.01) | -2.08  (-2.61 to -1.55) | <0.001 |
|  | Lao People's Democratic Republic | 1.90  (1.06 to 3.35) | 1.86  (1.03 to 3.29) | -0.08  (-0.17 to 0.01) | 0.068 | 0.45  (0.23 to 0.87) | 0.43  (0.22 to 0.83) | -0.11  (-0.25 to 0.03) | 0.11 |
|  | Latvia | 5.93  (3.97 to 8.63) | 4.33  (2.55 to 7.09) | -0.99  (-1.27 to -0.72) | <0.001 | 2.23  (1.62 to 3.13) | 1.27  (0.83 to 1.98) | -1.83  (-2.1 to -1.55) | <0.001 |
|  | Lebanon | 2.36  (1.40 to 3.76) | 2.06  (1.19 to 3.38) | -0.43  (-0.48 to -0.38) | <0.001 | 0.66  (0.38 to 1.12) | 0.56  (0.30 to 0.96) | -0.52  (-0.59 to -0.45) | <0.001 |
|  | Lesotho | 7.85  (4.07 to 13.93) | 8.44  (4.40 to 14.93) | 0.18  (-0.03 to 0.38) | 0.09 | 3.02  (1.56 to 5.29) | 3.04  (1.65 to 5.27) | -0.02  (-0.18 to 0.14) | 0.815 |
|  | Liberia | 13.17  (7.27 to 21.74) | 10.06  (5.09 to 17.85) | -0.9  (-1.04 to -0.76) | <0.001 | 4.32  (2.31 to 7.41) | 2.90  (1.38 to 5.43) | -1.27  (-1.44 to -1.1) | <0.001 |
|  | Libya | 2.01  (1.10 to 3.38) | 2.00  (1.11 to 3.33) | 0  (-0.05 to 0.04) | 0.851 | 0.54  (0.28 to 0.98) | 0.55  (0.28 to 0.97) | 0.08  (0.02 to 0.14) | 0.01 |
|  | Lithuania | 4.01  (2.36 to 6.46) | 3.53  (1.88 to 6.13) | -0.41  (-0.72 to -0.09) | 0.011 | 1.41  (0.92 to 2.22) | 1.00  (0.60 to 1.65) | -1.2  (-1.6 to -0.79) | <0.001 |
|  | Luxembourg | 1.55  (0.95 to 2.45) | 1.39  (0.77 to 2.35) | -0.34  (-0.51 to -0.17) | <0.001 | 0.51  (0.31 to 0.85) | 0.35  (0.19 to 0.60) | -1.25  (-1.39 to -1.11) | <0.001 |
|  | Madagascar | 19.43  (8.20 to 38.84) | 14.93  (7.03 to 28.30) | -0.89  (-1.05 to -0.74) | <0.001 | 6.94  (3.06 to 13.63) | 5.24  (2.57 to 9.83) | -0.95  (-1.11 to -0.79) | <0.001 |
|  | Malawi | 14.73  (7.07 to 28.02) | 10.51  (5.02 to 20.48) | -1.05  (-1.3 to -0.8) | <0.001 | 5.36  (2.62 to 10.13) | 3.76  (1.89 to 7.19) | -1.14  (-1.4 to -0.89) | <0.001 |
|  | Malaysia | 1.90  (1.12 to 3.07) | 1.50  (0.88 to 2.45) | -0.77  (-0.89 to -0.65) | <0.001 | 0.42  (0.23 to 0.72) | 0.30  (0.17 to 0.51) | -1.05  (-1.23 to -0.86) | <0.001 |
|  | Maldives | 4.75  (2.31 to 8.93) | 2.05  (1.20 to 3.42) | -2.69  (-2.9 to -2.47) | <0.001 | 1.44  (0.63 to 2.82) | 0.50  (0.27 to 0.87) | -3.43  (-3.74 to -3.12) | <0.001 |
|  | Mali | 20.14  (11.53 to 32.47) | 9.89  (5.25 to 17.12) | -2.3  (-2.4 to -2.2) | <0.001 | 6.77  (3.53 to 11.42) | 2.92  (1.49 to 5.11) | -2.73  (-2.9 to -2.56) | <0.001 |
|  | Malta | 0.79  (0.45 to 1.31) | 0.70  (0.38 to 1.23) | -0.37  (-0.88 to 0.13) | 0.147 | 0.25  (0.15 to 0.41) | 0.18  (0.09 to 0.32) | -1.04  (-1.4 to -0.69) | <0.001 |
|  | Marshall Islands | 2.73  (1.38 to 4.80) | 2.65  (1.32 to 4.67) | -0.1  (-0.17 to -0.03) | 0.003 | 0.70  (0.37 to 1.23) | 0.66  (0.36 to 1.14) | -0.19  (-0.31 to -0.08) | 0.001 |
|  | Mauritania | 12.42  (6.86 to 20.52) | 8.79  (4.65 to 14.92) | -1.15  (-1.27 to -1.02) | <0.001 | 4.07  (2.08 to 7.22) | 2.42  (1.23 to 4.38) | -1.72  (-1.88 to -1.55) | <0.001 |
|  | Mauritius | 1.24  (0.69 to 2.11) | 1.72  (1.13 to 2.64) | 1.05  (0.75 to 1.36) | <0.001 | 0.22  (0.12 to 0.39) | 0.35  (0.24 to 0.52) | 1.53  (0.97 to 2.11) | <0.001 |
|  | Mexico | 4.59  (3.32 to 6.42) | 5.09  (3.58 to 7.29) | 0.37  (0.13 to 0.6) | 0.002 | 1.32  (0.99 to 1.83) | 1.21  (0.90 to 1.70) | -0.25  (-0.41 to -0.08) | 0.004 |
|  | Micronesia (Federated States of) | 3.00  (1.52 to 5.17) | 2.80  (1.44 to 4.88) | -0.25  (-0.32 to -0.19) | <0.001 | 0.77  (0.42 to 1.36) | 0.67  (0.36 to 1.17) | -0.46  (-0.53 to -0.39) | <0.001 |
|  | Monaco | 0.97  (0.54 to 1.67) | 0.92  (0.50 to 1.58) | -0.17  (-0.21 to -0.13) | <0.001 | 0.27  (0.15 to 0.47) | 0.22  (0.12 to 0.38) | -0.73  (-0.8 to -0.67) | <0.001 |
|  | Mongolia | 9.68  (5.01 to 18.34) | 5.99  (3.52 to 9.52) | -1.63  (-1.94 to -1.32) | <0.001 | 3.67  (1.83 to 7.24) | 1.98  (1.18 to 3.18) | -1.99  (-2.32 to -1.66) | <0.001 |
|  | Montenegro | 2.95  (1.49 to 5.26) | 2.93  (1.45 to 5.34) | -0.03  (-0.07 to 0.02) | 0.324 | 0.90  (0.50 to 1.62) | 0.81  (0.44 to 1.45) | -0.29  (-0.4 to -0.17) | <0.001 |
|  | Morocco | 2.10  (1.14 to 3.58) | 2.06  (1.10 to 3.55) | -0.06  (-0.08 to -0.03) | <0.001 | 0.61  (0.30 to 1.09) | 0.58  (0.30 to 1.05) | -0.14  (-0.21 to -0.06) | <0.001 |
|  | Mozambique | 26.02  (10.63 to 53.20) | 14.18  (6.48 to 29.32) | -2.02  (-2.24 to -1.8) | <0.001 | 9.42  (4.02 to 19.04) | 5.02  (2.40 to 10.11) | -2.1  (-2.31 to -1.88) | <0.001 |
|  | Myanmar | 2.54  (1.38 to 4.92) | 2.06  (1.16 to 3.42) | -0.7  (-0.85 to -0.55) | <0.001 | 0.63  (0.32 to 1.38) | 0.51  (0.27 to 0.89) | -0.73  (-0.87 to -0.59) | <0.001 |
|  | Namibia | 8.65  (4.47 to 15.15) | 6.72  (3.47 to 12.15) | -0.79  (-1.12 to -0.47) | <0.001 | 3.30  (1.73 to 5.84) | 2.32  (1.30 to 3.99) | -1.14  (-1.47 to -0.82) | <0.001 |
|  | Nauru | 2.91  (1.46 to 5.13) | 2.74  (1.40 to 4.88) | -0.18  (-0.23 to -0.12) | <0.001 | 0.67  (0.37 to 1.20) | 0.60  (0.33 to 1.06) | -0.36  (-0.45 to -0.28) | <0.001 |
|  | Nepal | 9.07  (4.84 to 16.52) | 6.69  (3.63 to 11.22) | -0.99  (-1.11 to -0.87) | <0.001 | 2.85  (1.57 to 5.23) | 1.82  (1.07 to 3.05) | -1.44  (-1.52 to -1.36) | <0.001 |
|  | Netherlands | 1.16  (0.70 to 1.86) | 0.99  (0.55 to 1.71) | -0.5  (-0.59 to -0.42) | <0.001 | 0.36  (0.23 to 0.58) | 0.24  (0.14 to 0.41) | -1.28  (-1.39 to -1.18) | <0.001 |
|  | New Zealand | 6.58  (3.12 to 12.23) | 8.50  (4.19 to 15.00) | 0.81  (0.7 to 0.93) | <0.001 | 2.21  (0.99 to 4.26) | 2.08  (0.96 to 3.88) | -0.18  (-0.42 to 0.06) | 0.141 |
|  | Nicaragua | 3.57  (2.03 to 5.67) | 3.16  (1.74 to 5.22) | -0.44  (-0.58 to -0.29) | <0.001 | 1.07  (0.65 to 1.72) | 0.81  (0.49 to 1.31) | -0.92  (-1.15 to -0.69) | <0.001 |
|  | Niger | 12.35  (7.01 to 20.56) | 8.41  (4.27 to 14.94) | -1.27  (-1.38 to -1.15) | <0.001 | 3.89  (2.09 to 6.72) | 2.29  (1.09 to 4.15) | -1.75  (-1.93 to -1.57) | <0.001 |
|  | Nigeria | 10.91  (6.39 to 17.68) | 7.49  (4.48 to 12.04) | -1.24  (-1.3 to -1.18) | <0.001 | 2.51  (1.46 to 4.09) | 1.52  (0.91 to 2.46) | -1.65  (-1.73 to -1.56) | <0.001 |
|  | Niue | 2.69  (1.32 to 4.71) | 2.63  (1.33 to 4.70) | -0.09  (-0.16 to -0.02) | 0.008 | 0.58  (0.31 to 1.06) | 0.53  (0.28 to 0.92) | -0.31  (-0.36 to -0.26) | <0.001 |
|  | North Macedonia | 3.11  (1.57 to 5.36) | 2.96  (1.47 to 5.37) | -0.16  (-0.24 to -0.07) | 0.001 | 0.89  (0.49 to 1.57) | 0.76  (0.40 to 1.39) | -0.5  (-0.56 to -0.44) | <0.001 |
|  | Northern Mariana Islands | 3.09  (1.58 to 5.31) | 2.52  (1.20 to 4.61) | -0.66  (-0.82 to -0.5) | <0.001 | 0.74  (0.41 to 1.33) | 0.51  (0.26 to 0.94) | -1.19  (-1.4 to -0.98) | <0.001 |
|  | Norway | 3.33  (1.81 to 5.56) | 3.26  (1.79 to 5.44) | -0.06  (-0.19 to 0.07) | 0.358 | 0.70  (0.39 to 1.23) | 0.76  (0.43 to 1.26) | 0.26  (0.17 to 0.36) | <0.001 |
|  | Oman | 2.13  (1.16 to 3.61) | 2.02  (1.07 to 3.42) | -0.18  (-0.22 to -0.14) | <0.001 | 0.59  (0.32 to 1.04) | 0.54  (0.28 to 0.98) | -0.32  (-0.38 to -0.25) | <0.001 |
|  | Pakistan | 5.37  (3.10 to 8.87) | 5.58  (3.30 to 8.89) | 0.1  (-0.17 to 0.37) | 0.486 | 1.30  (0.76 to 2.21) | 1.23  (0.74 to 1.99) | -0.19  (-0.51 to 0.13) | 0.25 |
|  | Palau | 2.66  (1.29 to 4.64) | 2.56  (1.24 to 4.61) | -0.11  (-0.24 to 0.01) | 0.082 | 0.58  (0.31 to 1.02) | 0.51  (0.27 to 0.91) | -0.36  (-0.47 to -0.25) | <0.001 |
|  | Palestine | 2.21  (1.28 to 3.58) | 2.17  (1.15 to 3.70) | -0.05  (-0.11 to 0) | 0.074 | 0.65  (0.35 to 1.15) | 0.62  (0.35 to 1.06) | -0.13  (-0.19 to -0.06) | <0.001 |
|  | Panama | 3.97  (2.50 to 6.17) | 3.72  (2.29 to 5.90) | -0.21  (-0.48 to 0.07) | 0.149 | 1.11  (0.75 to 1.64) | 0.98  (0.66 to 1.46) | -0.41  (-0.74 to -0.09) | 0.012 |
|  | Papua New Guinea | 5.36  (2.92 to 8.84) | 2.95  (1.47 to 5.22) | -1.81  (-2 to -1.62) | <0.001 | 1.63  (0.92 to 2.77) | 1.04  (0.52 to 1.92) | -1.3  (-1.52 to -1.07) | <0.001 |
|  | Paraguay | 4.66  (3.21 to 6.68) | 4.09  (2.70 to 6.10) | -0.38  (-0.53 to -0.22) | <0.001 | 1.48  (1.01 to 2.16) | 1.25  (0.81 to 1.86) | -0.45  (-0.61 to -0.28) | <0.001 |
|  | Peru | 5.13  (2.68 to 8.71) | 5.14  (2.53 to 9.10) | 0.07  (-0.12 to 0.26) | 0.466 | 1.62  (0.96 to 2.61) | 1.40  (0.79 to 2.39) | -0.46  (-0.63 to -0.28) | <0.001 |
|  | Philippines | 1.75  (1.05 to 2.76) | 2.74  (1.65 to 4.17) | 1.49  (1.34 to 1.64) | <0.001 | 0.29  (0.18 to 0.46) | 0.55  (0.31 to 0.81) | 2.1  (1.84 to 2.36) | <0.001 |
|  | Poland | 3.36  (2.01 to 5.28) | 3.40  (1.92 to 5.57) | 0.05  (-0.09 to 0.18) | 0.513 | 0.96  (0.60 to 1.57) | 0.61  (0.35 to 1.07) | -1.47  (-1.6 to -1.35) | <0.001 |
|  | Portugal | 0.75  (0.48 to 1.18) | 0.82  (0.46 to 1.37) | 0.32  (-0.01 to 0.66) | 0.061 | 0.27  (0.18 to 0.42) | 0.21  (0.12 to 0.37) | -0.67  (-1.23 to -0.1) | 0.022 |
|  | Puerto Rico | 3.54  (1.93 to 5.92) | 3.65  (2.09 to 6.02) | 0.11  (-0.23 to 0.46) | 0.514 | 1.09  (0.68 to 1.78) | 1.05  (0.67 to 1.63) | -0.12  (-0.8 to 0.56) | 0.728 |
|  | Qatar | 2.56  (1.54 to 4.08) | 2.18  (1.20 to 3.65) | -0.44  (-0.8 to -0.08) | 0.016 | 0.73  (0.41 to 1.24) | 0.59  (0.32 to 1.04) | -0.69  (-1.07 to -0.31) | <0.001 |
|  | Republic of Korea | 4.97  (2.11 to 9.22) | 5.07  (2.05 to 9.75) | 0.07  (0.01 to 0.13) | 0.016 | 2.07  (1.11 to 3.66) | 1.55  (0.80 to 2.76) | -0.93  (-1.01 to -0.85) | <0.001 |
|  | Republic of Moldova | 5.92  (4.01 to 8.63) | 4.26  (2.59 to 6.84) | -1.26  (-1.46 to -1.06) | <0.001 | 2.14  (1.52 to 3.06) | 1.31  (0.87 to 1.99) | -1.72  (-1.99 to -1.44) | <0.001 |
|  | Romania | 3.17  (1.69 to 5.45) | 2.97  (1.52 to 5.28) | -0.17  (-0.26 to -0.07) | 0.001 | 0.96  (0.56 to 1.66) | 0.74  (0.41 to 1.31) | -0.82  (-0.9 to -0.73) | <0.001 |
|  | Russian Federation | 5.23  (3.80 to 7.26) | 4.54  (3.24 to 6.46) | -0.43  (-1.04 to 0.19) | 0.173 | 1.70  (1.29 to 2.40) | 1.33  (0.98 to 1.87) | -0.69  (-1.41 to 0.04) | 0.064 |
|  | Rwanda | 21.58  (9.74 to 45.99) | 8.82  (4.04 to 18.70) | -2.84  (-3.17 to -2.51) | <0.001 | 7.69  (3.52 to 16.15) | 3.04  (1.53 to 6.32) | -2.96  (-3.29 to -2.63) | <0.001 |
|  | Saint Kitts and Nevis | 6.07  (4.17 to 8.65) | 4.97  (3.11 to 7.63) | -0.59  (-1.5 to 0.33) | 0.205 | 2.02  (1.45 to 2.79) | 1.47  (1.01 to 2.12) | -0.98  (-1.94 to 0) | 0.049 |
|  | Saint Lucia | 5.26  (3.52 to 7.87) | 6.17  (4.13 to 8.99) | 0.53  (-0.38 to 1.44) | 0.258 | 1.84  (1.30 to 2.62) | 1.98  (1.42 to 2.78) | 0.33  (-0.68 to 1.35) | 0.519 |
|  | Saint Vincent and the Grenadines | 3.40  (1.86 to 5.69) | 3.67  (2.12 to 5.93) | 0.2  (-0.15 to 0.56) | 0.263 | 1.25  (0.77 to 2.06) | 1.16  (0.74 to 1.82) | -0.31  (-0.7 to 0.08) | 0.119 |
|  | Samoa | 2.78  (1.32 to 4.83) | 2.70  (1.31 to 4.92) | -0.12  (-0.22 to -0.02) | 0.015 | 0.65  (0.34 to 1.20) | 0.60  (0.31 to 1.07) | -0.25  (-0.29 to -0.21) | <0.001 |
|  | San Marino | 0.96  (0.52 to 1.66) | 0.92  (0.49 to 1.61) | -0.14  (-0.2 to -0.08) | <0.001 | 0.27  (0.14 to 0.47) | 0.22  (0.12 to 0.38) | -0.71  (-0.77 to -0.66) | <0.001 |
|  | Sao Tome and Principe | 8.87  (4.51 to 15.66) | 8.46  (4.38 to 14.77) | -0.16  (-0.31 to -0.01) | 0.034 | 2.66  (1.34 to 4.90) | 2.28  (1.11 to 4.23) | -0.47  (-0.73 to -0.21) | <0.001 |
|  | Saudi Arabia | 2.31  (1.23 to 3.91) | 2.16  (1.13 to 3.71) | -0.22  (-0.25 to -0.19) | <0.001 | 0.54  (0.30 to 0.94) | 0.50  (0.27 to 0.89) | -0.25  (-0.3 to -0.19) | <0.001 |
|  | Senegal | 13.81  (7.59 to 22.51) | 8.53  (4.69 to 14.71) | -1.58  (-1.79 to -1.37) | <0.001 | 4.44  (2.21 to 7.72) | 2.31  (1.14 to 4.22) | -2.14  (-2.34 to -1.94) | <0.001 |
|  | Serbia | 2.97  (1.56 to 5.10) | 2.87  (1.42 to 5.13) | -0.13  (-0.35 to 0.1) | 0.273 | 0.90  (0.51 to 1.53) | 0.78  (0.41 to 1.40) | -0.46  (-0.58 to -0.33) | <0.001 |
|  | Seychelles | 4.83  (2.82 to 7.37) | 2.33  (1.50 to 3.54) | -2.24  (-2.58 to -1.89) | <0.001 | 1.46  (0.80 to 2.28) | 0.63  (0.41 to 0.99) | -2.6  (-3 to -2.2) | <0.001 |
|  | Sierra Leone | 10.19  (5.51 to 16.87) | 8.80  (4.63 to 14.94) | -0.51  (-0.68 to -0.35) | <0.001 | 3.10  (1.63 to 5.38) | 2.43  (1.23 to 4.40) | -0.8  (-0.95 to -0.64) | <0.001 |
|  | Singapore | 5.01  (2.15 to 9.57) | 5.10  (2.13 to 9.69) | 0.04  (0.01 to 0.07) | 0.023 | 1.94  (1.01 to 3.33) | 1.38  (0.70 to 2.44) | -1.1  (-1.16 to -1.04) | <0.001 |
|  | Slovakia | 3.08  (1.78 to 5.00) | 2.86  (1.43 to 5.16) | -0.22  (-0.51 to 0.07) | 0.14 | 0.95  (0.58 to 1.57) | 0.82  (0.45 to 1.45) | -0.49  (-0.68 to -0.31) | <0.001 |
|  | Slovenia | 3.02  (1.57 to 5.28) | 2.86  (1.41 to 5.19) | -0.19  (-0.26 to -0.12) | <0.001 | 0.89  (0.51 to 1.52) | 0.64  (0.34 to 1.17) | -1.1  (-1.19 to -1.01) | <0.001 |
|  | Solomon Islands | 3.15  (1.61 to 5.39) | 2.84  (1.43 to 4.94) | -0.33  (-0.42 to -0.24) | <0.001 | 0.77  (0.44 to 1.35) | 0.66  (0.37 to 1.13) | -0.48  (-0.59 to -0.36) | <0.001 |
|  | Somalia | 17.77  (7.29 to 44.13) | 15.00  (6.30 to 35.43) | -0.57  (-0.71 to -0.42) | <0.001 | 6.39  (2.78 to 15.48) | 5.33  (2.39 to 12.51) | -0.58  (-0.68 to -0.48) | <0.001 |
|  | South Africa | 16.73  (10.42 to 23.07) | 6.87  (4.66 to 10.11) | -2.84  (-3.34 to -2.33) | <0.001 | 6.12  (3.80 to 8.52) | 2.37  (1.58 to 3.56) | -3.01  (-3.56 to -2.47) | <0.001 |
|  | South Sudan | 15.42  (7.22 to 34.48) | 15.65  (6.58 to 37.42) | 0.02  (-0.6 to 0.64) | 0.954 | 5.77  (2.72 to 12.26) | 5.64  (2.54 to 13.34) | -0.11  (-0.67 to 0.45) | 0.697 |
|  | Spain | 0.94  (0.59 to 1.48) | 1.53  (0.86 to 2.54) | 1.58  (1.28 to 1.88) | <0.001 | 0.34  (0.21 to 0.54) | 0.38  (0.22 to 0.64) | 0.46  (0.18 to 0.73) | 0.001 |
|  | Sri Lanka | 2.42  (1.57 to 3.60) | 1.73  (1.08 to 2.71) | -1.08  (-1.26 to -0.9) | <0.001 | 0.63  (0.40 to 0.97) | 0.41  (0.25 to 0.66) | -1.39  (-1.6 to -1.18) | <0.001 |
|  | Sudan | 2.06  (1.13 to 3.53) | 2.09  (1.17 to 3.49) | 0.04  (-0.02 to 0.1) | 0.198 | 0.63  (0.33 to 1.12) | 0.62  (0.33 to 1.08) | -0.06  (-0.11 to -0.01) | 0.012 |
|  | Suriname | 3.93  (2.23 to 6.45) | 4.01  (2.18 to 6.82) | 0.1  (-0.26 to 0.47) | 0.588 | 1.33  (0.82 to 2.11) | 1.19  (0.70 to 1.96) | -0.32  (-0.72 to 0.08) | 0.116 |
|  | Sweden | 0.77  (0.46 to 1.29) | 0.87  (0.49 to 1.40) | 0.4  (0.1 to 0.7) | 0.008 | 0.36  (0.22 to 0.59) | 0.47  (0.27 to 0.79) | 0.82  (0.64 to 1.01) | <0.001 |
|  | Switzerland | 1.64  (1.02 to 2.63) | 1.48  (0.86 to 2.47) | -0.35  (-0.56 to -0.13) | 0.001 | 0.48  (0.30 to 0.78) | 0.35  (0.20 to 0.58) | -1.07  (-1.22 to -0.92) | <0.001 |
|  | Syrian Arab Republic | 4.28  (2.69 to 6.53) | 2.97  (1.73 to 5.17) | -1.21  (-1.5 to -0.91) | <0.001 | 1.34  (0.85 to 2.03) | 0.86  (0.50 to 1.59) | -1.46  (-1.83 to -1.09) | <0.001 |
|  | Taiwan (Province of China) | 1.65  (0.97 to 2.68) | 1.58  (0.91 to 2.63) | -0.15  (-0.34 to 0.03) | 0.101 | 0.37  (0.21 to 0.65) | 0.33  (0.18 to 0.60) | -0.4  (-0.68 to -0.12) | 0.006 |
|  | Tajikistan | 7.26  (4.56 to 11.27) | 5.04  (2.68 to 8.74) | -1.19  (-1.3 to -1.08) | <0.001 | 2.96  (1.91 to 4.50) | 1.89  (1.07 to 3.27) | -1.51  (-1.61 to -1.41) | <0.001 |
|  | Thailand | 2.02  (1.17 to 3.32) | 1.66  (0.99 to 2.67) | -0.55  (-0.83 to -0.26) | <0.001 | 0.43  (0.23 to 0.78) | 0.35  (0.20 to 0.57) | -0.68  (-1.14 to -0.21) | 0.004 |
|  | Timor-Leste | 1.61  (0.91 to 2.77) | 1.83  (1.04 to 3.10) | 0.35  (0.1 to 0.6) | 0.006 | 0.36  (0.19 to 0.69) | 0.42  (0.22 to 0.76) | 0.49  (0.08 to 0.9) | 0.018 |
|  | Togo | 12.17  (7.02 to 19.92) | 8.82  (4.79 to 15.21) | -1.06  (-1.22 to -0.89) | <0.001 | 3.80  (2.01 to 6.41) | 2.40  (1.21 to 4.40) | -1.49  (-1.72 to -1.25) | <0.001 |
|  | Tokelau | 2.78  (1.39 to 4.95) | 2.67  (1.34 to 4.73) | -0.13  (-0.2 to -0.07) | <0.001 | 0.65  (0.35 to 1.16) | 0.57  (0.30 to 1.02) | -0.4  (-0.45 to -0.35) | <0.001 |
|  | Tonga | 2.77  (1.40 to 5.04) | 2.62  (1.26 to 4.67) | -0.13  (-0.17 to -0.1) | <0.001 | 0.64  (0.34 to 1.15) | 0.57  (0.30 to 1.01) | -0.35  (-0.39 to -0.32) | <0.001 |
|  | Trinidad and Tobago | 3.90  (2.28 to 6.41) | 4.08  (2.45 to 6.59) | 0.14  (-0.57 to 0.85) | 0.71 | 1.14  (0.74 to 1.80) | 1.12  (0.74 to 1.70) | -0.04  (-0.53 to 0.46) | 0.878 |
|  | Tunisia | 2.04  (1.13 to 3.41) | 2.01  (1.11 to 3.41) | -0.05  (-0.09 to -0.02) | 0.001 | 0.60  (0.30 to 1.11) | 0.55  (0.28 to 1.00) | -0.27  (-0.37 to -0.17) | <0.001 |
|  | Turkmenistan | 6.63  (4.47 to 9.75) | 5.05  (2.98 to 8.26) | -0.89  (-1.57 to -0.2) | 0.011 | 2.59  (1.85 to 3.74) | 1.38  (0.91 to 2.12) | -2.04  (-2.68 to -1.4) | <0.001 |
|  | Tuvalu | 2.85  (1.45 to 4.86) | 2.64  (1.35 to 4.73) | -0.23  (-0.31 to -0.15) | <0.001 | 0.67  (0.37 to 1.21) | 0.57  (0.30 to 1.01) | -0.54  (-0.6 to -0.49) | <0.001 |
|  | Türkiye | 2.63  (1.29 to 4.75) | 2.59  (1.26 to 4.69) | -0.06 (-0.22 to 0.11) | 0.508 | 0.83  (0.43 to 1.51) | 0.73  (0.37 to 1.33) | -0.39 (-0.54 to -0.23) | <0.001 |
|  | Uganda | 10.10  (4.95 to 19.80) | 11.50  (5.52 to 21.63) | 0.39  (0 to 0.78) | 0.051 | 3.64  (1.91 to 7.07) | 4.01  (1.99 to 7.58) | 0.35  (0.01 to 0.68) | 0.043 |
|  | Ukraine | 3.76  (2.10 to 6.17) | 3.94  (2.26 to 6.33) | 0.16  (-0.12 to 0.44) | 0.276 | 1.34  (0.84 to 2.16) | 1.11  (0.69 to 1.70) | -0.65  (-1.06 to -0.24) | 0.002 |
|  | United Arab Emirates | 3.09  (1.74 to 5.66) | 2.46  (1.41 to 4.15) | -0.73  (-1.04 to -0.43) | <0.001 | 0.91  (0.48 to 1.75) | 0.71  (0.40 to 1.21) | -0.79  (-1.04 to -0.54) | <0.001 |
|  | United Kingdom | 3.36  (2.02 to 5.29) | 5.53  (3.30 to 8.85) | 1.58  (1.41 to 1.74) | <0.001 | 0.85  (0.53 to 1.36) | 1.09  (0.66 to 1.77) | 0.79  (0.61 to 0.97) | <0.001 |
|  | United Republic of Tanzania | 16.61  (7.33 to 32.92) | 9.68  (4.77 to 19.37) | -1.75  (-1.99 to -1.51) | <0.001 | 5.90  (2.76 to 11.58) | 3.30  (1.72 to 6.43) | -1.88  (-2.11 to -1.65) | <0.001 |
|  | United States of America | 2.79  (1.66 to 4.43) | 2.03  (1.25 to 3.12) | -1.06  (-1.19 to -0.94) | <0.001 | 1.60  (0.94 to 2.66) | 0.98  (0.61 to 1.57) | -1.62  (-1.76 to -1.47) | <0.001 |
|  | United States Virgin Islands | 4.10  (2.22 to 6.99) | 3.83  (2.00 to 6.74) | -0.04  (-0.39 to 0.31) | 0.829 | 1.45  (0.86 to 2.33) | 1.23  (0.70 to 2.07) | -0.35  (-0.73 to 0.04) | 0.08 |
|  | Uruguay | 4.76  (2.76 to 7.84) | 4.79  (2.71 to 8.02) | -0.02  (-0.21 to 0.18) | 0.863 | 2.55  (1.51 to 4.34) | 2.08  (1.32 to 3.31) | -0.69  (-0.84 to -0.53) | <0.001 |
|  | Uzbekistan | 5.61  (3.55 to 8.65) | 4.20  (2.23 to 7.32) | -0.95  (-1.32 to -0.59) | <0.001 | 2.18  (1.48 to 3.29) | 1.15  (0.67 to 1.94) | -2.09  (-2.51 to -1.66) | <0.001 |
|  | Vanuatu | 2.96  (1.45 to 5.19) | 2.76  (1.37 to 4.81) | -0.23  (-0.29 to -0.18) | <0.001 | 0.74  (0.39 to 1.33) | 0.68  (0.36 to 1.20) | -0.26  (-0.32 to -0.2) | <0.001 |
|  | Venezuela (Bolivarian Republic of) | 3.94  (2.51 to 6.04) | 4.19  (2.67 to 6.39) | 0.19  (-0.1 to 0.48) | 0.189 | 1.17  (0.78 to 1.78) | 1.16  (0.79 to 1.69) | -0.02  (-0.29 to 0.25) | 0.883 |
|  | Viet Nam | 1.23  (0.70 to 2.03) | 1.45  (0.82 to 2.42) | 0.5  (0.45 to 0.54) | <0.001 | 0.22  (0.12 to 0.39) | 0.22  (0.12 to 0.37) | -0.07  (-0.15 to 0.02) | 0.125 |
|  | Yemen | 1.96  (1.10 to 3.26) | 2.00  (1.13 to 3.32) | 0.06  (-0.02 to 0.14) | 0.141 | 0.58  (0.30 to 1.05) | 0.60  (0.32 to 1.08) | 0.11  (-0.09 to 0.31) | 0.261 |
|  | Zambia | 21.02  (9.01 to 40.73) | 13.55  (5.97 to 31.80) | -1.38  (-1.53 to -1.23) | <0.001 | 7.51  (3.26 to 14.64) | 4.71  (2.18 to 10.96) | -1.47  (-1.61 to -1.33) | <0.001 |
|  | Zimbabwe | 7.18  (3.48 to 14.11) | 8.36  (4.16 to 16.13) | 0.49  (0.2 to 0.77) | 0.001 | 2.67  (1.34 to 5.22) | 2.97  (1.53 to 5.71) | 0.33  (0.08 to 0.59) | 0.009 |

**Abbreviations:** DALYs, Disability-Adjusted Life Years; AAPC, average annual percent change; CI, confidence interval.
