# Supplementary material for: Global, regional, and national burden of HIV and other sexually transmitted infections among women of childbearing age from 1990 to 2021
Source: Microbiol Spectr. 2025 Oct 24;13(12):e00488-25. doi: 10.1128/spectrum.00488-25 (PMC12671144; doi:10.1128/spectrum.00488-25)
Supplement: Table S5 — The age-standardized incidence, prevalence, mortality, and DALY rates of 204 countries and territories. [file spectrum.00488-25-s0006.docx]

**Table 5:** The age-standardized incidence, prevalence, mortality and DALYs rates of 204 countries and territories.

|  |  | Trichomoniasis | | | | Genital herpes | | | |
| --- | --- | --- | --- | --- | --- | --- | --- | --- | --- |
|  |  | 1990 | 2021 | AAPC (95% CI) | P | 1990 | 2021 | AAPC (95% CI) | P |
| Age-standardized Incidence | Afghanistan | 4023.89  (2195.70 to 6606.05) | 4005.24  (2211.79 to 6615.19) | -0.01  (-0.19 to 0.17) | 0.88 | 948.84  (636.34 to 1344.42) | 949.50  (636.34 to 1328.41) | 0  (-0.02 to 0.02) | 0.795 |
|  | Albania | 5025.39  (2753.08 to 8255.89) | 5086.82  (2809.63 to 8394.39) | 0.05  (0.01 to 0.09) | 0.009 | 563.70  (379.21 to 797.29) | 564.72  (375.15 to 799.88) | 0  (-0.02 to 0.02) | 0.902 |
|  | Algeria | 4098.83  (2259.48 to 6691.50) | 4098.70  (2232.11 to 6700.76) | -0.02  (-0.26 to 0.23) | 0.89 | 949.51  (640.18 to 1336.29) | 946.96  (635.61 to 1335.72) | -0.01  (-0.03 to 0.01) | 0.264 |
|  | American Samoa | 16556.91  (9436.89 to 26379.89) | 15718.57  (8929.92 to 25077.11) | -0.09  (-0.24 to 0.06) | 0.261 | 1221.44  (835.70 to 1671.97) | 1245.78  (856.53 to 1721.89) | 0.05  (0.03 to 0.08) | <0.001 |
|  | Andorra | 2039.42  (1113.07 to 3329.66) | 2084.02  (1129.24 to 3457.92) | 0.09  (-0.06 to 0.24) | 0.247 | 775.58  (524.47 to 1089.87) | 774.22  (521.21 to 1087.30) | -0.01  (-0.02 to 0) | 0.136 |
|  | Angola | 8260.89  (4609.16 to 13360.68) | 8379.62  (4668.09 to 13552.70) | 0.05  (0.01 to 0.09) | 0.02 | 3105.25  (2267.99 to 4091.03) | 3104.07  (2284.49 to 4092.54) | -0.01  (-0.03 to 0.01) | 0.5 |
|  | Antigua and Barbuda | 8789.61  (4949.68 to 14292.58) | 9067.25  (5000.53 to 14770.77) | 0.09  (0.02 to 0.15) | 0.007 | 2051.92  (1434.28 to 2773.56) | 2069.00  (1450.47 to 2797.00) | 0.03  (-0.01 to 0.07) | 0.202 |
|  | Argentina | 4015.88  (2201.38 to 6557.35) | 4119.97  (2255.67 to 6870.23) | 0.1  (-0.02 to 0.22) | 0.113 | 1916.87  (1671.63 to 2181.66) | 1869.76  (1293.72 to 2554.49) | -0.08  (-0.11 to -0.05) | <0.001 |
|  | Armenia | 7615.56  (4219.37 to 12441.45) | 7723.97  (4271.35 to 12598.69) | 0.05  (0.03 to 0.06) | <0.001 | 778.87  (524.53 to 1079.12) | 785.28  (528.02 to 1094.57) | 0.02  (0.01 to 0.04) | <0.001 |
|  | Australia | 3576.01  (1969.79 to 5918.02) | 3714.32  (2025.54 to 6071.65) | 0.13  (0.06 to 0.2) | <0.001 | 1243.86  (870.05 to 1697.58) | 961.75  (657.07 to 1337.67) | -0.81  (-0.88 to -0.74) | <0.001 |
|  | Austria | 2013.49  (1097.68 to 3277.45) | 2078.69  (1131.31 to 3438.24) | 0.11  (0.02 to 0.21) | 0.017 | 637.01  (429.49 to 888.43) | 643.72  (429.05 to 914.59) | 0.04  (0.01 to 0.07) | 0.015 |
|  | Azerbaijan | 7586.12  (4212.93 to 12275.53) | 7710.57  (4318.19 to 12372.29) | 0.05  (-0.04 to 0.14) | 0.307 | 780.40  (524.74 to 1083.31) | 784.63  (524.51 to 1101.04) | 0.02  (0 to 0.03) | 0.027 |
|  | Bahamas | 8979.32  (4920.14 to 14623.74) | 9063.36  (4975.96 to 14737.63) | 0.01  (-0.09 to 0.11) | 0.806 | 2048.80  (1426.79 to 2798.00) | 2077.49  (1445.49 to 2808.41) | 0.04  (0.02 to 0.05) | <0.001 |
|  | Bahrain | 4039.54  (2211.93 to 6621.64) | 4112.74  (2252.45 to 6773.70) | 0.08  (-0.12 to 0.28) | 0.442 | 948.84  (636.34 to 1344.42) | 950.11  (637.76 to 1327.65) | 0  (-0.01 to 0.02) | 0.827 |
|  | Bangladesh | 3602.22  (1968.03 to 6006.33) | 3802.53  (2070.66 to 6339.74) | 0.17  (0.16 to 0.18) | <0.001 | 614.65  (411.33 to 866.02) | 612.78  (406.57 to 867.10) | -0.01  (-0.01 to -0.01) | <0.001 |
|  | Barbados | 8967.43  (5015.42 to 14487.72) | 9077.33  (4972.21 to 14918.62) | 0.03  (-0.04 to 0.1) | 0.358 | 2044.65  (1440.03 to 2769.97) | 2071.93  (1437.79 to 2811.60) | 0.02  (-0.03 to 0.07) | 0.364 |
|  | Belarus | 3725.97  (2058.28 to 6222.98) | 3719.43  (2032.95 to 6177.64) | 0.07  (0.01 to 0.13) | 0.029 | 1150.24  (795.93 to 1597.96) | 1151.50  (797.21 to 1590.33) | 0  (-0.01 to 0.01) | 0.677 |
|  | Belgium | 1394.56  (772.77 to 2309.48) | 1472.11  (796.40 to 2466.10) | 0.19  (-0.06 to 0.45) | 0.137 | 790.63  (534.89 to 1102.76) | 794.43  (532.61 to 1115.31) | 0.01  (-0.01 to 0.04) | 0.324 |
|  | Belize | 8799.11  (4884.31 to 14455.68) | 8918.21  (4937.92 to 14583.00) | 0.04  (-0.03 to 0.12) | 0.221 | 2046.99  (1425.48 to 2768.19) | 2068.57  (1447.62 to 2809.58) | 0.03  (-0.01 to 0.06) | 0.176 |
|  | Benin | 9984.38  (5564.15 to 15992.77) | 10545.37  (5961.96 to 17115.45) | 0.22  (0.02 to 0.42) | 0.029 | 1925.82  (1350.68 to 2597.93) | 1934.39  (1353.66 to 2621.17) | 0.04  (0.01 to 0.07) | 0.013 |
|  | Bermuda | 8915.96  (5030.49 to 14599.91) | 9047.90  (5076.28 to 14603.35) | 0.04  (-0.02 to 0.11) | 0.194 | 2046.99  (1425.48 to 2768.19) | 2071.12  (1443.91 to 2796.49) | 0.03  (-0.03 to 0.08) | 0.33 |
|  | Bhutan | 3574.21  (1962.67 to 5878.88) | 3743.14  (2040.68 to 6207.21) | 0.15  (0.14 to 0.16) | <0.001 | 614.23  (409.49 to 861.14) | 613.69  (408.60 to 860.00) | 0  (-0.01 to 0.01) | 0.773 |
|  | Bolivia (Plurinational State of) | 6197.59  (3425.56 to 10125.24) | 6314.17  (3453.84 to 10274.67) | 0.04  (0 to 0.08) | 0.056 | 2352.53  (1654.95 to 3135.01) | 2352.92  (1652.23 to 3164.94) | 0.01  (-0.01 to 0.03) | 0.287 |
|  | Bosnia and Herzegovina | 4894.02  (2694.11 to 7974.98) | 5076.62  (2760.87 to 8280.01) | 0.13  (0.08 to 0.19) | <0.001 | 563.70  (379.21 to 797.29) | 564.72  (375.15 to 799.88) | 0  (-0.02 to 0.02) | 0.902 |
|  | Botswana | 21155.56  (12632.92 to 32501.91) | 20190.88  (11532.39 to 31642.77) | -0.16  (-0.31 to -0.02) | 0.025 | 2569.10  (1854.20 to 3383.06) | 2706.05  (1963.58 to 3554.25) | 0.16  (0.13 to 0.19) | <0.001 |
|  | Brazil | 10109.78  (5492.18 to 16451.87) | 9979.36  (5399.76 to 16368.21) | -0.04  (-0.09 to 0.01) | 0.088 | 2216.57  (1580.61 to 2961.04) | 2209.48  (1565.23 to 2959.21) | -0.01  (-0.04 to 0.03) | 0.592 |
|  | Brunei Darussalam | 4292.05  (2342.61 to 7037.27) | 4347.65  (2342.53 to 7176.01) | 0.06  (0.01 to 0.11) | 0.03 | 976.38  (659.28 to 1350.12) | 978.48  (662.21 to 1369.73) | 0.01  (-0.01 to 0.03) | 0.304 |
|  | Bulgaria | 4925.71  (2701.09 to 8087.11) | 5069.96  (2789.94 to 8424.16) | 0.12  (0.06 to 0.17) | <0.001 | 765.15  (505.38 to 1097.81) | 766.43  (506.48 to 1094.69) | 0  (-0.08 to 0.08) | 0.958 |
|  | Burkina Faso | 22402.60  (13324.61 to 34636.11) | 18441.82  (10488.75 to 29067.35) | -0.65  (-0.82 to -0.48) | <0.001 | 1905.08  (1328.00 to 2587.18) | 1890.36  (1321.51 to 2559.29) | -0.03  (-0.04 to -0.01) | <0.001 |
|  | Burundi | 15943.42  (9028.05 to 25202.37) | 16442.46  (9302.03 to 25591.91) | 0.1  (0.05 to 0.15) | <0.001 | 2220.76  (1590.25 to 2989.57) | 2220.36  (1583.00 to 2988.08) | -0.01  (-0.02 to 0.01) | 0.369 |
|  | Cabo Verde | 14209.50  (8024.51 to 22663.80) | 14679.77  (8282.28 to 23505.23) | 0.11  (0.08 to 0.14) | <0.001 | 2033.43  (1415.57 to 2737.71) | 2036.45  (1417.93 to 2745.41) | 0  (-0.01 to 0.01) | 0.804 |
|  | Cambodia | 6179.23  (3453.51 to 10062.37) | 5916.22  (3262.21 to 9781.23) | -0.07  (-0.22 to 0.08) | 0.373 | 1260.69  (868.93 to 1746.59) | 1263.89  (873.12 to 1735.24) | 0.01  (0 to 0.01) | <0.001 |
|  | Cameroon | 16614.74  (9449.81 to 26004.65) | 16912.16  (9572.71 to 26615.92) | 0.05  (-0.01 to 0.12) | 0.107 | 2382.33  (1683.93 to 3195.59) | 2408.03  (1696.17 to 3237.55) | 0  (-0.04 to 0.04) | 0.919 |
|  | Canada | 8423.40  (4624.45 to 13589.00) | 8801.31  (4883.60 to 14267.23) | 0.17  (-0.04 to 0.38) | 0.109 | 748.11  (507.58 to 1053.36) | 743.73  (507.13 to 1040.66) | 0.01  (-0.18 to 0.21) | 0.888 |
|  | Central African Republic | 8203.22  (4570.85 to 13210.92) | 8421.67  (4645.44 to 13735.30) | 0.11  (0.01 to 0.21) | 0.03 | 3117.04  (2289.66 to 4078.54) | 3099.30  (2287.89 to 4073.87) | -0.04  (-0.11 to 0.04) | 0.318 |
|  | Chad | 14214.15  (8167.22 to 22571.69) | 14651.94  (8356.72 to 23172.69) | 0.09  (0.04 to 0.13) | <0.001 | 1673.27  (1173.20 to 2273.59) | 1686.57  (1176.95 to 2292.94) | 0.02  (-0.02 to 0.05) | 0.406 |
|  | Chile | 4022.61  (2203.79 to 6595.66) | 4175.41  (2265.89 to 6871.12) | 0.12  (0.04 to 0.21) | 0.005 | 1862.26  (1294.97 to 2528.77) | 1867.32  (1299.25 to 2546.67) | 0.02  (-0.01 to 0.04) | 0.138 |
|  | China | 5085.78  (2726.99 to 8397.63) | 4750.07  (2547.05 to 7860.50) | -0.21  (-0.3 to -0.13) | <0.001 | 923.62  (625.04 to 1292.71) | 923.85  (629.97 to 1284.94) | -0.06  (-0.22 to 0.1) | 0.467 |
|  | Colombia | 13585.53  (7684.29 to 21542.96) | 13524.31  (7509.81 to 21835.46) | -0.06  (-0.22 to 0.11) | 0.487 | 2742.85  (2144.54 to 3401.95) | 2299.24  (1594.76 to 3137.06) | -0.6  (-0.77 to -0.43) | <0.001 |
|  | Comoros | 15962.06  (9092.22 to 25375.16) | 16451.72  (9189.39 to 26024.44) | 0.1  (0.03 to 0.17) | 0.004 | 2190.44  (1562.24 to 2943.71) | 2212.16  (1583.13 to 2948.36) | 0.03  (0.02 to 0.04) | <0.001 |
|  | Congo | 8132.73  (4518.63 to 13270.30) | 8346.30  (4680.99 to 13366.16) | 0.11  (0.06 to 0.15) | <0.001 | 3121.16  (2299.02 to 4050.85) | 3110.72  (2298.81 to 4052.28) | -0.04  (-0.1 to 0.02) | 0.248 |
|  | Cook Islands | 17653.29  (9874.66 to 28330.07) | 16036.05  (8906.30 to 25819.25) | -0.27  (-0.31 to -0.22) | <0.001 | 1223.85  (835.61 to 1687.20) | 1247.79  (858.35 to 1734.88) | 0.06  (0.05 to 0.07) | <0.001 |
|  | Costa Rica | 13585.83  (7662.14 to 21750.65) | 13418.15  (7492.81 to 21706.80) | -0.08  (-0.24 to 0.07) | 0.273 | 2196.35  (1759.76 to 2666.66) | 2104.23  (1471.13 to 2868.29) | -0.16  (-0.18 to -0.13) | <0.001 |
|  | Coted'Ivoire | 12588.74  (7109.84 to 20112.00) | 13019.81  (7296.42 to 20946.00) | 0.12  (0.08 to 0.15) | <0.001 | 2059.90  (1446.15 to 2765.49) | 2055.47  (1446.22 to 2778.43) | -0.01  (-0.02 to 0) | 0.007 |
|  | Croatia | 4960.54  (2743.63 to 8096.40) | 5131.22  (2826.82 to 8489.48) | 0.12  (0.09 to 0.15) | <0.001 | 627.84  (418.51 to 888.04) | 623.19  (415.25 to 876.84) | -0.05  (-0.17 to 0.06) | 0.366 |
|  | Cuba | 8878.20  (4988.20 to 14215.72) | 8996.64  (4980.44 to 14587.68) | 0.03  (-0.04 to 0.11) | 0.423 | 2044.22  (1443.34 to 2748.75) | 2068.52  (1453.73 to 2784.65) | 0.02  (-0.02 to 0.06) | 0.346 |
|  | Cyprus | 2013.12  (1096.24 to 3336.21) | 2059.54  (1136.97 to 3378.98) | 0.07  (-0.1 to 0.24) | 0.399 | 772.77  (518.45 to 1088.43) | 776.00  (523.55 to 1091.07) | 0.01  (0.01 to 0.02) | <0.001 |
|  | Czechia | 5010.01  (2758.83 to 8285.47) | 5147.26  (2811.02 to 8476.23) | 0.12  (0.09 to 0.15) | <0.001 | 423.27  (359.09 to 489.94) | 496.30  (330.95 to 695.31) | 0.53  (0.46 to 0.59) | <0.001 |
|  | Democratic People's Republic of Korea | 4903.61  (2703.21 to 8041.18) | 4696.54  (2572.11 to 7679.19) | -0.14  (-0.36 to 0.09) | 0.244 | 915.84  (614.55 to 1274.42) | 913.79  (617.72 to 1280.61) | -0.01  (-0.01 to 0) | 0.096 |
|  | Democratic Republic of the Congo | 8283.52  (4647.13 to 13316.19) | 8408.71  (4657.31 to 13705.70) | 0.06  (0.02 to 0.11) | 0.003 | 3116.49  (2281.05 to 4093.05) | 3096.46  (2293.16 to 4068.01) | -0.04  (-0.06 to -0.02) | <0.001 |
|  | Denmark | 2009.80  (1102.57 to 3310.24) | 2073.89  (1134.94 to 3435.71) | 0.12  (-0.04 to 0.28) | 0.148 | 1061.00  (706.59 to 1530.52) | 926.86  (619.14 to 1297.94) | -0.43  (-0.46 to -0.41) | <0.001 |
|  | Djibouti | 16111.49  (9097.36 to 25436.95) | 16571.70  (9464.15 to 26224.78) | 0.1  (0.05 to 0.14) | <0.001 | 2105.61  (1487.18 to 2817.94) | 2127.86  (1514.89 to 2879.30) | 0.04  (-0.03 to 0.12) | 0.276 |
|  | Dominica | 8848.78  (4937.37 to 14284.93) | 8957.33  (4928.67 to 14575.30) | 0.05  (0 to 0.09) | 0.031 | 2051.92  (1434.28 to 2773.56) | 2071.12  (1443.91 to 2796.49) | 0.02  (-0.03 to 0.07) | 0.422 |
|  | Dominican Republic | 8826.84  (4958.25 to 14219.46) | 8941.53  (4989.43 to 14450.87) | 0.05  (-0.02 to 0.11) | 0.15 | 2044.98  (1416.39 to 2768.52) | 2071.87  (1442.73 to 2798.90) | 0.04  (0 to 0.07) | 0.025 |
|  | Ecuador | 6205.21  (3410.78 to 10148.50) | 6350.91  (3493.92 to 10412.85) | 0.07  (0.04 to 0.1) | <0.001 | 2347.10  (1653.42 to 3128.06) | 2354.47  (1675.23 to 3161.12) | 0.02  (0 to 0.03) | 0.014 |
|  | Egypt | 5444.19  (2985.80 to 8709.23) | 5262.19  (2901.58 to 8512.94) | -0.14  (-0.31 to 0.02) | 0.089 | 949.51  (640.18 to 1336.29) | 950.11  (637.76 to 1327.65) | 0  (-0.03 to 0.03) | 0.941 |
|  | El Salvador | 13493.43  (7598.53 to 21620.99) | 13371.04  (7472.40 to 21347.43) | -0.08  (-0.23 to 0.07) | 0.317 | 2140.80  (1483.93 to 2919.64) | 2142.24  (1495.35 to 2901.30) | 0  (-0.02 to 0.03) | 0.731 |
|  | Equatorial Guinea | 8198.19  (4605.02 to 13255.07) | 8453.06  (4734.60 to 13713.82) | 0.11  (0.09 to 0.13) | <0.001 | 3108.70  (2309.73 to 4058.64) | 3124.69  (2304.16 to 4072.00) | 0  (-0.05 to 0.05) | 0.936 |
|  | Eritrea | 15950.78  (9085.24 to 25127.63) | 16294.96  (9264.58 to 25773.56) | 0.08  (0.01 to 0.14) | 0.023 | 1772.54  (1279.63 to 2339.57) | 1854.15  (1309.80 to 2523.71) | 0.14  (0.11 to 0.17) | <0.001 |
|  | Estonia | 3740.16  (2050.49 to 6130.90) | 3730.27  (2024.74 to 6268.47) | 0.04  (-0.02 to 0.11) | 0.178 | 1149.43  (796.78 to 1582.53) | 1153.72  (795.54 to 1593.30) | 0  (-0.02 to 0.03) | 0.732 |
|  | Eswatini | 20737.80  (12078.08 to 32100.86) | 21238.06  (12152.68 to 33607.28) | 0.09  (0.06 to 0.12) | <0.001 | 2557.86  (1860.67 to 3343.63) | 2819.23  (2042.05 to 3698.40) | 0.31  (0.3 to 0.33) | <0.001 |
|  | Ethiopia | 16149.12  (8952.94 to 26089.85) | 16330.66  (9020.89 to 26714.73) | 0.05  (0.02 to 0.08) | 0.001 | 1742.45  (1218.87 to 2363.28) | 1745.80  (1223.45 to 2376.62) | -0.05  (-0.21 to 0.12) | 0.581 |
|  | Fiji | 13937.63  (8494.33 to 21852.87) | 13769.83  (7803.52 to 22123.50) | 0.04  (-0.09 to 0.17) | 0.527 | 1221.44  (835.70 to 1671.97) | 1244.00  (852.54 to 1729.28) | 0.05  (0.04 to 0.07) | <0.001 |
|  | Finland | 2019.45  (1109.32 to 3317.50) | 2087.48  (1140.08 to 3396.81) | 0.13  (-0.03 to 0.28) | 0.116 | 1135.55  (855.23 to 1437.10) | 771.66  (523.25 to 1089.10) | -1.2  (-1.26 to -1.13) | <0.001 |
|  | France | 2727.73  (1479.36 to 4503.26) | 2664.84  (1449.43 to 4400.86) | -0.05  (-0.2 to 0.09) | 0.461 | 761.06  (511.20 to 1073.01) | 760.16  (512.80 to 1072.33) | 0  (-0.03 to 0.02) | 0.821 |
|  | Gabon | 8173.09  (4574.13 to 13110.50) | 8403.68  (4617.14 to 13696.28) | 0.1  (0.08 to 0.13) | <0.001 | 3118.66  (2289.78 to 4111.87) | 3113.93  (2282.78 to 4088.19) | -0.04  (-0.08 to 0) | 0.042 |
|  | Gambia | 14171.27  (8081.10 to 22670.37) | 14605.12  (8258.64 to 23347.01) | 0.1  (-0.02 to 0.22) | 0.099 | 1944.33  (1340.76 to 2658.16) | 1964.41  (1370.79 to 2679.46) | 0.03  (0.01 to 0.05) | 0.001 |
|  | Georgia | 7593.21  (4233.47 to 12408.22) | 7713.44  (4229.80 to 12555.32) | 0.03  (-0.09 to 0.16) | 0.582 | 778.87  (524.53 to 1079.12) | 787.20  (533.48 to 1087.68) | 0.03  (0.02 to 0.04) | <0.001 |
|  | Germany | 2002.36  (1107.18 to 3292.39) | 2059.94  (1125.59 to 3367.95) | 0.1  (-0.02 to 0.23) | 0.103 | 1121.80  (758.17 to 1590.40) | 925.30  (619.70 to 1298.07) | -0.57  (-0.78 to -0.36) | <0.001 |
|  | Ghana | 14049.18  (8001.10 to 22389.86) | 14558.37  (8196.49 to 23224.92) | 0.11  (0.09 to 0.14) | <0.001 | 2034.40  (1419.30 to 2751.10) | 2047.46  (1429.98 to 2798.04) | 0.02  (0.01 to 0.03) | <0.001 |
|  | Greece | 2926.94  (1596.68 to 4829.75) | 2919.51  (1574.51 to 4767.97) | -0.08  (-0.49 to 0.34) | 0.718 | 630.77  (427.53 to 877.95) | 630.79  (429.95 to 884.18) | 0  (-0.03 to 0.02) | 0.809 |
|  | Greenland | 8196.28  (4543.74 to 13184.73) | 8654.32  (4799.24 to 13907.26) | 0.21  (-0.05 to 0.46) | 0.107 | 940.16  (628.76 to 1326.59) | 935.07  (627.58 to 1318.05) | -0.02  (-0.02 to -0.01) | <0.001 |
|  | Grenada | 8811.98  (4929.26 to 14483.11) | 8939.86  (4914.68 to 14579.08) | 0.04  (-0.02 to 0.09) | 0.196 | 2048.89  (1431.65 to 2766.44) | 2065.63  (1448.70 to 2793.72) | 0.02  (-0.02 to 0.06) | 0.344 |
|  | Guam | 16701.04  (9483.92 to 26338.56) | 15810.28  (8865.89 to 25146.81) | -0.1  (-0.23 to 0.03) | 0.134 | 1222.43  (832.43 to 1687.26) | 1243.53  (846.29 to 1723.90) | 0.05  (0.04 to 0.06) | <0.001 |
|  | Guatemala | 13419.71  (7589.42 to 21497.63) | 13154.44  (7359.48 to 21215.98) | -0.1  (-0.19 to -0.01) | 0.025 | 2140.71  (1493.68 to 2907.96) | 2144.76  (1490.32 to 2900.31) | 0.01  (-0.02 to 0.03) | 0.674 |
|  | Guinea | 14144.11  (8067.33 to 22525.69) | 14624.50  (8277.75 to 23184.20) | 0.1  (0.06 to 0.14) | <0.001 | 2025.82  (1419.16 to 2727.70) | 2043.71  (1421.68 to 2778.48) | 0.03  (0.02 to 0.04) | <0.001 |
|  | Guinea-Bissau | 13893.45  (7837.39 to 22110.65) | 14412.49  (8168.84 to 22810.54) | 0.12  (0.05 to 0.18) | <0.001 | 2029.00  (1408.42 to 2754.05) | 2061.19  (1441.87 to 2803.40) | 0.05  (0.04 to 0.05) | <0.001 |
|  | Guyana | 8748.52  (4929.73 to 13997.33) | 8908.65  (4977.98 to 14440.83) | 0.05  (-0.02 to 0.12) | 0.16 | 2044.65  (1440.03 to 2769.97) | 2084.14  (1459.72 to 2807.41) | 0.06  (0.03 to 0.08) | <0.001 |
|  | Haiti | 8733.88  (4916.37 to 14216.41) | 8834.37  (4904.04 to 14409.34) | 0.03  (-0.01 to 0.08) | 0.158 | 1713.59  (1201.84 to 2315.72) | 1703.95  (1185.05 to 2320.31) | -0.05  (-0.07 to -0.03) | <0.001 |
|  | Honduras | 13418.22  (7587.54 to 21395.53) | 13211.52  (7342.25 to 21442.68) | -0.11  (-0.27 to 0.05) | 0.178 | 2478.09  (1741.04 to 3370.05) | 2487.18  (1757.23 to 3366.38) | 0.04  (-0.02 to 0.1) | 0.163 |
|  | Hungary | 4989.59  (2747.85 to 8231.86) | 5093.89  (2794.67 to 8369.61) | 0.08  (0.05 to 0.11) | <0.001 | 438.98  (292.86 to 614.28) | 438.69  (292.96 to 616.32) | 0.02  (-0.02 to 0.06) | 0.36 |
|  | Iceland | 2027.56  (1112.52 to 3354.13) | 2113.62  (1147.39 to 3502.49) | 0.16  (0.01 to 0.3) | 0.036 | 883.41  (588.35 to 1241.85) | 885.79  (593.14 to 1263.55) | 0.02  (-0.02 to 0.05) | 0.396 |
|  | India | 3751.33  (2008.41 to 6247.82) | 3860.47  (2057.29 to 6430.97) | 0.05  (-0.08 to 0.17) | 0.483 | 579.44  (388.25 to 815.43) | 635.83  (428.62 to 881.73) | 0.3  (0.28 to 0.33) | <0.001 |
|  | Indonesia | 6402.66  (3453.52 to 10577.89) | 6078.85  (3272.42 to 10068.15) | -0.1  (-0.14 to -0.06) | <0.001 | 1068.81  (728.12 to 1476.34) | 1071.49  (737.79 to 1474.78) | 0.02  (0 to 0.03) | 0.04 |
|  | Iran (Islamic Republic of) | 3694.39  (1983.65 to 6162.19) | 3685.67  (1975.76 to 6159.45) | 0.29  (-0.36 to 0.96) | 0.382 | 1139.72  (775.84 to 1584.22) | 1143.70  (776.68 to 1581.32) | 0.01  (-0.02 to 0.05) | 0.367 |
|  | Iraq | 7581.21  (4198.92 to 12247.66) | 5842.59  (3213.83 to 9438.41) | -0.87  (-1.06 to -0.67) | <0.001 | 949.51  (640.18 to 1336.29) | 949.50  (636.34 to 1328.41) | 0  (-0.01 to 0.02) | 0.724 |
|  | Ireland | 2014.66  (1107.92 to 3380.61) | 2090.40  (1154.48 to 3440.84) | 0.13  (-0.01 to 0.28) | 0.078 | 775.58  (524.47 to 1089.87) | 774.24  (518.67 to 1091.30) | -0.01  (-0.01 to 0) | 0.06 |
|  | Israel | 1983.97  (1086.42 to 3253.45) | 2052.43  (1118.91 to 3345.90) | 0.12  (0.02 to 0.22) | 0.017 | 761.57  (516.71 to 1049.27) | 744.21  (501.27 to 1043.38) | -0.1  (-0.27 to 0.07) | 0.243 |
|  | Italy | 2331.86  (1257.69 to 3827.42) | 2370.01  (1280.94 to 3860.38) | 0.06  (-0.01 to 0.13) | 0.107 | 657.99  (443.17 to 920.15) | 657.33  (440.56 to 919.39) | 0  (-0.02 to 0.02) | 0.941 |
|  | Jamaica | 8841.83  (5001.72 to 14184.31) | 8963.34  (4949.09 to 14493.51) | 0.04  (-0.03 to 0.11) | 0.286 | 2812.67  (2071.33 to 3526.10) | 2440.92  (1730.23 to 3291.89) | -0.46  (-0.57 to -0.35) | <0.001 |
|  | Japan | 4564.03  (2448.25 to 7608.07) | 4520.42  (2405.41 to 7526.69) | -0.03  (-0.08 to 0.03) | 0.323 | 680.59  (452.36 to 961.52) | 703.87  (471.17 to 988.49) | 0.1  (0.04 to 0.17) | 0.002 |
|  | Jordan | 2846.81  (1568.04 to 4722.59) | 2916.39  (1609.68 to 4819.22) | 0.07  (-0.01 to 0.16) | 0.082 | 1008.59  (669.49 to 1438.52) | 1005.19  (670.00 to 1407.44) | -0.02  (-0.04 to 0.01) | 0.189 |
|  | Kazakhstan | 7487.02  (4201.07 to 12299.54) | 7671.90  (4201.94 to 12521.19) | 0.08  (0.05 to 0.12) | <0.001 | 780.40  (524.74 to 1083.31) | 784.10  (528.11 to 1097.14) | 0.02  (0 to 0.03) | 0.019 |
|  | Kenya | 12759.48  (7024.03 to 20692.70) | 15162.09  (8339.40 to 24660.91) | 0.56  (0.38 to 0.73) | <0.001 | 2317.26  (1653.67 to 3084.14) | 2333.76  (1665.39 to 3112.13) | 0  (-0.16 to 0.15) | 0.956 |
|  | Kiribati | 16319.68  (9243.56 to 25965.72) | 15443.58  (8746.88 to 24497.56) | -0.14  (-0.2 to -0.09) | <0.001 | 1220.13  (836.33 to 1673.01) | 1244.34  (848.80 to 1711.20) | 0.06  (0.05 to 0.07) | <0.001 |
|  | Kuwait | 3034.85  (1666.26 to 5005.34) | 3049.72  (1658.85 to 5078.67) | -0.03  (-0.29 to 0.24) | 0.829 | 949.51  (640.18 to 1336.29) | 949.50  (634.93 to 1326.97) | 0  (-0.02 to 0.02) | 0.714 |
|  | Kyrgyzstan | 7480.55  (4134.61 to 12155.81) | 7612.40  (4235.33 to 12294.80) | 0.05  (0.02 to 0.08) | 0.001 | 780.40  (524.74 to 1083.31) | 786.05  (533.38 to 1094.12) | 0.02  (0.01 to 0.03) | <0.001 |
|  | Lao People's Democratic Republic | 6143.33  (3405.88 to 9899.20) | 5900.59  (3266.15 to 9576.93) | -0.04  (-0.32 to 0.23) | 0.752 | 1260.69  (868.93 to 1746.59) | 1262.03  (860.83 to 1762.04) | 0  (0 to 0.01) | 0.348 |
|  | Latvia | 3761.13  (2065.18 to 6132.05) | 3684.37  (2012.64 to 6001.82) | 0.03  (-0.05 to 0.11) | 0.424 | 1151.56  (798.36 to 1589.67) | 1151.50  (797.21 to 1590.33) | 0  (0 to 0) | 0.556 |
|  | Lebanon | 2795.59  (1551.43 to 4629.72) | 2882.41  (1572.12 to 4751.38) | 0.14  (-0.17 to 0.46) | 0.363 | 948.84  (636.34 to 1344.42) | 950.23  (633.73 to 1353.83) | 0.01  (-0.01 to 0.02) | 0.536 |
|  | Lesotho | 20598.11  (11913.69 to 31806.68) | 20989.53  (11913.06 to 33029.36) | 0.09  (0.06 to 0.11) | <0.001 | 2574.42  (1857.59 to 3417.12) | 2818.83  (2053.59 to 3695.70) | 0.29  (0.28 to 0.31) | <0.001 |
|  | Liberia | 14029.64  (7947.68 to 22291.59) | 14460.99  (8167.44 to 22992.36) | 0.1  (0.07 to 0.12) | <0.001 | 2028.50  (1413.42 to 2730.09) | 2035.49  (1426.00 to 2755.74) | 0.01  (0 to 0.01) | 0.037 |
|  | Libya | 2813.60  (1548.45 to 4565.69) | 2838.56  (1554.16 to 4639.72) | 0.08  (-0.17 to 0.33) | 0.529 | 949.51  (640.18 to 1336.29) | 949.50  (636.34 to 1328.41) | 0  (-0.02 to 0.02) | 0.837 |
|  | Lithuania | 3740.45  (2073.25 to 6174.34) | 3711.82  (2008.72 to 6108.26) | 0.03  (-0.07 to 0.12) | 0.588 | 1150.24  (795.93 to 1597.96) | 1153.61  (795.73 to 1602.18) | 0.01  (0 to 0.01) | <0.001 |
|  | Luxembourg | 2023.75  (1114.09 to 3350.12) | 2082.07  (1130.87 to 3425.94) | 0.11  (-0.05 to 0.26) | 0.172 | 774.02  (522.87 to 1087.55) | 775.92  (522.98 to 1104.14) | 0  (-0.01 to 0.01) | 0.58 |
|  | Madagascar | 16071.29  (9199.54 to 25232.17) | 16452.03  (9355.17 to 25711.80) | 0.08  (0 to 0.17) | 0.064 | 2190.44  (1562.24 to 2943.71) | 2210.63  (1583.61 to 2929.67) | 0.03  (0.01 to 0.04) | <0.001 |
|  | Malawi | 11436.24  (6489.61 to 18160.19) | 12101.67  (6792.73 to 19523.84) | 0.24  (-0.21 to 0.69) | 0.292 | 2570.36  (1987.88 to 3200.46) | 2472.41  (1752.57 to 3319.27) | -0.13  (-0.16 to -0.09) | <0.001 |
|  | Malaysia | 6182.07  (3435.00 to 10126.15) | 6020.41  (3343.77 to 9882.63) | -0.02  (-0.33 to 0.29) | 0.905 | 1257.85  (862.16 to 1745.25) | 1262.24  (870.11 to 1736.37) | 0.01  (0 to 0.03) | 0.084 |
|  | Maldives | 6174.72  (3405.30 to 10024.77) | 6003.66  (3307.29 to 9777.57) | -0.01  (-0.29 to 0.27) | 0.946 | 1260.34  (861.52 to 1733.49) | 1259.37  (863.43 to 1751.52) | 0  (-0.02 to 0.01) | 0.749 |
|  | Mali | 19248.40  (10956.29 to 30169.98) | 19399.95  (11062.04 to 30419.92) | -0.01  (-0.11 to 0.1) | 0.92 | 2856.71  (2319.18 to 3446.97) | 2376.51  (1691.07 to 3197.83) | -0.59  (-0.64 to -0.55) | <0.001 |
|  | Malta | 2000.22  (1117.37 to 3257.30) | 2065.02  (1137.73 to 3391.03) | 0.12  (0 to 0.24) | 0.059 | 775.58  (524.47 to 1089.87) | 774.24  (518.67 to 1091.30) | -0.01  (-0.01 to 0) | 0.046 |
|  | Marshall Islands | 16139.45  (9217.31 to 25711.74) | 15352.77  (8683.51 to 24296.25) | -0.07  (-0.19 to 0.04) | 0.217 | 1223.85  (835.61 to 1687.20) | 1243.18  (852.78 to 1710.71) | 0.05  (0.03 to 0.07) | <0.001 |
|  | Mauritania | 14340.07  (8147.24 to 22860.68) | 14652.26  (8272.59 to 23323.97) | 0.06  (0.04 to 0.09) | <0.001 | 2029.00  (1408.42 to 2754.05) | 2039.94  (1427.82 to 2752.58) | 0.01  (0 to 0.03) | 0.079 |
|  | Mauritius | 6160.97  (3400.68 to 10026.68) | 5990.83  (3330.35 to 9936.53) | -0.02  (-0.3 to 0.26) | 0.892 | 1260.34  (861.52 to 1733.49) | 1262.24  (870.11 to 1736.37) | 0  (0 to 0.01) | 0.28 |
|  | Mexico | 14002.94  (7703.96 to 22791.09) | 13496.72  (7356.25 to 21992.18) | -0.15  (-0.29 to 0) | 0.044 | 1628.76  (1122.85 to 2231.22) | 1670.93  (1156.65 to 2263.39) | 0.09  (0.02 to 0.16) | 0.011 |
|  | Micronesia (Federated States of) | 16335.44  (9411.12 to 25881.50) | 15571.48  (8784.45 to 24783.77) | -0.07  (-0.29 to 0.15) | 0.508 | 1223.85  (835.61 to 1687.20) | 1245.26  (853.64 to 1716.75) | 0.05  (0.04 to 0.07) | <0.001 |
|  | Monaco | 2181.38  (1197.38 to 3546.44) | 2165.50  (1163.49 to 3634.78) | -0.01  (-0.09 to 0.08) | 0.906 | 775.58  (524.47 to 1089.87) | 774.22  (521.21 to 1087.30) | -0.01  (-0.02 to 0) | 0.09 |
|  | Mongolia | 7499.34  (4191.64 to 12074.01) | 7582.98  (4167.33 to 12431.86) | 0.04  (-0.05 to 0.14) | 0.382 | 778.87  (524.53 to 1079.12) | 782.80  (527.49 to 1087.86) | 0.02  (0.01 to 0.03) | 0.003 |
|  | Montenegro | 4970.96  (2734.85 to 8217.95) | 5104.08  (2796.26 to 8490.26) | 0.1  (0.06 to 0.13) | <0.001 | 563.70  (379.21 to 797.29) | 565.74  (376.20 to 802.02) | 0.01  (0 to 0.02) | 0.21 |
|  | Morocco | 4724.73  (2607.26 to 7759.58) | 4672.07  (2532.02 to 7738.83) | -0.05  (-0.3 to 0.2) | 0.698 | 1515.52  (1281.59 to 1762.72) | 1079.65  (717.10 to 1535.71) | -1.07  (-1.1 to -1.04) | <0.001 |
|  | Mozambique | 22787.08  (13267.08 to 35384.33) | 19970.66  (11512.22 to 31199.23) | -0.42  (-0.47 to -0.38) | <0.001 | 2495.64  (1794.88 to 3339.99) | 2576.11  (1854.82 to 3422.05) | 0.1  (0.09 to 0.12) | <0.001 |
|  | Myanmar | 6162.53  (3352.12 to 10141.38) | 5910.88  (3244.93 to 9731.96) | -0.09  (-0.23 to 0.05) | 0.224 | 1255.43  (866.71 to 1734.33) | 1259.88  (862.56 to 1750.98) | 0.01  (0 to 0.03) | 0.153 |
|  | Namibia | 20449.44  (11815.34 to 31907.87) | 21211.87  (12140.10 to 33315.47) | 0.13  (0.08 to 0.18) | <0.001 | 2569.64  (1843.79 to 3406.16) | 2716.76  (1980.56 to 3589.43) | 0.18  (0.16 to 0.2) | <0.001 |
|  | Nauru | 17675.60  (9862.24 to 28433.66) | 16255.17  (9130.60 to 26154.54) | -0.23  (-0.35 to -0.11) | <0.001 | 1223.85  (835.61 to 1687.20) | 1247.79  (858.35 to 1734.88) | 0.06  (0.05 to 0.07) | <0.001 |
|  | Nepal | 3609.83  (1987.74 to 5879.89) | 3737.89  (2068.86 to 6196.12) | 0.1  (0.07 to 0.13) | <0.001 | 614.65  (411.33 to 866.02) | 613.69  (408.60 to 860.00) | 0  (-0.01 to 0) | <0.001 |
|  | Netherlands | 1434.84  (787.13 to 2386.96) | 1503.32  (813.35 to 2480.06) | 0.18  (-0.01 to 0.36) | 0.066 | 939.39  (630.94 to 1336.81) | 939.43  (630.09 to 1325.49) | -0.01  (-0.05 to 0.02) | 0.474 |
|  | New Zealand | 3725.52  (1971.12 to 6194.35) | 3864.91  (2052.12 to 6500.72) | 0.13  (0.02 to 0.24) | 0.017 | 676.10  (454.72 to 953.99) | 685.55  (467.09 to 948.04) | 0.05  (0.03 to 0.07) | <0.001 |
|  | Nicaragua | 13637.46  (7692.45 to 21931.17) | 13355.22  (7362.41 to 21487.23) | -0.08  (-0.24 to 0.07) | 0.281 | 2141.26  (1502.98 to 2904.21) | 2142.95  (1494.89 to 2915.56) | 0.01  (0 to 0.03) | 0.16 |
|  | Niger | 14096.22  (8008.71 to 22516.71) | 14649.14  (8223.62 to 23268.26) | 0.12  (-0.05 to 0.3) | 0.166 | 2028.99  (1411.58 to 2768.25) | 2033.34  (1418.80 to 2736.58) | 0  (-0.01 to 0.01) | 0.571 |
|  | Nigeria | 14840.26  (8279.63 to 23759.02) | 16720.15  (9306.46 to 26867.72) | 0.34  (0.1 to 0.58) | 0.005 | 2325.62  (1667.38 to 3087.51) | 2308.65  (1649.76 to 3079.12) | -0.03  (-0.06 to 0) | 0.046 |
|  | Niue | 17560.35  (9889.27 to 28100.72) | 16223.51  (9019.97 to 26036.11) | -0.19  (-0.31 to -0.07) | 0.002 | 1223.85  (835.61 to 1687.20) | 1247.79  (858.35 to 1734.88) | 0.06  (0.05 to 0.07) | <0.001 |
|  | North Macedonia | 4943.53  (2689.23 to 8105.75) | 5044.14  (2784.25 to 8281.23) | 0.07  (0.01 to 0.14) | 0.018 | 563.70  (379.21 to 797.29) | 564.72  (375.15 to 799.88) | 0  (-0.02 to 0.02) | 0.902 |
|  | Northern Mariana Islands | 16795.64  (9652.18 to 26559.17) | 15748.20  (8885.42 to 24999.48) | -0.16  (-0.28 to -0.05) | 0.005 | 1221.44  (835.70 to 1671.97) | 1245.23  (857.30 to 1717.91) | 0.06  (0.04 to 0.07) | <0.001 |
|  | Norway | 2180.27  (1165.88 to 3625.74) | 2219.23  (1191.37 to 3683.97) | 0.07  (-0.07 to 0.22) | 0.339 | 965.62  (654.74 to 1348.66) | 875.58  (597.92 to 1218.12) | -0.32  (-0.34 to -0.3) | <0.001 |
|  | Oman | 4087.14  (2204.08 to 6785.44) | 4096.54  (2267.63 to 6739.63) | 0  (-0.16 to 0.17) | 0.98 | 948.84  (636.34 to 1344.42) | 946.96  (635.61 to 1335.72) | -0.01  (-0.03 to 0.01) | 0.237 |
|  | Pakistan | 4182.99  (2259.91 to 6825.58) | 4272.86  (2323.91 to 7044.55) | 0.07  (-0.01 to 0.14) | 0.089 | 609.54  (411.07 to 856.81) | 610.91  (413.08 to 859.34) | 0.01  (0 to 0.02) | 0.13 |
|  | Palau | 17563.02  (9815.52 to 28487.04) | 16227.16  (9022.40 to 26223.31) | -0.2  (-0.3 to -0.1) | <0.001 | 1223.85  (835.61 to 1687.20) | 1247.58  (858.05 to 1732.23) | 0.06  (0.04 to 0.08) | <0.001 |
|  | Palestine | 5537.26  (3089.18 to 8932.69) | 5438.95  (2965.83 to 8950.78) | -0.07  (-0.19 to 0.05) | 0.252 | 949.51  (640.18 to 1336.29) | 950.11  (637.76 to 1327.65) | 0  (-0.03 to 0.02) | 0.88 |
|  | Panama | 13478.39  (7654.17 to 21428.48) | 13444.11  (7539.99 to 21675.35) | -0.07  (-0.23 to 0.1) | 0.444 | 2135.35  (1479.12 to 2919.06) | 2157.52  (1483.25 to 2962.38) | 0.03  (0.03 to 0.04) | <0.001 |
|  | Papua New Guinea | 18356.74  (10502.18 to 28535.47) | 17092.47  (9696.03 to 26987.04) | -0.18  (-0.54 to 0.18) | 0.331 | 1475.58  (1012.55 to 2026.20) | 1496.99  (1025.44 to 2071.31) | 0.02  (-0.03 to 0.08) | 0.35 |
|  | Paraguay | 9720.36  (5496.59 to 15676.88) | 9768.21  (5391.26 to 16049.11) | 0.01  (-0.11 to 0.12) | 0.924 | 2188.57  (1523.65 to 2954.08) | 2198.99  (1528.05 to 2993.50) | 0.01  (0.01 to 0.02) | <0.001 |
|  | Peru | 6237.59  (3422.36 to 10216.58) | 6376.26  (3537.57 to 10445.86) | 0.07  (0.04 to 0.09) | <0.001 | 2277.36  (1773.87 to 2833.70) | 2356.24  (1671.55 to 3152.76) | 0.12  (0.08 to 0.17) | <0.001 |
|  | Philippines | 7075.00  (3872.77 to 11509.01) | 6803.98  (3683.45 to 11122.89) | -0.04  (-0.34 to 0.26) | 0.784 | 1101.52  (745.59 to 1525.26) | 1128.78  (773.03 to 1546.73) | 0.08  (0.06 to 0.11) | <0.001 |
|  | Poland | 5630.05  (3034.43 to 9336.51) | 5824.85  (3149.88 to 9661.88) | 0.16  (0.13 to 0.18) | <0.001 | 480.77  (320.42 to 673.96) | 489.64  (326.65 to 689.49) | 0.03  (-0.05 to 0.1) | 0.468 |
|  | Portugal | 2334.17  (1279.68 to 3824.35) | 2365.44  (1275.74 to 3881.87) | 0.05  (-0.08 to 0.19) | 0.452 | 774.27  (522.50 to 1090.86) | 777.03  (521.84 to 1083.97) | 0.01  (0.01 to 0.01) | <0.001 |
|  | Puerto Rico | 8923.33  (5023.14 to 14436.53) | 9006.41  (4957.42 to 14465.81) | 0.03  (-0.04 to 0.1) | 0.434 | 2049.68  (1424.07 to 2773.61) | 2076.55  (1441.20 to 2788.99) | 0.02  (-0.02 to 0.07) | 0.284 |
|  | Qatar | 4074.82  (2247.90 to 6574.49) | 4137.97  (2286.68 to 6869.14) | 0.05  (-0.09 to 0.18) | 0.509 | 948.84  (636.34 to 1344.42) | 950.11  (637.76 to 1327.65) | 0  (-0.02 to 0.02) | 0.95 |
|  | Republic of Korea | 4387.28  (2410.67 to 7309.90) | 4442.33  (2444.02 to 7294.02) | 0.06  (-0.05 to 0.17) | 0.304 | 2151.76  (1866.05 to 2447.73) | 1292.04  (879.56 to 1827.49) | -1.62  (-1.71 to -1.52) | <0.001 |
|  | Republic of Moldova | 3704.07  (2020.43 to 6107.94) | 3634.64  (1991.43 to 6040.70) | 0.04  (-0.03 to 0.11) | 0.278 | 1151.66  (794.88 to 1593.43) | 1149.23  (793.83 to 1566.87) | -0.01  (-0.01 to 0) | 0.032 |
|  | Romania | 4964.27  (2722.61 to 8217.00) | 5130.02  (2843.36 to 8372.08) | 0.1  (0.08 to 0.13) | <0.001 | 563.70  (379.21 to 797.29) | 562.94  (376.39 to 788.95) | 0  (-0.01 to 0.01) | 0.997 |
|  | Russian Federation | 3851.24  (2060.24 to 6418.84) | 3769.81  (2006.18 to 6311.97) | -0.01  (-0.1 to 0.09) | 0.915 | 1122.61  (775.76 to 1543.61) | 1125.49  (783.92 to 1535.13) | 0.01  (0 to 0.02) | 0.02 |
|  | Rwanda | 16200.80  (9281.77 to 25694.56) | 16457.23  (9302.00 to 26045.41) | 0.06  (0.01 to 0.11) | 0.03 | 2092.21  (1478.92 to 2829.32) | 2128.53  (1518.09 to 2845.87) | 0.06  (0.05 to 0.07) | <0.001 |
|  | Saint Kitts and Nevis | 9492.31  (5179.21 to 15625.08) | 9341.94  (5124.52 to 15171.10) | -0.06  (-0.11 to -0.01) | 0.015 | 2046.99  (1425.48 to 2768.19) | 2072.31  (1441.66 to 2804.49) | 0.02  (-0.02 to 0.06) | 0.256 |
|  | Saint Lucia | 8840.60  (4844.78 to 14386.64) | 8986.30  (4993.41 to 14689.82) | 0.05  (-0.02 to 0.12) | 0.187 | 2044.27  (1428.77 to 2748.98) | 2069.53  (1447.35 to 2815.48) | 0.03  (0.02 to 0.04) | <0.001 |
|  | Saint Vincent and the Grenadines | 8759.75  (4805.75 to 14128.50) | 8883.79  (4908.60 to 14462.08) | 0.05  (-0.01 to 0.11) | 0.076 | 2044.65  (1440.03 to 2769.97) | 2070.25  (1453.09 to 2818.74) | 0.02  (0 to 0.05) | 0.113 |
|  | Samoa | 16423.23  (9310.87 to 25737.22) | 15681.76  (8865.36 to 25020.30) | -0.1  (-0.21 to 0.01) | 0.074 | 1223.85  (835.61 to 1687.20) | 1243.18  (852.78 to 1710.71) | 0.05  (0.03 to 0.07) | <0.001 |
|  | San Marino | 2180.42  (1196.48 to 3547.65) | 2165.50  (1163.49 to 3634.78) | 0  (-0.12 to 0.12) | 0.945 | 775.58  (524.47 to 1089.87) | 774.22  (521.21 to 1087.30) | -0.01  (-0.02 to 0.01) | 0.282 |
|  | Sao Tome and Principe | 14103.09  (8068.63 to 22407.78) | 14530.16  (8186.60 to 22862.01) | 0.1  (0.03 to 0.16) | 0.003 | 2029.56  (1409.69 to 2746.28) | 2042.93  (1432.35 to 2759.75) | 0.02  (0.01 to 0.04) | <0.001 |
|  | Saudi Arabia | 4584.86  (2519.79 to 7503.02) | 4590.38  (2519.45 to 7677.11) | 0  (-0.22 to 0.21) | 0.967 | 948.84  (636.34 to 1344.42) | 947.74  (639.83 to 1324.69) | -0.01  (-0.03 to 0.01) | 0.441 |
|  | Senegal | 14162.75  (8045.49 to 22440.67) | 14561.20  (8138.72 to 23338.98) | 0.09  (0.04 to 0.14) | 0.001 | 1698.37  (1176.90 to 2304.04) | 1680.79  (1167.47 to 2288.84) | -0.04  (-0.06 to -0.03) | <0.001 |
|  | Serbia | 4951.70  (2738.96 to 8157.71) | 5068.59  (2786.01 to 8334.98) | 0.08  (0.06 to 0.1) | <0.001 | 563.70  (379.21 to 797.29) | 563.02  (376.05 to 786.82) | -0.01  (-0.01 to 0) | <0.001 |
|  | Seychelles | 6149.38  (3442.93 to 10024.54) | 6007.83  (3303.91 to 9829.23) | -0.05  (-0.17 to 0.08) | 0.457 | 1258.59  (865.12 to 1752.52) | 1262.28  (860.81 to 1752.15) | 0.01  (0 to 0.03) | 0.159 |
|  | Sierra Leone | 14009.02  (7978.94 to 22195.21) | 14493.24  (8240.99 to 22943.00) | 0.11  (0.09 to 0.14) | <0.001 | 2032.69  (1412.35 to 2749.60) | 2045.72  (1433.61 to 2765.49) | 0.02  (0.01 to 0.03) | <0.001 |
|  | Singapore | 4341.79  (2386.82 to 7129.28) | 4437.99  (2430.78 to 7203.47) | 0.08  (-0.03 to 0.18) | 0.152 | 976.38  (659.28 to 1350.12) | 977.49  (659.82 to 1365.08) | 0.01  (-0.01 to 0.03) | 0.292 |
|  | Slovakia | 4975.99  (2704.69 to 8056.44) | 5130.63  (2814.41 to 8425.22) | 0.11  (0.07 to 0.15) | <0.001 | 563.70  (379.21 to 797.29) | 564.72  (375.15 to 799.88) | 0  (-0.02 to 0.02) | 0.902 |
|  | Slovenia | 5020.79  (2789.66 to 8200.29) | 5142.32  (2812.13 to 8325.00) | 0.09  (0.06 to 0.12) | <0.001 | 563.70  (379.21 to 797.29) | 563.02  (376.05 to 786.82) | -0.01  (-0.01 to 0) | <0.001 |
|  | Solomon Islands | 16279.27  (9267.12 to 25509.13) | 15217.70  (8619.33 to 24033.94) | -0.11  (-0.28 to 0.06) | 0.189 | 1223.85  (835.61 to 1687.20) | 1245.26  (853.64 to 1716.75) | 0.05  (0.04 to 0.07) | <0.001 |
|  | Somalia | 16105.10  (9302.69 to 25173.12) | 16478.39  (9369.36 to 26021.43) | 0.09  (0.01 to 0.17) | 0.029 | 2192.56  (1546.84 to 2946.78) | 2208.65  (1580.58 to 2956.97) | 0.02  (0.01 to 0.03) | <0.001 |
|  | South Africa | 24533.69  (13850.05 to 38512.93) | 21204.92  (11886.12 to 33890.18) | -0.46  (-0.62 to -0.3) | <0.001 | 2739.30  (2015.75 to 3546.51) | 2881.90  (2127.05 to 3754.65) | 0.18  (0.13 to 0.22) | <0.001 |
|  | South Sudan | 16026.79  (9220.42 to 25032.84) | 16462.13  (9253.51 to 26125.22) | 0.11  (0.07 to 0.16) | <0.001 | 2190.28  (1548.64 to 2952.81) | 2221.80  (1583.63 to 2984.96) | 0.04  (0.03 to 0.05) | <0.001 |
|  | Spain | 2018.14  (1092.71 to 3296.27) | 2070.53  (1130.11 to 3404.06) | 0.1  (-0.03 to 0.22) | 0.125 | 546.06  (385.69 to 734.71) | 615.03  (412.68 to 856.64) | 0.4  (0.35 to 0.46) | <0.001 |
|  | Sri Lanka | 8534.16  (4778.02 to 13752.55) | 7966.10  (4376.22 to 12846.09) | -0.18  (-0.32 to -0.05) | 0.006 | 1260.34  (861.52 to 1733.49) | 1260.56  (866.11 to 1744.39) | 0  (-0.01 to 0.01) | 0.655 |
|  | Sudan | 5968.49  (3302.76 to 9859.23) | 5820.15  (3223.69 to 9426.79) | 0  (-0.18 to 0.18) | 0.991 | 947.97  (638.32 to 1320.90) | 948.52  (638.24 to 1335.63) | -0.01  (-0.02 to 0.01) | 0.595 |
|  | Suriname | 8915.88  (4989.63 to 14364.13) | 9005.49  (4989.40 to 14550.18) | 0.03  (-0.04 to 0.1) | 0.397 | 2046.99  (1425.48 to 2768.19) | 2073.73  (1447.02 to 2808.28) | 0.02  (0 to 0.05) | 0.081 |
|  | Sweden | 2117.42  (1134.18 to 3551.43) | 2180.98  (1144.61 to 3670.04) | 0.11  (-0.02 to 0.23) | 0.094 | 1209.21  (844.56 to 1643.41) | 1198.43  (812.69 to 1640.04) | -0.04  (-0.1 to 0.03) | 0.251 |
|  | Switzerland | 2028.32  (1105.41 to 3352.38) | 2081.24  (1139.29 to 3439.09) | 0.1  (0 to 0.21) | 0.047 | 916.14  (699.75 to 1160.46) | 817.97  (549.70 to 1163.58) | -0.38  (-0.43 to -0.32) | <0.001 |
|  | Syrian Arab Republic | 4144.38  (2262.58 to 6845.51) | 4175.06  (2285.68 to 6859.86) | 0.03  (-0.16 to 0.22) | 0.79 | 748.70  (503.70 to 1050.25) | 760.55  (513.20 to 1061.13) | 0.04  (0.02 to 0.06) | <0.001 |
|  | Taiwan (Province of China) | 4726.17  (2794.44 to 7249.02) | 4858.29  (2660.65 to 7928.20) | 0.14  (-0.1 to 0.38) | 0.25 | 915.84  (614.55 to 1274.42) | 911.76  (621.74 to 1265.58) | -0.01  (-0.03 to 0) | 0.127 |
|  | Tajikistan | 7516.95  (4202.23 to 12194.25) | 7601.47  (4159.59 to 12456.10) | 0.04  (0.03 to 0.06) | <0.001 | 780.40  (524.74 to 1083.31) | 787.20  (533.48 to 1087.68) | 0.03  (0.01 to 0.05) | 0.002 |
|  | Thailand | 6234.46  (3474.33 to 9974.77) | 6007.87  (3358.12 to 9807.69) | -0.06  (-0.19 to 0.07) | 0.357 | 1766.76  (1441.25 to 2133.26) | 1606.54  (1112.82 to 2222.78) | -0.35  (-0.49 to -0.22) | <0.001 |
|  | Timor-Leste | 6205.67  (3429.02 to 10029.94) | 5952.00  (3290.06 to 9991.70) | -0.06  (-0.38 to 0.25) | 0.692 | 1258.45  (869.52 to 1736.35) | 1261.04  (866.31 to 1737.23) | 0.01  (-0.02 to 0.04) | 0.473 |
|  | Togo | 10539.31  (5956.43 to 16904.44) | 11060.28  (6224.69 to 17643.30) | 0.17  (0.08 to 0.25) | <0.001 | 2030.60  (1406.09 to 2746.21) | 2051.19  (1442.92 to 2767.13) | 0.03  (0.01 to 0.05) | 0.004 |
|  | Tokelau | 17640.10  (9877.74 to 28071.01) | 16095.57  (8944.92 to 25550.17) | -0.22  (-0.37 to -0.08) | 0.003 | 1223.85  (835.61 to 1687.20) | 1247.79  (858.35 to 1734.88) | 0.06  (0.05 to 0.07) | <0.001 |
|  | Tonga | 16330.62  (9288.42 to 26011.91) | 15514.96  (8692.54 to 24530.93) | -0.08  (-0.2 to 0.03) | 0.156 | 1221.44  (835.70 to 1671.97) | 1244.74  (854.85 to 1725.10) | 0.05  (0.03 to 0.08) | <0.001 |
|  | Trinidad and Tobago | 8809.07  (4866.65 to 14416.13) | 9052.26  (4983.02 to 14727.49) | 0.07  (0 to 0.15) | 0.067 | 2051.92  (1434.28 to 2773.56) | 2076.88  (1454.22 to 2802.93) | 0.04  (0.01 to 0.06) | 0.004 |
|  | Tunisia | 5891.81  (3301.16 to 9513.59) | 5717.09  (3171.13 to 9411.81) | -0.13  (-0.26 to 0) | 0.054 | 949.51  (640.18 to 1336.29) | 949.50  (636.34 to 1328.41) | 0  (-0.02 to 0.02) | 0.842 |
|  | Turkmenistan | 7439.75  (4120.20 to 12022.49) | 7640.07  (4213.15 to 12473.26) | 0.07  (0.02 to 0.13) | 0.011 | 783.03  (524.36 to 1088.10) | 785.28  (528.02 to 1094.57) | 0.01  (-0.01 to 0.02) | 0.233 |
|  | Tuvalu | 17576.32  (9755.13 to 28301.56) | 16106.67  (8980.43 to 26140.27) | -0.21  (-0.34 to -0.08) | 0.002 | 1223.85  (835.61 to 1687.20) | 1247.79  (858.35 to 1734.88) | 0.06  (0.03 to 0.09) | <0.001 |
|  | Türkiye | 2791.99  (1524.42 to 4554.30) | 2880.45  (1577.48 to 4725.21) | 0.2 (-1.04 to 1.44) | 0.756 | 841.03  (567.06 to 1179.12) | 820.93  (550.89 to 1151.52) | -0.08 (-0.14 to -0.01) | 0.016 |
|  | Uganda | 14969.68  (8573.65 to 23753.67) | 15492.31  (8866.10 to 24627.83) | 0.12  (-0.03 to 0.28) | 0.126 | 3129.23  (2643.51 to 3608.05) | 2529.33  (1837.65 to 3365.58) | -0.7  (-0.78 to -0.63) | <0.001 |
|  | Ukraine | 3844.66  (2029.23 to 6433.06) | 3847.43  (2059.80 to 6416.51) | 0.09  (0.03 to 0.14) | 0.002 | 1123.67  (770.60 to 1525.16) | 1126.08  (770.53 to 1548.14) | 0  (-0.01 to 0.01) | 0.849 |
|  | United Arab Emirates | 4085.76  (2227.87 to 6673.02) | 4064.04  (2232.55 to 6693.54) | -0.01  (-0.16 to 0.13) | 0.87 | 949.51  (640.18 to 1336.29) | 949.50  (636.34 to 1328.41) | 0  (-0.02 to 0.02) | 0.963 |
|  | United Kingdom | 2091.62  (1119.08 to 3474.29) | 2114.83  (1131.98 to 3504.28) | 0.05  (-0.11 to 0.21) | 0.558 | 708.31  (484.12 to 987.76) | 722.04  (488.52 to 1001.09) | 0.06  (0.05 to 0.07) | <0.001 |
|  | United Republic of Tanzania | 16691.79  (9601.31 to 26336.37) | 15436.95  (8742.93 to 24417.39) | -0.25  (-0.39 to -0.11) | 0.001 | 2026.70  (1444.94 to 2730.57) | 2061.08  (1458.00 to 2753.86) | 0.04  (-0.13 to 0.21) | 0.639 |
|  | United States of America | 9181.04  (5003.03 to 14987.00) | 8777.52  (4747.25 to 14484.22) | -0.14  (-0.38 to 0.11) | 0.28 | 1373.19  (951.83 to 1883.25) | 1232.68  (845.77 to 1700.90) | -0.33  (-0.4 to -0.27) | <0.001 |
|  | United States Virgin Islands | 8939.11  (4936.03 to 14619.47) | 9064.84  (4975.10 to 14805.38) | 0.04  (-0.01 to 0.1) | 0.095 | 2051.92  (1434.28 to 2773.56) | 2071.15  (1451.44 to 2800.49) | 0.02  (-0.02 to 0.05) | 0.392 |
|  | Uruguay | 3998.83  (2183.55 to 6512.44) | 4115.79  (2259.87 to 6843.87) | 0.11  (-0.02 to 0.23) | 0.091 | 1862.64  (1295.24 to 2526.08) | 1869.26  (1299.45 to 2534.79) | 0.02  (0 to 0.04) | 0.063 |
|  | Uzbekistan | 7481.49  (4158.40 to 12271.31) | 7580.31  (4199.42 to 12294.84) | 0.04  (0.02 to 0.05) | <0.001 | 780.40  (524.74 to 1083.31) | 786.12  (531.49 to 1093.86) | 0.02  (0.01 to 0.04) | <0.001 |
|  | Vanuatu | 16198.39  (9275.19 to 25472.52) | 15397.71  (8819.74 to 24610.51) | -0.08  (-0.23 to 0.07) | 0.278 | 997.88  (674.67 to 1373.55) | 1026.59  (694.78 to 1416.90) | 0.07  (0.04 to 0.1) | <0.001 |
|  | Venezuela (Bolivarian Republic of) | 13487.25  (7545.48 to 21809.47) | 13406.43  (7524.04 to 21377.34) | -0.07  (-0.23 to 0.08) | 0.358 | 2141.26  (1502.98 to 2904.21) | 2150.65  (1493.96 to 2922.93) | 0.02  (0.01 to 0.04) | 0.009 |
|  | Viet Nam | 4633.85  (2562.74 to 7602.05) | 4451.01  (2443.53 to 7331.12) | -0.02  (-0.22 to 0.18) | 0.824 | 1157.77  (824.93 to 1560.89) | 1182.84  (811.14 to 1631.01) | 0.07  (0.06 to 0.08) | <0.001 |
|  | Yemen | 4058.31  (2221.23 to 6642.88) | 4067.79  (2237.82 to 6697.97) | 0  (-0.22 to 0.23) | 0.967 | 949.51  (640.18 to 1336.29) | 946.96  (635.61 to 1335.72) | -0.01  (-0.02 to 0) | 0.099 |
|  | Zambia | 20743.98  (12048.36 to 31884.96) | 20886.19  (11922.10 to 32751.77) | 0.02  (-0.02 to 0.06) | 0.244 | 2363.35  (1672.87 to 3168.78) | 2420.70  (1750.29 to 3227.57) | 0.07  (0 to 0.14) | 0.051 |
|  | Zimbabwe | 22121.50  (12814.24 to 34052.77) | 22426.64  (13111.81 to 34757.76) | 0.04  (-0.12 to 0.19) | 0.654 | 2573.82  (1853.06 to 3379.65) | 2724.43  (1990.98 to 3572.75) | 0.17  (0.13 to 0.21) | <0.001 |
| Age-standardized Prevalence | Afghanistan | 3722.90  (1940.33 to 6355.72) | 3522.73  (1802.68 to 6001.50) | -0.18  (-0.19 to -0.16) | <0.001 | 13874.24  (10555.02 to 17636.75) | 13847.84  (10712.53 to 17364.62) | -0.01  (-0.03 to 0.01) | 0.199 |
|  | Albania | 4211.14  (2173.04 to 7237.41) | 3981.47  (2048.62 to 6807.81) | -0.18  (-0.31 to -0.05) | 0.006 | 7389.00  (5621.45 to 9460.39) | 7407.12  (5609.20 to 9544.85) | 0  (-0.01 to 0.02) | 0.558 |
|  | Algeria | 3584.41  (1863.82 to 6118.92) | 3339.04  (1739.36 to 5686.44) | -0.23  (-0.26 to -0.2) | <0.001 | 13876.16  (10622.45 to 17578.87) | 13842.53  (10594.64 to 17593.09) | -0.01  (-0.03 to 0.01) | 0.229 |
|  | American Samoa | 14399.89  (7831.83 to 23382.06) | 13796.37  (7399.36 to 22298.27) | -0.14  (-0.17 to -0.11) | <0.001 | 18065.24  (14009.11 to 22488.37) | 18397.71  (14362.05 to 22972.74) | 0.05  (0.01 to 0.09) | 0.012 |
|  | Andorra | 1557.30  (796.27 to 2696.26) | 1502.91  (760.35 to 2592.02) | -0.11  (-0.19 to -0.04) | 0.004 | 11420.45  (8677.93 to 14488.37) | 11361.18  (8727.40 to 14466.12) | -0.02  (-0.03 to 0) | 0.016 |
|  | Angola | 7738.18  (4114.33 to 12961.30) | 7261.33  (3828.95 to 12183.39) | -0.19  (-0.23 to -0.15) | <0.001 | 56695.76  (47211.33 to 66352.10) | 56880.44  (47543.72 to 66836.48) | 0  (-0.06 to 0.05) | 0.941 |
|  | Antigua and Barbuda | 7281.43  (3895.47 to 12205.29) | 7182.05  (3743.46 to 11943.98) | -0.06  (-0.11 to -0.01) | 0.015 | 31240.60  (24677.78 to 38408.49) | 31688.22  (24984.24 to 38912.61) | 0.04  (0.01 to 0.08) | 0.007 |
|  | Argentina | 3287.24  (1714.42 to 5635.33) | 3134.39  (1602.88 to 5381.45) | -0.15  (-0.18 to -0.13) | <0.001 | 31609.45  (30034.92 to 33201.85) | 28818.35  (22731.26 to 35717.27) | -0.31  (-0.35 to -0.28) | <0.001 |
|  | Armenia | 6433.34  (3412.18 to 10888.92) | 6151.42  (3218.48 to 10413.47) | -0.14  (-0.17 to -0.12) | <0.001 | 10013.74  (7593.98 to 12748.57) | 10082.27  (7632.18 to 12944.72) | 0.02  (0.01 to 0.03) | <0.001 |
|  | Australia | 2743.54  (1420.88 to 4756.85) | 2656.14  (1341.76 to 4608.37) | -0.12  (-0.14 to -0.09) | <0.001 | 21128.92  (16126.29 to 26665.51) | 15002.78  (11608.52 to 18839.91) | -0.94  (-1.25 to -0.64) | <0.001 |
|  | Austria | 1563.88  (803.19 to 2704.76) | 1516.84  (760.15 to 2628.31) | -0.1  (-0.14 to -0.05) | <0.001 | 9232.57  (7056.86 to 11787.28) | 9309.96  (7061.99 to 11877.45) | 0.03  (-0.01 to 0.06) | 0.105 |
|  | Azerbaijan | 6554.71  (3492.95 to 10989.51) | 6239.37  (3276.93 to 10451.19) | -0.16  (-0.18 to -0.13) | <0.001 | 10020.80  (7627.31 to 12808.71) | 10079.57  (7664.46 to 12929.02) | 0.02  (0.01 to 0.03) | 0.005 |
|  | Bahamas | 7418.87  (3914.88 to 12526.26) | 7252.80  (3810.17 to 12145.21) | -0.11  (-0.24 to 0.01) | 0.075 | 31209.98  (24513.35 to 38501.69) | 31823.50  (24948.91 to 38925.28) | 0.05  (0.05 to 0.06) | <0.001 |
|  | Bahrain | 3489.41  (1789.31 to 5975.17) | 3297.10  (1698.44 to 5623.31) | -0.19  (-0.21 to -0.16) | <0.001 | 13874.24  (10555.02 to 17636.75) | 13861.43  (10729.79 to 17358.82) | -0.01  (-0.02 to 0) | 0.181 |
|  | Bangladesh | 3485.49  (1812.75 to 5948.38) | 3298.31  (1689.68 to 5663.44) | -0.18  (-0.21 to -0.14) | <0.001 | 8092.50  (6124.51 to 10320.60) | 8081.50  (6114.42 to 10362.39) | 0  (-0.01 to 0) | <0.001 |
|  | Barbados | 7326.64  (3933.38 to 12242.28) | 7191.45  (3747.83 to 12078.35) | -0.07  (-0.12 to -0.03) | <0.001 | 31136.49  (24559.54 to 38460.33) | 31704.83  (24990.92 to 38971.99) | 0.05  (0.03 to 0.06) | <0.001 |
|  | Belarus | 3017.94  (1577.23 to 5216.65) | 2986.60  (1519.78 to 5102.87) | -0.04  (-0.22 to 0.13) | 0.642 | 17797.64  (13833.12 to 22261.49) | 17901.14  (13928.26 to 22348.53) | 0.02  (0.02 to 0.02) | <0.001 |
|  | Belgium | 937.13  (477.92 to 1625.32) | 880.48  (440.16 to 1537.68) | -0.23  (-0.37 to -0.08) | 0.002 | 11675.81  (8957.57 to 14706.09) | 11684.48  (8911.39 to 14896.59) | 0.01  (-0.02 to 0.04) | 0.437 |
|  | Belize | 7421.94  (3948.50 to 12506.06) | 7256.34  (3780.21 to 12217.95) | -0.09  (-0.15 to -0.02) | 0.014 | 31132.53  (24523.48 to 38317.14) | 31676.65  (25178.43 to 39113.78) | 0.04  (0.02 to 0.06) | 0.001 |
|  | Benin | 7754.50  (4054.00 to 13159.22) | 7240.27  (3794.32 to 12209.75) | -0.17  (-0.29 to -0.04) | 0.009 | 30056.28  (23812.57 to 37146.81) | 30207.90  (23738.14 to 37304.85) | 0.02  (0.01 to 0.02) | <0.001 |
|  | Bermuda | 7250.45  (3829.92 to 12116.41) | 7057.70  (3611.74 to 11964.58) | -0.12  (-0.21 to -0.03) | 0.01 | 31132.53  (24523.48 to 38317.14) | 31651.12  (24954.31 to 38893.30) | 0.04  (0.03 to 0.05) | <0.001 |
|  | Bhutan | 3458.92  (1797.35 to 5948.07) | 3227.41  (1651.63 to 5561.55) | -0.21  (-0.27 to -0.15) | <0.001 | 8108.03  (6107.78 to 10401.75) | 8081.78  (6127.02 to 10359.33) | -0.01  (-0.02 to 0) | 0.001 |
|  | Bolivia (Plurinational State of) | 5514.82  (2895.34 to 9346.50) | 5262.96  (2727.77 to 8823.52) | -0.16  (-0.19 to -0.12) | <0.001 | 37088.87  (29294.04 to 45057.26) | 37147.32  (29552.46 to 45236.25) | 0.01  (0 to 0.02) | 0.184 |
|  | Bosnia and Herzegovina | 4137.90  (2177.54 to 7095.90) | 3984.07  (2060.76 to 6813.40) | -0.1  (-0.21 to 0.01) | 0.063 | 7389.00  (5621.45 to 9460.39) | 7407.12  (5609.20 to 9544.85) | 0  (-0.01 to 0.02) | 0.558 |
|  | Botswana | 20232.43  (13534.87 to 28061.84) | 16592.05  (9024.03 to 26438.18) | -0.68  (-0.78 to -0.58) | <0.001 | 42473.34  (34313.79 to 51122.70) | 45144.48  (36641.18 to 54070.14) | 0.19  (0.15 to 0.22) | <0.001 |
|  | Brazil | 8219.46  (4260.54 to 13765.05) | 8060.38  (4162.36 to 13619.96) | -0.06  (-0.08 to -0.03) | <0.001 | 33738.58  (27076.42 to 40897.35) | 31198.44  (24693.13 to 38266.69) | -0.26  (-0.3 to -0.23) | <0.001 |
|  | Brunei Darussalam | 3490.64  (1825.88 to 5916.93) | 3313.70  (1711.18 to 5746.50) | -0.17  (-0.23 to -0.11) | <0.001 | 13524.14  (10407.47 to 16980.02) | 13552.30  (10459.69 to 17117.88) | 0.01  (0 to 0.03) | 0.052 |
|  | Bulgaria | 4079.68  (2119.52 to 6959.54) | 3935.36  (2032.13 to 6630.06) | -0.12  (-0.2 to -0.03) | 0.009 | 10200.31  (7716.00 to 13250.00) | 10215.45  (7731.72 to 13130.10) | 0  (-0.08 to 0.09) | 0.966 |
|  | Burkina Faso | 26696.29  (19632.29 to 33613.42) | 17613.75  (9805.58 to 28155.99) | -1.34  (-1.43 to -1.25) | <0.001 | 29680.12  (23176.12 to 36795.46) | 29339.11  (23043.86 to 36171.97) | -0.05  (-0.09 to -0.02) | 0.005 |
|  | Burundi | 15578.06  (8499.29 to 25174.18) | 14947.89  (8100.74 to 24397.88) | -0.13  (-0.15 to -0.11) | <0.001 | 38223.48  (30518.04 to 46683.94) | 37965.91  (30428.74 to 46188.00) | -0.03  (-0.04 to -0.01) | <0.001 |
|  | Cabo Verde | 12588.65  (6830.35 to 20500.62) | 11978.82  (6495.01 to 19700.44) | -0.18  (-0.21 to -0.15) | <0.001 | 32094.46  (25246.33 to 39572.77) | 32131.54  (25274.29 to 39352.18) | 0  (-0.01 to 0.01) | 0.796 |
|  | Cambodia | 5219.56  (2718.65 to 8867.44) | 4837.34  (2509.70 to 8284.89) | -0.25  (-0.26 to -0.23) | <0.001 | 18831.22  (14686.73 to 23615.74) | 18846.16  (14725.51 to 23476.37) | 0  (-0.02 to 0.01) | 0.955 |
|  | Cameroon | 15748.41  (8681.99 to 25480.12) | 15371.63  (8375.17 to 24886.05) | -0.09  (-0.16 to -0.01) | 0.029 | 39101.99  (31338.84 to 47281.11) | 39699.40  (31944.56 to 47975.20) | -0.05  (-0.16 to 0.06) | 0.345 |
|  | Canada | 6604.23  (3459.44 to 11103.88) | 6381.07  (3321.93 to 10704.20) | -0.12  (-0.13 to -0.1) | <0.001 | 11045.99  (8527.77 to 13954.80) | 10995.42  (8424.05 to 13850.86) | 0.01  (-0.2 to 0.21) | 0.956 |
|  | Central African Republic | 7717.53  (4136.91 to 12895.50) | 7414.89  (3926.18 to 12378.89) | -0.11  (-0.16 to -0.07) | <0.001 | 57033.24  (47500.10 to 66331.46) | 56813.27  (47443.50 to 66651.94) | -0.05  (-0.12 to 0.03) | 0.22 |
|  | Chad | 13071.47  (7066.98 to 21458.63) | 12440.59  (6740.14 to 20439.91) | -0.17  (-0.22 to -0.13) | <0.001 | 25533.91  (19879.71 to 31709.84) | 25719.26  (20165.54 to 31984.13) | 0.03  (-0.01 to 0.07) | 0.09 |
|  | Chile | 3275.83  (1717.73 to 5603.07) | 3107.21  (1606.92 to 5320.09) | -0.17  (-0.21 to -0.13) | <0.001 | 28716.55  (22662.48 to 35396.08) | 28715.61  (22535.91 to 35319.47) | 0.01  (0 to 0.02) | 0.116 |
|  | China | 3910.79  (1955.30 to 6728.75) | 3705.21  (1857.23 to 6454.02) | -0.17  (-0.3 to -0.03) | 0.016 | 11585.16  (8900.63 to 14792.27) | 11604.80  (8884.22 to 14750.58) | -0.02  (-0.14 to 0.11) | 0.786 |
|  | Colombia | 11513.61  (6220.16 to 18825.92) | 11096.94  (5919.53 to 18209.78) | -0.11  (-0.15 to -0.08) | <0.001 | 45265.07  (40428.27 to 50198.42) | 36211.85  (28651.51 to 44169.14) | -0.75  (-0.87 to -0.62) | <0.001 |
|  | Comoros | 15377.02  (8424.54 to 24876.74) | 14860.80  (8058.62 to 24240.00) | -0.12  (-0.15 to -0.09) | <0.001 | 37463.91  (29992.09 to 45617.00) | 37761.17  (30321.35 to 45690.85) | 0.02  (0.01 to 0.04) | <0.001 |
|  | Congo | 7603.72  (4048.40 to 12637.02) | 7196.47  (3763.48 to 12076.29) | -0.17  (-0.21 to -0.12) | <0.001 | 57111.23  (47807.16 to 66176.26) | 56945.07  (47525.70 to 66073.91) | -0.02  (-0.08 to 0.05) | 0.596 |
|  | Cook Islands | 13295.81  (7016.52 to 21992.33) | 13189.80  (6922.09 to 21662.08) | -0.03  (-0.04 to -0.02) | <0.001 | 18132.52  (13959.60 to 22737.28) | 18463.40  (14415.97 to 23178.61) | 0.05  (0.04 to 0.06) | <0.001 |
|  | Costa Rica | 11325.69  (6158.17 to 18537.55) | 11032.54  (5941.99 to 18144.15) | -0.08  (-0.1 to -0.05) | <0.001 | 35882.25  (32566.36 to 39279.79) | 32355.00  (25563.63 to 39852.31) | -0.36  (-0.41 to -0.31) | <0.001 |
|  | Coted'Ivoire | 10901.45  (5786.59 to 18086.58) | 10378.55  (5457.82 to 17456.06) | -0.16  (-0.24 to -0.09) | <0.001 | 32561.52  (25633.99 to 39637.97) | 32491.99  (25864.44 to 39924.65) | -0.01  (-0.02 to 0) | 0.016 |
|  | Croatia | 4039.54  (2104.89 to 6943.93) | 3912.41  (2025.06 to 6680.19) | -0.08  (-0.26 to 0.09) | 0.351 | 8282.08  (6284.46 to 10607.49) | 8223.75  (6176.70 to 10519.67) | -0.03  (-0.08 to 0.02) | 0.179 |
|  | Cuba | 7229.73  (3821.52 to 12061.67) | 7078.66  (3724.95 to 11848.17) | -0.11  (-0.22 to 0) | 0.061 | 31117.95  (24624.14 to 38019.84) | 31637.26  (25058.96 to 38776.55) | 0.04  (0.03 to 0.06) | <0.001 |
|  | Cyprus | 1589.29  (803.79 to 2789.58) | 1518.31  (773.98 to 2654.67) | -0.15  (-0.22 to -0.09) | <0.001 | 11392.02  (8816.34 to 14462.15) | 11380.99  (8723.44 to 14456.40) | 0  (-0.01 to 0) | 0.731 |
|  | Czechia | 4046.94  (2108.39 to 6849.18) | 3895.81  (2021.73 to 6711.79) | -0.1  (-0.28 to 0.08) | 0.267 | 6485.83  (6051.51 to 6905.90) | 6465.20  (4887.35 to 8269.69) | -0.01  (-0.02 to 0) | 0.174 |
|  | Democratic People's Republic of Korea | 3821.54  (1998.65 to 6482.52) | 3659.08  (1891.90 to 6325.00) | -0.14  (-0.16 to -0.13) | <0.001 | 12144.79  (9295.07 to 15502.04) | 12101.56  (9315.42 to 15463.97) | -0.01  (-0.02 to 0) | 0.032 |
|  | Democratic Republic of the Congo | 7731.72  (4094.12 to 12896.05) | 7297.79  (3834.83 to 12252.67) | -0.17  (-0.23 to -0.11) | <0.001 | 56970.58  (47780.87 to 66756.35) | 56648.52  (47439.66 to 66174.86) | -0.04  (-0.06 to -0.01) | 0.002 |
|  | Denmark | 1564.42  (799.26 to 2685.20) | 1510.08  (765.39 to 2634.96) | -0.11  (-0.22 to -0.01) | 0.03 | 16775.55  (12581.58 to 22265.07) | 13871.76  (10601.55 to 17508.31) | -0.62  (-0.69 to -0.55) | <0.001 |
|  | Djibouti | 15567.15  (8559.93 to 25217.73) | 14915.02  (8110.16 to 24123.25) | -0.13  (-0.16 to -0.11) | <0.001 | 35638.54  (28338.12 to 43238.72) | 36001.42  (28592.71 to 44064.12) | 0.02  (-0.06 to 0.11) | 0.596 |
|  | Dominica | 7373.08  (3925.94 to 12420.44) | 7201.55  (3757.27 to 12235.05) | -0.12  (-0.24 to 0.01) | 0.069 | 31240.60  (24677.78 to 38408.49) | 31651.12  (24954.31 to 38893.30) | 0.04  (0.02 to 0.06) | <0.001 |
|  | Dominican Republic | 7390.57  (3920.55 to 12526.57) | 7218.50  (3837.37 to 12018.07) | -0.08  (-0.16 to -0.01) | 0.021 | 31187.10  (24266.03 to 38484.16) | 31694.80  (24713.61 to 38987.35) | 0.04  (0 to 0.07) | 0.07 |
|  | Ecuador | 5363.78  (2846.80 to 9014.70) | 5208.06  (2682.63 to 8839.23) | -0.1  (-0.17 to -0.03) | 0.004 | 36997.22  (29305.89 to 44821.44) | 37156.04  (29542.63 to 45231.99) | 0.02  (0.01 to 0.04) | 0.002 |
|  | Egypt | 5194.75  (2727.54 to 8889.75) | 4910.01  (2547.37 to 8429.47) | -0.23  (-0.38 to -0.08) | 0.002 | 13876.16  (10622.45 to 17578.87) | 13861.43  (10729.79 to 17358.82) | -0.01  (-0.02 to 0) | 0.194 |
|  | El Salvador | 11575.09  (6231.63 to 18999.82) | 11128.67  (5952.00 to 18145.35) | -0.12  (-0.15 to -0.09) | <0.001 | 33186.42  (26135.87 to 40839.98) | 33127.20  (26298.14 to 40512.65) | 0  (-0.01 to 0) | <0.001 |
|  | Equatorial Guinea | 7791.30  (4151.12 to 12957.42) | 7188.38  (3773.63 to 12117.82) | -0.26  (-0.31 to -0.22) | <0.001 | 56744.64  (47810.89 to 65996.91) | 57776.05  (48415.58 to 67132.42) | 0.04  (-0.02 to 0.1) | 0.205 |
|  | Eritrea | 15579.62  (8511.38 to 25117.37) | 14912.69  (8094.03 to 24368.53) | -0.15  (-0.19 to -0.12) | <0.001 | 28858.21  (23391.29 to 34922.43) | 30439.51  (24120.76 to 37522.00) | 0.16  (0.13 to 0.2) | <0.001 |
|  | Estonia | 3034.13  (1580.28 to 5220.27) | 2978.54  (1543.93 to 5124.67) | -0.04  (-0.29 to 0.22) | 0.782 | 17813.32  (13833.85 to 22349.35) | 17896.97  (13910.56 to 22509.12) | -0.03  (-0.06 to 0.01) | 0.126 |
|  | Eswatini | 19166.82  (10620.31 to 30137.52) | 18652.80  (10312.97 to 29327.53) | -0.1  (-0.13 to -0.07) | <0.001 | 42282.21  (34346.38 to 50695.51) | 47579.18  (38675.60 to 56677.14) | 0.39  (0.37 to 0.4) | <0.001 |
|  | Ethiopia | 15964.82  (8407.41 to 26225.66) | 15272.68  (7982.97 to 25324.34) | -0.15  (-0.18 to -0.12) | <0.001 | 26225.46  (20664.65 to 32594.54) | 26434.07  (20721.00 to 32788.45) | -0.04  (-0.22 to 0.14) | 0.652 |
|  | Fiji | 11201.46  (7537.45 to 15751.70) | 11468.18  (6106.74 to 18836.26) | 0.07  (0.02 to 0.12) | 0.004 | 18065.24  (14009.11 to 22488.37) | 18398.33  (14225.52 to 23120.83) | 0.05  (0.02 to 0.08) | <0.001 |
|  | Finland | 1567.00  (807.97 to 2724.98) | 1508.23  (768.18 to 2611.06) | -0.12  (-0.18 to -0.07) | <0.001 | 17593.32  (15208.28 to 20018.09) | 11325.19  (8744.19 to 14377.45) | -1.41  (-1.49 to -1.33) | <0.001 |
|  | France | 2302.54  (1162.64 to 3998.44) | 2163.12  (1087.59 to 3749.99) | -0.2  (-0.23 to -0.16) | <0.001 | 11151.94  (8433.23 to 14183.79) | 11143.02  (8527.12 to 14118.31) | -0.01  (-0.03 to 0.02) | 0.593 |
|  | Gabon | 7599.64  (3987.49 to 12673.68) | 7197.96  (3775.08 to 12243.15) | -0.15  (-0.17 to -0.14) | <0.001 | 56997.45  (47649.55 to 66781.47) | 57269.63  (47928.61 to 66989.08) | -0.05  (-0.1 to 0.01) | 0.087 |
|  | Gambia | 12670.43  (6837.17 to 20634.72) | 12149.16  (6530.52 to 19824.36) | -0.13  (-0.17 to -0.1) | <0.001 | 30394.29  (23785.04 to 37633.88) | 30798.91  (24056.63 to 38096.25) | 0.02  (0.01 to 0.04) | 0.004 |
|  | Georgia | 6399.91  (3376.39 to 10835.02) | 6136.35  (3234.17 to 10283.86) | -0.14  (-0.15 to -0.12) | <0.001 | 10013.74  (7593.98 to 12748.57) | 10078.45  (7629.08 to 12790.46) | 0.02  (0.02 to 0.03) | <0.001 |
|  | Germany | 1566.62  (812.86 to 2701.82) | 1511.66  (758.33 to 2618.71) | -0.13  (-0.17 to -0.09) | <0.001 | 17554.97  (13324.82 to 22588.63) | 13857.89  (10635.96 to 17562.62) | -0.69  (-0.98 to -0.39) | <0.001 |
|  | Ghana | 12635.00  (6879.13 to 20792.88) | 12081.28  (6503.03 to 19932.92) | -0.15  (-0.18 to -0.11) | <0.001 | 32078.36  (25262.48 to 39362.72) | 32421.33  (25571.39 to 40055.26) | 0.03  (0.03 to 0.04) | <0.001 |
|  | Greece | 2476.61  (1279.55 to 4296.13) | 2485.86  (1266.46 to 4321.94) | 0.02  (-0.05 to 0.09) | 0.504 | 9132.59  (6962.57 to 11606.56) | 9112.40  (6955.22 to 11624.03) | -0.01  (-0.03 to 0.01) | 0.422 |
|  | Greenland | 6779.74  (3555.31 to 11541.14) | 6551.85  (3420.51 to 10949.96) | -0.11  (-0.13 to -0.08) | <0.001 | 14182.97  (10833.43 to 17917.92) | 14173.49  (10899.85 to 17805.50) | -0.01  (-0.01 to 0) | 0.219 |
|  | Grenada | 7400.67  (3955.26 to 12344.62) | 7244.39  (3785.32 to 12120.54) | -0.11  (-0.23 to 0.02) | 0.088 | 31199.30  (24598.51 to 38291.31) | 31542.28  (24887.24 to 38757.13) | 0.03  (0.01 to 0.05) | 0.001 |
|  | Guam | 14017.76  (7635.67 to 22981.32) | 13523.92  (7285.45 to 22151.26) | -0.11  (-0.15 to -0.08) | <0.001 | 18091.27  (14005.11 to 22618.85) | 18356.68  (14225.29 to 22883.47) | 0.04  (0.03 to 0.06) | <0.001 |
|  | Guatemala | 11696.41  (6360.37 to 19134.50) | 11296.62  (6073.16 to 18539.00) | -0.11  (-0.18 to -0.04) | 0.001 | 33215.25  (26361.24 to 40870.25) | 33211.42  (26234.53 to 40513.53) | 0  (0 to 0) | 0.862 |
|  | Guinea | 12937.53  (6925.42 to 21208.58) | 12426.12  (6690.64 to 20335.98) | -0.13  (-0.14 to -0.12) | <0.001 | 31930.20  (25075.13 to 39222.71) | 32391.92  (25566.51 to 39909.37) | 0.04  (0.04 to 0.05) | <0.001 |
|  | Guinea-Bissau | 12833.27  (6945.17 to 20857.61) | 12309.17  (6630.15 to 20221.12) | -0.15  (-0.2 to -0.1) | <0.001 | 31999.10  (25123.82 to 39379.08) | 32597.19  (25837.22 to 40147.73) | 0.06  (0.04 to 0.07) | <0.001 |
|  | Guyana | 7449.54  (3993.71 to 12423.25) | 7309.28  (3857.53 to 12249.08) | -0.08  (-0.22 to 0.06) | 0.281 | 31136.49  (24559.54 to 38460.33) | 31885.34  (25150.66 to 39098.21) | 0.07  (0.06 to 0.09) | <0.001 |
|  | Haiti | 7772.59  (4166.73 to 12906.58) | 7487.55  (3920.41 to 12560.77) | -0.15  (-0.24 to -0.06) | 0.001 | 25324.10  (19767.89 to 31452.86) | 25223.79  (19614.42 to 31385.11) | -0.04  (-0.08 to -0.01) | 0.013 |
|  | Honduras | 11753.59  (6314.28 to 19288.38) | 11392.29  (6057.11 to 18643.78) | -0.1  (-0.2 to 0) | 0.049 | 39950.16  (32252.25 to 48555.91) | 40070.07  (32133.35 to 48543.05) | -0.04  (-0.08 to 0) | 0.054 |
|  | Hungary | 4082.16  (2116.77 to 6987.73) | 3908.00  (2035.20 to 6647.20) | -0.13  (-0.22 to -0.05) | 0.002 | 5693.32  (4296.54 to 7334.50) | 5704.10  (4327.12 to 7373.03) | 0.02  (-0.01 to 0.04) | 0.162 |
|  | Iceland | 1562.23  (801.59 to 2715.74) | 1496.73  (762.01 to 2574.42) | -0.16  (-0.21 to -0.1) | <0.001 | 13165.28  (10014.95 to 16796.52) | 13181.14  (10105.06 to 16683.52) | -0.01  (-0.19 to 0.16) | 0.877 |
|  | India | 3427.15  (1725.54 to 5930.76) | 3333.04  (1673.74 to 5752.56) | -0.07  (-0.25 to 0.12) | 0.474 | 7084.25  (5360.46 to 9069.66) | 7969.88  (6038.77 to 10136.74) | 0.39  (0.32 to 0.45) | <0.001 |
|  | Indonesia | 5204.77  (2667.25 to 8940.95) | 4928.60  (2518.38 to 8346.37) | -0.15  (-0.2 to -0.09) | <0.001 | 14652.75  (11259.98 to 18465.20) | 14723.52  (11368.88 to 18516.32) | 0.02  (0 to 0.04) | 0.012 |
|  | Iran (Islamic Republic of) | 3202.43  (1613.88 to 5532.83) | 3078.48  (1531.50 to 5337.88) | 0.2  (-0.46 to 0.86) | 0.556 | 15853.78  (12212.20 to 19980.95) | 15879.94  (12306.23 to 19928.75) | 0.01  (-0.01 to 0.03) | 0.324 |
|  | Iraq | 8442.37  (4788.64 to 13273.05) | 5714.59  (2978.00 to 9716.17) | -1.19  (-1.45 to -0.94) | <0.001 | 13876.16  (10622.45 to 17578.87) | 13847.84  (10712.53 to 17364.62) | -0.01  (-0.02 to 0) | 0.294 |
|  | Ireland | 1577.93  (801.32 to 2757.72) | 1509.65  (757.72 to 2584.86) | -0.16  (-0.25 to -0.07) | <0.001 | 11420.45  (8677.93 to 14488.37) | 11361.92  (8692.90 to 14537.14) | -0.02  (-0.03 to -0.01) | <0.001 |
|  | Israel | 1580.74  (807.43 to 2734.53) | 1532.37  (774.09 to 2629.40) | -0.1  (-0.18 to -0.02) | 0.016 | 11457.75  (8867.25 to 14287.28) | 10866.38  (8216.95 to 13861.53) | -0.19  (-0.35 to -0.03) | 0.021 |
|  | Italy | 1407.57  (709.95 to 2435.66) | 1352.04  (681.43 to 2321.43) | -0.14  (-0.17 to -0.1) | <0.001 | 8907.90  (6783.28 to 11279.44) | 8879.23  (6751.18 to 11266.78) | -0.01  (-0.02 to 0.01) | 0.401 |
|  | Jamaica | 7356.33  (3924.38 to 12421.43) | 7221.79  (3799.12 to 12007.80) | -0.07  (-0.16 to 0.03) | 0.164 | 47702.79  (38304.47 to 54372.35) | 38984.32  (31438.75 to 47309.72) | -0.66  (-0.79 to -0.53) | <0.001 |
|  | Japan | 3425.48  (1718.42 to 5922.55) | 3327.29  (1680.25 to 5717.65) | -0.1  (-0.14 to -0.05) | <0.001 | 8614.00  (6495.06 to 11111.32) | 8881.27  (6689.73 to 11449.04) | 0.1  (0.04 to 0.15) | 0.001 |
|  | Jordan | 2117.30  (1095.18 to 3616.49) | 1970.91  (992.02 to 3376.11) | -0.24  (-0.32 to -0.15) | <0.001 | 14842.82  (11285.60 to 18837.19) | 14782.79  (11333.32 to 18694.79) | -0.02  (-0.06 to 0.01) | 0.19 |
|  | Kazakhstan | 6372.50  (3343.06 to 10756.60) | 6158.36  (3227.27 to 10310.08) | -0.11  (-0.16 to -0.05) | <0.001 | 10020.80  (7627.31 to 12808.71) | 10072.48  (7606.78 to 12859.43) | 0.02  (0.01 to 0.02) | <0.001 |
|  | Kenya | 11190.06  (5896.84 to 18618.28) | 14165.95  (7397.48 to 23288.82) | 0.74  (0.53 to 0.95) | <0.001 | 37439.21  (29901.66 to 45347.70) | 37553.10  (30019.45 to 45416.94) | 0  (-0.08 to 0.07) | 0.92 |
|  | Kiribati | 14702.44  (8103.39 to 23660.91) | 14198.58  (7620.79 to 22787.09) | -0.12  (-0.15 to -0.08) | <0.001 | 18048.40  (13974.68 to 22493.52) | 18386.71  (14236.89 to 23057.91) | 0.06  (0.04 to 0.09) | <0.001 |
|  | Kuwait | 2257.44  (1153.33 to 3871.06) | 2076.34  (1047.12 to 3563.19) | -0.31  (-0.41 to -0.21) | <0.001 | 13876.16  (10622.45 to 17578.87) | 13858.57  (10712.51 to 17382.50) | -0.01  (-0.02 to 0) | 0.139 |
|  | Kyrgyzstan | 6413.28  (3419.11 to 10812.90) | 6183.56  (3247.59 to 10374.54) | -0.11  (-0.13 to -0.09) | <0.001 | 10020.80  (7627.31 to 12808.71) | 10081.23  (7639.77 to 12868.17) | 0.02  (-0.01 to 0.04) | 0.16 |
|  | Lao People's Democratic Republic | 5230.23  (2714.19 to 8797.88) | 4880.51  (2547.52 to 8394.78) | -0.22  (-0.25 to -0.18) | <0.001 | 18831.22  (14686.73 to 23615.74) | 18838.60  (14550.21 to 23712.24) | 0  (0 to 0) | 0.98 |
|  | Latvia | 3051.26  (1590.47 to 5209.06) | 2966.73  (1522.37 to 5032.12) | -0.09  (-0.3 to 0.12) | 0.385 | 17857.39  (13925.33 to 22375.10) | 17901.14  (13928.26 to 22348.53) | 0.01  (0 to 0.01) | 0.001 |
|  | Lebanon | 2021.77  (1043.90 to 3506.00) | 1850.51  (943.81 to 3239.16) | -0.34  (-0.71 to 0.04) | 0.076 | 13874.24  (10555.02 to 17636.75) | 13880.10  (10600.59 to 17664.57) | 0  (-0.03 to 0.02) | 0.792 |
|  | Lesotho | 19198.26  (10718.83 to 30052.72) | 18710.56  (10383.41 to 29665.69) | -0.09  (-0.12 to -0.06) | <0.001 | 42555.29  (34310.81 to 51380.02) | 47682.81  (38667.82 to 56663.32) | 0.37  (0.36 to 0.38) | <0.001 |
|  | Liberia | 12718.49  (6836.98 to 20628.10) | 12178.65  (6584.96 to 20102.26) | -0.14  (-0.17 to -0.12) | <0.001 | 31997.13  (25212.17 to 39439.34) | 32190.34  (25452.68 to 39601.24) | 0.01  (0.01 to 0.02) | <0.001 |
|  | Libya | 2075.04  (1058.47 to 3562.49) | 1879.07  (958.57 to 3238.91) | -0.35  (-0.55 to -0.14) | 0.001 | 13876.16  (10622.45 to 17578.87) | 13847.84  (10712.53 to 17364.62) | -0.01  (-0.02 to 0.01) | 0.259 |
|  | Lithuania | 3003.33  (1558.09 to 5150.22) | 2971.47  (1512.23 to 5116.62) | -0.05  (-0.23 to 0.14) | 0.633 | 17797.64  (13833.12 to 22261.49) | 17902.58  (13864.47 to 22598.77) | 0.02  (0 to 0.03) | 0.013 |
|  | Luxembourg | 1552.97  (783.91 to 2695.41) | 1502.41  (764.28 to 2611.22) | -0.11  (-0.16 to -0.05) | <0.001 | 11406.39  (8714.89 to 14512.66) | 11394.37  (8725.51 to 14527.41) | -0.01  (-0.01 to 0) | <0.001 |
|  | Madagascar | 15334.39  (8425.83 to 24947.59) | 14907.02  (8193.08 to 24270.68) | -0.09  (-0.11 to -0.08) | <0.001 | 37463.91  (29992.09 to 45617.00) | 37751.29  (30242.00 to 45566.99) | 0.02  (0.01 to 0.03) | 0.003 |
|  | Malawi | 9374.34  (4938.98 to 15535.99) | 8955.71  (4689.80 to 15177.32) | -0.21  (-0.44 to 0.02) | 0.079 | 48614.55  (41764.57 to 54711.66) | 43814.36  (35318.88 to 52673.18) | -0.35  (-0.4 to -0.31) | <0.001 |
|  | Malaysia | 4966.71  (2614.97 to 8378.03) | 4708.70  (2442.59 to 7959.61) | -0.17  (-0.22 to -0.12) | <0.001 | 18813.12  (14702.63 to 23473.52) | 18822.47  (14761.25 to 23508.86) | 0  (0 to 0.01) | 0.662 |
|  | Maldives | 5013.67  (2657.27 to 8536.45) | 4685.61  (2417.56 to 7990.23) | -0.23  (-0.27 to -0.19) | <0.001 | 18833.69  (14712.15 to 23522.16) | 18817.74  (14673.66 to 23761.95) | 0  (-0.01 to 0) | 0.633 |
|  | Mali | 19117.46  (10706.91 to 30100.93) | 18855.23  (10575.73 to 29903.42) | -0.07  (-0.24 to 0.1) | 0.425 | 48611.32  (44288.24 to 52993.77) | 39044.26  (31715.34 to 47172.04) | -0.7  (-0.71 to -0.68) | <0.001 |
|  | Malta | 1579.81  (810.45 to 2768.77) | 1522.56  (775.13 to 2628.71) | -0.12  (-0.22 to -0.01) | 0.026 | 11420.45  (8677.93 to 14488.37) | 11361.92  (8692.90 to 14537.14) | -0.02  (-0.02 to -0.01) | <0.001 |
|  | Marshall Islands | 14372.71  (7798.58 to 23175.27) | 13839.93  (7455.23 to 22461.83) | -0.13  (-0.16 to -0.1) | <0.001 | 18132.52  (13959.60 to 22737.28) | 18409.89  (14325.87 to 23202.43) | 0.04  (0.03 to 0.06) | <0.001 |
|  | Mauritania | 12899.77  (6923.45 to 21128.86) | 12186.86  (6571.63 to 20042.30) | -0.19  (-0.23 to -0.15) | <0.001 | 31999.10  (25123.82 to 39379.08) | 32206.19  (25539.36 to 39543.99) | 0.02  (0.01 to 0.03) | <0.001 |
|  | Mauritius | 4912.00  (2577.51 to 8464.16) | 4703.37  (2463.53 to 7983.92) | -0.14  (-0.15 to -0.13) | <0.001 | 18833.69  (14712.15 to 23522.16) | 18822.47  (14761.25 to 23508.86) | 0  (-0.01 to 0.01) | 0.455 |
|  | Mexico | 11257.96  (5963.67 to 18484.62) | 11241.47  (5910.18 to 18416.24) | -0.02  (-0.08 to 0.03) | 0.366 | 23956.62  (18794.16 to 29778.31) | 23141.01  (17989.48 to 28679.29) | -0.11  (-0.33 to 0.11) | 0.328 |
|  | Micronesia (Federated States of) | 14687.58  (8000.22 to 23749.89) | 13968.67  (7576.13 to 22696.39) | -0.16  (-0.19 to -0.12) | <0.001 | 18132.52  (13959.60 to 22737.28) | 18417.30  (14368.93 to 23032.94) | 0.04  (0.02 to 0.07) | <0.001 |
|  | Monaco | 1436.74  (721.72 to 2495.28) | 1447.24  (720.82 to 2539.85) | 0.02  (0 to 0.04) | 0.014 | 11420.45  (8677.93 to 14488.37) | 11361.18  (8727.40 to 14466.12) | -0.02  (-0.02 to -0.01) | <0.001 |
|  | Mongolia | 6535.20  (3506.38 to 10755.09) | 6257.47  (3246.78 to 10639.04) | -0.14  (-0.18 to -0.11) | <0.001 | 10013.74  (7593.98 to 12748.57) | 10045.15  (7630.82 to 12765.97) | 0.01  (0 to 0.02) | 0.125 |
|  | Montenegro | 4099.69  (2128.51 to 7055.40) | 3971.14  (2023.51 to 6826.10) | -0.09  (-0.23 to 0.05) | 0.208 | 7389.00  (5621.45 to 9460.39) | 7421.27  (5617.06 to 9619.70) | 0.01  (-0.03 to 0.04) | 0.716 |
|  | Morocco | 4335.47  (2244.45 to 7472.29) | 4095.46  (2108.73 to 7031.62) | -0.13  (-0.42 to 0.16) | 0.374 | 25306.93  (23599.79 to 27039.22) | 16007.95  (12317.09 to 20330.96) | -1.45  (-1.51 to -1.39) | <0.001 |
|  | Mozambique | 27694.16  (16986.01 to 40312.41) | 20419.46  (11438.08 to 32289.67) | -1  (-1.03 to -0.96) | <0.001 | 44557.31  (35998.10 to 53374.39) | 46514.99  (38040.55 to 55090.61) | 0.15  (0.1 to 0.2) | <0.001 |
|  | Myanmar | 5188.29  (2699.43 to 8825.38) | 4845.57  (2512.09 to 8184.18) | -0.22  (-0.25 to -0.19) | <0.001 | 18765.05  (14604.36 to 23457.18) | 18817.88  (14660.06 to 23531.30) | 0.01  (-0.01 to 0.03) | 0.262 |
|  | Namibia | 19326.14  (10743.52 to 30692.50) | 18599.41  (10227.95 to 29564.14) | -0.13  (-0.13 to -0.12) | <0.001 | 42427.97  (34015.75 to 51302.11) | 45317.90  (36757.94 to 54460.82) | 0.21  (0.2 to 0.22) | <0.001 |
|  | Nauru | 13556.99  (7308.87 to 22316.08) | 13425.66  (7137.27 to 22131.68) | -0.02  (-0.04 to 0) | 0.015 | 18132.52  (13959.60 to 22737.28) | 18463.40  (14415.97 to 23178.61) | 0.05  (0.04 to 0.06) | <0.001 |
|  | Nepal | 3479.58  (1818.33 to 5976.40) | 3251.76  (1681.10 to 5616.17) | -0.21  (-0.24 to -0.18) | <0.001 | 8092.50  (6124.51 to 10320.60) | 8081.78  (6127.02 to 10359.33) | 0  (-0.01 to 0) | <0.001 |
|  | Netherlands | 960.55  (480.73 to 1660.83) | 905.19  (448.67 to 1587.35) | -0.19  (-0.49 to 0.11) | 0.216 | 14097.68  (10789.46 to 17934.19) | 14079.15  (10746.54 to 17741.71) | -0.01  (-0.13 to 0.12) | 0.9 |
|  | New Zealand | 2758.90  (1380.26 to 4888.56) | 2656.27  (1334.45 to 4706.35) | -0.14  (-0.23 to -0.06) | 0.001 | 9418.64  (7197.26 to 11986.23) | 9658.07  (7416.39 to 12240.96) | 0.08  (0.03 to 0.12) | 0.001 |
|  | Nicaragua | 11664.36  (6318.28 to 19056.45) | 11107.49  (5961.21 to 18417.30) | -0.15  (-0.24 to -0.06) | 0.001 | 33160.48  (26368.15 to 40798.43) | 33189.33  (26285.93 to 40760.17) | 0.01  (0 to 0.01) | 0.046 |
|  | Niger | 12959.72  (7098.51 to 21200.67) | 12480.12  (6728.45 to 20476.67) | -0.13  (-0.17 to -0.1) | <0.001 | 32003.06  (25198.94 to 39734.77) | 32040.94  (25326.33 to 39201.50) | 0  (0 to 0.01) | 0.454 |
|  | Nigeria | 10523.80  (5642.29 to 17144.59) | 11322.31  (6064.72 to 18403.96) | 0.23  (0.05 to 0.41) | 0.012 | 35483.78  (28351.31 to 43121.62) | 35064.00  (28000.11 to 42668.09) | -0.03  (-0.09 to 0.04) | 0.449 |
|  | Niue | 13303.70  (7033.20 to 21898.99) | 13276.33  (7028.07 to 21766.71) | -0.02  (-0.06 to 0.02) | 0.299 | 18132.52  (13959.60 to 22737.28) | 18463.40  (14415.97 to 23178.61) | 0.05  (0.04 to 0.06) | <0.001 |
|  | North Macedonia | 4089.77  (2142.90 to 6950.68) | 3962.29  (2052.45 to 6807.83) | -0.09  (-0.21 to 0.04) | 0.172 | 7389.00  (5621.45 to 9460.39) | 7407.12  (5609.20 to 9544.85) | 0  (-0.01 to 0.02) | 0.558 |
|  | Northern Mariana Islands | 13907.85  (7560.93 to 22792.64) | 13447.36  (7152.50 to 21660.84) | -0.11  (-0.13 to -0.09) | <0.001 | 18065.24  (14009.11 to 22488.37) | 18387.57  (14387.45 to 22932.45) | 0.05  (0.03 to 0.08) | <0.001 |
|  | Norway | 1520.35  (756.39 to 2639.78) | 1471.33  (730.16 to 2529.11) | -0.11  (-0.11 to -0.1) | <0.001 | 13907.12  (10690.59 to 17655.75) | 12084.22  (9261.62 to 15338.40) | -0.46  (-0.48 to -0.44) | <0.001 |
|  | Oman | 3489.12  (1785.33 to 5964.62) | 3287.51  (1685.39 to 5708.42) | -0.19  (-0.21 to -0.17) | <0.001 | 13874.24  (10555.02 to 17636.75) | 13842.53  (10594.64 to 17593.09) | -0.01  (-0.02 to 0) | 0.005 |
|  | Pakistan | 3091.11  (1546.05 to 5336.95) | 2904.80  (1443.13 to 4997.13) | -0.19  (-0.29 to -0.08) | 0.001 | 7580.67  (5772.60 to 9747.05) | 7601.60  (5745.83 to 9807.97) | 0.01  (-0.01 to 0.03) | 0.518 |
|  | Palau | 13273.25  (7093.31 to 21773.53) | 13251.65  (6988.08 to 22171.43) | 0  (-0.02 to 0.02) | 0.946 | 18132.52  (13959.60 to 22737.28) | 18461.97  (14415.78 to 23157.16) | 0.05  (0.03 to 0.08) | <0.001 |
|  | Palestine | 5227.92  (2746.34 to 8893.43) | 5165.58  (2674.94 to 8861.65) | -0.05  (-0.15 to 0.04) | 0.28 | 13876.16  (10622.45 to 17578.87) | 13861.43  (10729.79 to 17358.82) | -0.01  (-0.02 to 0) | 0.145 |
|  | Panama | 11394.76  (6188.11 to 18598.87) | 11058.66  (5955.28 to 17956.40) | -0.1  (-0.21 to 0) | 0.056 | 33115.31  (26038.31 to 40695.63) | 33426.68  (26324.35 to 41088.79) | 0.02  (0.01 to 0.03) | <0.001 |
|  | Papua New Guinea | 17067.12  (9507.23 to 27190.23) | 16903.52  (9311.84 to 27094.02) | -0.05  (-0.12 to 0.02) | 0.184 | 22390.27  (17437.80 to 27874.54) | 22685.50  (17620.58 to 28366.74) | 0.04  (-0.04 to 0.11) | 0.319 |
|  | Paraguay | 8013.41  (4241.33 to 13356.79) | 7702.39  (4058.75 to 12905.34) | -0.13  (-0.14 to -0.11) | <0.001 | 32750.12  (25821.01 to 40330.79) | 32873.18  (25904.25 to 40414.06) | 0.01  (-0.01 to 0.03) | 0.171 |
|  | Peru | 5383.67  (2837.51 to 9182.32) | 5146.74  (2659.12 to 8716.48) | -0.14  (-0.18 to -0.11) | <0.001 | 38524.89  (33636.10 to 43271.52) | 37149.51  (29663.70 to 45077.22) | -0.1  (-0.15 to -0.06) | <0.001 |
|  | Philippines | 4578.84  (2367.95 to 7757.82) | 4337.14  (2242.38 to 7333.52) | -0.17  (-0.2 to -0.15) | <0.001 | 15181.64  (11615.68 to 19245.61) | 15526.25  (11945.33 to 19458.89) | 0.07  (0.05 to 0.1) | <0.001 |
|  | Poland | 3725.86  (1908.87 to 6293.43) | 3571.73  (1820.65 to 6115.18) | -0.15  (-0.24 to -0.05) | 0.003 | 5894.89  (4407.19 to 7649.53) | 6000.91  (4490.87 to 7807.76) | 0.05  (0.02 to 0.07) | <0.001 |
|  | Portugal | 1932.59  (989.06 to 3334.78) | 1859.50  (937.67 to 3258.12) | -0.12  (-0.14 to -0.11) | <0.001 | 11401.55  (8673.72 to 14476.54) | 11423.20  (8660.19 to 14457.17) | 0.01  (0 to 0.01) | 0.001 |
|  | Puerto Rico | 7243.19  (3833.19 to 12028.58) | 7054.37  (3715.99 to 11785.18) | -0.09  (-0.17 to -0.01) | 0.021 | 31206.30  (24391.61 to 38344.99) | 31700.07  (24898.21 to 38783.39) | 0.04  (0.03 to 0.06) | <0.001 |
|  | Qatar | 3452.70  (1799.97 to 5867.78) | 3265.50  (1677.78 to 5564.68) | -0.19  (-0.2 to -0.18) | <0.001 | 13874.24  (10555.02 to 17636.75) | 13861.43  (10729.79 to 17358.82) | -0.01  (-0.02 to 0) | 0.189 |
|  | Republic of Korea | 3529.16  (1823.52 to 6055.01) | 3265.67  (1665.86 to 5638.81) | -0.25  (-0.34 to -0.17) | <0.001 | 35941.58  (34258.43 to 37677.73) | 18467.65  (14339.01 to 23514.47) | -2.12  (-2.23 to -2.02) | <0.001 |
|  | Republic of Moldova | 3059.54  (1585.22 to 5232.52) | 3030.98  (1541.17 to 5150.53) | -0.04  (-0.21 to 0.13) | 0.634 | 17851.75  (13910.52 to 22389.38) | 17862.56  (13988.01 to 22339.49) | 0  (-0.01 to 0.01) | 0.887 |
|  | Romania | 4133.85  (2142.51 to 7028.03) | 4027.54  (2079.70 to 6985.04) | -0.08  (-0.16 to 0) | 0.058 | 7389.00  (5621.45 to 9460.39) | 7398.59  (5601.03 to 9423.86) | 0.01  (-0.02 to 0.04) | 0.405 |
|  | Russian Federation | 3038.53  (1533.86 to 5265.76) | 3089.99  (1558.10 to 5401.81) | 0.08  (-0.05 to 0.21) | 0.22 | 16173.91  (12656.39 to 20258.48) | 16247.44  (12654.71 to 20213.35) | 0.02  (0.01 to 0.02) | 0.001 |
|  | Rwanda | 15651.14  (8555.83 to 25230.31) | 14840.08  (8042.12 to 24185.17) | -0.17  (-0.19 to -0.15) | <0.001 | 35448.56  (28374.48 to 43481.81) | 36119.65  (28652.76 to 43938.70) | 0.06  (0.04 to 0.09) | <0.001 |
|  | Saint Kitts and Nevis | 6787.80  (3511.04 to 11502.06) | 6871.59  (3539.88 to 11820.67) | 0.02  (0.01 to 0.03) | 0.001 | 31132.53  (24523.48 to 38317.14) | 31696.86  (24993.41 to 39105.17) | 0.03  (-0.01 to 0.07) | 0.12 |
|  | Saint Lucia | 7390.40  (3957.97 to 12234.01) | 7193.27  (3771.50 to 11952.08) | -0.11  (-0.21 to -0.01) | 0.026 | 31089.14  (24482.58 to 38146.81) | 31641.43  (25082.25 to 39048.30) | 0.05  (0.03 to 0.07) | <0.001 |
|  | Saint Vincent and the Grenadines | 7359.92  (3912.46 to 12298.22) | 7257.16  (3768.76 to 12106.25) | -0.06  (-0.11 to -0.01) | 0.017 | 31136.49  (24559.54 to 38460.33) | 31671.71  (24846.61 to 39066.98) | 0.05  (0.03 to 0.06) | <0.001 |
|  | Samoa | 14509.19  (7861.12 to 23475.45) | 13878.08  (7540.45 to 22379.73) | -0.15  (-0.15 to -0.14) | <0.001 | 18132.52  (13959.60 to 22737.28) | 18409.89  (14325.87 to 23202.43) | 0.04  (0.03 to 0.06) | <0.001 |
|  | San Marino | 1437.32  (721.84 to 2488.77) | 1447.24  (720.82 to 2539.85) | 0.02  (0.01 to 0.03) | <0.001 | 11420.45  (8677.93 to 14488.37) | 11361.18  (8727.40 to 14466.12) | -0.02  (-0.02 to -0.01) | <0.001 |
|  | Sao Tome and Principe | 12637.05  (6783.87 to 20897.35) | 12041.00  (6493.97 to 19832.46) | -0.17  (-0.2 to -0.14) | <0.001 | 31999.58  (25221.05 to 39279.28) | 32224.83  (25515.86 to 39591.45) | 0.02  (0.02 to 0.03) | <0.001 |
|  | Saudi Arabia | 4089.79  (2134.17 to 6978.51) | 3856.86  (1983.84 to 6597.53) | -0.19  (-0.25 to -0.13) | <0.001 | 13874.24  (10555.02 to 17636.75) | 13817.78  (10623.68 to 17520.59) | -0.02  (-0.02 to -0.01) | <0.001 |
|  | Senegal | 12900.46  (7050.42 to 21086.86) | 12319.24  (6701.93 to 20490.05) | -0.15  (-0.17 to -0.14) | <0.001 | 25959.60  (20272.11 to 32195.61) | 25580.93  (19976.48 to 31655.27) | -0.05  (-0.1 to 0) | 0.036 |
|  | Serbia | 4111.61  (2152.68 to 6989.92) | 3940.32  (2016.26 to 6681.24) | -0.15  (-0.21 to -0.08) | <0.001 | 7389.00  (5621.45 to 9460.39) | 7394.80  (5606.55 to 9472.34) | 0  (-0.01 to 0) | 0.773 |
|  | Seychelles | 4950.33  (2589.79 to 8355.12) | 4701.36  (2440.63 to 7986.70) | -0.18  (-0.2 to -0.15) | <0.001 | 18777.23  (14610.87 to 23534.02) | 18852.69  (14561.59 to 23645.74) | 0.01  (0 to 0.02) | 0.007 |
|  | Sierra Leone | 12785.60  (6881.64 to 21025.65) | 12202.81  (6549.96 to 19931.51) | -0.15  (-0.19 to -0.12) | <0.001 | 32048.71  (25250.28 to 39326.33) | 32327.56  (25636.76 to 39716.75) | 0.03  (0.02 to 0.04) | <0.001 |
|  | Singapore | 3456.36  (1786.24 to 5965.62) | 3268.03  (1682.67 to 5551.13) | -0.19  (-0.22 to -0.16) | <0.001 | 13524.14  (10407.47 to 16980.02) | 13543.95  (10330.78 to 17066.56) | 0.01  (-0.01 to 0.03) | 0.378 |
|  | Slovakia | 4080.04  (2130.88 to 6903.84) | 3932.57  (2033.27 to 6751.90) | -0.1  (-0.28 to 0.08) | 0.282 | 7389.00  (5621.45 to 9460.39) | 7407.12  (5609.20 to 9544.85) | 0  (-0.01 to 0.02) | 0.558 |
|  | Slovenia | 4048.70  (2127.22 to 6912.99) | 3869.77  (1984.53 to 6648.96) | -0.13  (-0.22 to -0.03) | 0.009 | 7389.00  (5621.45 to 9460.39) | 7394.80  (5606.55 to 9472.34) | 0  (-0.01 to 0) | 0.773 |
|  | Solomon Islands | 14632.72  (8042.14 to 23462.61) | 13994.36  (7693.91 to 22631.79) | -0.13  (-0.16 to -0.1) | <0.001 | 18132.52  (13959.60 to 22737.28) | 18417.30  (14368.93 to 23032.94) | 0.04  (0.02 to 0.07) | <0.001 |
|  | Somalia | 15886.90  (8698.46 to 25896.66) | 15261.69  (8287.96 to 24846.75) | -0.14  (-0.18 to -0.1) | <0.001 | 37532.36  (29969.78 to 45538.80) | 37741.77  (30068.42 to 45701.48) | 0.02  (0.01 to 0.02) | 0.001 |
|  | South Africa | 25274.65  (14208.91 to 38595.49) | 19907.20  (10770.70 to 31426.05) | -0.75  (-1.13 to -0.38) | <0.001 | 43698.45  (35730.53 to 51769.51) | 46344.26  (37940.63 to 54675.90) | 0.21  (0.14 to 0.28) | <0.001 |
|  | South Sudan | 15657.00  (8601.18 to 25183.00) | 15147.78  (8259.50 to 24792.08) | -0.11  (-0.12 to -0.1) | <0.001 | 37561.16  (29957.10 to 45806.82) | 38081.52  (30234.76 to 46470.81) | 0.04  (0.04 to 0.05) | <0.001 |
|  | Spain | 1577.38  (811.32 to 2747.50) | 1507.35  (762.81 to 2613.54) | -0.15  (-0.26 to -0.04) | 0.006 | 8308.99  (6687.90 to 10129.16) | 8881.72  (6748.83 to 11268.85) | 0.22  (0.18 to 0.26) | <0.001 |
|  | Sri Lanka | 7280.36  (3920.03 to 12315.68) | 7079.40  (3704.85 to 12037.90) | -0.1  (-0.13 to -0.06) | <0.001 | 18833.69  (14712.15 to 23522.16) | 18797.81  (14641.32 to 23502.93) | -0.01  (-0.01 to 0) | 0.006 |
|  | Sudan | 5899.02  (3088.83 to 10039.21) | 5725.73  (3011.82 to 9691.07) | 0.1  (-0.25 to 0.45) | 0.573 | 13838.82  (10598.39 to 17370.68) | 13847.02  (10627.69 to 17550.44) | -0.01  (-0.03 to 0.01) | 0.579 |
|  | Suriname | 7546.49  (4010.83 to 12566.85) | 7334.28  (3856.05 to 12372.18) | -0.12  (-0.22 to -0.03) | 0.008 | 31132.53  (24523.48 to 38317.14) | 31765.90  (25052.81 to 39194.29) | 0.06  (0.05 to 0.07) | <0.001 |
|  | Sweden | 1551.92  (770.54 to 2715.71) | 1502.01  (734.22 to 2680.04) | -0.12  (-0.15 to -0.09) | <0.001 | 19144.17  (15245.64 to 23395.18) | 17140.06  (13206.43 to 21545.01) | -0.35  (-0.38 to -0.31) | <0.001 |
|  | Switzerland | 1555.30  (806.90 to 2698.36) | 1512.76  (766.84 to 2623.89) | -0.08  (-0.14 to -0.02) | 0.006 | 13090.06  (11594.22 to 14717.02) | 12082.82  (9264.53 to 15338.04) | -0.26  (-0.3 to -0.22) | <0.001 |
|  | Syrian Arab Republic | 3572.58  (1848.85 to 6141.30) | 3396.35  (1757.64 to 5858.89) | -0.16  (-0.27 to -0.05) | 0.003 | 10664.28  (8116.57 to 13588.82) | 10889.93  (8313.59 to 13826.06) | 0.06  (0.04 to 0.08) | <0.001 |
|  | Taiwan (Province of China) | 3506.17  (2249.82 to 5206.04) | 3641.45  (1892.74 to 6294.42) | 0.13  (0.06 to 0.2) | <0.001 | 12144.79  (9295.07 to 15502.04) | 12063.94  (9312.81 to 15194.03) | -0.02  (-0.04 to 0) | 0.012 |
|  | Tajikistan | 6583.16  (3491.42 to 11063.07) | 6326.83  (3274.53 to 10757.59) | -0.12  (-0.14 to -0.1) | <0.001 | 10020.80  (7627.31 to 12808.71) | 10078.45  (7629.08 to 12790.46) | 0.02  (0 to 0.04) | 0.042 |
|  | Thailand | 4974.44  (2614.58 to 8446.74) | 4674.53  (2412.24 to 7916.51) | -0.19  (-0.22 to -0.16) | <0.001 | 28688.77  (26336.12 to 31143.64) | 24805.32  (19419.91 to 30854.26) | -0.52  (-0.7 to -0.34) | <0.001 |
|  | Timor-Leste | 5224.77  (2729.35 to 8876.66) | 4869.48  (2534.59 to 8360.92) | -0.23  (-0.25 to -0.2) | <0.001 | 18823.40  (14636.20 to 23638.28) | 18801.07  (14670.40 to 23408.70) | 0  (-0.01 to 0.01) | 0.904 |
|  | Togo | 8475.49  (4498.31 to 13991.04) | 7922.86  (4156.63 to 13351.87) | -0.23  (-0.35 to -0.11) | <0.001 | 32058.97  (24997.15 to 39263.94) | 32400.19  (25629.21 to 39811.83) | 0.03  (0.02 to 0.04) | <0.001 |
|  | Tokelau | 13371.02  (7094.26 to 22032.64) | 13223.93  (7058.45 to 21803.57) | -0.03  (-0.04 to -0.03) | <0.001 | 18132.52  (13959.60 to 22737.28) | 18463.40  (14415.97 to 23178.61) | 0.05  (0.04 to 0.06) | <0.001 |
|  | Tonga | 14207.49  (7725.34 to 22969.67) | 13724.16  (7350.48 to 22349.78) | -0.12  (-0.14 to -0.1) | <0.001 | 18065.24  (14009.11 to 22488.37) | 18378.57  (14364.85 to 23024.03) | 0.05  (0.01 to 0.09) | 0.018 |
|  | Trinidad and Tobago | 7334.23  (3881.95 to 12298.08) | 7234.17  (3777.37 to 12060.64) | -0.06  (-0.21 to 0.09) | 0.42 | 31240.60  (24677.78 to 38408.49) | 31788.66  (25022.72 to 39371.15) | 0.05  (0.04 to 0.06) | <0.001 |
|  | Tunisia | 5574.03  (2916.12 to 9521.93) | 5328.62  (2764.20 to 9079.34) | -0.22  (-0.39 to -0.04) | 0.016 | 13876.16  (10622.45 to 17578.87) | 13847.84  (10712.53 to 17364.62) | 0  (-0.03 to 0.02) | 0.675 |
|  | Turkmenistan | 6467.73  (3372.51 to 10909.70) | 6190.84  (3179.47 to 10492.25) | -0.14  (-0.15 to -0.12) | <0.001 | 10075.10  (7594.89 to 12918.54) | 10082.27  (7632.18 to 12944.72) | 0  (-0.02 to 0.01) | 0.75 |
|  | Tuvalu | 13384.35  (7157.48 to 22078.95) | 13337.36  (7092.39 to 21879.33) | -0.02  (-0.03 to -0.01) | 0.002 | 18132.52  (13959.60 to 22737.28) | 18463.40  (14415.97 to 23178.61) | 0.05  (0.01 to 0.09) | 0.008 |
|  | Türkiye | 2066.91  (1067.66 to 3492.27) | 1883.15  (958.28 to 3218.49) | -0.06 (-1.73 to 1.64) | 0.944 | 12264.68  (9355.05 to 15535.93) | 11806.68  (8991.24 to 14972.88) | -0.12 (-0.18 to -0.07) | <0.001 |
|  | Uganda | 14162.45  (7729.43 to 22996.42) | 13634.86  (7400.48 to 22549.91) | -0.13  (-0.37 to 0.11) | 0.293 | 62423.29  (57106.72 to 66928.84) | 45208.60  (36964.98 to 53922.62) | -1.03  (-1.08 to -0.98) | <0.001 |
|  | Ukraine | 3059.42  (1519.41 to 5375.55) | 3035.46  (1522.55 to 5305.96) | -0.04  (-0.23 to 0.16) | 0.717 | 16157.92  (12558.79 to 20008.79) | 16243.77  (12570.46 to 20502.35) | 0.01  (-0.01 to 0.02) | 0.224 |
|  | United Arab Emirates | 3507.76  (1837.67 to 6028.28) | 3281.87  (1723.91 to 5605.94) | -0.21  (-0.25 to -0.16) | <0.001 | 13876.16  (10622.45 to 17578.87) | 13847.84  (10712.53 to 17364.62) | -0.01  (-0.02 to 0) | 0.017 |
|  | United Kingdom | 1595.89  (798.15 to 2784.83) | 1552.91  (773.34 to 2702.90) | -0.1  (-0.13 to -0.06) | <0.001 | 9757.49  (7462.80 to 12423.55) | 9908.69  (7607.54 to 12543.55) | 0.06  (0.02 to 0.09) | 0.003 |
|  | United Republic of Tanzania | 17729.06  (10036.76 to 27752.31) | 13375.50  (7243.34 to 22155.56) | -0.87  (-1.13 to -0.61) | <0.001 | 34014.40  (26934.41 to 41706.07) | 34547.41  (27525.34 to 42126.37) | 0.04  (-0.1 to 0.17) | 0.614 |
|  | United States of America | 7053.20  (3653.64 to 11806.67) | 6758.09  (3441.24 to 11480.69) | -0.14  (-0.19 to -0.1) | <0.001 | 20780.29  (16327.37 to 25851.07) | 17926.17  (13975.42 to 22482.79) | -0.47  (-0.52 to -0.42) | <0.001 |
|  | United States Virgin Islands | 7341.14  (3855.53 to 12255.29) | 7128.43  (3708.15 to 11812.82) | -0.11  (-0.2 to -0.01) | 0.026 | 31240.60  (24677.78 to 38408.49) | 31688.32  (24922.49 to 39185.58) | 0.02  (-0.01 to 0.05) | 0.198 |
|  | Uruguay | 3269.92  (1690.01 to 5576.45) | 3100.09  (1605.56 to 5251.83) | -0.17  (-0.2 to -0.15) | <0.001 | 28717.78  (22688.59 to 35422.12) | 28819.55  (22487.15 to 35506.14) | 0.01  (0.01 to 0.01) | <0.001 |
|  | Uzbekistan | 6424.05  (3399.60 to 10871.55) | 6156.75  (3249.72 to 10227.40) | -0.13  (-0.17 to -0.09) | <0.001 | 10020.80  (7627.31 to 12808.71) | 10085.88  (7639.54 to 12901.45) | 0.02  (0.01 to 0.02) | <0.001 |
|  | Vanuatu | 14671.86  (8143.29 to 23567.24) | 14086.02  (7628.80 to 22637.02) | -0.13  (-0.14 to -0.13) | <0.001 | 14500.66  (11127.34 to 18175.53) | 14883.27  (11431.93 to 18751.94) | 0.07  (0.05 to 0.09) | <0.001 |
|  | Venezuela (Bolivarian Republic of) | 11468.55  (6170.16 to 18861.96) | 11086.45  (5897.16 to 18089.57) | -0.1  (-0.14 to -0.06) | <0.001 | 33160.48  (26368.15 to 40798.43) | 33270.56  (26250.53 to 40950.96) | 0.01  (-0.01 to 0.03) | 0.225 |
|  | Viet Nam | 3376.48  (1722.23 to 5737.86) | 3040.38  (1550.52 to 5219.65) | -0.36  (-0.43 to -0.28) | <0.001 | 17418.16  (13997.48 to 21302.58) | 17543.43  (13672.58 to 21980.84) | 0.02  (0.02 to 0.03) | <0.001 |
|  | Yemen | 3651.60  (1884.26 to 6250.83) | 3474.75  (1784.60 to 5922.37) | -0.17  (-0.19 to -0.14) | <0.001 | 13876.16  (10622.45 to 17578.87) | 13842.53  (10594.64 to 17593.09) | -0.01  (-0.02 to 0) | 0.024 |
|  | Zambia | 21883.23  (12460.72 to 34374.37) | 21910.47  (12391.83 to 34278.10) | -0.03  (-0.22 to 0.15) | 0.716 | 41387.93  (33205.63 to 49930.16) | 42533.62  (34544.35 to 51015.32) | 0.07  (0 to 0.13) | 0.035 |
|  | Zimbabwe | 20710.30  (11678.73 to 32316.31) | 20746.34  (11537.16 to 32444.32) | -0.03  (-0.1 to 0.04) | 0.404 | 42564.32  (34283.87 to 51085.33) | 45533.52  (36959.06 to 54614.97) | 0.18  (0.09 to 0.27) | <0.001 |
| Age-standardized DALYs | Afghanistan | 7.00  (2.29 to 16.57) | 6.63  (2.10 to 15.71) | -0.17  (-0.21 to -0.14) | <0.001 | 3.81  (1.53 to 7.75) | 3.82  (1.58 to 7.83) | 0  (-0.01 to 0.01) | 0.898 |
|  | Albania | 8.04  (2.61 to 18.77) | 7.60  (2.44 to 17.73) | -0.17  (-0.29 to -0.06) | 0.004 | 2.09  (0.86 to 4.27) | 2.10  (0.86 to 4.41) | 0.01  (-0.03 to 0.04) | 0.764 |
|  | Algeria | 6.78  (2.19 to 15.80) | 6.32  (2.08 to 15.04) | -0.22  (-0.25 to -0.19) | <0.001 | 3.85  (1.59 to 7.90) | 3.84  (1.58 to 8.06) | 0  (-0.05 to 0.05) | 0.864 |
|  | American Samoa | 27.47  (9.32 to 62.78) | 26.29  (8.67 to 60.16) | -0.14  (-0.18 to -0.11) | <0.001 | 5.02  (2.02 to 10.62) | 5.13  (2.03 to 10.43) | 0.04  (-0.01 to 0.1) | 0.086 |
|  | Andorra | 2.95  (0.93 to 6.89) | 2.84  (0.87 to 6.84) | -0.14  (-0.21 to -0.06) | <0.001 | 3.18  (1.31 to 6.49) | 3.15  (1.27 to 6.50) | -0.03  (-0.05 to -0.01) | 0.009 |
|  | Angola | 14.58  (4.82 to 33.73) | 13.70  (4.55 to 31.60) | -0.2  (-0.24 to -0.16) | <0.001 | 15.23  (6.38 to 30.70) | 15.30  (6.37 to 31.01) | 0.01  (-0.04 to 0.05) | 0.775 |
|  | Antigua and Barbuda | 13.80  (4.70 to 32.00) | 13.64  (4.56 to 31.30) | -0.05  (-0.13 to 0.03) | 0.188 | 8.63  (3.61 to 17.68) | 8.72  (3.58 to 17.98) | 0.04  (-0.02 to 0.1) | 0.216 |
|  | Argentina | 6.26  (2.05 to 14.96) | 5.95  (1.93 to 13.86) | -0.16  (-0.21 to -0.12) | <0.001 | 8.67  (3.66 to 17.82) | 7.94  (3.20 to 16.27) | -0.29  (-0.32 to -0.26) | <0.001 |
|  | Armenia | 12.30  (3.99 to 29.09) | 11.77  (3.82 to 27.47) | -0.14  (-0.18 to -0.1) | <0.001 | 2.83  (1.13 to 5.96) | 2.86  (1.13 to 5.97) | 0.04  (-0.04 to 0.11) | 0.357 |
|  | Australia | 5.19  (1.67 to 12.30) | 5.04  (1.64 to 12.10) | -0.11  (-0.17 to -0.05) | <0.001 | 5.75  (2.32 to 11.78) | 4.14  (1.66 to 8.35) | -1.04  (-1.11 to -0.97) | <0.001 |
|  | Austria | 2.97  (0.94 to 7.13) | 2.88  (0.89 to 6.79) | -0.09  (-0.17 to -0.02) | 0.014 | 2.58  (1.05 to 5.38) | 2.60  (1.09 to 5.38) | 0.03  (-0.02 to 0.08) | 0.284 |
|  | Azerbaijan | 12.48  (4.23 to 29.13) | 11.89  (3.98 to 27.56) | -0.16  (-0.18 to -0.14) | <0.001 | 2.83  (1.15 to 5.93) | 2.85  (1.12 to 5.91) | 0.01  (-0.07 to 0.09) | 0.868 |
|  | Bahamas | 14.10  (4.67 to 32.74) | 13.75  (4.48 to 31.50) | -0.12  (-0.25 to 0) | 0.052 | 8.60  (3.59 to 17.86) | 8.76  (3.63 to 17.94) | 0.05  (0.01 to 0.08) | 0.004 |
|  | Bahrain | 6.61  (2.13 to 15.71) | 6.24  (2.01 to 15.04) | -0.19  (-0.23 to -0.15) | <0.001 | 3.85  (1.55 to 7.82) | 3.83  (1.56 to 7.90) | -0.01  (-0.05 to 0.02) | 0.404 |
|  | Bangladesh | 6.59  (2.15 to 15.55) | 6.25  (2.05 to 15.01) | -0.16  (-0.2 to -0.13) | <0.001 | 2.28  (0.92 to 4.76) | 2.27  (0.93 to 4.61) | -0.01  (-0.02 to 0) | 0.189 |
|  | Barbados | 13.94  (4.62 to 32.13) | 13.69  (4.59 to 32.25) | -0.07  (-0.12 to -0.03) | 0.001 | 8.63  (3.55 to 17.54) | 8.75  (3.59 to 18.00) | 0.01  (-0.01 to 0.04) | 0.258 |
|  | Belarus | 5.75  (1.83 to 13.64) | 5.68  (1.83 to 13.56) | -0.04  (-0.22 to 0.13) | 0.614 | 4.92  (1.99 to 10.02) | 4.94  (1.97 to 10.34) | 0  (-0.02 to 0.03) | 0.674 |
|  | Belgium | 1.77  (0.55 to 4.22) | 1.67  (0.49 to 3.94) | -0.23  (-0.41 to -0.05) | 0.013 | 3.25  (1.34 to 6.61) | 3.25  (1.31 to 6.65) | 0.01  (-0.08 to 0.09) | 0.872 |
|  | Belize | 14.11  (4.78 to 32.91) | 13.76  (4.57 to 32.20) | -0.09  (-0.16 to -0.02) | 0.009 | 8.60  (3.47 to 17.77) | 8.77  (3.56 to 18.13) | 0.03  (-0.01 to 0.08) | 0.117 |
|  | Benin | 14.63  (4.85 to 34.02) | 13.69  (4.41 to 31.92) | -0.17  (-0.28 to -0.06) | 0.003 | 8.22  (3.46 to 16.81) | 8.29  (3.42 to 16.79) | 0.03  (0.01 to 0.04) | <0.001 |
|  | Bermuda | 13.81  (4.53 to 32.28) | 13.46  (4.36 to 31.48) | -0.11  (-0.21 to -0.02) | 0.024 | 8.61  (3.55 to 17.64) | 8.75  (3.59 to 18.27) | 0.04  (-0.01 to 0.09) | 0.098 |
|  | Bhutan | 6.56  (2.17 to 15.59) | 6.14  (1.96 to 14.38) | -0.2  (-0.26 to -0.14) | <0.001 | 2.28  (0.94 to 4.73) | 2.28  (0.92 to 4.66) | 0  (-0.01 to 0.01) | 0.508 |
|  | Bolivia (Plurinational State of) | 10.46  (3.47 to 24.22) | 9.97  (3.26 to 23.52) | -0.15  (-0.19 to -0.11) | <0.001 | 10.20  (4.26 to 21.13) | 10.18  (4.24 to 20.83) | 0  (-0.04 to 0.05) | 0.945 |
|  | Bosnia and Herzegovina | 7.90  (2.60 to 18.72) | 7.60  (2.47 to 17.75) | -0.11  (-0.28 to 0.05) | 0.186 | 2.10  (0.88 to 4.44) | 2.11  (0.85 to 4.40) | 0  (-0.01 to 0.01) | 0.746 |
|  | Botswana | 38.39  (14.49 to 83.22) | 31.28  (10.73 to 71.49) | -0.69  (-0.8 to -0.59) | <0.001 | 11.61  (4.86 to 23.79) | 12.21  (5.08 to 25.11) | 0.16  (0.09 to 0.23) | <0.001 |
|  | Brazil | 15.56  (5.06 to 36.15) | 15.26  (5.02 to 35.74) | -0.06  (-0.07 to -0.04) | <0.001 | 9.27  (3.93 to 18.81) | 8.63  (3.64 to 17.45) | -0.23  (-0.28 to -0.19) | <0.001 |
|  | Brunei Darussalam | 6.69  (2.14 to 16.02) | 6.33  (2.01 to 15.25) | -0.17  (-0.23 to -0.1) | <0.001 | 3.82  (1.58 to 7.78) | 3.81  (1.56 to 7.87) | -0.01  (-0.06 to 0.05) | 0.83 |
|  | Bulgaria | 7.79  (2.55 to 18.80) | 7.50  (2.36 to 17.73) | -0.12  (-0.2 to -0.03) | 0.011 | 2.88  (1.18 to 5.93) | 2.88  (1.18 to 5.90) | 0.01  (-0.14 to 0.16) | 0.889 |
|  | Burkina Faso | 50.53  (20.12 to 106.75) | 33.44  (11.39 to 76.63) | -1.36  (-1.44 to -1.28) | <0.001 | 8.13  (3.43 to 16.65) | 8.06  (3.33 to 16.53) | -0.04  (-0.09 to 0.01) | 0.118 |
|  | Burundi | 29.48  (9.98 to 66.89) | 28.39  (9.62 to 63.94) | -0.12  (-0.15 to -0.09) | <0.001 | 10.38  (4.31 to 21.12) | 10.36  (4.35 to 21.12) | -0.01  (-0.04 to 0.02) | 0.527 |
|  | Cabo Verde | 23.90  (8.20 to 54.63) | 22.77  (7.40 to 52.01) | -0.17  (-0.21 to -0.14) | <0.001 | 8.82  (3.70 to 18.13) | 8.83  (3.66 to 18.12) | -0.01  (-0.04 to 0.03) | 0.783 |
|  | Cambodia | 9.89  (3.34 to 23.38) | 9.25  (3.04 to 22.06) | -0.23  (-0.27 to -0.18) | <0.001 | 5.22  (2.10 to 10.80) | 5.24  (2.16 to 10.64) | 0.02  (0 to 0.04) | 0.086 |
|  | Cameroon | 29.75  (10.14 to 68.12) | 29.11  (9.84 to 66.80) | -0.09  (-0.13 to -0.04) | <0.001 | 10.64  (4.47 to 21.56) | 10.81  (4.49 to 21.97) | 0.05  (-0.1 to 0.19) | 0.516 |
|  | Canada | 12.56  (4.15 to 29.68) | 12.17  (4.02 to 28.64) | -0.11  (-0.13 to -0.08) | <0.001 | 3.09  (1.30 to 6.24) | 3.06  (1.25 to 6.33) | -0.01  (-0.19 to 0.17) | 0.901 |
|  | Central African Republic | 14.47  (4.77 to 33.97) | 13.98  (4.53 to 33.25) | -0.09  (-0.13 to -0.05) | <0.001 | 15.28  (6.41 to 30.77) | 15.29  (6.43 to 31.52) | -0.03  (-0.13 to 0.07) | 0.573 |
|  | Chad | 24.65  (8.44 to 56.16) | 23.54  (7.80 to 53.46) | -0.16  (-0.21 to -0.11) | <0.001 | 6.97  (2.84 to 14.36) | 7.06  (2.95 to 14.70) | 0.03  (-0.04 to 0.11) | 0.378 |
|  | Chile | 6.20  (1.97 to 14.55) | 5.90  (1.93 to 14.02) | -0.17  (-0.23 to -0.1) | <0.001 | 7.91  (3.20 to 16.30) | 7.90  (3.20 to 16.10) | 0.02  (0 to 0.04) | 0.047 |
|  | China | 7.49  (2.39 to 17.75) | 7.11  (2.27 to 16.90) | -0.16  (-0.29 to -0.02) | 0.021 | 3.30  (1.40 to 6.64) | 3.32  (1.39 to 6.77) | 0  (-0.13 to 0.12) | 0.982 |
|  | Colombia | 21.92  (7.35 to 50.41) | 21.15  (6.91 to 48.74) | -0.12  (-0.19 to -0.04) | 0.004 | 12.41  (5.29 to 25.33) | 9.98  (4.07 to 20.40) | -0.72  (-0.81 to -0.63) | <0.001 |
|  | Comoros | 29.15  (9.99 to 65.97) | 28.26  (9.64 to 64.59) | -0.11  (-0.14 to -0.08) | <0.001 | 10.21  (4.20 to 20.81) | 10.28  (4.24 to 21.00) | 0.03  (0 to 0.06) | 0.081 |
|  | Congo | 14.30  (4.75 to 33.06) | 13.53  (4.44 to 30.47) | -0.16  (-0.21 to -0.11) | <0.001 | 15.33  (6.44 to 31.10) | 15.28  (6.32 to 31.17) | -0.02  (-0.07 to 0.03) | 0.444 |
|  | Cook Islands | 25.41  (8.35 to 58.48) | 25.17  (8.35 to 59.29) | -0.03  (-0.06 to -0.01) | 0.001 | 5.03  (2.01 to 10.23) | 5.14  (2.07 to 10.59) | 0.04  (-0.01 to 0.1) | 0.128 |
|  | Costa Rica | 21.57  (7.27 to 49.19) | 20.96  (7.04 to 48.60) | -0.09  (-0.13 to -0.05) | <0.001 | 9.86  (4.17 to 20.60) | 8.93  (3.65 to 18.03) | -0.32  (-0.42 to -0.21) | <0.001 |
|  | Coted'Ivoire | 20.49  (6.91 to 47.42) | 19.69  (6.44 to 45.54) | -0.14  (-0.23 to -0.05) | 0.004 | 8.85  (3.53 to 18.21) | 8.90  (3.60 to 18.14) | 0.02  (-0.01 to 0.04) | 0.191 |
|  | Croatia | 7.71  (2.52 to 18.39) | 7.45  (2.41 to 17.14) | -0.1  (-0.2 to 0) | 0.05 | 2.34  (0.95 to 4.85) | 2.33  (0.95 to 4.81) | -0.03  (-0.09 to 0.04) | 0.451 |
|  | Cuba | 13.72  (4.56 to 32.39) | 13.47  (4.41 to 31.63) | -0.08  (-0.21 to 0.04) | 0.182 | 8.57  (3.57 to 17.46) | 8.75  (3.61 to 17.77) | 0.04  (0 to 0.08) | 0.039 |
|  | Cyprus | 3.03  (0.95 to 7.23) | 2.88  (0.90 to 6.95) | -0.16  (-0.29 to -0.04) | 0.009 | 3.18  (1.30 to 6.50) | 3.17  (1.29 to 6.50) | -0.01  (-0.04 to 0.03) | 0.685 |
|  | Czechia | 7.71  (2.52 to 18.11) | 7.43  (2.42 to 17.75) | -0.12  (-0.2 to -0.03) | 0.011 | 1.81  (0.78 to 3.72) | 1.84  (0.75 to 3.82) | 0.04  (-0.01 to 0.09) | 0.105 |
|  | Democratic People's Republic of Korea | 7.32  (2.39 to 17.41) | 7.01  (2.23 to 16.81) | -0.14  (-0.19 to -0.09) | <0.001 | 3.43  (1.40 to 7.15) | 3.43  (1.37 to 7.06) | -0.01  (-0.08 to 0.06) | 0.808 |
|  | Democratic Republic of the Congo | 14.42  (4.79 to 33.68) | 13.75  (4.52 to 32.27) | -0.13  (-0.19 to -0.07) | <0.001 | 15.17  (6.36 to 30.34) | 15.22  (6.37 to 30.30) | -0.01  (-0.04 to 0.02) | 0.493 |
|  | Denmark | 2.97  (0.94 to 7.04) | 2.86  (0.91 to 6.75) | -0.15  (-0.22 to -0.07) | <0.001 | 4.61  (1.83 to 9.54) | 3.85  (1.55 to 8.07) | -0.58  (-0.69 to -0.47) | <0.001 |
|  | Djibouti | 29.52  (10.07 to 66.08) | 28.34  (9.43 to 65.23) | -0.13  (-0.17 to -0.09) | <0.001 | 9.73  (4.03 to 20.17) | 9.82  (4.08 to 19.96) | 0.02  (-0.07 to 0.11) | 0.669 |
|  | Dominica | 14.01  (4.65 to 32.86) | 13.63  (4.57 to 31.94) | -0.1  (-0.23 to 0.03) | 0.117 | 8.62  (3.55 to 17.33) | 8.72  (3.62 to 17.88) | 0.02  (0 to 0.04) | 0.091 |
|  | Dominican Republic | 14.02  (4.61 to 32.92) | 13.66  (4.35 to 31.59) | -0.09  (-0.16 to -0.02) | 0.008 | 8.60  (3.57 to 17.86) | 8.72  (3.57 to 17.99) | 0.03  (0 to 0.06) | 0.033 |
|  | Ecuador | 10.19  (3.36 to 23.61) | 9.91  (3.21 to 23.24) | -0.09  (-0.16 to -0.02) | 0.011 | 10.20  (4.23 to 21.00) | 10.23  (4.22 to 20.76) | 0.02  (0 to 0.04) | 0.084 |
|  | Egypt | 9.83  (3.29 to 22.82) | 9.30  (3.13 to 21.73) | -0.23  (-0.35 to -0.11) | <0.001 | 3.84  (1.58 to 7.75) | 3.84  (1.58 to 7.99) | 0.01  (-0.03 to 0.04) | 0.666 |
|  | El Salvador | 21.99  (7.43 to 50.68) | 21.24  (7.08 to 49.19) | -0.11  (-0.16 to -0.06) | <0.001 | 9.13  (3.79 to 18.71) | 9.16  (3.80 to 18.38) | 0.01  (-0.04 to 0.06) | 0.716 |
|  | Equatorial Guinea | 14.60  (4.88 to 33.93) | 13.53  (4.52 to 31.20) | -0.24  (-0.29 to -0.2) | <0.001 | 15.16  (6.49 to 30.78) | 15.54  (6.45 to 31.19) | 0.06  (-0.03 to 0.14) | 0.172 |
|  | Eritrea | 29.53  (10.07 to 67.03) | 28.33  (9.70 to 64.16) | -0.14  (-0.17 to -0.11) | <0.001 | 7.86  (3.28 to 15.92) | 8.34  (3.45 to 17.04) | 0.18  (0.1 to 0.26) | <0.001 |
|  | Estonia | 5.78  (1.87 to 13.57) | 5.68  (1.85 to 13.17) | -0.04  (-0.29 to 0.22) | 0.776 | 4.94  (1.99 to 10.31) | 4.95  (2.02 to 10.38) | -0.02  (-0.1 to 0.06) | 0.668 |
|  | Eswatini | 36.42  (12.52 to 82.76) | 35.01  (12.06 to 79.47) | -0.14  (-0.18 to -0.1) | <0.001 | 11.58  (4.90 to 23.74) | 12.81  (5.38 to 26.56) | 0.34  (0.29 to 0.38) | <0.001 |
|  | Ethiopia | 30.20  (10.10 to 69.97) | 29.01  (9.55 to 67.79) | -0.14  (-0.17 to -0.1) | <0.001 | 7.21  (3.05 to 14.50) | 7.28  (3.07 to 14.91) | -0.03  (-0.21 to 0.15) | 0.764 |
|  | Fiji | 21.37  (8.01 to 46.81) | 21.88  (7.17 to 51.08) | 0.07  (0.02 to 0.12) | 0.007 | 5.01  (2.00 to 10.24) | 5.11  (2.06 to 10.72) | 0.05  (-0.02 to 0.13) | 0.157 |
|  | Finland | 2.96  (0.95 to 7.00) | 2.86  (0.86 to 6.88) | -0.11  (-0.25 to 0.03) | 0.116 | 4.86  (2.00 to 10.11) | 3.15  (1.30 to 6.51) | -1.39  (-1.48 to -1.31) | <0.001 |
|  | France | 4.35  (1.41 to 10.40) | 4.10  (1.28 to 9.78) | -0.19  (-0.26 to -0.12) | <0.001 | 3.10  (1.25 to 6.40) | 3.11  (1.27 to 6.27) | 0  (-0.05 to 0.06) | 0.952 |
|  | Gabon | 14.30  (4.79 to 33.40) | 13.56  (4.49 to 31.67) | -0.17  (-0.19 to -0.15) | <0.001 | 15.31  (6.42 to 31.67) | 15.41  (6.49 to 31.32) | -0.02  (-0.08 to 0.04) | 0.463 |
|  | Gambia | 23.90  (8.02 to 54.60) | 22.90  (7.74 to 52.17) | -0.14  (-0.19 to -0.09) | <0.001 | 8.30  (3.47 to 17.36) | 8.40  (3.49 to 17.21) | 0.04  (0 to 0.07) | 0.042 |
|  | Georgia | 12.22  (4.00 to 28.52) | 11.71  (3.74 to 27.72) | -0.14  (-0.16 to -0.12) | <0.001 | 2.84  (1.14 to 5.84) | 2.86  (1.18 to 5.85) | 0.01  (-0.06 to 0.07) | 0.88 |
|  | Germany | 2.97  (0.92 to 6.99) | 2.87  (0.89 to 6.80) | -0.13  (-0.2 to -0.05) | 0.001 | 4.83  (1.96 to 10.15) | 3.84  (1.56 to 7.73) | -0.74  (-0.8 to -0.68) | <0.001 |
|  | Ghana | 23.86  (8.05 to 54.66) | 22.86  (7.72 to 53.28) | -0.15  (-0.18 to -0.11) | <0.001 | 8.79  (3.55 to 18.22) | 8.86  (3.67 to 18.05) | 0.04  (-0.02 to 0.09) | 0.236 |
|  | Greece | 4.71  (1.53 to 11.31) | 4.72  (1.52 to 11.25) | 0.03  (-0.07 to 0.13) | 0.584 | 2.54  (1.05 to 5.14) | 2.54  (1.05 to 5.33) | -0.01  (-0.07 to 0.05) | 0.802 |
|  | Greenland | 12.82  (4.25 to 30.00) | 12.40  (4.10 to 29.33) | -0.1  (-0.14 to -0.07) | <0.001 | 3.91  (1.55 to 8.01) | 3.91  (1.61 to 8.10) | 0.01  (0.01 to 0.02) | 0.002 |
|  | Grenada | 14.03  (4.67 to 32.66) | 13.74  (4.67 to 32.15) | -0.1  (-0.22 to 0.02) | 0.09 | 8.63  (3.55 to 17.89) | 8.68  (3.57 to 17.83) | 0.01  (-0.02 to 0.05) | 0.518 |
|  | Guam | 26.81  (9.00 to 62.82) | 25.78  (8.62 to 58.91) | -0.13  (-0.16 to -0.1) | <0.001 | 5.02  (2.03 to 10.43) | 5.14  (2.07 to 10.71) | 0.07  (0 to 0.14) | 0.052 |
|  | Guatemala | 22.13  (7.38 to 50.91) | 21.38  (7.31 to 49.21) | -0.11  (-0.19 to -0.03) | 0.007 | 9.07  (3.74 to 18.40) | 9.13  (3.83 to 18.41) | 0.01  (-0.03 to 0.05) | 0.537 |
|  | Guinea | 24.43  (8.26 to 56.26) | 23.56  (7.92 to 53.89) | -0.12  (-0.15 to -0.09) | <0.001 | 8.74  (3.61 to 17.95) | 8.87  (3.67 to 18.10) | 0.05  (0.03 to 0.07) | <0.001 |
|  | Guinea-Bissau | 24.22  (8.10 to 55.96) | 23.26  (7.78 to 53.97) | -0.14  (-0.2 to -0.08) | <0.001 | 8.73  (3.63 to 18.13) | 8.91  (3.68 to 18.27) | 0.06  (0.02 to 0.1) | 0.003 |
|  | Guyana | 14.10  (4.61 to 32.70) | 13.80  (4.55 to 31.96) | -0.1  (-0.24 to 0.03) | 0.135 | 8.55  (3.59 to 17.48) | 8.73  (3.61 to 17.90) | 0.06  (0.01 to 0.1) | 0.009 |
|  | Haiti | 14.61  (4.86 to 33.74) | 14.10  (4.69 to 33.00) | -0.13  (-0.24 to -0.03) | 0.012 | 6.96  (2.82 to 14.10) | 6.93  (2.83 to 14.28) | -0.06  (-0.08 to -0.03) | <0.001 |
|  | Honduras | 22.31  (7.43 to 51.67) | 21.63  (7.23 to 49.80) | -0.1  (-0.19 to -0.01) | 0.036 | 10.97  (4.60 to 22.37) | 11.00  (4.60 to 22.60) | -0.02  (-0.12 to 0.08) | 0.673 |
|  | Hungary | 7.78  (2.49 to 18.27) | 7.46  (2.41 to 17.61) | -0.12  (-0.21 to -0.03) | 0.011 | 1.62  (0.67 to 3.36) | 1.62  (0.65 to 3.36) | 0.02  (-0.05 to 0.08) | 0.652 |
|  | Iceland | 2.96  (0.91 to 7.01) | 2.86  (0.88 to 6.90) | -0.13  (-0.24 to -0.03) | 0.013 | 3.66  (1.48 to 7.54) | 3.64  (1.47 to 7.53) | -0.03  (-0.11 to 0.06) | 0.561 |
|  | India | 6.46  (2.10 to 15.12) | 6.30  (2.03 to 14.96) | -0.06  (-0.25 to 0.13) | 0.56 | 2.00  (0.85 to 4.03) | 2.25  (0.95 to 4.57) | 0.4  (0.34 to 0.46) | <0.001 |
|  | Indonesia | 9.95  (3.26 to 23.40) | 9.43  (3.08 to 22.21) | -0.14  (-0.2 to -0.09) | <0.001 | 4.11  (1.71 to 8.24) | 4.14  (1.75 to 8.34) | 0.03  (0.01 to 0.05) | 0.004 |
|  | Iran (Islamic Republic of) | 6.06  (1.95 to 14.28) | 5.82  (1.87 to 13.78) | 0.2  (-0.46 to 0.86) | 0.562 | 4.40  (1.85 to 9.01) | 4.40  (1.87 to 8.93) | 0.01  (-0.02 to 0.04) | 0.495 |
|  | Iraq | 15.97  (5.48 to 36.13) | 10.78  (3.56 to 25.14) | -1.21  (-1.45 to -0.95) | <0.001 | 3.84  (1.56 to 7.85) | 3.82  (1.59 to 7.89) | -0.01  (-0.07 to 0.05) | 0.706 |
|  | Ireland | 2.99  (0.96 to 7.05) | 2.85  (0.88 to 6.90) | -0.17  (-0.25 to -0.09) | <0.001 | 3.18  (1.32 to 6.54) | 3.17  (1.30 to 6.63) | -0.01  (-0.04 to 0.02) | 0.393 |
|  | Israel | 3.01  (0.93 to 7.30) | 2.91  (0.90 to 6.81) | -0.1  (-0.2 to -0.01) | 0.038 | 3.18  (1.30 to 6.53) | 3.03  (1.26 to 6.31) | -0.16  (-0.36 to 0.05) | 0.129 |
|  | Italy | 2.67  (0.86 to 6.37) | 2.57  (0.83 to 6.01) | -0.14  (-0.18 to -0.1) | <0.001 | 2.50  (1.04 to 5.01) | 2.50  (1.04 to 5.02) | 0  (-0.02 to 0.03) | 0.833 |
|  | Jamaica | 13.99  (4.63 to 32.91) | 13.71  (4.58 to 31.85) | -0.07  (-0.17 to 0.02) | 0.142 | 13.07  (5.39 to 26.86) | 10.72  (4.49 to 22.15) | -0.64  (-0.76 to -0.51) | <0.001 |
|  | Japan | 6.56  (2.11 to 15.56) | 6.37  (2.04 to 15.09) | -0.1  (-0.14 to -0.05) | <0.001 | 2.46  (1.04 to 5.01) | 2.54  (1.07 to 5.24) | 0.1  (0.03 to 0.16) | 0.005 |
|  | Jordan | 4.02  (1.29 to 9.54) | 3.73  (1.16 to 8.80) | -0.25  (-0.33 to -0.17) | <0.001 | 4.10  (1.70 to 8.29) | 4.08  (1.65 to 8.42) | -0.03  (-0.09 to 0.03) | 0.397 |
|  | Kazakhstan | 12.14  (3.99 to 28.53) | 11.72  (3.83 to 27.43) | -0.12  (-0.15 to -0.09) | <0.001 | 2.85  (1.12 to 5.86) | 2.87  (1.15 to 5.82) | 0.02  (-0.02 to 0.06) | 0.287 |
|  | Kenya | 21.24  (7.05 to 49.69) | 26.88  (8.91 to 62.32) | 0.72  (0.58 to 0.85) | <0.001 | 10.25  (4.36 to 20.73) | 10.28  (4.34 to 20.72) | 0  (-0.07 to 0.06) | 0.979 |
|  | Kiribati | 27.88  (9.53 to 64.25) | 27.02  (8.86 to 61.72) | -0.11  (-0.13 to -0.09) | <0.001 | 5.01  (2.03 to 10.15) | 5.11  (2.06 to 10.64) | 0.07  (0.02 to 0.12) | 0.009 |
|  | Kuwait | 4.29  (1.37 to 10.18) | 3.93  (1.22 to 9.17) | -0.3  (-0.42 to -0.19) | <0.001 | 3.85  (1.56 to 8.06) | 3.84  (1.57 to 7.74) | -0.01  (-0.07 to 0.06) | 0.837 |
|  | Kyrgyzstan | 12.23  (4.08 to 28.70) | 11.77  (3.87 to 27.19) | -0.13  (-0.18 to -0.08) | <0.001 | 2.83  (1.13 to 5.81) | 2.86  (1.13 to 5.95) | 0.02  (-0.03 to 0.08) | 0.388 |
|  | Lao People's Democratic Republic | 9.94  (3.33 to 23.18) | 9.30  (3.07 to 22.04) | -0.21  (-0.23 to -0.18) | <0.001 | 5.23  (2.13 to 10.72) | 5.25  (2.10 to 10.85) | 0.01  (-0.02 to 0.04) | 0.549 |
|  | Latvia | 5.81  (1.84 to 13.69) | 5.65  (1.80 to 13.20) | -0.08  (-0.29 to 0.13) | 0.445 | 4.95  (1.96 to 10.42) | 4.95  (1.95 to 10.28) | 0  (-0.01 to 0.01) | 0.753 |
|  | Lebanon | 3.83  (1.18 to 9.20) | 3.49  (1.08 to 8.10) | -0.36  (-0.67 to -0.05) | 0.023 | 3.83  (1.58 to 7.93) | 3.83  (1.55 to 7.83) | 0  (-0.06 to 0.05) | 0.875 |
|  | Lesotho | 36.33  (12.68 to 82.99) | 35.09  (12.14 to 79.30) | -0.12  (-0.16 to -0.09) | <0.001 | 11.60  (4.80 to 24.17) | 12.82  (5.38 to 26.68) | 0.32  (0.3 to 0.35) | <0.001 |
|  | Liberia | 23.68  (7.86 to 53.21) | 22.82  (7.56 to 52.10) | -0.12  (-0.17 to -0.08) | <0.001 | 8.64  (3.52 to 17.94) | 8.73  (3.60 to 17.80) | 0.03  (-0.01 to 0.08) | 0.133 |
|  | Libya | 3.94  (1.25 to 9.23) | 3.56  (1.11 to 8.46) | -0.36  (-0.58 to -0.14) | 0.001 | 3.85  (1.56 to 7.78) | 3.83  (1.61 to 7.88) | -0.02  (-0.08 to 0.04) | 0.468 |
|  | Lithuania | 5.71  (1.87 to 13.53) | 5.65  (1.78 to 13.38) | -0.04  (-0.21 to 0.14) | 0.693 | 4.93  (1.98 to 10.11) | 4.98  (1.99 to 10.37) | 0.03  (0.01 to 0.05) | 0.004 |
|  | Luxembourg | 2.95  (0.94 to 7.12) | 2.85  (0.90 to 6.71) | -0.11  (-0.18 to -0.05) | 0.001 | 3.17  (1.35 to 6.46) | 3.17  (1.28 to 6.54) | 0  (-0.02 to 0.01) | 0.629 |
|  | Madagascar | 29.05  (9.94 to 66.59) | 28.32  (9.76 to 64.38) | -0.08  (-0.12 to -0.05) | <0.001 | 10.18  (4.15 to 20.83) | 10.29  (4.27 to 20.99) | 0.03  (-0.01 to 0.08) | 0.165 |
|  | Malawi | 17.60  (5.77 to 41.19) | 16.95  (5.59 to 39.71) | -0.18  (-0.42 to 0.06) | 0.148 | 13.00  (5.42 to 26.00) | 11.87  (4.95 to 24.56) | -0.31  (-0.37 to -0.25) | <0.001 |
|  | Malaysia | 9.46  (3.07 to 21.96) | 8.97  (2.93 to 21.40) | -0.17  (-0.24 to -0.1) | <0.001 | 5.22  (2.10 to 10.73) | 5.23  (2.12 to 10.57) | -0.01  (-0.01 to 0) | 0.113 |
|  | Maldives | 9.50  (3.15 to 22.36) | 8.92  (2.93 to 21.13) | -0.21  (-0.27 to -0.15) | <0.001 | 5.22  (2.06 to 10.72) | 5.21  (2.10 to 10.73) | -0.01  (-0.02 to 0) | 0.143 |
|  | Mali | 36.05  (12.61 to 81.22) | 35.69  (12.52 to 80.49) | -0.05  (-0.22 to 0.11) | 0.519 | 13.20  (5.64 to 27.05) | 10.61  (4.41 to 21.86) | -0.68  (-0.71 to -0.65) | <0.001 |
|  | Malta | 3.01  (0.98 to 7.21) | 2.89  (0.92 to 6.84) | -0.14  (-0.24 to -0.03) | 0.012 | 3.18  (1.30 to 6.53) | 3.16  (1.30 to 6.57) | -0.02  (-0.02 to -0.01) | <0.001 |
|  | Marshall Islands | 27.39  (9.20 to 63.52) | 26.27  (8.90 to 59.12) | -0.14  (-0.16 to -0.12) | <0.001 | 5.04  (2.05 to 10.40) | 5.11  (2.08 to 10.70) | 0.05  (0.02 to 0.08) | 0.001 |
|  | Mauritania | 24.43  (8.48 to 56.69) | 23.14  (7.79 to 53.10) | -0.18  (-0.23 to -0.14) | <0.001 | 8.75  (3.62 to 17.82) | 8.85  (3.50 to 17.98) | 0.03  (0.01 to 0.06) | 0.007 |
|  | Mauritius | 9.34  (3.09 to 21.57) | 8.94  (2.93 to 20.75) | -0.14  (-0.17 to -0.11) | <0.001 | 5.21  (2.13 to 10.78) | 5.20  (2.15 to 10.55) | 0  (-0.03 to 0.04) | 0.801 |
|  | Mexico | 21.42  (7.07 to 49.43) | 21.35  (7.05 to 49.24) | -0.03  (-0.08 to 0.03) | 0.306 | 6.64  (2.80 to 13.38) | 6.45  (2.73 to 13.23) | -0.09  (-0.29 to 0.11) | 0.362 |
|  | Micronesia (Federated States of) | 27.98  (9.60 to 63.47) | 26.63  (9.09 to 60.67) | -0.15  (-0.18 to -0.13) | <0.001 | 5.03  (2.03 to 10.26) | 5.11  (2.09 to 10.58) | 0.05  (0 to 0.09) | 0.032 |
|  | Monaco | 2.73  (0.85 to 6.48) | 2.74  (0.84 to 6.59) | 0.01  (-0.05 to 0.07) | 0.684 | 3.19  (1.31 to 6.61) | 3.17  (1.28 to 6.35) | -0.02  (-0.05 to 0.01) | 0.119 |
|  | Mongolia | 12.41  (4.11 to 28.69) | 11.94  (3.90 to 28.11) | -0.13  (-0.17 to -0.09) | <0.001 | 2.83  (1.10 to 6.00) | 2.85  (1.14 to 5.92) | 0.02  (-0.04 to 0.09) | 0.478 |
|  | Montenegro | 7.84  (2.56 to 18.29) | 7.59  (2.47 to 18.22) | -0.09  (-0.23 to 0.05) | 0.226 | 2.09  (0.86 to 4.36) | 2.10  (0.88 to 4.31) | 0.01  (-0.01 to 0.03) | 0.201 |
|  | Morocco | 8.19  (2.64 to 19.50) | 7.74  (2.49 to 18.07) | -0.14  (-0.41 to 0.14) | 0.322 | 6.93  (2.93 to 14.51) | 4.41  (1.78 to 9.00) | -1.42  (-1.5 to -1.34) | <0.001 |
|  | Mozambique | 52.06  (19.22 to 112.98) | 38.25  (13.32 to 86.20) | -1  (-1.06 to -0.94) | <0.001 | 11.95  (5.08 to 24.27) | 12.43  (5.22 to 25.00) | 0.14  (0.1 to 0.18) | <0.001 |
|  | Myanmar | 9.86  (3.31 to 23.20) | 9.25  (3.04 to 21.95) | -0.21  (-0.27 to -0.15) | <0.001 | 5.21  (2.11 to 10.90) | 5.23  (2.17 to 10.73) | 0  (-0.06 to 0.06) | 0.94 |
|  | Namibia | 36.63  (12.58 to 82.63) | 35.17  (12.13 to 79.36) | -0.13  (-0.15 to -0.12) | <0.001 | 11.56  (4.80 to 23.65) | 12.36  (5.12 to 25.50) | 0.21  (0.18 to 0.25) | <0.001 |
|  | Nauru | 25.88  (8.61 to 59.88) | 25.55  (8.59 to 59.98) | -0.04  (-0.05 to -0.02) | <0.001 | 5.05  (2.06 to 10.33) | 5.10  (2.03 to 10.42) | 0.03  (-0.02 to 0.08) | 0.232 |
|  | Nepal | 6.57  (2.15 to 15.53) | 6.16  (1.96 to 14.39) | -0.2  (-0.25 to -0.15) | <0.001 | 2.27  (0.93 to 4.80) | 2.28  (0.92 to 4.74) | 0.01  (-0.03 to 0.05) | 0.652 |
|  | Netherlands | 1.83  (0.55 to 4.28) | 1.73  (0.52 to 4.13) | -0.2  (-0.54 to 0.13) | 0.23 | 3.92  (1.59 to 8.10) | 3.90  (1.58 to 8.17) | -0.02  (-0.09 to 0.05) | 0.624 |
|  | New Zealand | 5.21  (1.61 to 12.28) | 5.03  (1.58 to 12.13) | -0.13  (-0.23 to -0.03) | 0.008 | 2.62  (1.09 to 5.36) | 2.68  (1.10 to 5.53) | 0.08  (0.01 to 0.15) | 0.026 |
|  | Nicaragua | 22.17  (7.44 to 51.11) | 21.13  (7.17 to 48.25) | -0.15  (-0.22 to -0.07) | <0.001 | 9.16  (3.74 to 18.57) | 9.14  (3.80 to 18.53) | 0.01  (0 to 0.01) | 0.022 |
|  | Niger | 24.46  (8.30 to 56.51) | 23.69  (8.03 to 53.46) | -0.12  (-0.15 to -0.08) | <0.001 | 8.73  (3.66 to 17.76) | 8.79  (3.61 to 17.89) | 0.02  (-0.01 to 0.05) | 0.274 |
|  | Nigeria | 19.83  (6.73 to 45.36) | 21.42  (7.24 to 48.94) | 0.25  (0.06 to 0.43) | 0.01 | 9.70  (4.14 to 19.58) | 9.63  (4.06 to 19.44) | -0.03  (-0.09 to 0.04) | 0.459 |
|  | Niue | 25.43  (8.33 to 58.84) | 25.30  (8.55 to 58.40) | -0.03  (-0.08 to 0.03) | 0.321 | 5.06  (1.99 to 10.47) | 5.14  (2.06 to 10.61) | 0.04  (-0.02 to 0.11) | 0.202 |
|  | North Macedonia | 7.81  (2.55 to 18.68) | 7.55  (2.50 to 17.71) | -0.11  (-0.18 to -0.04) | 0.001 | 2.11  (0.86 to 4.41) | 2.10  (0.86 to 4.36) | -0.01  (-0.04 to 0.03) | 0.703 |
|  | Northern Mariana Islands | 26.59  (8.96 to 61.53) | 25.68  (8.57 to 58.80) | -0.11  (-0.16 to -0.07) | <0.001 | 5.03  (2.03 to 10.38) | 5.13  (2.03 to 10.58) | 0.06  (0.01 to 0.12) | 0.028 |
|  | Norway | 2.89  (0.92 to 6.77) | 2.80  (0.88 to 6.62) | -0.11  (-0.12 to -0.09) | <0.001 | 3.87  (1.60 to 7.86) | 3.38  (1.40 to 6.93) | -0.43  (-0.48 to -0.38) | <0.001 |
|  | Oman | 6.60  (2.16 to 15.62) | 6.23  (1.98 to 14.94) | -0.19  (-0.24 to -0.14) | <0.001 | 3.85  (1.54 to 7.80) | 3.83  (1.54 to 7.90) | -0.02  (-0.07 to 0.03) | 0.42 |
|  | Pakistan | 5.85  (1.84 to 13.85) | 5.49  (1.79 to 12.85) | -0.19  (-0.3 to -0.08) | 0.001 | 2.14  (0.90 to 4.35) | 2.15  (0.89 to 4.28) | 0  (-0.05 to 0.05) | 0.961 |
|  | Palau | 25.37  (8.40 to 59.02) | 25.29  (8.46 to 58.83) | -0.01  (-0.02 to 0.01) | 0.582 | 5.05  (2.07 to 10.40) | 5.12  (2.06 to 10.42) | 0.04  (-0.01 to 0.1) | 0.138 |
|  | Palestine | 9.91  (3.37 to 23.42) | 9.77  (3.21 to 23.10) | -0.05  (-0.14 to 0.03) | 0.193 | 3.83  (1.59 to 7.87) | 3.83  (1.54 to 7.91) | 0  (-0.03 to 0.04) | 0.944 |
|  | Panama | 21.71  (7.41 to 50.94) | 21.03  (7.01 to 47.59) | -0.11  (-0.22 to 0.01) | 0.064 | 9.15  (3.82 to 18.86) | 9.21  (3.90 to 19.26) | 0.02  (0.01 to 0.02) | <0.001 |
|  | Papua New Guinea | 32.33  (11.20 to 73.18) | 32.12  (11.08 to 73.62) | -0.04  (-0.13 to 0.05) | 0.402 | 6.18  (2.53 to 12.73) | 6.27  (2.58 to 12.94) | 0.05  (-0.04 to 0.13) | 0.259 |
|  | Paraguay | 15.18  (5.06 to 35.25) | 14.58  (4.78 to 33.78) | -0.13  (-0.17 to -0.09) | <0.001 | 9.05  (3.74 to 18.68) | 9.07  (3.78 to 18.43) | 0.02  (0.01 to 0.03) | <0.001 |
|  | Peru | 10.26  (3.33 to 24.37) | 9.79  (3.15 to 22.97) | -0.15  (-0.19 to -0.11) | <0.001 | 10.54  (4.48 to 21.67) | 10.25  (4.22 to 21.48) | -0.09  (-0.15 to -0.03) | 0.006 |
|  | Philippines | 8.73  (2.87 to 20.39) | 8.27  (2.71 to 19.23) | -0.17  (-0.19 to -0.16) | <0.001 | 4.26  (1.77 to 8.62) | 4.35  (1.84 to 8.83) | 0.07  (0.04 to 0.09) | <0.001 |
|  | Poland | 7.10  (2.31 to 16.63) | 6.82  (2.23 to 15.86) | -0.13  (-0.23 to -0.04) | 0.006 | 1.68  (0.71 to 3.43) | 1.72  (0.72 to 3.51) | 0.03  (-0.04 to 0.11) | 0.391 |
|  | Portugal | 3.66  (1.18 to 8.59) | 3.52  (1.09 to 8.32) | -0.14  (-0.17 to -0.11) | <0.001 | 3.17  (1.30 to 6.45) | 3.18  (1.31 to 6.66) | 0  (-0.04 to 0.05) | 0.889 |
|  | Puerto Rico | 13.78  (4.51 to 31.74) | 13.44  (4.43 to 31.07) | -0.09  (-0.19 to 0.01) | 0.073 | 8.61  (3.56 to 17.65) | 8.75  (3.63 to 17.81) | 0.04  (0.02 to 0.06) | <0.001 |
|  | Qatar | 6.54  (2.11 to 15.45) | 6.18  (1.99 to 14.60) | -0.19  (-0.23 to -0.15) | <0.001 | 3.84  (1.57 to 7.89) | 3.84  (1.55 to 7.95) | 0  (-0.03 to 0.04) | 0.936 |
|  | Republic of Korea | 6.74  (2.17 to 15.73) | 6.25  (2.00 to 14.85) | -0.25  (-0.33 to -0.16) | <0.001 | 9.92  (4.24 to 20.45) | 5.18  (2.06 to 10.73) | -2.06  (-2.19 to -1.94) | <0.001 |
|  | Republic of Moldova | 5.83  (1.84 to 13.77) | 5.77  (1.85 to 13.64) | -0.04  (-0.23 to 0.15) | 0.68 | 4.95  (1.98 to 10.53) | 4.94  (1.96 to 10.15) | -0.01  (-0.06 to 0.04) | 0.826 |
|  | Romania | 7.87  (2.56 to 18.68) | 7.69  (2.51 to 18.10) | -0.07  (-0.11 to -0.03) | <0.001 | 2.09  (0.85 to 4.31) | 2.10  (0.86 to 4.28) | 0  (-0.03 to 0.04) | 0.868 |
|  | Russian Federation | 5.79  (1.87 to 13.67) | 5.88  (1.91 to 13.96) | 0.08  (-0.05 to 0.21) | 0.204 | 4.50  (1.89 to 9.08) | 4.52  (1.88 to 9.10) | 0.01  (0 to 0.03) | 0.027 |
|  | Rwanda | 29.62  (9.96 to 68.14) | 28.18  (9.43 to 64.03) | -0.16  (-0.2 to -0.12) | <0.001 | 9.63  (4.00 to 19.49) | 9.84  (4.14 to 19.95) | 0.07  (0 to 0.14) | 0.047 |
|  | Saint Kitts and Nevis | 12.87  (4.17 to 30.64) | 13.07  (4.19 to 30.66) | 0.03  (0 to 0.07) | 0.07 | 8.59  (3.60 to 17.59) | 8.75  (3.53 to 18.06) | 0.04  (-0.03 to 0.1) | 0.28 |
|  | Saint Lucia | 13.97  (4.69 to 32.38) | 13.66  (4.49 to 32.23) | -0.1  (-0.2 to 0.01) | 0.072 | 8.57  (3.59 to 18.07) | 8.71  (3.60 to 18.14) | 0.05  (0 to 0.11) | 0.061 |
|  | Saint Vincent and the Grenadines | 13.98  (4.58 to 32.65) | 13.81  (4.50 to 32.06) | -0.07  (-0.17 to 0.03) | 0.166 | 8.58  (3.56 to 17.59) | 8.72  (3.63 to 17.93) | 0.04  (-0.01 to 0.09) | 0.109 |
|  | Samoa | 27.68  (9.22 to 62.95) | 26.49  (8.87 to 62.33) | -0.15  (-0.17 to -0.12) | <0.001 | 5.05  (2.05 to 10.39) | 5.12  (2.05 to 10.45) | 0.04  (0.01 to 0.07) | 0.024 |
|  | San Marino | 2.74  (0.84 to 6.29) | 2.75  (0.86 to 6.60) | 0.01  (0.01 to 0.02) | <0.001 | 3.18  (1.29 to 6.42) | 3.17  (1.31 to 6.42) | -0.02  (-0.03 to -0.01) | <0.001 |
|  | Sao Tome and Principe | 24.00  (8.15 to 55.11) | 22.90  (7.67 to 53.44) | -0.17  (-0.2 to -0.14) | <0.001 | 8.80  (3.69 to 18.19) | 8.86  (3.64 to 18.21) | 0.03  (0.01 to 0.04) | <0.001 |
|  | Saudi Arabia | 7.74  (2.55 to 18.03) | 7.30  (2.35 to 17.12) | -0.19  (-0.25 to -0.13) | <0.001 | 3.85  (1.54 to 7.89) | 3.83  (1.59 to 7.89) | -0.01  (-0.06 to 0.04) | 0.64 |
|  | Senegal | 24.28  (8.21 to 56.43) | 23.29  (7.76 to 53.77) | -0.14  (-0.16 to -0.11) | <0.001 | 7.09  (2.93 to 14.54) | 7.07  (2.89 to 14.61) | -0.02  (-0.1 to 0.05) | 0.537 |
|  | Serbia | 7.85  (2.55 to 18.40) | 7.53  (2.39 to 17.48) | -0.13  (-0.19 to -0.07) | <0.001 | 2.09  (0.85 to 4.32) | 2.11  (0.85 to 4.30) | 0.01  (0 to 0.02) | 0.039 |
|  | Seychelles | 9.45  (3.07 to 22.13) | 8.98  (2.88 to 21.75) | -0.17  (-0.2 to -0.14) | <0.001 | 5.21  (2.11 to 10.66) | 5.24  (2.07 to 10.90) | 0.02  (-0.02 to 0.05) | 0.383 |
|  | Sierra Leone | 24.13  (8.15 to 55.54) | 23.11  (7.86 to 53.24) | -0.15  (-0.18 to -0.11) | <0.001 | 8.77  (3.61 to 17.97) | 8.86  (3.75 to 18.24) | 0.03  (0.01 to 0.05) | 0.005 |
|  | Singapore | 6.60  (2.13 to 15.74) | 6.27  (2.03 to 14.79) | -0.18  (-0.22 to -0.13) | <0.001 | 3.81  (1.59 to 7.81) | 3.83  (1.54 to 7.91) | 0.03  (0 to 0.06) | 0.048 |
|  | Slovakia | 7.77  (2.50 to 18.51) | 7.51  (2.40 to 17.78) | -0.12  (-0.22 to -0.03) | 0.013 | 2.10  (0.86 to 4.40) | 2.10  (0.88 to 4.35) | 0  (-0.04 to 0.04) | 0.943 |
|  | Slovenia | 7.70  (2.49 to 18.11) | 7.40  (2.37 to 17.36) | -0.12  (-0.21 to -0.02) | 0.014 | 2.10  (0.85 to 4.24) | 2.10  (0.87 to 4.32) | 0  (-0.05 to 0.05) | 0.954 |
|  | Solomon Islands | 27.82  (9.42 to 64.01) | 26.63  (9.18 to 61.27) | -0.13  (-0.16 to -0.1) | <0.001 | 5.05  (2.01 to 10.33) | 5.10  (2.04 to 10.26) | 0.03  (0 to 0.06) | 0.08 |
|  | Somalia | 29.87  (10.17 to 68.41) | 28.82  (9.75 to 65.06) | -0.12  (-0.14 to -0.09) | <0.001 | 10.13  (4.17 to 20.50) | 10.23  (4.30 to 20.95) | 0.03  (0.01 to 0.05) | 0.013 |
|  | South Africa | 47.78  (16.75 to 107.19) | 37.33  (12.96 to 84.79) | -0.78  (-1.15 to -0.42) | <0.001 | 11.94  (5.03 to 24.06) | 12.54  (5.29 to 25.45) | 0.18  (0.11 to 0.26) | <0.001 |
|  | South Sudan | 29.51  (10.29 to 65.40) | 28.72  (9.54 to 65.69) | -0.09  (-0.1 to -0.07) | <0.001 | 10.16  (4.18 to 20.89) | 10.32  (4.28 to 20.89) | 0.05  (0.02 to 0.07) | 0.001 |
|  | Spain | 3.00  (0.92 to 7.21) | 2.86  (0.90 to 6.75) | -0.17  (-0.27 to -0.08) | <0.001 | 2.31  (0.95 to 4.75) | 2.48  (1.04 to 5.09) | 0.23  (0.17 to 0.29) | <0.001 |
|  | Sri Lanka | 13.86  (4.63 to 32.68) | 13.48  (4.43 to 31.30) | -0.09  (-0.13 to -0.05) | <0.001 | 5.20  (2.13 to 10.81) | 5.22  (2.06 to 10.87) | 0  (-0.01 to 0.01) | 0.876 |
|  | Sudan | 11.12  (3.65 to 26.19) | 10.81  (3.61 to 25.87) | 0.09  (-0.24 to 0.42) | 0.593 | 3.81  (1.59 to 7.80) | 3.83  (1.57 to 7.91) | 0.02  (-0.01 to 0.05) | 0.272 |
|  | Suriname | 14.33  (4.73 to 33.28) | 13.88  (4.65 to 32.86) | -0.13  (-0.22 to -0.04) | 0.003 | 8.58  (3.47 to 17.60) | 8.70  (3.56 to 17.61) | 0.04  (0.01 to 0.06) | 0.005 |
|  | Sweden | 2.95  (0.92 to 7.14) | 2.86  (0.87 to 6.84) | -0.12  (-0.18 to -0.05) | <0.001 | 5.28  (2.15 to 10.64) | 4.77  (1.94 to 9.73) | -0.32  (-0.36 to -0.28) | <0.001 |
|  | Switzerland | 2.95  (0.93 to 6.99) | 2.87  (0.90 to 6.81) | -0.08  (-0.2 to 0.05) | 0.219 | 3.64  (1.51 to 7.60) | 3.35  (1.39 to 6.74) | -0.28  (-0.35 to -0.2) | <0.001 |
|  | Syrian Arab Republic | 6.74  (2.20 to 16.19) | 6.40  (2.03 to 15.08) | -0.16  (-0.27 to -0.06) | 0.002 | 2.97  (1.21 to 6.08) | 3.03  (1.23 to 6.15) | 0.06  (0.01 to 0.1) | 0.014 |
|  | Taiwan (Province of China) | 6.73  (2.42 to 14.97) | 6.96  (2.26 to 16.47) | 0.12  (0.05 to 0.2) | 0.001 | 3.43  (1.40 to 7.07) | 3.42  (1.38 to 7.20) | -0.01  (-0.04 to 0.02) | 0.686 |
|  | Tajikistan | 12.56  (4.17 to 29.05) | 12.05  (3.93 to 27.89) | -0.13  (-0.21 to -0.05) | 0.001 | 2.84  (1.15 to 5.85) | 2.86  (1.16 to 5.94) | 0.03  (-0.02 to 0.08) | 0.305 |
|  | Thailand | 9.47  (3.15 to 21.91) | 8.93  (2.83 to 20.95) | -0.17  (-0.21 to -0.12) | <0.001 | 7.92  (3.40 to 16.41) | 6.89  (2.77 to 14.16) | -0.48  (-0.67 to -0.28) | <0.001 |
|  | Timor-Leste | 9.95  (3.28 to 23.36) | 9.29  (2.99 to 21.87) | -0.22  (-0.26 to -0.18) | <0.001 | 5.22  (2.11 to 10.79) | 5.24  (2.12 to 10.51) | 0.01  (-0.01 to 0.04) | 0.405 |
|  | Togo | 16.01  (5.33 to 37.59) | 15.01  (4.88 to 35.07) | -0.22  (-0.38 to -0.06) | 0.007 | 8.75  (3.62 to 17.98) | 8.87  (3.65 to 18.53) | 0.04  (0 to 0.08) | 0.079 |
|  | Tokelau | 25.52  (8.33 to 57.96) | 25.27  (8.44 to 59.31) | -0.03  (-0.05 to -0.02) | <0.001 | 5.04  (2.01 to 10.30) | 5.12  (2.06 to 10.48) | 0.05  (0.01 to 0.09) | 0.01 |
|  | Tonga | 27.08  (9.31 to 62.69) | 26.17  (8.76 to 59.33) | -0.12  (-0.14 to -0.09) | <0.001 | 5.02  (2.00 to 10.32) | 5.12  (2.05 to 10.48) | 0.06  (0 to 0.12) | 0.071 |
|  | Trinidad and Tobago | 13.89  (4.72 to 32.96) | 13.73  (4.56 to 32.50) | -0.06  (-0.21 to 0.1) | 0.473 | 8.60  (3.50 to 17.55) | 8.75  (3.51 to 17.94) | 0.05  (0 to 0.1) | 0.072 |
|  | Tunisia | 10.56  (3.52 to 25.17) | 10.08  (3.30 to 23.87) | -0.15  (-0.36 to 0.06) | 0.168 | 3.84  (1.57 to 7.99) | 3.84  (1.55 to 7.97) | 0  (-0.03 to 0.04) | 0.83 |
|  | Turkmenistan | 12.31  (3.99 to 28.55) | 11.80  (3.88 to 27.51) | -0.13  (-0.17 to -0.09) | <0.001 | 2.86  (1.14 to 5.83) | 2.85  (1.14 to 5.97) | -0.04  (-0.07 to -0.01) | 0.014 |
|  | Tuvalu | 25.54  (8.51 to 59.68) | 25.46  (8.66 to 58.86) | -0.02  (-0.03 to 0) | 0.126 | 5.04  (1.99 to 10.32) | 5.13  (2.08 to 10.57) | 0.06  (0 to 0.12) | 0.071 |
|  | Türkiye | 3.92  (1.22 to 9.29) | 3.57 (1.11 to 8.43) | 0.04 (-1.47 to 1.58) | 0.956 | 3.40  (1.39 to 6.97) | 3.27  (1.36 to 6.62) | -0.16 (-0.24 to -0.07) | <0.001 |
|  | Uganda | 26.61  (9.07 to 61.26) | 25.90  (8.54 to 59.97) | -0.11  (-0.34 to 0.13) | 0.384 | 16.67  (7.17 to 33.94) | 12.25  (5.13 to 24.99) | -1.02  (-1.17 to -0.87) | <0.001 |
|  | Ukraine | 5.81  (1.82 to 13.86) | 5.78  (1.82 to 13.57) | -0.03  (-0.21 to 0.15) | 0.755 | 4.52  (1.82 to 9.47) | 4.52  (1.84 to 9.23) | 0  (-0.04 to 0.03) | 0.978 |
|  | United Arab Emirates | 6.63  (2.14 to 15.53) | 6.22  (1.98 to 14.98) | -0.2  (-0.24 to -0.17) | <0.001 | 3.85  (1.59 to 7.81) | 3.83  (1.55 to 7.86) | -0.02  (-0.05 to 0.01) | 0.21 |
|  | United Kingdom | 3.03  (0.97 to 7.10) | 2.94  (0.94 to 7.00) | -0.1  (-0.13 to -0.07) | <0.001 | 2.73  (1.14 to 5.48) | 2.78  (1.16 to 5.63) | 0.05  (0.01 to 0.08) | 0.004 |
|  | United Republic of Tanzania | 33.15  (11.52 to 74.69) | 25.31  (8.57 to 58.95) | -0.96  (-1.24 to -0.67) | <0.001 | 9.13  (3.79 to 18.65) | 9.39  (3.97 to 19.13) | 0.09  (-0.13 to 0.31) | 0.435 |
|  | United States of America | 13.41  (4.37 to 31.27) | 12.78  (4.15 to 30.25) | -0.16  (-0.21 to -0.11) | <0.001 | 5.74  (2.40 to 11.60) | 4.95  (2.08 to 10.03) | -0.47  (-0.52 to -0.42) | <0.001 |
|  | United States Virgin Islands | 13.96  (4.65 to 32.62) | 13.56  (4.45 to 32.32) | -0.11  (-0.21 to 0) | 0.042 | 8.64  (3.55 to 17.49) | 8.75  (3.63 to 18.20) | 0.02  (-0.01 to 0.04) | 0.144 |
|  | Uruguay | 6.23  (2.01 to 15.00) | 5.89  (1.90 to 13.88) | -0.18  (-0.23 to -0.12) | <0.001 | 7.93  (3.31 to 16.45) | 7.95  (3.28 to 16.25) | 0.01  (-0.02 to 0.03) | 0.481 |
|  | Uzbekistan | 12.21  (3.98 to 28.51) | 11.71  (3.89 to 26.75) | -0.12  (-0.17 to -0.07) | <0.001 | 2.83  (1.14 to 5.86) | 2.85  (1.17 to 5.78) | 0.03  (-0.04 to 0.1) | 0.351 |
|  | Vanuatu | 27.97  (9.23 to 64.83) | 26.85  (9.00 to 62.08) | -0.14  (-0.15 to -0.12) | <0.001 | 4.04  (1.60 to 8.34) | 4.14  (1.69 to 8.46) | 0.07  (0 to 0.14) | 0.05 |
|  | Venezuela (Bolivarian Republic of) | 21.85  (7.24 to 50.88) | 21.11  (7.16 to 49.81) | -0.1  (-0.13 to -0.07) | <0.001 | 9.17  (3.80 to 18.74) | 9.17  (3.75 to 18.52) | 0  (-0.01 to 0.02) | 0.686 |
|  | Viet Nam | 6.44  (2.10 to 15.21) | 5.82  (1.93 to 13.92) | -0.36  (-0.47 to -0.25) | <0.001 | 4.83  (1.93 to 9.79) | 4.90  (1.98 to 10.12) | 0.03  (0 to 0.07) | 0.072 |
|  | Yemen | 6.84  (2.20 to 16.12) | 6.52  (2.08 to 15.48) | -0.16  (-0.21 to -0.12) | <0.001 | 3.82  (1.55 to 7.78) | 3.81  (1.59 to 7.92) | 0  (-0.04 to 0.04) | 0.954 |
|  | Zambia | 41.32  (14.36 to 92.50) | 41.39  (14.77 to 94.31) | -0.03  (-0.22 to 0.16) | 0.758 | 11.19  (4.67 to 22.78) | 11.51  (4.75 to 23.08) | 0.07  (-0.02 to 0.17) | 0.12 |
|  | Zimbabwe | 39.17  (13.79 to 89.18) | 39.28  (13.39 to 88.30) | -0.02  (-0.1 to 0.05) | 0.543 | 11.59  (4.84 to 23.49) | 12.35  (5.18 to 25.12) | 0.19  (0.08 to 0.3) | <0.001 |

**Abbreviations:** DALYs, Disability-Adjusted Life Years; AAPC, average annual percent change; CI, confidence interval.
